# Complete mitochondrial genomes of Thai and Lao populations indicate an ancient origin of Austroasiatic groups and demic diffusion in the spread of Tai–Kadai languages

**DOI:** 10.1007/s00439-016-1742-y

**Published:** 2016-11-11

**Authors:** Wibhu Kutanan, Jatupol Kampuansai, Metawee Srikummool, Daoroong Kangwanpong, Silvia Ghirotto, Andrea Brunelli, Mark Stoneking

**Affiliations:** 1 Department of Biology, Faculty of Science, Khon Kaen University, Mittapap Road, Khon Kaen, 40002 Thailand; 2 Department of Evolutionary Genetics, Max Planck Institute for Evolutionary Anthropology, Deutscher Platz 6, 04103 Leipzig, Germany; 3 Department of Biology, Faculty of Science, Chiang Mai University, Chiang Mai, 50202 Thailand; 4 Department of Biochemistry, Faculty of Medical Science, Naresuan University, Phitsanulok, 65000 Thailand; 5 Department of Life Science and Biotechnology, University of Ferrara, 44121 Ferrara, Italy

## Abstract

The Tai–Kadai (TK) language family is thought to have originated in southern China and spread to Thailand and Laos, but it is not clear if TK languages spread by demic diffusion (i.e., a migration of people from southern China) or by cultural diffusion, with native Austroasiatic (AA) speakers switching to TK languages. To address this and other questions, we obtained 1234 complete mtDNA genome sequences from 51 TK and AA groups from Thailand and Laos. We find high genetic heterogeneity across the region, with 212 different haplogroups, and significant genetic differentiation among different samples from the same ethnolinguistic group. TK groups are more genetically homogeneous than AA groups, with the latter exhibiting more ancient/basal mtDNA lineages, and showing more drift effects. Modeling of demic diffusion, cultural diffusion, and admixture scenarios consistently supports the spread of TK languages by demic diffusion.

**Electronic supplementary material:**

The online version of this article (doi:10.1007/s00439-016-1742-y) contains supplementary material, which is available to authorized users.

## Introduction

**Fig. 1:**
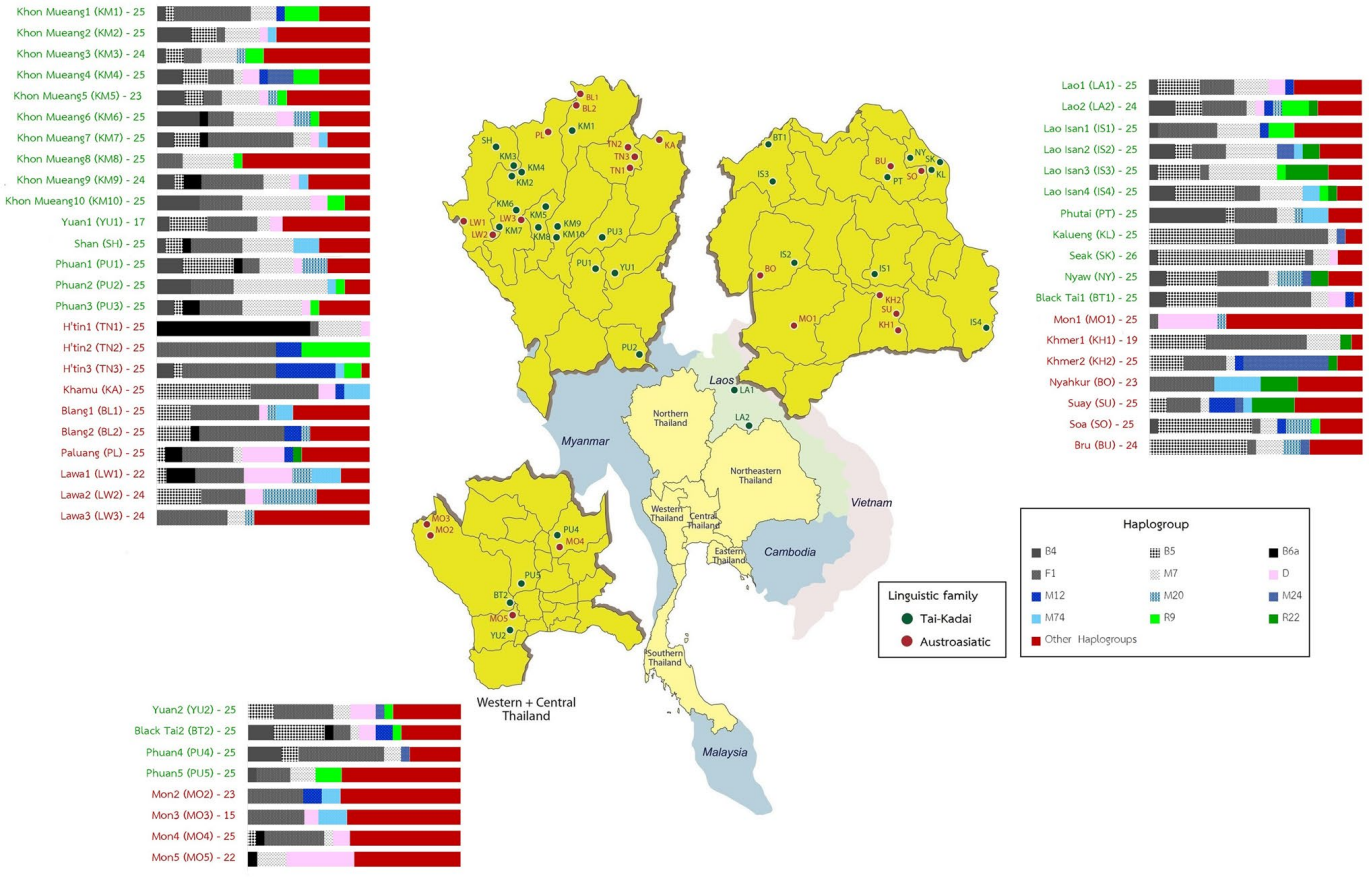
Map showing the geographic locations of the studied populations and their language family affiliation*. Bar plots* illustrate the relative frequency of major haplogroups by population. *Dark* and *white shades* show haplogroups B, F and M7, which are specific to Southeast Asian populations, whereas the remaining haplogroups (D, M12, M20, M24, M74, R9, R22 and other haplogroups) are represented by *various colors*

Thailand and Laos are regarded as the geographical heart of Mainland Southeast Asia (MSEA) (Fig. 1). Archaeological evidence suggests a long history of human occupation of the area, with the oldest human remains dated to 46–63 thousand years ago (kya) from Tam Pa Ling Cave (Demeter et al. 2012), and cultural remains dating to 35–40 kya (Anderson 1990; Shoocondej 2006). A potential role for Thailand/Laos as a corridor between southern China and Island Southeast Asia (ISEA) is further indicated by archaeological evidence for agricultural communities that may have expanded from the center of the Yangtze valley during the Neolithic period (Higham and Higham 2009; Higham 2014).

There is also considerable linguistic diversity, with five language families [Tai–Kadai (TK), Austroasiatic (AA), Sino–Tibetan (ST), Hmong–Mien (HM) and Austronesian (AN)], spoken in the area. Most people speak TK languages (94.40%, in Thailand and 69.60% in Laos), while AA is the second most common language family (4.10% in Thailand and 22.70% in Laos) (Lewis et al. 2016). However, the AA family is more diverse (27 languages in Thailand and 47 languages in Laos) than TK (16 languages in Thailand and 21 languages in Laos). The ST and HM families are concentrated in the area of northern and northwestern Thailand as well as northern and central Laos (ST: 19 languages in Thailand and 11 languages in Laos; HM: 3 languages in Thailand and 4 languages in Laos). The AN family is restricted to southern Thailand with just six languages (Lewis et al. 2016). Both major families (AA and TK) are widespread across Asia; there are 167 AA languages spoken by ~102 million people from South Asia (Bangladesh and India) to southern China and MSEA, including Malaysia; and 92 TK languages spoken by ~80 million people in northeast India, southern China, Vietnam, Myanmar, Cambodia, Thailand and Laos (Lewis et al. 2016). Although the origin and spread of AA is debatable (Chaubey et al. 2011; Diffloth 2005), AA people are generally considered to be descended from the earliest inhabitants of the region (Condominas 1990; Penth 2000). TK is generally considered to have arisen in southeast China prior to 2.5 kya and then spread to SEA between 1 and 2 kya (O’Connor 1995; Pittayaporn 2014).

Although archaeological and linguistic evidence point to an expansion from southern China, physical anthropological studies indicate that the present-day Thai people resemble ancient people (Sangvichien 1966) as well as modern AA people in northern Thailand (Nakbunlung 1994). Therefore, there are two competing hypotheses concerning the origin of the modern Thai/Lao TK people: (1) a demic expansion of people from southern China that brought their genes, culture, and language to Thailand/Laos; or (2) a cultural diffusion from southern China that resulted in native AA people adopting the TK language and culture. This general question of demic vs. cultural diffusion is a long-standing one concerning expansions in other parts of the world, particularly those involving languages and/or agricultural practices, e.g., expansions associated with Indo-European, Bantu, Han and Austronesian languages (Ammerman and Cavalli-Sforza 1994; Battaglia et al. 2009; Chikhi et al. 2002; Diamond and Bellwood 2003; Pakendorf et al. 2011; Peng et al. 2010; Sokal et al. 1991; Wen et al. 2004). While genetic studies have proven to be informative in distinguishing between demic vs. cultural diffusion in these other contexts, to date, genetic studies have not been applied to this question with respect to TK people. In particular, previous mitochondrial (mt) DNA studies on Thai/Lao populations were too limited to address this question via phylogenetic or simulation-based analyses (Bodner et al. 2011; Kutanan et al. 2011, 2014). Therfore, to address the role of demic vs. cultural diffusion in the origins of the TK people as well as to investigate other aspects of Thai/Lao prehistory, we analyze here 1234 complete mtDNA genome sequences from 51 Thai/Laos populations, comprising a comprehensive sampling of TK and AA genetic diversity.

## Methods

### Samples

Blood or buccal samples were collected with informed consent from 1234 unrelated subjects belonging to 51 populations that were classified into 23 ethnolinguistic groups (Fig. 1; Table S1 in Online Resource 1). All groups speak either AA or TK languages and all are from Thailand, with the exception of two populations from Laos.

### MtDNA sequencing and multiple alignment

DNA was isolated as described previously from blood samples (Seielstad et al. 1999) and from buccal cells with the Gentra Puregene Buccal Cell Kit (Qiagen). Sequencing libraries were constructed using a multiplex protocol for the Illumina Genome Analyzer platform (Meyer and Kircher 2010) and were enriched for mtDNA as described previously (Maricic et al. 2010). Several Illumina platforms and lengths of sequencing reads were employed, with post-processing using Illumina software and the Improved Base Identification System (Kircher et al. 2010). The software MIA (Briggs et al. 2009), which is implemented in an in-house sequence assembly–analysis pipeline for calling consensus sequences and detecting mtDNA heteroplasmy (Li and Stoneking 2012), was used to map sequencing reads to the revised Cambridge Reference Sequence (Andrews et al. 1999). A multiple sequence alignment of the sequences and the Reconstructed Sapiens Reference Sequence (RSRS) (Behar et al. 2012) was executed by MAFFT 7.271 (Katoh and Standley 2013).

### Statistical analyses

The aligned sequences were assigned haplogroups using HaploGrep (Kloss-Brandstätter et al. 2010) with PhyloTree mtDNA tree build 17 (van Oven and Kayser 2009). MitoTool was also used to re-check haplogroup assignments (Fan and Yao 2011). The software Arlequin 3.5.1.3 (Excoffer and Lischer 2010) was used for the following analyses: measures of genetic diversity, pairwise genetic distances (*Φ*_st_, pairwise difference), analysis of molecular variance (AMOVA) and a Mantel test comparing genetic and geographic distances between populations; for the latter, we computed three types of geographic distance, i.e. great-circle distance, least cost path distance, and resistance distance. The great-circle distance matrix was generated by Geographic Distance Matrix Generator v 1.2.3 (Ersts 2006) and the other two distance matrices were computed by the functions *costDistance* in the package gdistance (van Etten 2012) and using CIRCUITSCAPE (McRae 2006) based on a constructed cost-surface raster, respectively. To create this cost-surface raster, briefly, R 3.2.0 was employed using the function *mosaic* from the package raster (Hijmans and Van Etten 2013) to merge two data, i.e. a 30-s elevation grid generated from the WorldClim database (Hijmans et al. 2005) and vector files containing major rivers in Thailand and Laos obtained from Natural Earth. Then, a cost-surface raster was reclassified with parameters known to affect human movements, e.g., mountain, terrain and river (Tassi et al. 2015).

Nonparametric multidimensional scaling (MDS) analysis (based on *Φ*_st_ values) as well as correspondence analysis (CA) using haplogroup counts were constructed using STATISTICA 10.0 (StatSoft, Inc., USA).

BEAST 1.8 was used to construct Bayesian skyline plots (BSP) by population and maximum clade credibility (MCC) trees by haplogroup, based on Bayesian Markov chain Monte Carlo (MCMC) analyses. The software jModel test 2.1.7 (Darriba et al. 2012) was employed to choose the most suitable model during creation of the input file of BEAST by BEAUTi v1.8 (Drummond et al. 2012). BSP calculations were conducted with the data partitioned between coding and noncoding regions with respective mutation rates of 1.708 × 10^−8^ and 9.883 × 10^−8^ (Soares et al. 2009). Tracer 1.6 was used to visualize the BSP plot. For the Bayesian MCMC estimates (BE) and credible intervals (CI) of haplogroup coalescence times, the RSRS was employed to root the mtDNA tree. The Bayesian MCC trees from the BEAST runs were assembled with TreeAnnotator and drawn with FigTree v 1.4.0. To check clustering of sequences by haplogroup, median-joining networks without pre- or post-processing steps were constructed by Network 4.11 and visualized in Network publisher 1.3.0.0 (Fluxus Technology, http://www.fluxus-engineering.com). Contour maps are generated by Golden Software Surfer 10.0 (Golden Software Inc., USA).

The newly generated 1234 mtDNA sequences were compared with a reference data set comprising 2129 Asian mtDNA genomes representing 62 populations retrieved from the literature (Table S2 in Online Resource 1). A neighbor-joining (NJ) tree (Saitou and Nei 1987) based on the *Φ*_st_ distances was generated by MEGA 7 (Kumar et al. 2016).

The analysis of approximate Bayesian computation (ABC) was employed to choose the best-supported hypothesis about the maternal origins of the Thai and Laotian populations. Owing to the different local histories specific to each region, three different mtDNA data sets from the TK and AA as well as a priori parameters (e.g. divergence times) was used in the simulation process. As the origin time of prehistorical TK-speaking groups is unknown, we employed the existing time of the Tai in southern China of ~3 kya, similar to a previous study (Sun et al. 2013). Then, some prehistorical TK groups started to separate from their common ancestor with the Chinese Dai from their homeland in southern China and spread southward to the area of present-day Thailand in the last 1–2 kya (O’Connor 1995; Penth 2000; Pittayaporn 2014). Some TK groups finally reached northern Thailand where LW groups are native inhabitants and founded their kingdom, named Lanna around the end of the thirteenth century A.D. (Condominas 1990). The KM people, the majority of northern Thai, are either genetically from LW groups or admixed with them, and, thus, should originate at this time. We, therefore, conduct the first analysis by pooling ten KM populations (KM1-KM10) as well as combining the three AA-speaking Lawa groups (LW1–LW3) and using the Xishuanbanna Dai as a representative of the Tai source from southern China (Diroma et al. 2014). Although, nowadays, the IS and LA people constitute the vast majority of populations in northeastern Thailand and Laos, respectively, both of them share ethnic identity, and the historical motherland of Lao Isan is in Laos (Schliesinger 2001). Allowing for the differences in both routes of migration and times of prehistorical TK groups, the migration from further north to the area of present-day Lao would have met the KH groups, one of the predominant AA people in SEA, who established the Angorian state around 1.2 kya (Higham 2014). In addition, SU, KA, BU and SO are the other AA groups distributed in the area of present-day Laos whose ancestors could have interacted with TK groups. In the second analysis, therefore, the Xishuanbanna Dai is utilized as the Tai sources, while all AA groups (KH1–KH2, SU, KA, BU, and SO) are combined and the TK-speaking Lao groups (LA1–LA2 and IS1–IS4) are pooled. In the last analysis, we focus on the IS, as they are a Lao group who recently migrated to northeastern Thailand, approximately 250 ya; evidence of biculturalism between KH and IS in northeastern Thailand has been recorded (Vail 2007). One potential scenario was that the IS (IS1–IS4) diverged from the LA (LA1–LA2) without any genetic contact with the KH (KH1–KH2); a second scenario is that IS did admix with KH after diverging from LA. Although an origin of IS from KH is unlikely, we also investigated this scenario.

The simulated data sets were generated by the software package ABCtoolbox (Wegmann et al. 2010). The posterior probabilities were calculated by employing two different approaches, acceptance–rejection procedure (AR) (Pritchard et al. 1999) and weighted multinomial logistic regression (LR) (Beaumont 2008). The former approach considers only a certain number of “best” simulations, and then simply counts the proportion of those retained simulations that were generated by each investigated model. After a few hundred simulations, an excellent fit with the observed data indicates that this approach is reliable (Beaumont 2008), and therefore, 100, 200 and 500 of the best simulations were used in this analysis. According to the latter approach, a logistic regression is fitted where the model is the categorical dependent variable and the summary statistics are the predictive variables. The regression is local around the vector of observed summary statistics, and at the point equivalent to the observed vector of summary statistics, the probability of each model is estimated. Maximum likelihood was used to evaluate the *β* coefficients of the regression, considering different numbers of retained simulations (50,000, 100,000 and 150,000). The posterior probabilities for each model were calculated by the modified R scripts (http://code.google.com/p/popabc/source/browse/#svn%2Ftrunk%2Fscripts). The following summary statistics were employed: the number of haplotypes, haplotype diversity, total number of segregating sites, number of private segregating sites, Tajima’s D, and mean number of pairwise differences for each population, as well as mean number of differences between pairs of populations and pairwise *Φ*_st_. The distribution of simulated data under different models with respect to the observed data was evaluated by a visual inspection of a principal component analysis (PCA) of the best 1000 (or 5000) simulations for each model, using the PCA function implemented in the R package FactoMineR (Husson et al. 2007).

The power to infer the correct model in all tests was estimated by generating 1000 pseudo-observed data sets according to each analyzed model, with parameter values randomly chosen from the corresponding prior distribution. These pseudo-observed datasets were examined along with the same ABC framework applied in the model selection (i.e., with logistic regression and 50,000 retained simulations). Three different sets of models were considered separately. For each model, we evaluated the proportion of cases where the true model was correctly chosen (i.e., true positives) as well as the proportion of cases where the model selection procedure assigned the highest support to one of the other two tested models (i.e., false positives), considering a posterior probability threshold of 0.5 to assign the support.

## Results

### Genetic diversity is higher in TK than in AA groups

For the 1234 mtDNA genome sequences obtained (GenBank under accession numbers KX456435–KX457668), there are 761 distinct sequences (haplotypes) belonging to 212 haplogroups (Table S3 in Online Resource 1). Details concerning sequencing results and sequence coverage are provided in Online Resource 2. The summary statistics for the genetic diversity in each population are provided in Table S1. Haplotype diversity (*h*) ranges from 1.00 in the LA2 (see Fig. 1 for population locations and population abbreviations) to 0.80 in the TN2 group. The SK, BO and TN1 groups also exhibit *h* values somewhat lower than the remaining populations; the same trend is observed for haplogroup diversity, as relatively large values are observed in almost all populations except in TN1, TN2, SK and BO. Both nucleotide diversity (*π*) and mean number of pairwise differences (MPD) are also the lowest in the TN1 group (0.0013 and 21.41, respectively), while the largest values are observed in the MO2 group (0.0026 and 42.6, respectively).

Haplotype and haplogroup diversity values as well as the number of segregating sites are significantly higher for TK than for AA groups (Mann–Whitney *U* tests: *h*: *Z* = 3.34, *P* = 0.0008, haplogroup diversity: *Z* = 3.53, *P* = 0.0004, number of segregating site: *Z* = 2.85, *P* = 0.0044). However, the *π* values of AA groups are not significantly different from those of the TK groups (*Z* = 1.45, *P* = 0.15).

### Greater genetic heterogeneity of AA groups

**Fig. 2:**
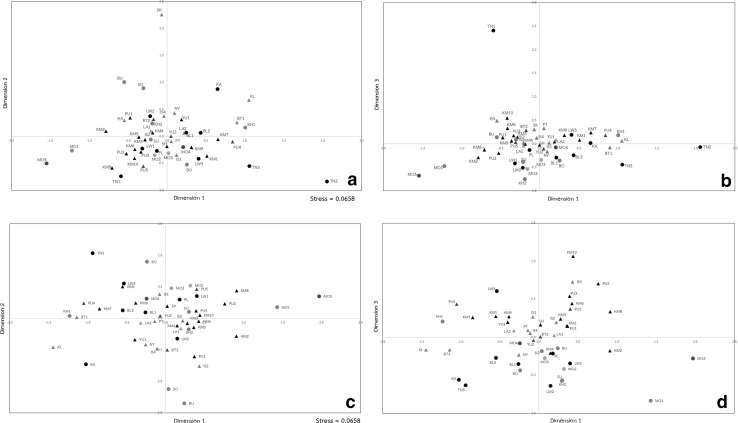
The MDS plot of dimension 1 vs. dimension 2 (**a**, **c**) and dimension 1 vs. dimension 3 (**b**, **d**) based on the *Φ*_st_ genetic distance matrix among the entire set of 51 populations (**a**, **b**) and after removal of three outliers, namely TN1, TN2 and SK (**c**, **d**). Population abbreviations are provided in Fig. 1. *Triangles* and *circles* represent TK- and AA-speaking populations, respectively. *Black*, *red*, *dark blue* and *pink* colors indicate North, Northeastern, Central and West geographic regions of Thailand respectively; *green* indicates the two Lao populations

The MDS analysis (Fig. 2a, b) revealed that in the third dimension, AA and TK groups tended to be separated; this separation was more apparent when three outliers were excluded (Fig. 2c, d). The CA analysis based on haplogroup frequencies (Fig. S1 in Online Resource 3) indicates that specific haplogroups are associated with the populations showing relatively high levels of genetic differentiation, namely: haplogroup B6a in TN1; haplogroup M12a1a in TN3; haplogroup F1a1a in TN2 and BO; and haplogroup B5a1d in SK and KA. Overall, the MDS and CA analyses revealed greater genetic heterogeneity among AA than TK groups. This result is supported by AMOVA (Table 1), as 11.44% of the variance is among AA populations, compared to 4.74% for the TK populations. However, neither linguistic nor geographic classifications of the populations provide a good match to the underlying genetic structure of the Thai/Laos populations, as in all such classifications, the among-population component of the variance is higher than the among-group component (Table 1). Moreover, the Mantel test for the correspondence between genetic and geographic distances between populations is not significant in all types of geographic distances tested (great-circle distance: *r* = 0.03, *P* = 0.31, least cost path distance: *r* = 0.04, *P* = 0.30 and resistance distance: *r* = −0.65, *P* = 0.75). Thus, the genetic structure of the Thai/Laos populations is more complicated than would be predicted from either linguistics or geography.

**Table 1:** Analysis of molecular variance (AMOVA) results

Grouping	Number of groups	Percent variation		
		Among groups	Among population (within group)	Within population
Geography				
Geography 1^a^	5	0.07	7.63**	92.3**
Geography 2^b^	4	0.36	7.77**	91.86**
Northern Thailand	1	–	7.76**	92.24
Northeastern Thailand	1	–	8.69**	91.31
Central Thailand	1	–	6.83**	93.17
Western Thailand	1	–	−0.43	100.43
Laos	1	–	0.66**	99.34
Language				
Language 1^c^	2	0.49*	7.42**	92.1**
Language 2^d^	6	2.56**	6.01**	91.43**
Language 3^e^	10	2.42**	5.68**	91.9**
Austroasiatic	1	–	11.44**	88.56
Tai–Kadai	1	–	4.74**	95.26
Ethnicity				
Mon	1	–	7.1**	92.9
H’tin	1	–	25.71**	74.29
Lawa	1	–	7.78**	92.22
Khmer	1	–	11.10**	88.90
Khon Mueang	1	–	3.43**	96.57
Lao Isan	1	–	2.31**	97.69
Phuan	1	–	5.29**	94.71

* Significant at 0.05 level; ** significant at 0.01 level

^a^Geography 1: Northern Thailand, Northeastern Thailand, Central Thailand, Western Thailand, Laos)

^b^Geography 2: (Northern Thailand, Northeastern Thailand, Central Thailand, Western Thailand)

^c^Language 1: (Austroasiatic, Tai–Kadai)

^d^Language 2: (Northern Tai, Southwestern Tai, Monic, Southern Monic, Eastern Mon-Khmer, Northern Mon–Khmer)

^e^Language 3: (Northern Tai, Chiang Saen, Lao–Phutai, Northwestern Tai, Monic, Southern Monic, Palaungic, Khmuic, Khmer, Katuic)

Greater genetic homogeneity among the TK populations was also reflected in the haplotype sharing analysis (Table S4 in Online Resource 1), which showed that they shared more haplotypes than the AA populations. In particular, the various KM populations shared a number of haplotypes, as did the PU populations, indicating some recent genetic exchange/ancestry among populations within the same ethnolinguistic group. The highest number of shared haplotypes is five, which are shared among the KM5–KM6 and PU2–PU4 groups. Many haplotypes in the PU are shared with almost all of the other TK populations. Among the AA populations, despite the relatively large genetic differences between the TN2 and TN3 populations, they share four haplotypes. Overall, only four populations (IS3, SK, MO1 and MO4) did not share any haplotypes with any other population.

### Significant genetic differentiation within ethnolinguistic groups

Surprisingly, we observed striking and significant genetic differences between populations classified as the same ethnolinguistically but sampled from different locations. This can be seen in the MDS analysis (Fig. 2a, b), in which two of the three most extreme outliers are from the same ethnolinguistic group, namely two of the three AA-speaking H’tin groups, TN1 and TN2 (the third outlier is the SK, a TK-speaking group from northeastern Thailand). In fact, the MDS analysis shows that in many cases, populations from the same ethnolinguistic group are not genetically similar. This is further indicated by an AMOVA for each separate ethnolinguistic group that was sampled from multiple locations (Table 1); in all such instances, the among-populations variance component is significantly different from zero. This unexpected high degree of heterogeneity within the same ethnolinguistic group contributes to the lack of correspondence between the genetic structure of the Thai/Laos populations and their geographic/linguistic relationships.

### Relationships with other Asian populations

**Fig. 3:**
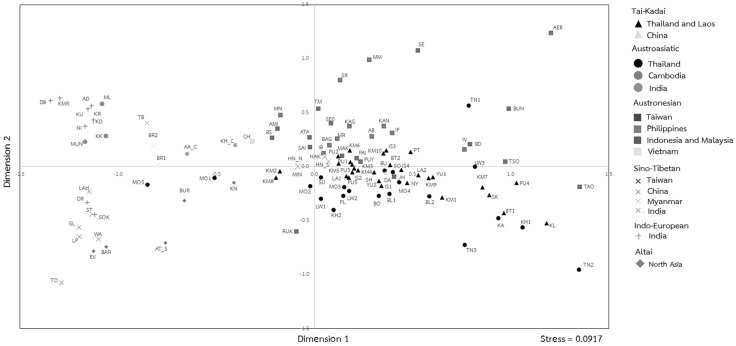
The MDS plot of dimension 1 vs. dimension 2 based on *Φ*_st_ genetic distance matrix from mtDNA genomes among the presently studied populations and other populations from the literature. Population abbreviations are provided in Fig. 1 and Table S2

The genetic relationships of 113 Asian populations (51 from the current study and 62 from the literature; Table S2), as revealed by MDS analysis, indicated, in general, population clustering by both language family and macro-geographic scale (Fig. 3). The SEA populations who speak AN, AA and TK languages are largely separated from North and South Asian populations. The AN and AA groups are further differentiated by the second dimension with the intermediate position of the TK populations among them. These results are also seen in the NJ tree, with the East Asian populations separated from the North and South Asian populations (Fig. S2 in Online Resource 3). Most of the AN groups from Taiwan, Philippines, and Island Southeast Asia (ISEA) are separated from the Thailand TK and AA populations. The TK and AA populations are mostly intermingled with a few AN populations also clustering with them. Overall, TK and AA populations are close to AN population in both MDS (Fig. 3) and NJ tree (Fig. S2). Among the presently studied populations, again, the TN1, TN2 and SK are extremely divergent (in keeping with their relatively low amounts of genetic diversity), but they, nonetheless, cluster with their neighbors from Thailand. There is also a clear division in the AA populations: MO1 and MO5 show affinities with populations from Myanmar and India, reflecting their genetic relatedness (Fig. 3), and are distinct from the other Mon and the other Thai populations. This could reflect either common ancestry of MO1 and MO5 with groups from Myanmar and India and/or gene flow. Surprisingly, even though the two Khmer populations (KH1 and KH2) from northeastern Thailand have close geographic proximity and shared haplotypes, they are genetically distinct from one another and from an ethnolinguistically related group, the Cambodian Khmer (KH_C).

### mtDNA lineages

**Fig. 4:**
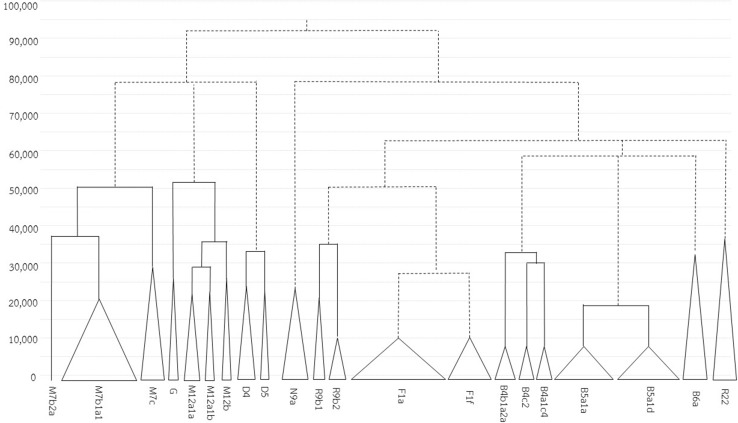
Schematic Bayesian MCMC tree of the major haplogroups found in this study. Bayesian maximum clade credibility trees were constructed for each haplogroup with parameters as described in the “Methods” and then manually combined (*dashed lines*) based on PhyloTree mtDNA tree Build 17. The full Bayesian maximum clade credibility tree for each haplogroup is shown in Fig. S3

The above population relationships are based on analyses of the entire set of mtDNA sequences; additional insights come from considering the distribution and other characteristics of specific haplogroups. Among the 1234 mtDNA genomes belonging to 212 haplogroups, F1 is by far the predominant lineage (21.80%), followed by B5 (13.13%), M7 (11.02%) and B4 (6.00%) (Fig. 1). All of these haplogroups are common in SEA populations and predominate in most of the studied populations, with the exception of two TK (KM8 and PU5) and 12 AA (PL, LW1–LW3, KH2, BO, SU and MO1–MO5) populations (Fig. 1). Haplogroup coalescence times using BE and CI by haplogroup are shown in Table 2. A schematic phylogeny of the main haplogroups, based on Bayesian MCMC analyses, is provided in Fig. 4, while full Bayesian MCC trees by haplogroup are presented in Fig. S3 (Online Resource 3). Networks of the sequences in each haplogroup are presented in Fig. S4 (Online Resource 3), and frequency maps of some haplogroups are in Fig. S5 (Online Resource 3). A detailed discussion of each main haplogroup is in Supplemental Text (Online Resource 4); here, we summarize the main findings.

**Table 2:** The Bayesian estimates (BE) of coalescence times with 95% credible intervals (CI) for each haplogroup

Haplogroup	Sample size	BE	CI
A	17	24,401	16,499–33,138
A14	14	18,176	11,437–25,939
A17	10	14,071	7976–20,878
B4	74	34,814	30,445–46,173
B4a1c4	14	10,240	6182–14,487
B4b1a2	19	13,455	8215–19,131
B4b1a2a	17	9067	4283–11,449
B4c2	11	10,623	7107–17,631
B4 g	10	19,684	12,839–26,407
B4e	7	15,661	11,310–24,892
B5	162	36,397	24,836–46,990
B5a	160	20,252	13,196–27,886
B5a1	158	16,857	11,693–22,532
B5a1a	65	9465	7267–11,972
B5a1b1	26	8507	6438–10,686
B5a1d	52	8705	6641–11,077
B6a	26	34,428	24,086–47,839
CZ	38	37,711	26,934–48,685
C7	32	18,599	12,417–26,106
D	58	34,847	26,392–44,310
D4	52	25,375	20,235–31,447
D5	6	23,206	16,365–30,866
F1a	184	17,825	12,565–23,276
F1a1a	134	10,075	7755–11,701
F1a1a1	69	8817	7092–10,643
F1a1d	17	6676	3163–9231
F1a3	15	7305	3495–10,057
F1f	65	12,517	7000–15,389
F3a1	15	21,808	13,903–31,295
G	6	28,215	18,885–39,320
H14	4	1685	162–4576
M4	2	752	0–3414
M5	8	36,248	26,787–46,432
M7	134	50,282	39,494–62,123
M7b	106	38,342	27,442–51,252
M7b1a1	104	16,723	12,570–21,211
M7b1a1a3	27	12,659	8873–18,282
M7b1a1b	17	12,098	5973–19,336
M7b1a1 (16192T)	15	11,180	6323–17,000
M7b1a1e1	13	5936	2224–11,313
M7c	28	30,547	21,905–41,116
M7c1	21	21,657	14,519–29,420
M7c1a	12	3656	997–7882
M7c2	7	8092	4066–14,357
M8a2a1	5	12,325	5976–19,514
M9	11	26,510	18,450–35,947
M10a1b	3	1478	48–4574
M12-G	35	53,006	42,129–65,779
M12	29	37,225	29,530–46,002
M12a1	20	31,096	24,221–38,387
M12a1a	15	23,184	16,770–30,030
M12a1b	5	24,369	17,342–31,650
M12b	14	27,475	19,665–35,577
M17	7	40,440	29,244–52,628
M20	29	12,229	7521–18,355
M21b	8	29,030	20,712–38,392
M24	21	19,305	12,300–28,703
M24a	12	7550	2961–14,017
M24b	9	10,000	5175–15,821
M45	3	21,338	11,949–32,348
M49	4	23,544	14,606–33,592
M51	11	30,097	21,140–40,588
M57a	2	764	0–3524
M59	3	13,391	6372–22,559
M61	8	2987	595–6794
M68a	2	16,056	8227–25,864
M71	17	28,170	21,736–36,130
M71 (151T)	12	27,643	19,633–35,905
M72a	9	9073	4409–15,129
M73	5	3143	630–6295
M74	32	34,866	26,622–44,683
M76	7	33,689	22,405–47,078
M79	2	804	0–3499
M91	5	34,931	23,358–48,322
M*	8	49,923	38,466–63,413
N8	4	3116	683–7162
N9a	31	25,754	18,075–33,982
N9a6	7	12,056	6415–18,767
N9a10	16	17,059	11,630–22,635
N9a10 (16311C)	14	13,741	8569–19,217
N10	8	52,013	37,525–68,350
N10a	7	11,312	6144–17,061
N21	11	10,248	5291–16,123
R5a1a	3	1568	59–4465
R6a2	3	12,622	5938–20,550
R9b	35	38,677	29,454–48,807
R9b1a3	15	9849	5758–14,818
R9b2	13	11,822	6899–18,096
R22	23	39,214	29,555–50,055
U2	3	43,295	30,742–55,978
W3a1b	7	13,418	6809–22,357
Z	6	21,428	14,175–29,084

The haplogroup profiles by population emphasize the greater genetic heterogeneity in AA groups than in TK groups (Fig. 1; Table S3). Some AA groups have extremely high frequencies of particular haplogroups, indicating the pronounced effect of genetic drift; examples include: R9b2 with a frequency of 32.00% in TN2; R22 with frequencies of 17.39% in BO and 20.00% in SU; D4 with frequencies of 28.00% in MO1, 31.81% in MO5, 22.73% in LW1, and 20.00% in PL; and B6a with a frequency of 72.00% in TN1. Overall, the greater heterogeneity in haplogroup distribution and pronounced haplogroup frequency differences are consistent with an older presence of AA groups in Thailand.

Some haplogroups prevalent in South Asia also occur in some AA groups, especially the Mon groups. These include D4, mentioned above, as well as W3a1b, which is reported here for the first time in MSEA. W3a1b was found in two Mon populations (24.00% in MO1 and 4.35% in MO2); these haplogroups provide further evidence for genetic connections between these Mon groups and South Asia.

Although many haplogroups are shared between MSEA and ISEA, there are distinct differences in the distribution of some sublineages. For example, haplogroup B4 is widespread throughout SEA; in our study, it is almost entirely restricted to TK groups (Fig. 1; Table S3), where it occurs as three primary sublineages, namely B4b1a2a, B4a1c4 and B4c2, all of which have been reported previously in MSEA (Peng et al. 2010; Zhang et al. 2013). Several other B4 sublineages characteristic of Taiwan (e.g., B4b1a2h, B4b1a2f and B4b1a2g) (Ko et al. 2014), the Philippines (e.g., B4b1a2b, B4b1a2c and B4b1a2d) (Gunnarsdottir et al. 2011) and Oceania (e.g., B4a1a1a) (Duggan et al. 2014) were not found in our study, in agreement with previous studies (Summerer et al. 2014; Zhang et al. 2013). Overall, the lack of sharing of recent sublineages indicates a lack of recent contact between MSEA and ISEA (Fig. S4).

Finally, the more extensive sampling of Thai/Laos mtDNA sequences in this study has resulted in much deeper ages for some haplogroups that were poorly sampled in previous studies. For example, we estimate that haplogroups R9b and R22 both coalesce at ~39 kya (Table 2), compared to previous estimates of ~29 kya (Hill et al. 2006) and ~19 kya (Zhang et al. 2013), respectively. Moreover, while R9b and R22 have been suggested to originate in southern China (Hill et al. 2006) and ISEA (Hill et al. 2007; Zhang et al. 2013), respectively, northeastern Thailand is also a potential source for these haplogroups (Fig. S5).

### Population size change trends over time

**Fig. 5:**
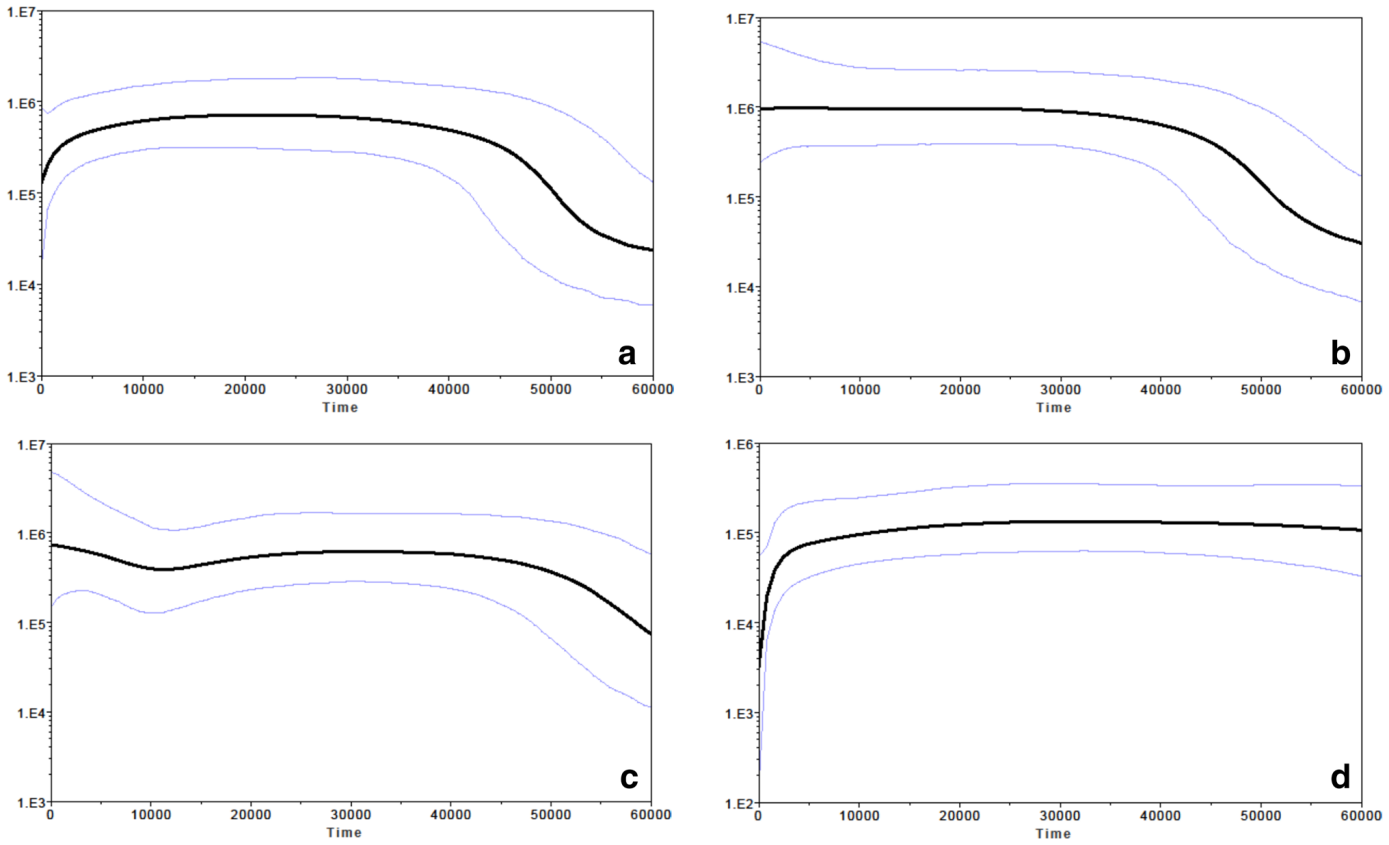
Four different trends of Bayesian skyline plots in fluctuation in maternal effective population size (*y*-axis) through time from the present in unit of years (*x*-axis) observed in the individual Bayesian skyline plots for the 51 populations (Fig. S6). The median estimate and the 95% highest posterior density limits are indicated by thick and thin lines, respectively. The plots were generated with 10,000,000 chains with the first 1,000,000 generations discarded as burn-in. Most populations (KM1–KM4, KM6–KM10, YU1–YU2, SH, IS3, PT, NY, KL, SK, BT1–BT2, PU1–PU5, MO1–MO5, KH2, BU, SO, SU, LW1, PL, BL1–BL2) show this trend in** a**; KM5, IS1–IS2 and LA2 show the trend in** b**; IS4 and LA1 show the trend in** c**; and KH1, BO, TN1–TN3, KA and LW2–LW3 show the trend in** d**

The BSP in each of the 51 populations individually (Fig. S6 in Online Resource 3) reveal four overall trends in change in *N*_e_ over time (Fig. 5). The most common trend (observed in 24 TK and 13 AA groups) is an increase in *N*_e_ around 50–40 kya, followed by stability and then a decline around 2 kya (Fig. 5a). A different trend is observed in most of the ethnic Lao populations (IS and LA) and one KM population; the IS1, IS2, LA2 and KM5 populations expanded continuously but stay stable for the present time (Fig. 5b), while IS4 and LA1 show population expansions at around 50 kya and again around 10 kya (Fig. 5c). Another pattern of observed demographic change (Fig. 5d) is a stable *N*_e_ since the upper Paleolithic, and, then, a sudden decline during the last 2 kya, which could produce a larger drift effect, and is seen in 8 AA groups.

### Testing models of demic diffusion vs. cultural diffusion vs. admixture

**Fig. 6:**
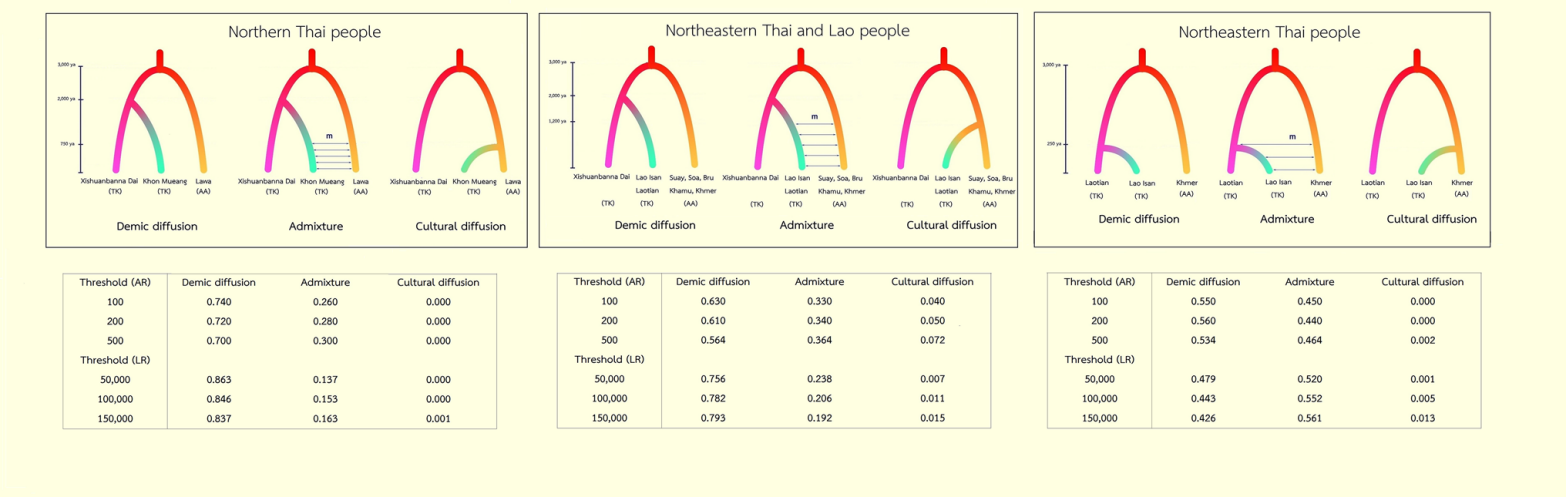
Proposed demographic models for three independent ABC tests concerning northern Thais, northeastern Thais combined with Laotian, and northeastern Thais. Each test consists of three scenarios according to three hypotheses, i.e., demic diffusion, admixture and cultural diffusion. The *tables* under each model are posterior probabilities computed by the acceptance–rejection procedure (AR) and by the weighted multinomial logistic regression (LR) approaches

To address the role of demic vs. cultural diffusion in the origins of Thai/Lao people, we proposed and tested demographic models according to immigrant vs. indigenous hypotheses (Fig. 6). The immigrant hypothesis (or demic diffusion) states that, nowadays, the TK people descend primarily from the TK-speaking groups from southern China who migrated southward in the last 1–2 kya (O’Connor 1995; Pittayaporn 2014). By contrast, the indigenous hypothesis (or cultural diffusion) suggests that the TK people descend primarily from native AA inhabitants who shifted culturally and linguistically (Condominas 1990). Also, we consider another possible scenario, namely admixture, which explains the dual origin of the current TK people as reflecting a genetic mixing of incoming TK and indigenous AA groups.

Although these three demographic scenarios are proposed for all TK people, archaeological, linguistic and historical evidence clearly indicate the potential for differences in the local history and demography, especially for groups from northern vs. northeastern Thailand (Penth 2000; Schliesinger 2001). We, therefore, performed ABC analyses using three different data sets in all three demographic scenarios: (1) northern Thai people (Khon Mueang, KM); (2) ethnic Lao including northeastern Thai people (Lao Isan, IS) and Laotian (LA); and (3) Lao Isan (to infer the history of this specific population, for reasons detailed in the “Methods” section). In each analysis, we used AA populations for comparison and set priors for some parameters (e.g., divergence and admixture time) based on historical evidence, as detailed in the “Methods” section.

In general, the results of the ABC analyses show that in all cases, the simulated data included the observed data (Fig. S7 in Online Resource 3) and the results of the model selection are consistent among different thresholds, i.e., the different numbers of simulations retained to fit the logistic regression curve. The highest posterior probabilities in both approaches, AR (0.70–0.74) and LR (0.84–0.86), support the demic diffusion model in the northern Thai KM (Fig. 6). Even though the AA-speaking LW groups have culturally interacted with the KM (Condominas 1990; Penth 2000), they are not the maternal ancestor of the KM. The test of ethnic Lao (IS and LA; scenario 2) shows the same trend in supporting the demic diffusion model, although it received higher support by LR (0.76–0.79) than by AR (0.56–0.63). The ethnic Lao are, thus, genetically distinct from the neighboring AA-speaking groups, including the KH, KA SO, SU and BU groups. These two results for TK groups across a vast area of Thailand and Laos, thus, indicate a genetic origin of the TK from southern China followed by a rapid population expansion from (presumably) a few groups to the current census size of around 50 million, within 1–2 kya. For the last analysis concerning the origin of the IS population, there is no distinction between the demic diffusion and admixture models, which differ by absence/presence of migration between KH and IS beginning ~250 years ago. The AR assigned a probability of about 0.55 to demic diffusion and about 0.45 to admixture but vice versa in LR. In either event, this analysis does not support the purely cultural diffusion model.

The results of power analysis for the three tested data sets indicated that the true positive rate is generally good, in particular for the demic diffusion model in the first two tests (which was unequivocally supported by the model selection procedures). The false positive rate is low in almost all of the comparisons (less than 0.05) for the selected model of the second test, and slightly higher (0.066) for the selected model of the first test (Table S5 in Online Resource 1). In sum, these results confirm the reliability of the posterior probabilities of the models.

## Discussion

The extensive and intensive sampling of complete mtDNA genomes in 51 AA and TK groups from Thailand and Laos shows a high genetic diversification with a total of 212 haplogroups observed. The proposed autochthonous ancient lineages are B5a1d, B6a, R22, R9b and F1f; the many basal lineages detected in this study suggests that the area of present-day Thailand and Laos may have been an ancient migratory route for modern humans, in accordance with the finding that the oldest modern human remains in East Asia are from Tam Pa Ling Cave in Laos (Demeter et al. 2012). Previous studies have suggested Myanmar (Li et al. 2015) and Cambodia (Zhang et al. 2013) as the corridor for initial settlers, assuming travel along river valleys; our results indicate that in addition, early modern human groups may have migrated through the interior upland, as also suggested by archaeological evidence found in caves in the highlands (Pureepatpong 2006; Shoocondej 2006).

Several lines of evidence point to a more ancient presence of AA groups than of TK groups, including greater genetic heterogeneity and, on average, older maternal lineages, in keeping with previous studies (Kutanan et al. 2011, 2014; Srithawong et al. 2015). There are also distinct affinities between some AA groups (especially the Mon groups) and South Asia, where AA groups are also found. TK groups are less heterogeneous, tend to show more signs of population expansion, and more genetic affinities with southern Chinese groups than with AN groups. The modeling of different demographic scenarios for different groups of populations further supports a demic diffusion of the ancestors of TK groups from southern China. However, the BSP results do not indicate population expansions in the history of some TK groups, e.g., KM. A possible explanation for this discrepancy is that sample collection procedures can produce a spurious signal of population decline in BSP analyses (Heller et al. 2013). Moreover, in addition to the ABC analyses, there is other evidence for demic diffusion of TK groups, e.g., the genetic distance analyses and the distribution of particular haplogroups.

The genetic affinities between TK and AN groups are in keeping with linguistic affinities between the TK and AN language families (Sagart 2004) and may be explained by the hypothesis that aboriginal Taiwanese (i.e., the first Austronesians) are descended from a migration associated with rice and millet farming that began in northern China between 9000 and 11,000 years ago and went both to Taiwan as well as continuing into southern China and MSEA (Ko et al. 2014). Thus, according to this view, both AN and TK groups have a common origin that reflects this agricultural expansion and can be seen in both the genetic and the linguistic data. There are further genetic affinities between MSEA and ISEA, but no sharing of recent sublineages, in keeping with previous studies that suggested a pre-Austronesian migration from MSEA to ISEA (Jinam et al. 2012).

Finally, there is widespread and significant genetic heterogeneity among samples from the same ethnolinguistic group from different locations. This result holds for all cases where there was more than one sampling location per ethnolinguistic group (Table 1). It appears that this heterogeneity arises from various sources. In the hill tribes, such as the Lawa and H’tin, isolation and drift due to geography and cultural constraints (e.g., matrilocality) appear to be the major factor. For the lowland populations (MO, KH, IS, KM, and PU), recent gene flow with other groups seems to be the major factor. Overall, these results suggest that multiple samples from the same ethnolinguistic group should be obtained whenever feasible, especially for ethnolinguistic groups distributed across a wide geographic area.

In conclusion, this study provides a comprehensive data set of complete mtDNA genome sequences, which we have utilized to gain new insights into the history of Thai/Laos populations. Information from other genetic markers, e.g., Y chromosome and genome-wide data, will provide even more insights into the genetic history of this region.

## Electronic supplementary material

Below is the link to the electronic supplementary material.

Supplementary material 1 (PDF 2260 kb)

Supplementary material 2 (PDF 1752 kb)

Supplementary material 3 (PDF 7072 kb)

Supplementary material 4 (PDF 1093 kb)
