# Supplementary material for: Complete mitochondrial genomes of Thai and Lao populations indicate an ancient origin of Austroasiatic groups and demic diffusion in the spread of Tai–Kadai languages
Source: Hum Genet. 2016 Nov 11;136(1):85–98. doi: 10.1007/s00439-016-1742-y (PMC5214972; doi:10.1007/s00439-016-1742-y)
Supplement: Supplementary file 1 — Supplementary material 1 (PDF 2260 kb) [file 439_2016_1742_MOESM1_ESM.pdf]

Online Resource 1 includes Table S1-S5 with respective legends. Table S1: Population information and genetic diversity indices. Table S2: Characteristics of the comparative populations. Table S3: Haplogroup frequencies in the studied populations. Table S4: Shared haplotypes in each pairwise comparison among the 51 Thai/Lao populations. Table S5: The results of the power analysis for the three tested datasets.

Table S1 Population information and genetic diversity indices.

Population	Code	Country		Linguistic family[a]	Linguistic branch[a]	Sample size	Haplotype information								Haplogroup information		
							Number of haplotype	Number of polymorphic site	*h*	SD	MPD	SD	*Pi*	SD	No. haplogroup	Haplogroup diversity	SD
Khon Mueang	KM1	North	Thailand	Tai-Kadai	Southwestern Tai	25	21	202	0.98	0.02	36.61	16.48	0.0022	0.00111	19	0.97	0.02
Khon Mueang	KM2	North	Thailand	Tai-Kadai	Southwestern Tai	25	20	224	0.98	0.02	34.02	15.34	0.0021	0.00104	19	0.98	0.02
Khon Mueang	KM3	North	Thailand	Tai-Kadai	Southwestern Tai	24	21	251	0.99	0.02	40.31	18.14	0.0024	0.00123	20	0.98	0.02
Khon Mueang	KM4	North	Thailand	Tai-Kadai	Southwestern Tai	25	22	234	0.99	0.02	37.62	16.93	0.0023	0.00114	17	0.96	0.02
Khon Mueang	KM5	North	Thailand	Tai-Kadai	Southwestern Tai	23	21	248	0.99	0.02	37.86	17.09	0.0023	0.00115	21	0.99	0.02
Khon Mueang	KM6	North	Thailand	Tai-Kadai	Southwestern Tai	25	18	213	0.96	0.02	39.32	17.68	0.0024	0.00119	14	0.94	0.03
Khon Mueang	KM7	North	Thailand	Tai-Kadai	Southwestern Tai	25	17	210	0.96	0.02	35	15.77	0.0021	0.00106	16	0.96	0.02
Khon Mueang	KM8	North	Thailand	Tai-Kadai	Southwestern Tai	25	18	187	0.95	0.03	38.96	17.52	0.0024	0.00118	15	0.94	0.03
Khon Mueang	KM9	North	Thailand	Tai-Kadai	Southwestern Tai	24	18	226	0.97	0.02	37.54	16.92	0.0023	0.00114	17	0.97	0.02
Khon Mueang	KM10	North	Thailand	Tai-Kadai	Southwestern Tai	25	15	158	0.95	0.02	35.83	16.14	0.0022	0.00109	11	0.87	0.05
Yuan	YU1	North	Thailand	Tai-Kadai	Southwestern Tai	17	15	174	0.99	0.03	36.79	16.85	0.0022	0.00114	12	0.94	0.04
Yuan	YU2	Central	Thailand	Tai-Kadai	Southwestern Tai	25	19	213	0.98	0.02	35.84	16.14	0.0022	0.00109	19	0.98	0.02
Shan	SH	North	Thailand	Tai-Kadai	Southwestern Tai	25	20	222	0.98	0.02	39.67	17.83	0.0024	0.0012	16	0.96	0.02
Lao Isan	IS1	Northeast	Thailand	Tai-Kadai	Southwestern Tai	25	23	250	0.99	0.01	41.17	18.5	0.0025	0.00125	20	0.97	0.02
Lao Isan	IS2	Northeast	Thailand	Tai-Kadai	Southwestern Tai	25	22	224	0.99	0.01	37.91	17.06	0.0023	0.00115	18	0.97	0.02
Lao Isan	IS3	Northeast	Thailand	Tai-Kadai	Southwestern Tai	25	17	158	0.95	0.03	35.88	16.16	0.0022	0.00109	11	0.86	0.04
Lao Isan	IS4	Northeast	Thailand	Tai-Kadai	Southwestern Tai	25	24	244	0.99	0.01	37.73	16.98	0.0023	0.00115	17	0.95	0.03
Lao	LA1	North	Laos	Tai-Kadai	Southwestern Tai	25	24	232	0.99	0.01	39.12	17.59	0.0024	0.00119	20	0.98	0.02
Lao	LA2	Central	Laos	Tai-Kadai	Southwestern Tai	24	24	258	1	0.01	36.55	16.48	0.0022	0.00111	21	0.99	0.02
Phutai	PT	Northeast	Thailand	Tai-Kadai	Southwestern Tai	25	19	193	0.97	0.02	36.15	16.28	0.0022	0.0011	14	0.94	0.03
Kalueng	KL	Northeast	Thailand	Tai-Kadai	Southwestern Tai	25	15	118	0.92	0.03	26.92	12.2	0.0016	0.00082	10	0.9	0.03

Seak	SK	Northeast	Thailand	Tai-Kadai	Northwestern Tai	26	12	147	0.83	0.06	24.21	10.99	0.0015	0.00074	10	0.67	0.1
Nyaw	NY	Northeast	Thaialnd	Tai-Kadai	Southwestern Tai	25	17	175	0.96	0.02	36.15	16.28	0.0022	0.0011	14	0.95	0.02
Black Tai	BT1	Northeast	Thailand	Tai-Kadai	Southwestern Tai	25	10	122	0.89	0.03	29.22	13.22	0.0018	0.00089	9	0.87	0.04
Black Tai	BT2	Central	Thailand	Tai-Kadai	Southwestern Tai	25	22	224	0.99	0.01	35.96	16.19	0.0022	0.00109	17	0.97	0.02
Phuan	PU1	North	Thailand	Tai-Kadai	Southwestern Tai	25	23	234	0.99	0.01	37.76	16.99	0.0023	0.00115	18	0.96	0.02
Phuan	PU2	North	Thailand	Tai-Kadai	Southwestern Tai	25	17	152	0.94	0.04	33.6	15.15	0.002	0.00102	13	0.9	0.04
Phuan	PU3	North	Thailand	Tai-Kadai	Southwestern Tai	25	19	202	0.97	0.02	35.71	16.09	0.0022	0.00109	16	0.95	0.03
Phuan	PU4	Central	Thailand	Tai-Kadai	Southwestern Tai	25	19	187	0.97	0.02	33.35	15.04	0.002	0.00102	15	0.96	0.02
Phuan	PU5	Central	Thailand	Tai-Kadai	Southwestern Tai	25	14	158	0.94	0.03	34.59	15.59	0.0021	0.00105	13	0.93	0.03
Mon	MO1	Northeast	Thailand	Austroasiatic	Monic	25	13	190	0.89	0.04	37.84	17.03	0.0023	0.00115	11	0.87	0.05
Mon	MO2	West	Thailand (Thailand Myanmar border)	Austroasiatic	Monic	23	18	250	0.97	0.03	42.6	19.19	0.0026	0.0013	17	0.96	0.03
Mon	MO3	East	Myanmar (Thailand Myanmar border)	Austroasiatic	Monic	15	13	180	0.98	0.03	38.9	17.93	0.0024	0.00122	12	0.96	0.04
Mon	MO4	Central	Thailand	Austroasiatic	Monic	25	18	220	0.96	0.02	34.22	15.43	0.0021	0.00104	18	0.96	0.02
Mon	MO5	Central	Thailand	Austroasiatic	Monic	22	14	191	0.95	0.03	30.86	14.01	0.0019	0.00095	14	0.95	0.03
Khmer	KH1	Northeast	Thailand	Austroasiatic	Eastern Mon-Khmer	19	12	127	0.90	0.06	32.77	14.96	0.002	0.00101	8	0.83	0.07
Khmer	KH2	Northeast	Thailand	Austroasiatic	Eastern Mon-Khmer	25	16	178	0.90	0.05	32.75	14.78	0.002	0.001	12	0.88	0.05
Nyahkur	BO	Northeast	Thailand	Austroasiatic	Southern Monic	23	8	114	0.85	0.04	39.62	17.87	0.0024	0.00121	5	0.79	0.04
Suay	SU	Northeast	Thailand	Austroasiatic	Eastern Mon-Khmer	25	16	197	0.95	0.02	38.34	17.24	0.0023	0.00116	12	0.92	0.03
Soa	SO	Northeast	Thailand	Austroasiatic	Eastern Mon-Khmer	25	20	211	0.98	0.02	34.89	15.72	0.0021	0.00106	15	0.94	0.03
Bru	BU	Northeast	Thailand	Austroasiatic	Eastern Mon-Khmer	24	13	136	0.93	0.03	31.52	14.26	0.0019	0.00096	10	0.88	0.04
H’tin	TN1	North	Thailand	Austroasiatic	Southern Mon-Khmer	25	8	88	0.85	0.04	21.41	9.77	0.0013	0.00066	5	0.51	0.1
H’tin	TN2	North	Thailand	Austroasiatic	Southern Mon-Khmer	25	7	78	0.80	0.05	26.14	11.86	0.0016	0.0008	4	0.74	0.04
H’tin	TN3	North	Thailand	Austroasiatic	Southern Mon-Khmer	25	13	146	0.88	0.05	36.07	16.25	0.0022	0.0011	9	0.8	0.06
Khamu	KA	North	Thailand	Austroasiatic	Southern Mon-Khmer	25	15	132	0.93	0.03	30.19	13.65	0.0018	0.00092	11	0.81	0.07
Blang	BL1	North	Thailand	Austroasiatic	Southern Mon-Khmer	25	19	186	0.98	0.02	34.85	15.71	0.0021	0.00106	14	0.94	0.03
Blang	BL2	North	Thailand	Austroasiatic	Southern Mon-Khmer	25	21	162	0.98	0.02	34.43	15.52	0.0021	0.00105	10	0.89	0.04

Paluang	PL	North	Thailand	Austroasiatic	Southern Mon-Khmer	25	17	213	0.97	0.02	37.31	16.79	0.0023	0.00113	15	0.95	0.02
Lawa	LW1	North	Thailand	Austroasiatic	Southern Mon-Khmer	22	11	152	0.93	0.03	35.36	16.01	0.0021	0.00108	10	0.91	0.03
Lawa	LW2	North	Thailand	Austroasiatic	Southern Mon-Khmer	24	16	169	0.93	0.04	38.29	17.25	0.0023	0.00116	10	0.88	0.04
Lawa	LW3	North	Thailand	Austroasiatic	Southern Mon-Khmer	24	11	142	0.87	0.05	34.58	15.61	0.0021	0.00105	8	0.84	0.04

[a] Linguistic classification is based on Ethnologue[1]

Reference

1. Lewis, M.P., Simons, G.F., Fennig, C.D. (2016). Ethnologue: Languages of the World, Nineteenth edition (SIL International). Available from http://www.ethnologue.com)

Table S2 Characteristics of the comparative populations.

	Population	Code	Sample size	Language	Country/Region	Reference
1	Buryat	BUR	91	Altai	Buryat Republic, North Asia	Derenko et al. (2007, 2010, 2012)
2	Evenki	EV	17	Altai	North Asia	Derenko et al. (2007, 2010, 2012); Sukernik et al. (2012)
3	Altaian-Kizhi Southern Altai	AT_S	18	Altai	Altai Republic, South Siberia, Russia	Derenko et al. (2007, 2010, 2012)
4	Khamnigan	KN	32	Altai	Buryat Republic, South Siberia, Russia	Derenko et al. (2007, 2010, 2012)
5	Bargat	BAR	55	Altai	Inner Mongolia, China	Derenko et al. (2007, 2010, 2012)
6	Khmer	KH_C	18	Austroasiatic	Cambodia	Zhang et al. (2013)
7	Austroasiatic Cambodian	AA_C	65	Austroasiatic	Cambodia	Zhang et al. (2013)
8	Malpaharia	ML	15	Austroasiatic	East India	Chandrasekar et al. (2009)
9	Munda	MUN	31	Austroasiatic	East India	Chandrasekar et al. (2009)
10	Korku	KK	15	Austroasiatic	Central India	Chandrasekar et al. (2009)
11	Cham	CH	16	Austronesian	Vietnam	Peng et al. (2010)
12	Temuan	TM	18	Austronesian	West Malaysia	Jinam et al. (2012)
13	Seletar	SE	21	Austronesian	West Malaysia	Jinam et al. (2012)
14	Jehai	JH	24	Austronesian	West Malaysia	Jinam et al. (2012)
15	Bidayuh	BD	23	Austronesian	Borneu, Indonesia	Jinam et al. (2012)
16	Besemah	BS	36	Austronesian	Sumatra, Indonesia	Gunnarsdóttir et al. (2011b)
17	Semende	SMD	35	Austronesian	Sumatra, Indonesia	Gunnarsdóttir et al. (2011b)
18	Mamanwa	MM	32	Austronesian	northern Mindanao, Philippines	Gunnarsdottir et al. (2011a)
19	Manobo	MN	40	Austronesian	northern Mindanao, Philippines	Gunnarsdottir et al. (2011a)

20	Surigaonon	SR	25	Austronesian	northern Mindanao, Philippines	Gunnarsdottir et al. (2011a)
21	Abaknon	AB	30	Austronesian	Philippines	Delfin et al. (2014)
22	Aeta_Bataan	AEB	21	Austronesian	Philippines	Delfin et al. (2014)
23	Bagalot	BAG	30	Austronesian	Philippines	Delfin et al. (2014)
24	Ibaloi	IB	26	Austronesian	Philippines	Delfin et al. (2014)
25	Ifugao	IF	29	Austronesian	Philippines	Delfin et al. (2014)
26	Ivatan	IV	29	Austronesian	Philippines	Delfin et al. (2014)
27	Kalangoya	KAG	26	Austronesian	Philippines	Delfin et al. (2014)
28	Kankanaey	KAN	30	Austronesian	Philippines	Delfin et al. (2014)
29	Maranao	MR	18	Austronesian	Philippines	Delfin et al. (2014)
30	Saisiat	SAI	24	Austronesian	Taiwan	Ko et al. (2014)
31	Atayal	ATA	50	Austronesian	Taiwan	Ko et al. (2014)
32	Tsou	TSO	48	Austronesian	Taiwan	Ko et al. (2014)
33	Bunun	BUN	51	Austronesian	Taiwan	Ko et al. (2014)
34	Puyuma	PUY	39	Austronesian	Taiwan	Ko et al. (2014)
35	Rukai	RUK	50	Austronesian	Taiwan	Ko et al. (2014)
36	Paiwan	PAI	50	Austronesian	Taiwan	Ko et al. (2014)
37	Ami	AMI	50	Austronesian	Taiwan	Ko et al. (2014)
38	Tao	TAO	44	Austronesian	Taiwan	Ko et al. (2014)
39	Makatao	MAK	50	Austronesian	Taiwan	Ko et al. (2014)
40	Andh	AD	19	Indo-European	Central India	Chandrasekar et al. (2009)
41	Dongri Bhill	DB	43	Indo-European	West India	Chandrasekar et al. (2009)
42	Kamar	KMR	54	Indo-European	Central India	Chandrasekar et al. (2009)
43	Kathakur	KU	18	Indo-European	West India	Chandrasekar et al. (2009)
44	Kathodi	KD	15	Indo-European	West India	Chandrasekar et al. (2009)
45	Katkari	KR	21	Indo-European	West India	Chandrasekar et al. (2009)
46	Nihal	NI	28	Indo-European	Central India	Chandrasekar et al. (2009)
47	Sonowal Kachari	SOK	19	Indo-European	Northeast India	Chandrasekar et al. (2009)
48	Barmar1	BR1	20	Sino-Tibetan	Myanmar	Summerer et al. (2014)
49	Barmar2	BR2	73	Sino-Tibetan	Myanmar	Li et al. (2015)

50	Southern Han Chinese	HN_S	55	Sino-Tibetan	Hunan and Fujian Provinces, Southern China	Zheng et al. (2011)
51	Northern Han Chinese	HN_N	89	Sino-Tibetan	Beijing, Northern China	Zheng et al. (2011)
52	Tibetan	TB	23	Sino-Tibetan	Tibet and Southern China	Zhao et al. (2009)
53	Hakka	HAK	45	Sino-Tibetan	Taiwan	Ko et al. (2014)
54	Minnan	MIN	50	Sino-Tibetan	Taiwan	Ko et al. (2014)
55	Dirang Monpa	DR	30	Sino-Tibetan	Northeast India	Chandrasekar et al. (2009)
56	Gallong	GL	39	Sino-Tibetan	Northeast India	Chandrasekar et al. (2009)
57	Lachungpa	LAH	25	Sino-Tibetan	Northeast India	Chandrasekar et al. (2009)
58	Lepcha	LP	20	Sino-Tibetan	Northeast India	Chandrasekar et al. (2009)
59	Shertukpen	ST	15	Sino-Tibetan	Northeast India	Chandrasekar et al. (2009)
60	Toto	TO	28	Sino-Tibetan	Northeast India	Chandrasekar et al. (2009)
61	Wanchoo	WA	22	Sino-Tibetan	Northeast India	Chandrasekar et al. (2009)
62	Dai	DA	56	Tai-Kadai	Yunnan, Southern China	Diroma et al. (2014)

References

Chandrasekar A, Kumar S, Sreenath J, Sarkar BN, Urade BP, Mallick S, Bandopadhyay SS, Pinuma Barua P, Barik SS, Basu D, Kiran U, Gangopadhyay P, Sahani R, Prasad BVR, Gangopadhyay S, Lakshmi GR, Ravuri RR, Padmaja K, Venugopal PN, Sharma MB, Rao VR (2009) Updating phylogeny of mitochondrial DNA macrohaplogroup M in India: dispersal of modern human in south Asian corridor. PLoS One 4:e7447

Delfin FS, Min-Shan KA, Li M, Gunnarsdóttir ED, Tabbada KA, Salvador JM, Calacal GC, Sagum MS, Datar FA, Padilla SG, De Ungria MC, Stoneking M (2014) Complete mtDNA genomes of Filipino ethnolinguistic groups: A melting pot of recent and ancient lineages in the Asia-Pacific region. Eur J Hum Genet 22:228-237

Derenko M, Malyarchuk B, Grzybowski T, Denisova G, Dambueva I, Perkova M, Dorzhu C, Luzina F, Lee HK, Vanecek T, Villems R, Zakharov I (2007) Phylogeographic analysis of mitochondrial DNA in Northern Asian populations. Am J Hum Genet 81:1025-1041

Derenko M, Malyarchuk B, Grzybowski T, Denisova G, Rogalla U, Perkova M, Dambueva I, Zakharov I (2010) Origin and post-glacial dispersal of mitochondrial DNA haplogroups C and D in Northern Asia. PLoS One 5:e15214

Derenko M, Malyarchuk B, Denisova G, Perkova M, Rogalla U, Grzybowski T, Khusnutdinova E, Dambueva I, Zakharov I (2012) Complete mitochondrial DNA analysis of Eastern Eurasian haplogroups rarely found in populations of Northern Asia and Eastern Europe. PLoS One 7:e32179analysis of Eastern Eurasian haplogroups rarely found in populations of Northern Asia and Eastern Europe. PLoS One *7*, e32179.

Diroma MA, Calabrese C, Simone D, Santorsola M, Calabrese, F.M., Gasparre G and Attimonelli M (2014) Extraction and annotation of human mitochondrial genomes from 1000 Genomes Whole Exome Sequencing data. BMC Genomics 15:S2

Gunnarsdóttir ED, Li M, Bauchet M, Finstermeier K, Stoneking M (2011a) High throughput sequencing of complete human mtDNA genomes from the Philippines. Genome Res 21:1-11

Gunnarsdóttir ED, Nandineni MR, Li M, Myles S, Gil D, Pakendorf B, Stoneking M (2011b) Larger mitochondrial DNA than Y-chromosome differences between matrilocal and patrilocal groups from Sumatra. Nat Commun 2:228

Jinam TA, Hong LC, Phipps ME, Stoneking M, Ameen M, Edo J, HUGO Pan-Asian SNP Consortium, Saitou N (2012) Evolutionary history of continental Southeast Asians: “early train” hypothesis based on genetic analysis of mitochondrial and autosomal DNA data. Mol Biol Evol 29:3513-3527

Ko AM, Chen CY, Fu Q, Delfin F, Li M, Chiu HL, Stoneking M, Ko YC (2014) Early Austronesians: Into and out of Taiwan. Am J Hum Genet 94:426-436

Li YC, Wang HW, Tian JY, Liu LN, Yang LQ, Zhu CL, Wu SF, Kong QP, Zhang YP (2015) Ancient inland human dispersals from Myanmar into interior East Asia since the Late Pleistocene. Sci Reports 5:9473

Peng MS, Quang HH, Dang KP, Trieu AV, Wang HW, Yao YG, Kong QP, Zhang YP (2010) Tracing the Austronesian footprint in mainland Southeast Asia: a perspective from mitochondrial DNA. Mol Biol Evol 27:2417-2430

Sukernik RI, Volodko NV, Mazunin IO, Eltsov NP, Dryomov SV, Starikovskaya EB (2012). Mitochondrial genome diversity in the Tubalar, Even, and Ulchi: contribution to prehistory of native Siberians and their affinities to native Americans. Am. J. Phys. Anthropol. 148:123-138.

Summerer M, Horst J, Erhart J, Weißensteiner H, Schönherr S, Pacher D, Forer L, Horst D, Manhart A, Horst B, Sanguansermsri T, Brandstätter AK (2014) Large-scale mitochondrial DNA analysis in Southeast Asia reveals evolutionary effects of cultural isolation in the multi-ethnic population of Myanmar. BMC Evol 14:17

Zhao M, Kong QP, Wang HW, Peng MS, Xie XD, Wang WZ, Jiayang, Duan JG, Cai MC, Zhao, SN, Cidanpingcuo, Tu YQ, Wua SF, Yao YG, Bandelt HJ, Zhang YP (2009) Mitochondrial genome evidence reveals successful late Paleolithic settlement on the Tibetan Plateau. Proc. Natl. Acad. Sci. USA 106:21230-21235.

Zhang X, Qi X, Yang Z, Serey B, Sovannary T, Bunnath L, Aun HS, Samnom H, Zhang H, Lin Q, Oven MV, Shi H, Su B (2013) Analysis of mitochondrial genome diversity identifies new and ancient maternal lineages in Cambodian aborigines. Nat Commun 4:2599

Zheng HX, Yan S, Qin ZD, Wang Y, Tan JZ, Li H, Jin L (2011) Major population expansion of East Asians began before Neolithic time: evidence of mtDNA genomes. PLoS One 6:e25835

Table S3 Haplogroup frequencies in the studied populations.

	Tai-Kadai (TK)																													
	KM1	KM2	KM3	KM4	KM5	KM6	KM7	KM8	KM9	KM10	YU1	YU2	SH	IS1	IS2	IS3	IS4	LA1	LA2	PT	KL	SK	NY	BT1	BT2	PU1	PU2	PU3	PU4	PU5
A (152C)									4																					
A13	4									4																				
A14																														
A17																														
B4 (16261)															4															
B4a1c4		8					4						4	4	4	4	4			8			8							4
B4a1e		4			4				4																					
B4b1																												4		
B4b1a2										8																				
B4b1a2a				12	4	16	4		4								4			16				8						
B4c1b					4	4																								
B4c1b2a										8																				
B4c1b2c		4																	4											
B4c2																	4	4				4				4	16		8	
B4e											6								8							4		4	8	
B4g															4					8					8					
B4g1a	4		4							4										4										
B4h																										4				
B4h1																									4					
B5a																									8					
B5a1												8			4								4		4	4				
B5a1a	4	8			4		12				6		4		4	2	2	4	8		8	8	12	24	4	4		4	4	
B5a1b1		4	8								12							8	4			4	4		8	16			4	
B5a1c																					2									
B5a1c1																		4												
B5a1d				12	4				4			4					8	4		4	12	58	4							
B5b1																														
B5b1a													4																	
B6a						4																			4	4		8		
B6a1									8																					
B6a1a							4						4																	
C4a3b						4																								
C4a5																														
C4d				4																										
C7	8	8	4		4									4																
C7a								4		4					8						4			4						4
C7a1	4		4																											
C7a1a																														
C7a2	4	12												4				8		4										
D4				8						8																				
D4a					4	8												8										4		
D4b2b																														
D4e1a																														
D4e3																						4		8						
D4g2a1																														
D4g2a1b							4																		8					

	Austroasiatic (AA)																				
	MO1	MO2	MO3	MO4	MO5	KH1	KH2	BO	SU	SO	BU	TN1	TN2	TN3	KA	BL1	BL2	PL	LW1	LW2	LW3
A (152C)																					
A13																					
A14				4																	13
A17					5												8			4	25
B4 (16261)																					
B4a1c4														4							
B4a1e																					
B4b1																					
B4b1a2																					
B4b1a2a																					
B4c1b																					
B4c1b2a																					
B4c1b2c																					
B4c2										4											
B4e																					
B4g														4							
B4g1a																					
B4h																					
B4h1																					
B5a																					
B5a1										4							8				
B5a1a				4		21	16		4	12					4	8	8	4	5	21	
B5a1b1										8	29										
B5a1c																					
B5a1c1																					
B5a1d						5				2	17			4	4	8					
B5b1									4												
B5b1a																					
B6a				4	5							68						8			
B6a1												4					4				
B6a1a																			14		
C4a3b																					
C4a5																		8			
C4d																					
C7						5															
C7a																					
C7a1		17	7																		
C7a1a				4																	
C7a2	4																				
D4				4												4			14		
D4a																					
D4b2b															4						
D4e1a	28																				
D4e3																					
D4g2a1				4																	
D4g2a1b																					

D4g2a1c																																	7		5																
D4h									4																																	4			4						
D4h3												4																																							
D4j1																																																16			
D4j1a1											6																								18														9	4	
D4j1b		4																																	5													4			
D4j3																																			5																
D5a2a1																																																		4	
D5b												8							4							4																									
F1 (16189C)														4																																					
F1a1	4			4															4																										4						
F1a1'4	16																																																		
F1a1a	4				4	12	8	4	13			4		4	8	4	4	8			16	4						4		4		9	7	8		11	4	3		4			36	36	2						
F1a1a1	8		4		4		4	4	13			4	12	16	4		8	4	4	16	8			4					4		4	4	2	4		37	8		12		4	4	2	8	4	12	12	8		8	4
F1a1d							8	4			6		4	4	4									2			8		12																						
F1a2							4					8																											4												
F1a2a																				4			8											4																	
F1a3											6	8												2	8					12																		8			
F1a4a1			4	4																																															
F1a'c'f																					2																														
F1c1a										4																																			4			8			
F1d																																4																			
F1d1																																4																			
F1e3																			4								4		4																						
F1f	4	4		4			16		4	16	12	4	8					4	8				16				4	16	12			4		12			8									2	28		23	13	29
F1g																										8	4		8																						
F2																			4						8																										
F2b1									4					4	4																																				
F2d				4	4																																														
F2e				4							6																	4	4																						
F3a1	4		8			4	4	8	8				8																	16																					
F4a2								4										4	4										8									4													
F4b			4											4																																					
G2a1d2a												4																																				4			
G2b1a																							4																												
G2b1a1		4									6																																								
G3b2																																																	5		
H14																																		16																	
I1e												4																																							
K1a4																	4																																		
M					4				4			4																				9			9		4														
M10a1a								12																																											
M1'20'51																																	7																		
M12a1a				4														4							8							4											12	28							
M12a1b	4																															4															8	4			
M12b																																													4						
M12b1a																			4																																
M12b1a2																																								4											
M12b1b														4										4													4		12												
M13c																																	7																		
M17a																		8																												4			5		
M17c			4																																																

M17c1a																														8																					
M19																																						4												
M20		4		4	8												4	4			12			12					4									12	8					4	4		9	25	4
M21b											4																						4					12								12				
M22a													4																																					
M23'75																								4																									
M24a				4								4			8																						32														
M24b				8																4		4						4								8		4		4										
M2c		4																																																	
M32c																									4																								
M33a3a																															4																		
M33c																		4																															
M37e																				4																													
M3a1 (204C)															4																																		
M4																														8																			
M45																																		5															
M45a																															9																		
M49																																		9															
M49e																																	4																
M49e1																																7																	
M51a															4						4																												
M51a1b																																				4	26													
M51b1a																						8																											
M57a																																		9															
M59													8														4																						
M5a																																	4	9															
M5a2a										6																																							
M5b				4																																														
M5c1																																	8																
M5c2																														4																			
M60																														4																			
M61																					4				4	8			16																					
M68a																											4												4										
M6a1a																														4																			
M71 (151T)							4					8				4		4									4																		8				13	4
M71a		4									4																																						
M71a1a																													4																				
M71a2																																													4				
M71b				4																																														
M72a																		4	4																		16	8	4									
M73					4																								12				5															
M74														4		4																																	
M74a		4					4		4																						4																		
M74b												12														4						7												4				14		
M74b1																4			12												4												4	8	4					
M74b2																																7					22	4							4					
M75													4																																				
M76							2																																										
M76a					4																																							4						
M79																																								8									
M7b1a1	4																									4		16									4					2									

M7b1a1 (16192T)			4					8				4		4	16	4		4					4			4		8																							
M7b1a1a																											4		4																			4			
M7b1a1a2		4			4	4																																													
M7b1a1a3	4		4				8	4		32			4		4		8	4	4	8						8									9	11															
M7b1a1b	4		4		9	16		8	8									4							4		8													4											
M7b1a1d 1												4																																							
M7b1a1e																	4																							4											
M7b1a1e1				4										4		28											4			8						5															
M7b1a1f					4						6		8	8							4									4											12										8
M7b2a																								8																											
M7c1a		8																4									28	4	4																						
M7c1b2b													4																					4	5																
M7c1c2		4	4					4	4					4																									4												
M7c2																						8																													
M7c2a													8		4		8																																		
M8a2a1				4				8														4																										4			
M91a					9				4																								7																		
M91b																									4																										
M9a1a1																																														4	8				
M9a1b1		4																																																	
M9a4a																		4																																	
M9a4a1																												8																							
M9a5		4																					4							4																					
M9b																										4																									
N10a							4										4									4														4											13
N10b							4																																												
N21 (195C)																				4					4																13					12					
N21a												12																																							
N22		4			4																																														
N8											24																																								
N9a			13																																																
N9a1						4										4																																			
N9a10					4																											4																			
N9a10 (16311C)			4			8	8		4	4			4			4		4							8	4	4																								
N9a2'4'5																		4																																	
N9a3																																	7	4																	
N9a6					4															4									12											4										4	
R																																	13																		
R11b1																																					4														
R2																																																	5		
R22															8	2	4		4				8													5	4	17	2									4			
R30																															4																				
R5a1a																																																12			
R6a2		4													4																			4																	
R9b														4			4																																		
R9b1																			4																																
R9b1a1a														4																																					
R9b1a2	4															4																																			
R9b1a3			4	12	4			4		8		4							4								4	4		12																					
R9b1b						4																																													
R9b2														4					4																					4			32	8							

**R9c1a1**	**4**																																																		
**R9c1b1**			**4**																						**4**																										
**R9c1b2**	**8**																																																		
**U2b2**																																**4**																			
**U2c1**													**4**																																						
**U2c1b**																																**4**																			
**W3a1b**																															**24**	**4**																			
**Z**																																														**4**					
**Z3**															**4**																																				
**Z3a**																																																**4**		**4**	
**Z3a1a**			**4**																																																
**Z4**																														**4**																					

Table S4 Shared haplotypes in each pairwise comparison among the 51 Thai/Lao populations. Population abbreviations are given in Fig. 1.

	KM1	KM2	KM3	KM4	KM5	KM6	KM7	KM8	KM9	KM10	YU1	YU2	SH	IS1	IS2	IS3	IS4	LA1	LA2	PT	KL	SK	NY	BT1	BT2	PU1	PU2	PU3	PU4	PU5	MO1	MO2	MO3	MO4	MO5	KH1	KH2	BO	SU	SO	BU	TN1	TN2	TN3	KA	BL1	BL2	PL	LW1	LW2	LW3
KM1																																																			
KM2	0																																																		
KM3	1	0																																																	
KM4	1	0	0																																																
KM5	1	0	0	2																																															
KM6	0	0	1	1	5																																														
KM7	0	0	1	1	1	2																																													
KM8	0	0	0	1	0	0	3																																												
KM9	1	2	0	0	0	0	0	0																																											
KM10	1	0	1	1	0	1	1	0	0																																										
YU1	0	0	0	0	0	0	0	0	0	0																																									
YU2	0	0	0	0	1	0	0	0	0	0	0																																								
SH	1	0	0	0	0	0	1	0	1	0	0	0																																							
IS1	0	0	1	0	0	0	0	0	0	0	0	1	0																																						
IS2	0	0	0	0	0	0	0	1	0	0	0	0	0	0																																					
IS3	0	0	0	0	0	0	0	0	0	0	0	0	0	0	0																																				
IS4	0	0	0	0	0	0	0	0	0	0	0	0	0	0	1	0																																			
LA1	0	0	0	0	0	0	0	1	0	0	0	0	0	0	0	0	1																																		
LA2	0	0	0	0	0	0	0	0	0	0	0	0	0	0	0	0	1	1																																	
PT	0	0	0	0	0	0	1	0	0	0	0	0	0	0	0	0	1	0	0																																
KL	0	0	0	0	0	0	0	0	0	0	0	0	0	0	0	0	0	0	0	0																															
SK	0	0	0	0	0	0	0	0	0	0	0	0	0	0	0	0	0	0	0	0	0																														
NY	0	0	0	0	0	0	1	0	0	0	0	0	0	0	0	0	0	0	0	1	0	0																													
BT1	0	0	0	0	0	0	0	0	0	0	0	0	0	0	0	0	0	0	0	0	0	0	0																												
BT2	0	0	0	0	0	0	0	0	0	0	0	0	0	0	0	0	0	0	0	0	0	0	0	0																											
PU1	0	0	2	0	0	0	0	0	0	0	0	0	0	0	0	0	0	0	1	0	0	0	1	0	0																										
PU2	0	0	0	0	0	0	0	0	1	0	0	0	1	0	0	0	0	0	1	0	0	0	0	0	0	2																									
PU3	0	0	0	0	0	0	0	0	0	0	1	0	0	0	0	0	1	1	1	0	0	0	0	0	0	3	1																								
PU4	0	0	0	0	0	0	0	0	0	0	1	0	0	0	0	0	0	0	2	0	1	0	1	0	0	3	5	2																							
PU5	1	0	0	0	0	0	1	0	1	0	0	0	1	0	0	0	0	0	0	1	0	0	1	0	1	1	2	0	0																						
MO1	0	0	0	0	0	0	0	0	0	0	0	0	0	0	0	0	0	0	0	0	0	0	0	0	0	0	0	0	0	0																					
MO2	0	0	0	0	0	0	0	0	0	0	0	0	0	0	0	0	0	0	0	0	0	0	0	0	0	0	0	0	0	0	0																				
MO3	0	0	0	0	0	0	0	0	0	0	0	0	0	0	0	0	0	0	0	0	0	0	0	0	0	0	0	0	0	0	0	1																			
MO4	0	0	0	0	0	0	0	0	0	0	0	0	0	0	0	0	0	0	0	0	0	0	0	0	0	0	0	0	0	0	0	0	0																		
MO5	0	0	0	0	0	1	0	0	0	0	0	0	0	0	0	0	0	0	0	0	0	0	0	0	0	0	0	0	0	0	0	0	0	0																	
KH1	0	0	0	0	0	0	0	0	0	0	0	0	0	0	0	0	1	1	2	0	0	0	0	0	0	0	0	0	0	0	0	0	0	0	0																
KH2	0	0	0	0	0	0	0	0	0	0	0	0	0	0	0	0	0	0	1	0	0	0	1	1	0	0	0	0	1	0	0	0	0	0	0	2															
BO	0	0	0	0	0	0	0	0	0	0	0	0	0	0	0	0	0	0	0	0	0	0	0	0	0	0	0	0	0	0	0	0	0	0	0	0	1														
SU	0	0	0	0	0	0	0	0	0	0	0	0	0	0	0	0	0	0	1	0	0	0	1	0	0	0	0	0	1	0	0	0	0	0	0	2	2	0													
SO	0	0	0	0	0	0	0	0	0	0	0	0	0	0	0	0	0	0	0	1	0	0	1	0	0	0	0	0	0	0	0	0	0	0	0	0	0	0	0												
BU	0	0	0	0	0	0	0	0	0	0	0	0	0	0	0	0	0	0	0	1	1	0	1	0	0	0	0	0	1	0	0	0	0	0	0	0	1	0	1	2											
TN1	0	0	0	0	0	0	0	0	0	0	0	0	0	0	0	0	0	0	0	0	0	0	0	0	0	0	0	0	0	0	0	0	0	0	0	0	0	0	0	0	0										
TN2	0	0	0	0	0	0	0	0	0	0	0	0	0	0	0	0	0	0	0	0	0	0	0	0	0	0	0	0	0	0	0	0	0	0	0	0	0	0	0	0	0	0									
TN3	0	0	0	0	0	0	0	0	0	0	0	0	0	0	0	0	0	0	0	0	0	0	0	0	0	0	0	0	0	0	0	0	0	0	0	0	0	0	0	0	0	0	4								
KA	0	0	0	0	0	0	0	0	1	0	0	0	0	0	0	0	0	1	0	0	0	0	0	0	0	0	0	0	0	0	0	0	0	0	0	1	0	0	0	0	0	1	0	0							
BL1	0	0	0	0	0	0	0	0	0	0	0	0	0	0	0	0	0	0	0	0	0	0	0	0	0	0	0	0	0	0	0	0	0	0	0	0	0	0	0	0	0	0	0	0	0						
BL2	0	0	0	0	0	0	0	0	0	0	0	0	0	0	0	0	0	0	0	0	0	0	0	0	0	0	0	0	0	0	0	0	0	0	0	0	0	0	0	0	0	0	0	0	0	1					
PL	0	0	0	0	0	0	0	0	0	0	0	0	0	0	0	0	0	0	0	0	0	0	0	0	0	0	0	0	0	0	0	1	0	0	1	0	0	0	0	0	0	0	0	0	0	0	0				
LW1	0	0	0	1	0	0	0	0	0	0	0	0	0	0	0	0	0	0	0	0	0	0	0	0	0	0	0	0	0	0	0	0	0	0	0	0	0	0	0	0	0	0	0	0	1	0	0	0			
LW2	0	0	0	0	0	0	0	0	0	0	0	0	0	0	0	0	2	0	1	0	0	0	0	0	0	0	0	0	0	0	0	0	0	0	0	1	0	0	0	0	0	0	0	0	0	0	0	0	0		
LW3	0	0	0	0	0	0	0	0	0	0	0	0	1	0	0	0	0	0	0	0	0	0	0	0	0	0	0	0	0	0	0	0	0	0	0	0	0	0	0	0	0	0	0	0	0	0	0	0	1	0	

Table S5 The results of the power analysis for the three tested datasets.

	True Positive	False Positive
Test1 Northern Thai people		
model1	0.574	0.066
model2	0.222	0.227
model3	0.734	0.0355
Test2 Northeastern Thai and Lao people		
model1	0.61	0.0485
model2	0.235	0.211
model3	0.589	0.0625
Test3 Northeastern Thai people		
model1	0.541	0.137
model2	0.373	0.207
model3	0.86	0.014
