# Supplementary material for: Complete mitochondrial genomes of Thai and Lao populations indicate an ancient origin of Austroasiatic groups and demic diffusion in the spread of Tai–Kadai languages
Source: Hum Genet. 2016 Nov 11;136(1):85–98. doi: 10.1007/s00439-016-1742-y (PMC5214972; doi:10.1007/s00439-016-1742-y)
Supplement: Supplementary file 2 — Supplementary material 2 (PDF 1752 kb) [file 439_2016_1742_MOESM2_ESM.pdf]

Online Resource 2 is sequence coverage for the studied mtDNA genomes.

Average coverage is indicated by black solid line while minimum and maximum coverages are represented by gray lines. The sample identification of each population is as follows:

Sample ID	Population Code	Sample ID	Population Code
A1YU101-A1YU137	KM1	PAA101-PCC144	PU2
A2YU101-A2YU152	KM2	PPA101-PPA144	PU3
A2YU202-A2YU238	KM3	LTP101-LTP147	PU4
A2YU301-A2YU357	KM4	SPP101-SPP135	PU5
A2YU401-A2YU439	KM5	MO101-MO147	MO1
A2YU501-A2YU548	KM6	MON201-MON251	MO2
A2YU601-A2YU652	KM7	MON301-MON329	MO3
A3YU103-A3YU147	KM8	MLB101-MLB141	MO4
A4YU101-A4YU143	KM9	RM101-RM129	MO5
A4YU201-A4YU227	KM10	KHM101-KHM125	KH1
UT401-UT427	YU1	KHM201-KHM239	KH2
RY101-RY145	YU2	BO101-BO147	BO
YA101-YA139	SH	SU101-SU148	SU
LAO101-LAO148	IS1	SO101-SO145	SO
LAO401-LAO449	IS2	BRU101-BRU137	BU
LAO501-LAO550	IS3	TN101-TN153	TN1
LAO601-LAO632	IS4	TN201-TN236	TN2
LUA101-LUA149	LA1	TN301-TN367	TN3
VIE101-VIE149	LA2	KM101-KM142	KA
PUT101-PUT144	PT	PP101-PP181	BL1
KAL101-KAL149	KL	PP203-PP280	BL2
SAK101-SAK140	SK	PL101-PL203	PL
YOH101-YOH141	NY	LW101-LW149	LW1
BAT101-BAT137	BT1	LW501-LW549	LW2
PT101-PT141	BT2	LW601-LW641	LW3
STP101-STP141	PU1		

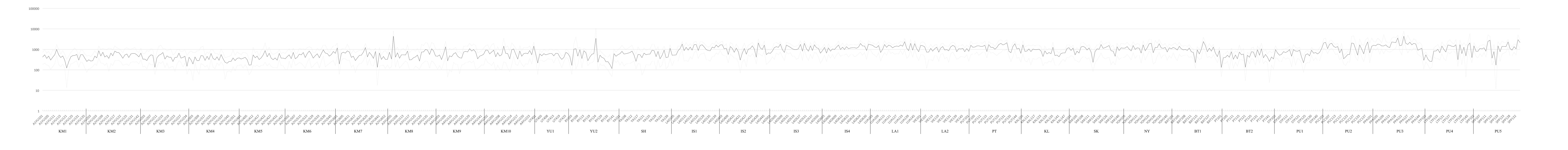
100000
10000
1000
100
10
1
KM1
KM2
KM3
KM4
KM5
KM6
KM7
KM8
KM9
KM10
YU1
YU2
SH
IS1
IS2
IS3
IS4
LA1
LA2
PT
KL
SK
NY
BT1
BT2
PU1
PU2
PU3
PU4
PU5

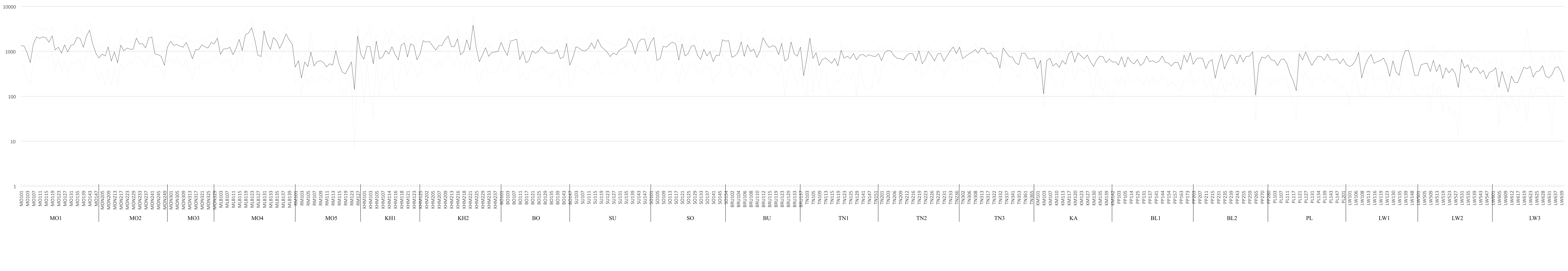
10000
1000
100
10
1
MO1
MO2
MO3
MO4
MO5
KH1
KH2
BO
SU
SO
BU
TN1
TN2
TN3
KA
BL1
BL2
PL
LW1
LW2
LW3
