# Supplementary material for: Complete mitochondrial genomes of Thai and Lao populations indicate an ancient origin of Austroasiatic groups and demic diffusion in the spread of Tai–Kadai languages
Source: Hum Genet. 2016 Nov 11;136(1):85–98. doi: 10.1007/s00439-016-1742-y (PMC5214972; doi:10.1007/s00439-016-1742-y)
Supplement: Supplementary file 3 — Supplementary material 3 (PDF 7072 kb) [file 439_2016_1742_MOESM3_ESM.pdf]

Online Resource 3 includes Figures S1-S7 with respective legends. Figure S1: Correspondence analysis (CA) based on haplogroup counts. Figure S2: Neighbor Joining tree based on the Φ_{st} genetic distance matrix for the populations in the present study and comparative populations. Figure S3: Bayesian maximum clade credibility (MCC) trees for 17 haplogroups (F1a, F1f, B5a, B4, B6a, R22, R9b, W3a1b, A, N9a, M7b1a, D, CZ, M20, M12-G, M24, and M74). Figure S4: Networks of 14 major haplogroups (F1a, F1f, B5a, B4, M7, D, M8, M12-G, M20, M24, M74, R9b, R22 and N9a) based on Thai/Lao mtDNA genomes and three haplogroups (B4c2, B6a and R22) based on sequences in Thai/Lao populations and other East Asian populations. Figure S5: The contour maps of the frequencies of 14 haplogroups (F1, F1f, B5, B5a1d, B4, B6a, D, M7, M12, M20, M24, M74, R9b and R22) in Thai/Lao populations. Figure S6: Bayesian skyline plots show the effective population size change over time in each studied population. Figure S7: Principal Component Analysis (PCA) plots reveal the fit between the simulated and observed data generated by the best 1,000 (or 5,000) simulations for each model.

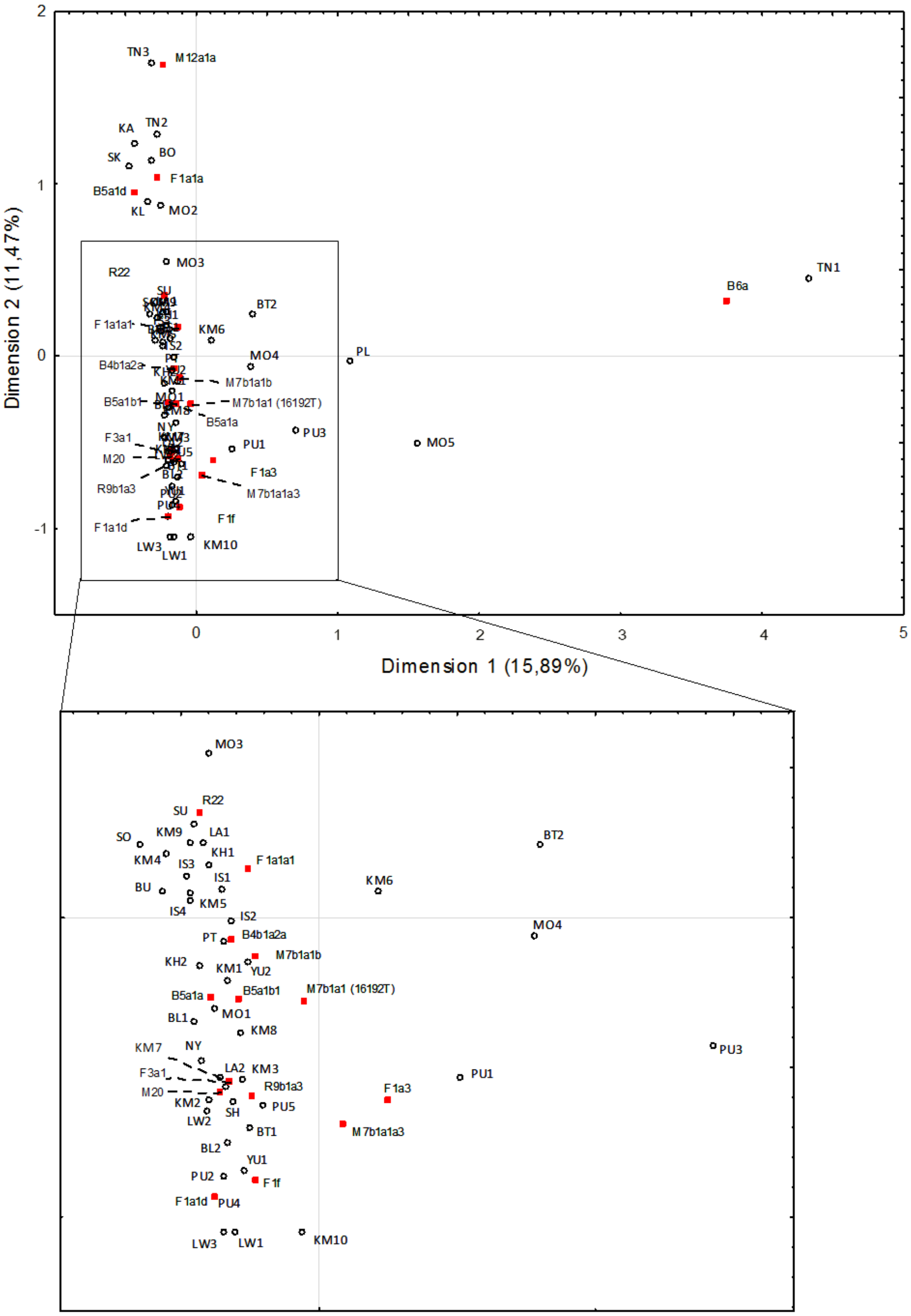

Dimension 2 (11,47%)
Dimension 1 (15,89%)
2
1
0
-1
0
1
2
3
4
5
TN3
M12a1a
KA
TN2
SK
BO
F1a1a
B5a1d
KL
MO2
MO3
R22
BT2
KM6
MO4
PL
B6a
TN1
F1a1a1
B4b1a2a
M7b1a1b
B5a1b1
M7b1a1 (16192T)
B5a1a
PU3
F3a1
PU1
MO5
M20
F1a3
R9b1a3
M7b1a1a3
F1f
F1a1d
LW3
LW1
KM10
SU
SO
KM9
LA1
KM4
IS3
KH1
IS1
BU
KM5
IS4
IS2
PT
KH2
KM1
YU2
MO1
BL1
KM8
KM7
NY
LA2
KM3
KM2
SH
PU5
LW2
BT1
BL2
YU1
PU2
PU4

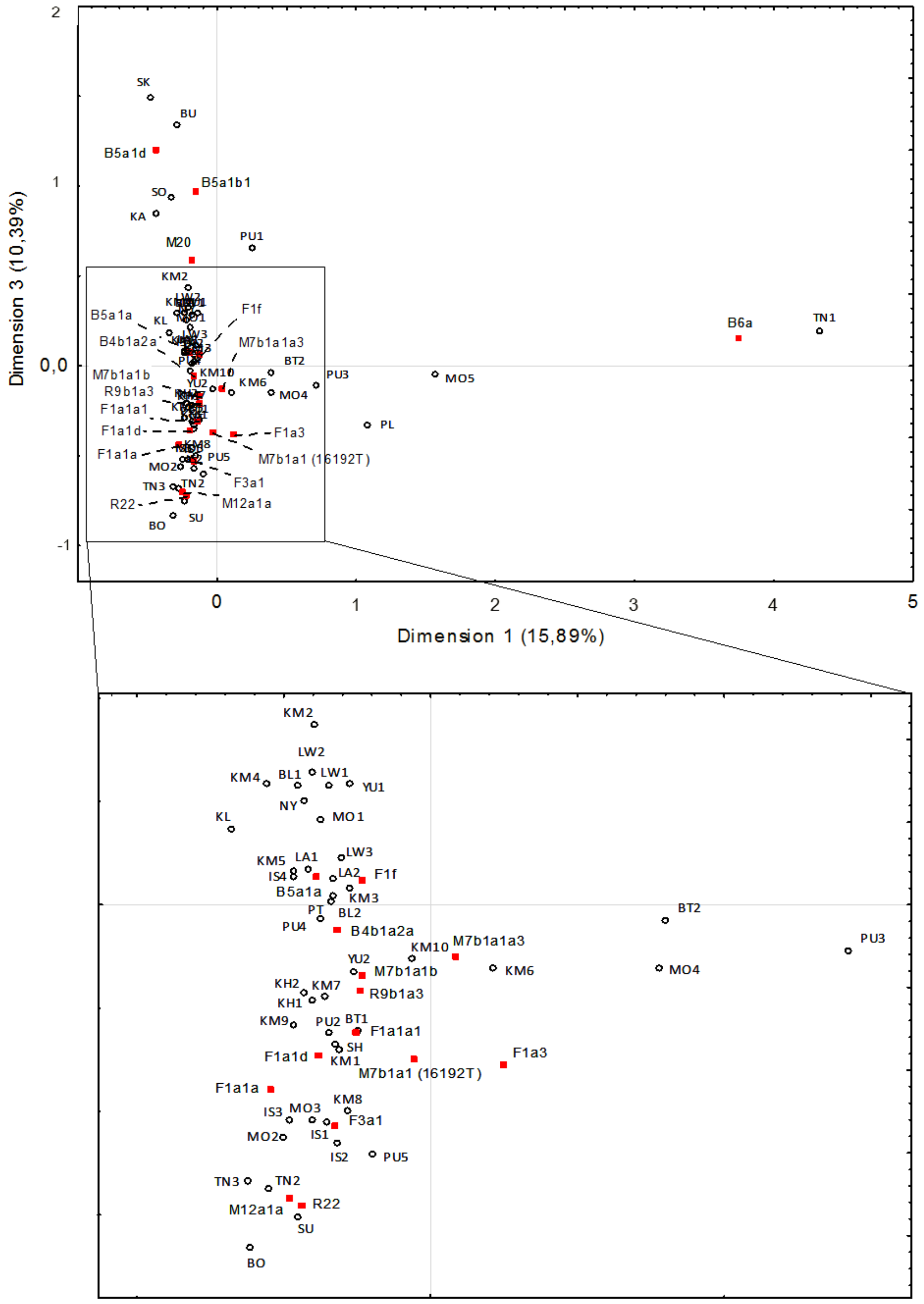

Fig. S1 Correspondence analysis (CA) based on haplogroup counts.

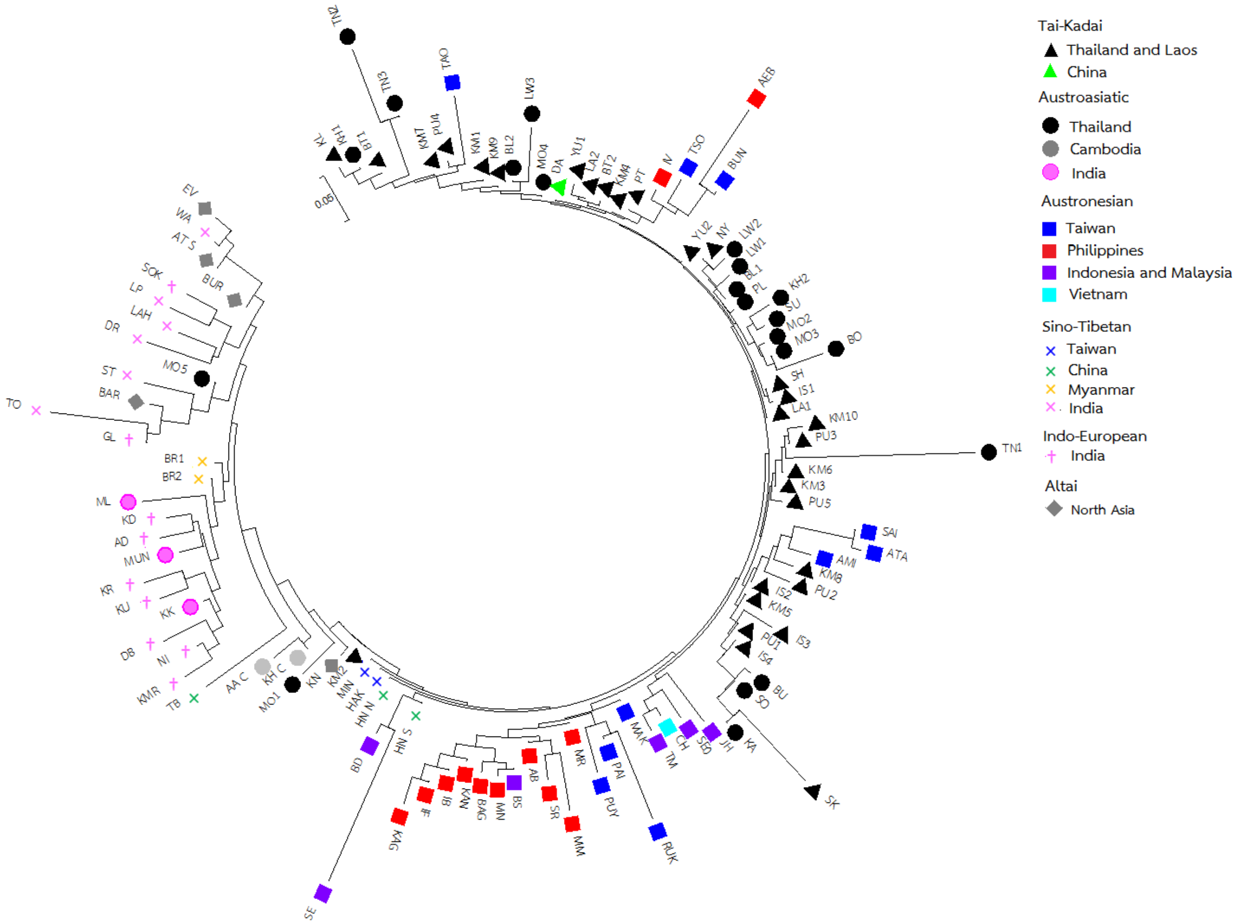

Fig. S2 Neighbor Joining tree based on the Φ_{st} genetic distance matrix for the populations in the present study and comparative populations. Population codes are provided in Fig. 1 and Table S2.

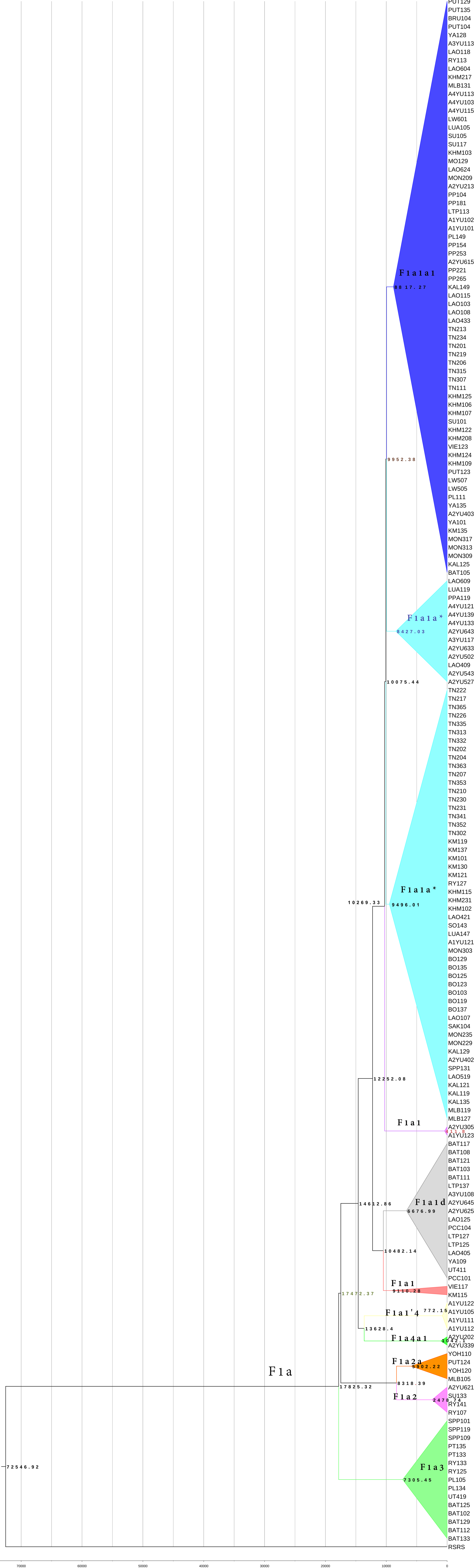

PUT135
BRU104
PUT104
YA128
A3YU113
LAO118
RY113
LAO604
KHM217
MLB131
A4YU113
A4YU103
A4YU115
LW601
LUA105
SU105
SU117
KHM103
MO129
LAO624
MON209
A2YU213
PP104
PP181
LTP113
A1YU102
A1YU101
PL149
PP154
PP253
A2YU615
PP221
PP265
KAL149
LAO115
LAO103
LAO108
LAO433
TN213
TN234
TN201
TN219
TN206
TN315
TN307
TN111
KHM125
KHM106
KHM107
SU101
KHM122
KHM208
VIE123
KHM124
KHM109
PUT123
LW507
LW505
PL111
YA135
A2YU403
YA101
KM135
MON317
MON313
MON309
KAL125
BAT105
LAO609
LUA119
PPA119
A4YU121
A4YU139
A4YU133
A2YU643
A3YU117
A2YU633
A2YU502
LAO409
A2YU543
A2YU527
TN222
TN217
TN365
TN226
TN335
TN313
TN332
TN202
TN204
TN363
TN207
TN353
TN210
TN230
TN231
TN341
TN352
TN302
KM119
KM137
KM101
KM130
KM121
RY127
KHM115
KHM231
KHM102
LAO421
SO143
LUA147
A1YU121
MON303
BO129
BO135
BO125
BO123
BO103
BO119
BO137
LAO107
SAK104
MON235
MON229
KAL129
A2YU402
SPP131
LAO519
KAL121
KAL119
KAL135
MLB119
MLB127
A2YU305
A1YU123
BAT117
BAT108
BAT121
BAT103
BAT111
LTP137
A3YU108
A2YU645
A2YU625
LAO125
PCC104
LTP127
LTP125
LAO405
YA109
UT411
PCC101
VIE117
KM115
A1YU122
A1YU105
A1YU111
A1YU112
A2YU202
A2YU339
YOH110
PUT124
YOH120
MLB105
A2YU621
SU133
RY141
RY107
SPP101
SPP119
SPP109
PT135
PT133
RY133
RY125
PL105
PL134
UT419
BAT125
BAT102
BAT129
BAT112
BAT133
RSRS
F1a1a1
9952.38
F1a1a*
8427.03
10075.44
F1a1a*
10269.33
9496.01
12252.08
F1a1
F1a1d
14612.86
6676.99
10482.14
F1a1
9110.28
17472.37
F1a1'4
772.15
13628.4
F1a4a1
F1a2a
5902.22
F1a
8318.39
17825.32
F1a2
F1a3
72546.92
7305.45
70000
60000
50000
40000
30000
20000
10000
0

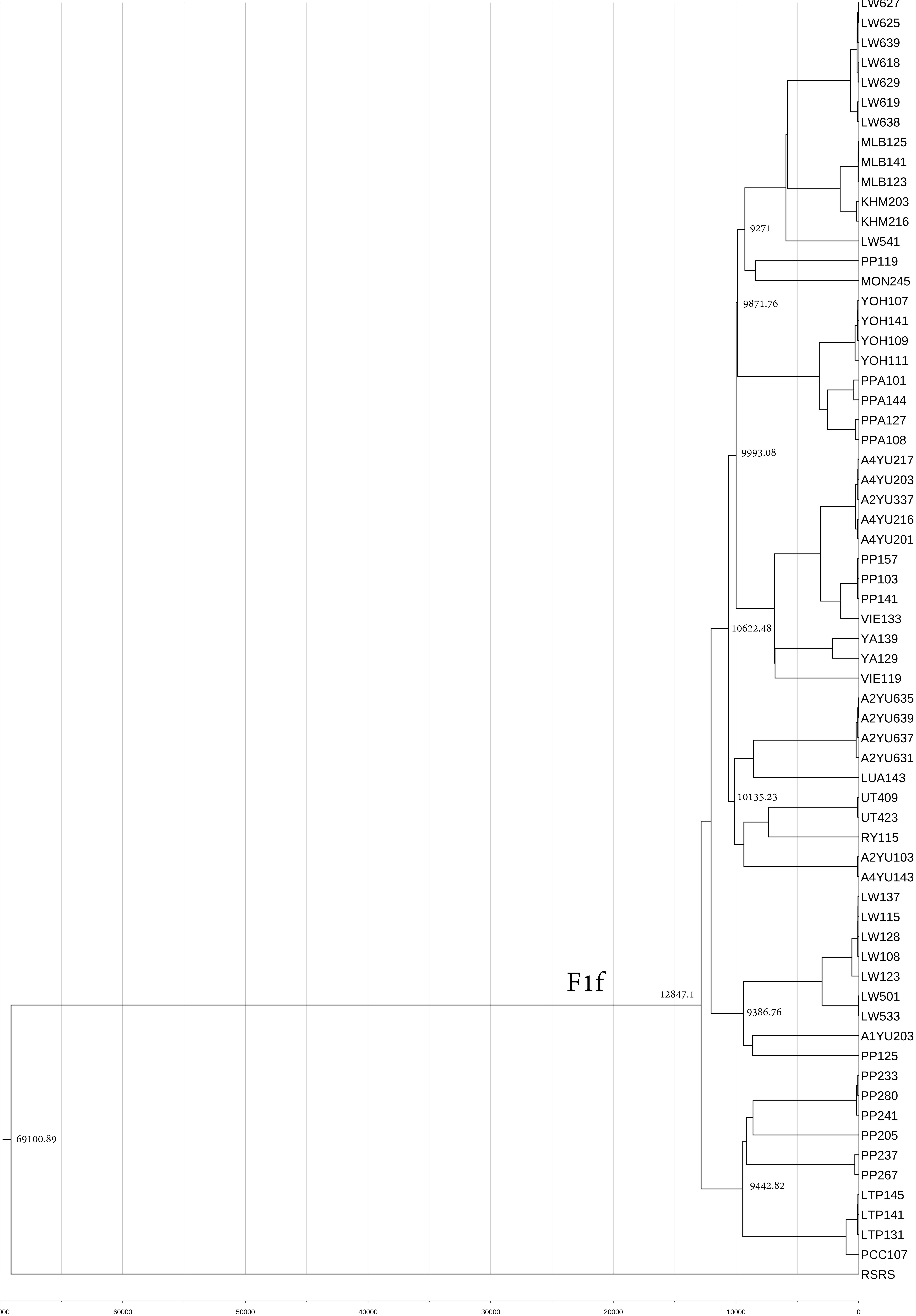

LW627
LW625
LW639
LW618
LW629
LW619
LW638
MLB125
MLB141
MLB123
KHM203
KHM216
LW541
PP119
MON245
YOH107
YOH141
YOH109
YOH111
PPA101
PPA144
PPA127
PPA108
A4YU217
A4YU203
A2YU337
A4YU216
A4YU201
PP157
PP103
PP141
VIE133
YA139
YA129
VIE119
A2YU635
A2YU639
A2YU637
A2YU631
LUA143
UT409
UT423
RY115
A2YU103
A4YU143
LW137
LW115
LW128
LW108
LW123
LW501
LW533
A1YU203
PP125
PP233
PP280
PP241
PP205
PP237
PP267
LTP145
LTP141
LTP131
PCC107
RSRS
9271
9871.76
9993.08
10622.48
10135.23
9386.76
9442.82
12847.1
F1f
69100.89
60000
50000
40000
30000
20000
10000
0

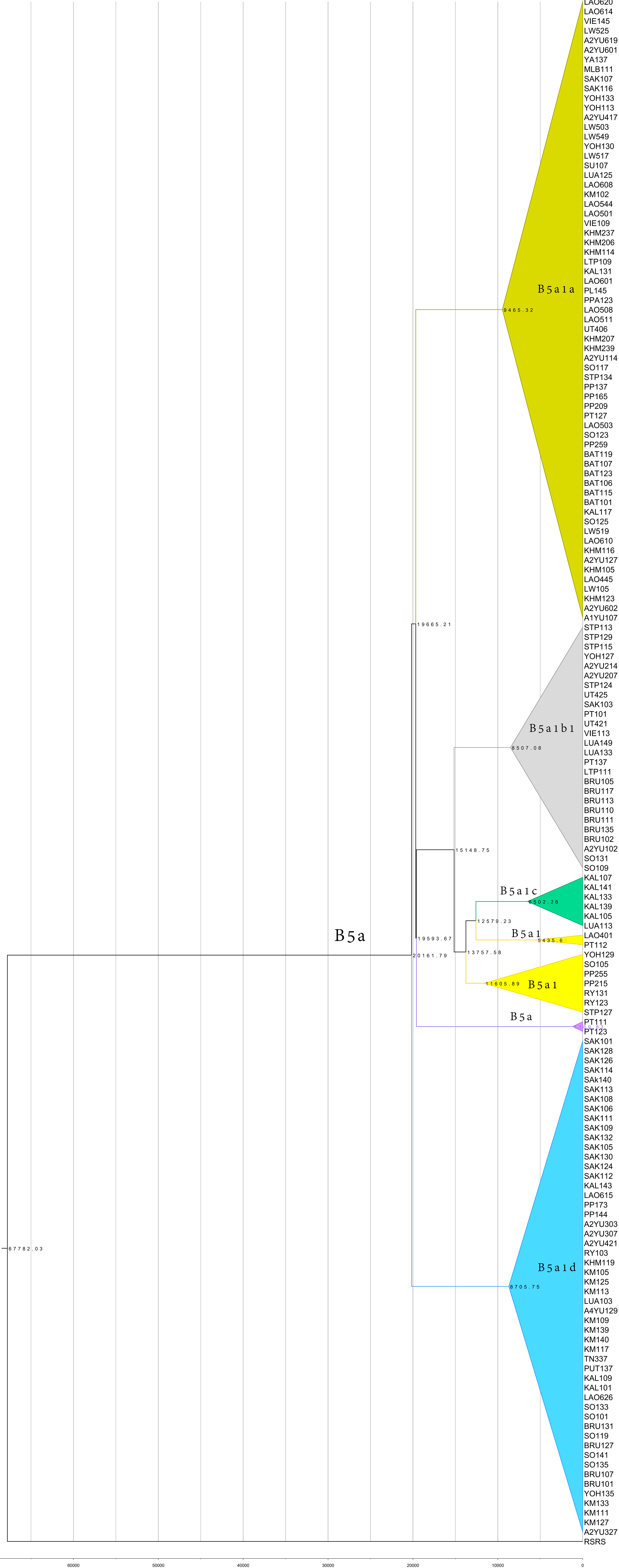
LAO620
LAO614
VIE145
LW525
A2YU619
A2YU601
YA137
MLB111
SAK107
SAK116
YOH133
YOH113
A2YU417
LW503
LW549
YOH130
LW517
SU107
LUA125
LAO608
KM102
LAO544
LAO501
VIE109
KHM237
KHM206
KHM114
LTP109
KAL131
LAO601
PL145
PPA123
LAO508
LAO511
UT406
KHM207
KHM239
A2YU114
SO117
STP134
PP137
PP165
PP209
PT127
LAO503
SO123
PP259
BAT119
BAT107
BAT123
BAT106
BAT115
BAT101
KAL117
SO125
LW519
LAO610
KHM116
A2YU127
KHM105
LAO445
LW105
KHM123
A2YU602
A1YU107
STP113
STP129
STP115
YOH127
A2YU214
A2YU207
STP124
UT425
SAK103
PT101
UT421
VIE113
LUA149
LUA133
PT137
LTP111
BRU105
BRU117
BRU113
BRU110
BRU111
BRU135
BRU102
A2YU102
SO131
SO109
KAL107
KAL141
KAL133
KAL139
KAL105
LUA113
LAO401
PT112
YOH129
SO105
PP255
PP215
RY131
RY123
STP127
PT111
PT123
SAK101
SAK128
SAK126
SAK114
SAk140
SAK113
SAK108
SAK106
SAK111
SAK109
SAK132
SAK105
SAK130
SAK124
SAK112
KAL143
LAO615
PP173
PP144
A2YU303
A2YU307
A2YU421
RY103
KHM119
KM105
KM125
KM113
LUA103
A4YU129
KM109
KM139
KM140
KM117
TN337
PUT137
KAL109
KAL101
LAO626
SO133
SO101
BRU131
SO119
BRU127
SO141
SO135
BRU107
BRU101
YOH135
KM133
KM111
KM127
A2YU327
RSRS
B5a1a
9465.32
19665.21
B5a1b1
8507.08
15148.75
B5a1c
6502.26
12579.23
B5a1
5435.6
B5a
19593.67
13757.58
20161.79
11605.89
B5a1
B5a
67782.03
B5a1d
8705.75
60000
50000
40000
30000
20000
10000
0

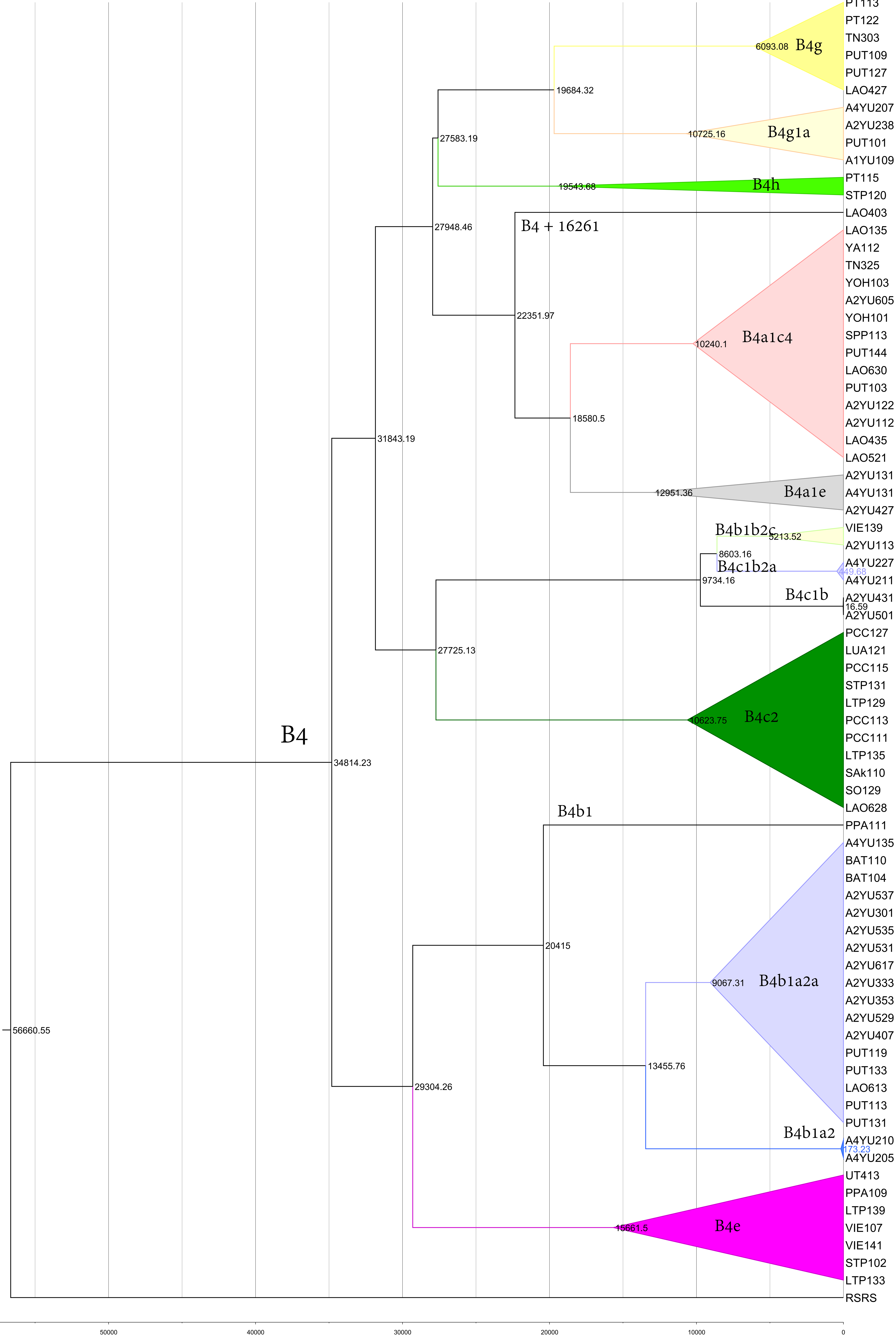

PT113
PT122
TN303
PUT109
PUT127
LAO427
A4YU207
A2YU238
PUT101
A1YU109
PT115
STP120
LAO403
LAO135
YA112
TN325
YOH103
A2YU605
YOH101
SPP113
PUT144
LAO630
PUT103
A2YU122
A2YU112
LAO435
LAO521
A2YU131
A4YU131
A2YU427
VIE139
A2YU113
A4YU227
A4YU211
A2YU431
A2YU501
PCC127
LUA121
PCC115
STP131
LTP129
PCC113
PCC111
LTP135
SAk110
SO129
LAO628
PPA111
A4YU135
BAT110
BAT104
A2YU537
A2YU301
A2YU535
A2YU531
A2YU617
A2YU333
A2YU353
A2YU529
A2YU407
PUT119
PUT133
LAO613
PUT113
PUT131
A4YU210
A4YU205
UT413
PPA109
LTP139
VIE107
VIE141
STP102
LTP133
RSRS
B4g
6093.08
19684.32
B4g1a
10725.16
27583.19
B4h
19543.68
27948.46
B4 + 16261
22351.97
B4a1c4
10240.1
18580.5
B4a1e
12951.36
31843.19
B4b1b2c
5213.52
8603.16
B4c1b2a
449.68
9734.16
B4c1b
16.59
27725.13
B4c2
10623.75
B4
34814.23
B4b1
20415
B4b1a2a
9067.31
13455.76
B4b1a2
173.23
29304.26
B4e
15661.5
56660.55
50000
40000
30000
20000
10000
0

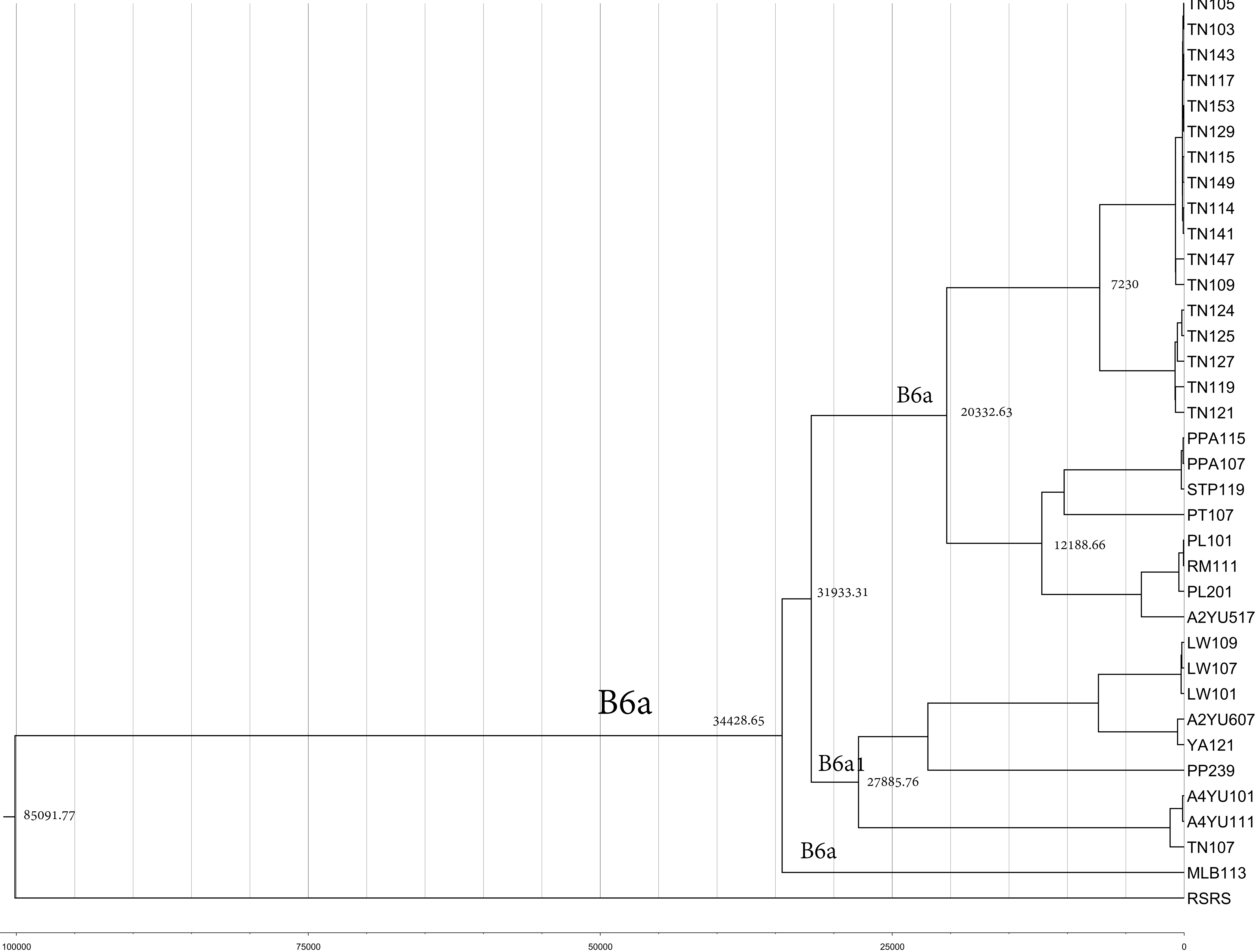

TN105
TN103
TN143
TN117
TN153
TN129
TN115
TN149
TN114
TN141
TN147
TN109
TN124
TN125
TN127
TN119
TN121
PPA115
PPA107
STP119
PT107
PL101
RM111
PL201
A2YU517
LW109
LW107
LW101
A2YU607
YA121
PP239
A4YU101
A4YU111
TN107
MLB113
RSRS
7230
B6a
20332.63
12188.66
31933.31
B6a
34428.65
B6a1
27885.76
B6a
85091.77
100000
75000
50000
25000
0

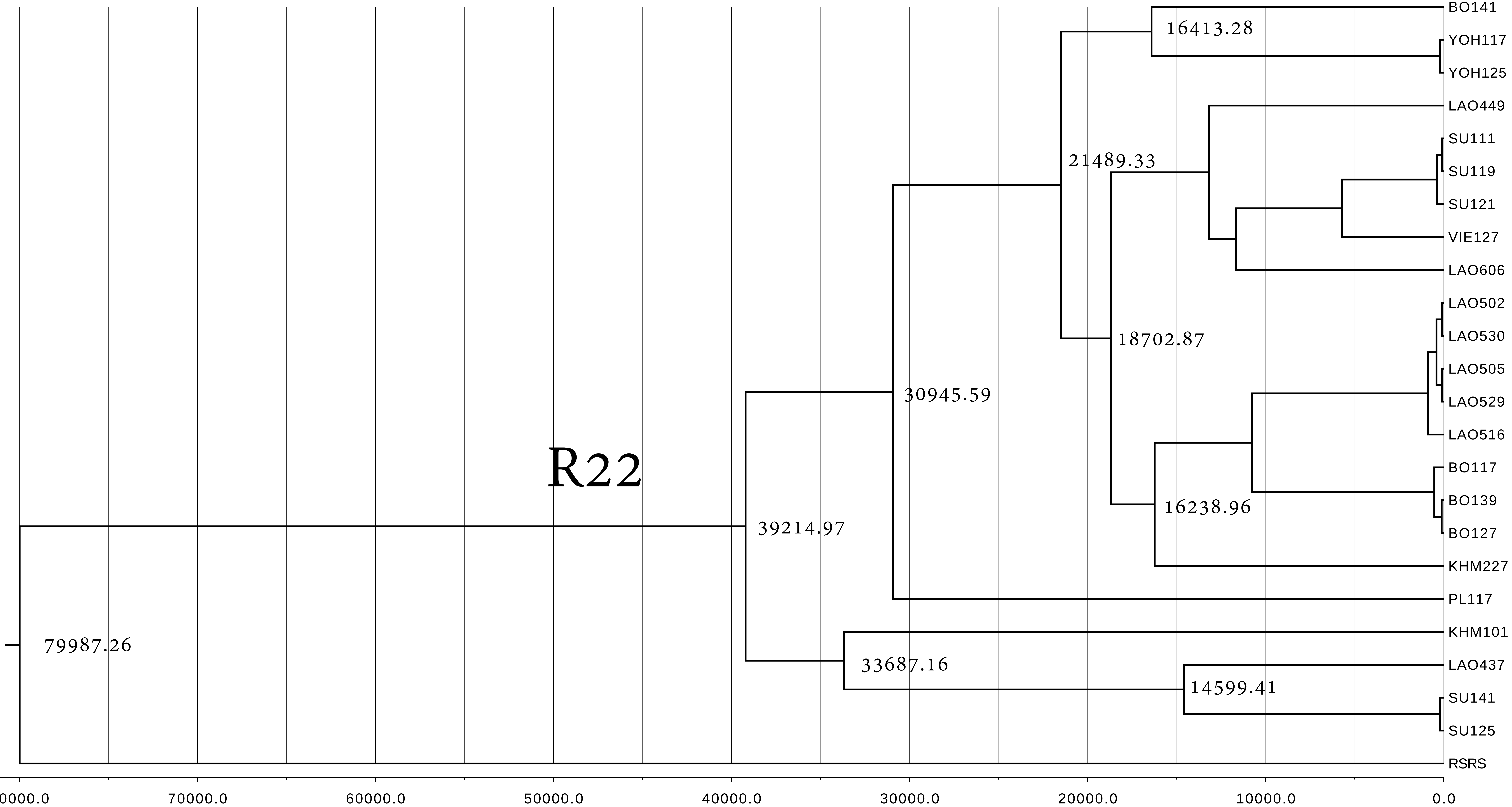

R22
BO141
YOH117
YOH125
LAO449
SU111
SU119
SU121
VIE127
LAO606
LAO502
LAO530
LAO505
LAO529
LAO516
BO117
BO139
BO127
KHM227
PL117
KHM101
LAO437
SU141
SU125
RSRS
16413.28
21489.33
18702.87
30945.59
16238.96
39214.97
79987.26
33687.16
14599.41
70000.0
60000.0
50000.0
40000.0
30000.0
20000.0
10000.0
0.0

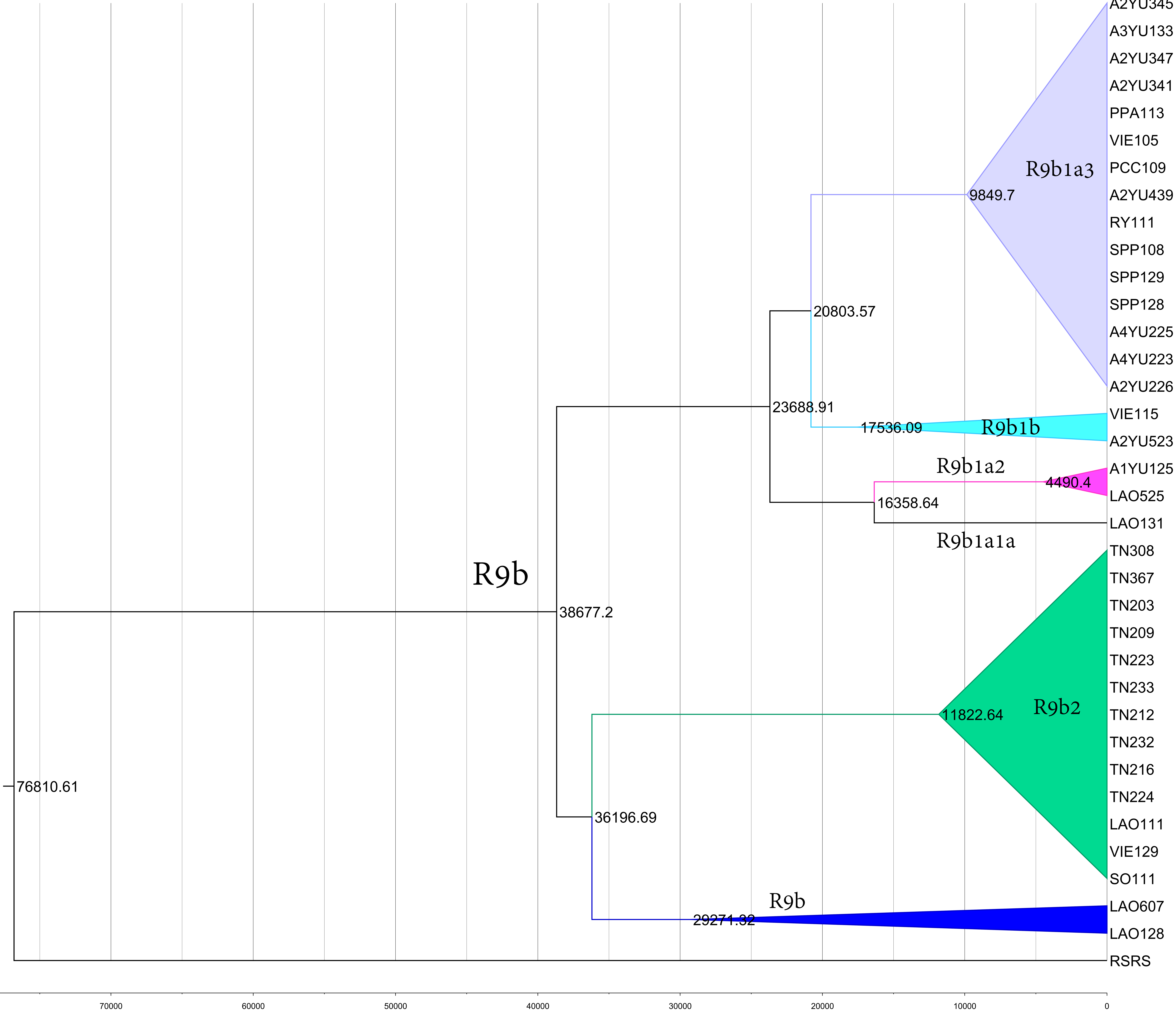

A2YU345
A3YU133
A2YU347
A2YU341
PPA113
VIE105
PCC109
A2YU439
RY111
SPP108
SPP129
SPP128
A4YU225
A4YU223
A2YU226
VIE115
A2YU523
A1YU125
LAO525
LAO131
TN308
TN367
TN203
TN209
TN223
TN233
TN212
TN232
TN216
TN224
LAO111
VIE129
SO111
LAO607
LAO128
RSRS
R9b1a3
9849.7
20803.57
23688.91
17536.09
R9b1b
R9b1a2
4490.4
16358.64
R9b1a1a
R9b
38677.2
11822.64
R9b2
76810.61
36196.69
R9b
29271.32
70000
60000
50000
40000
30000
20000
10000
0

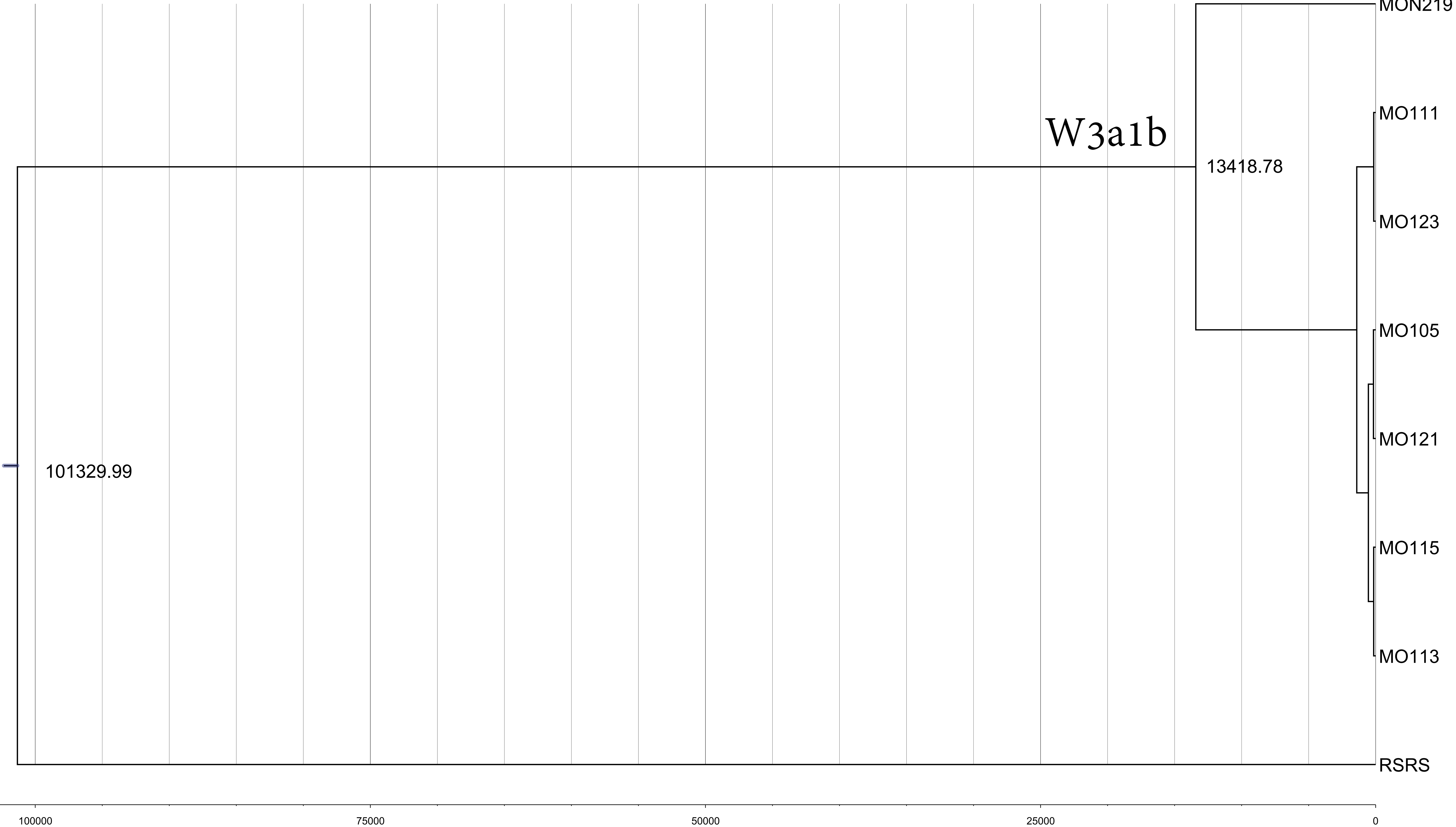

W3a1b
MON219
MO111
MO123
MO105
MO121
MO115
MO113
RSRS
13418.78
101329.99
100000
75000
50000
25000
0

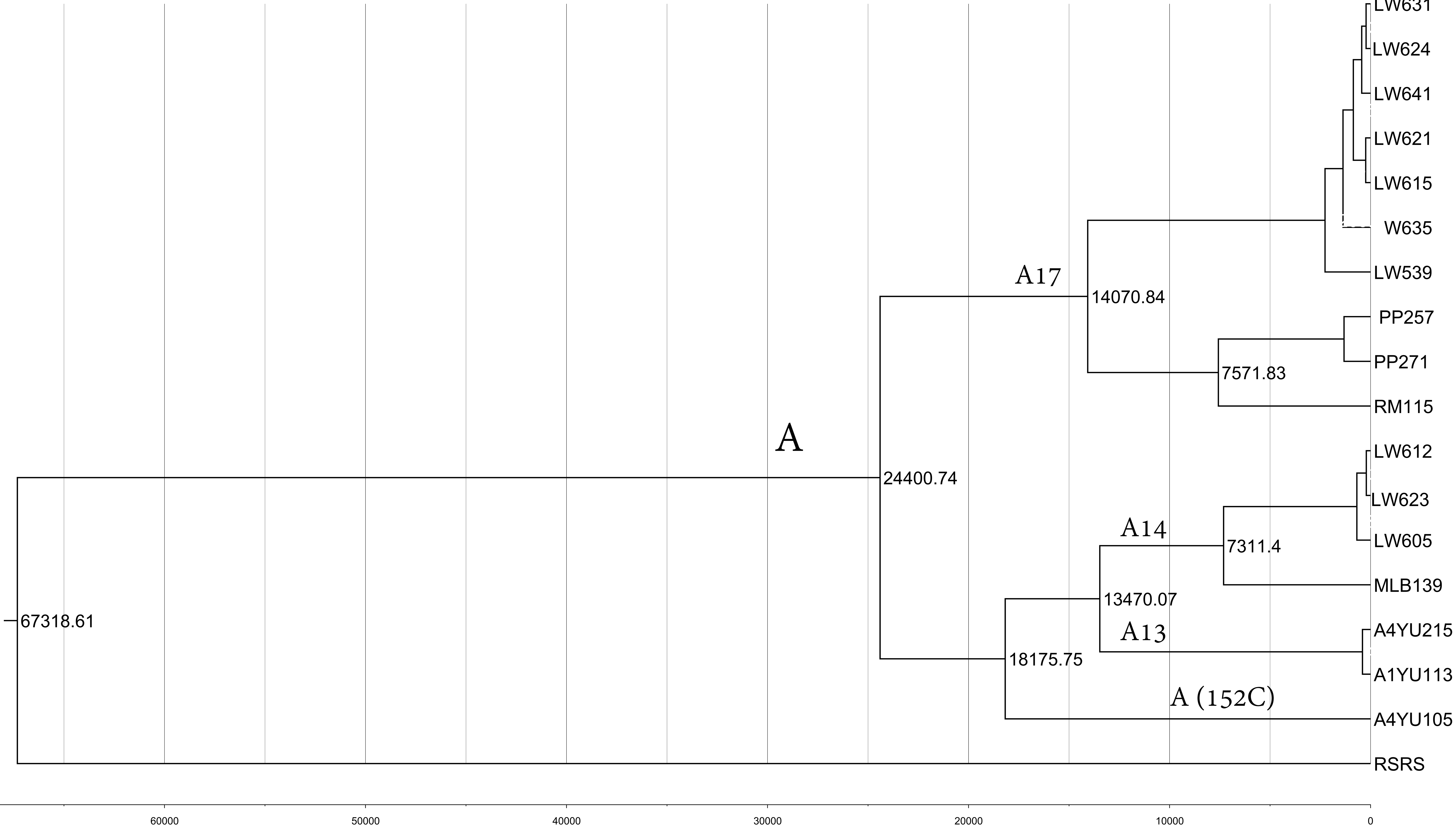
LW631
LW624
LW641
LW621
LW615
W635
LW539
A17
14070.84
PP257
PP271
7571.83
RM115
A
24400.74
LW612
LW623
A14
7311.4
LW605
MLB139
13470.07
A13
A4YU215
A1YU113
18175.75
A (152C)
A4YU105
67318.61
RSRS
60000
50000
40000
30000
20000
10000
0

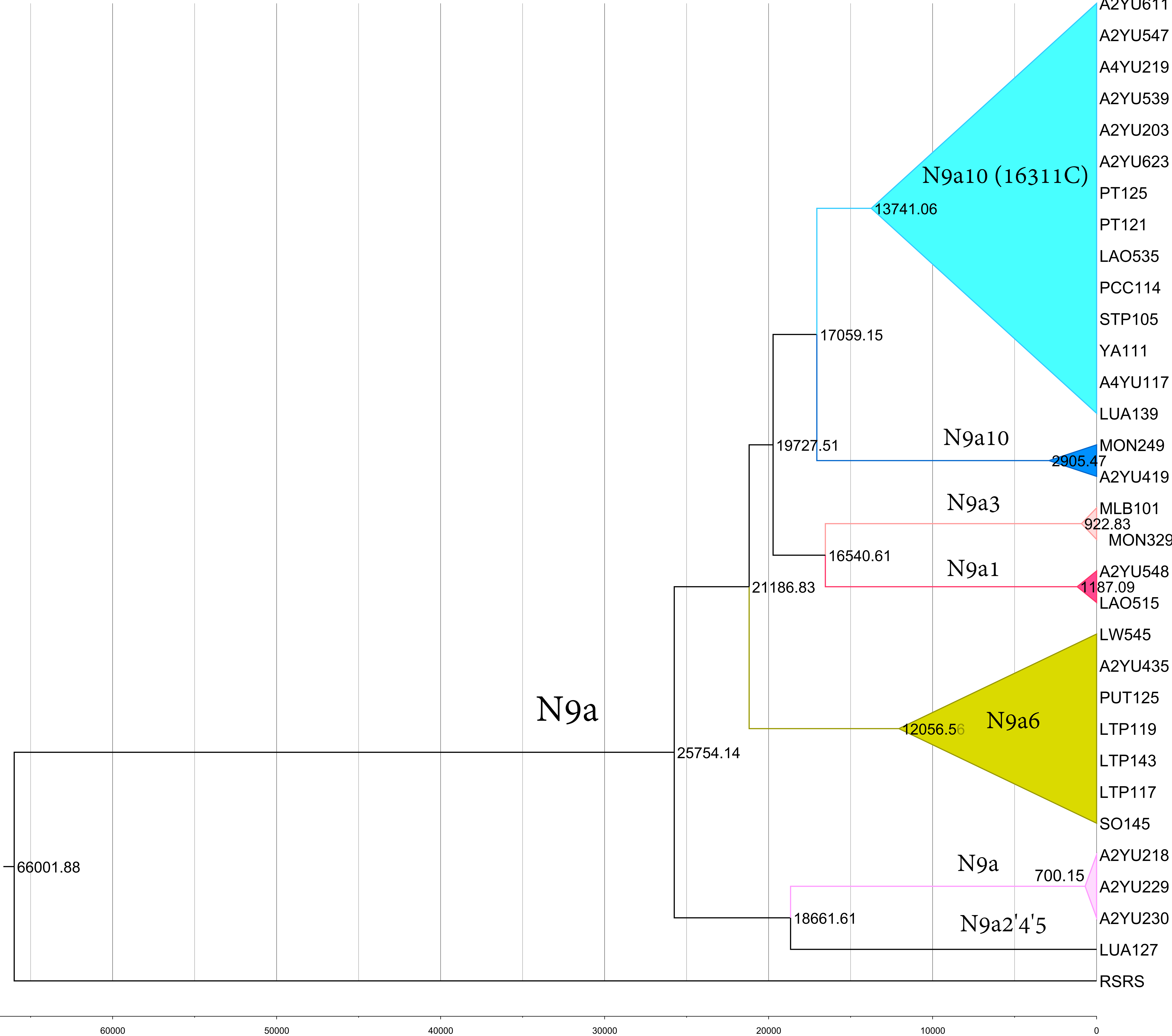

A2YU611
A2YU547
A4YU219
A2YU539
A2YU203
A2YU623
PT125
PT121
LAO535
PCC114
STP105
YA111
A4YU117
LUA139
N9a10 (16311C)
13741.06
17059.15
19727.51
N9a10
2905.47
MON249
A2YU419
N9a3
MLB101
922.83
MON329
16540.61
N9a1
A2YU548
1187.09
LAO515
21186.83
LW545
A2YU435
PUT125
LTP119
LTP143
LTP117
SO145
N9a6
12056.56
N9a
25754.14
66001.88
N9a
700.15
A2YU218
A2YU229
A2YU230
18661.61
N9a2'4'5
LUA127
RSRS
60000
50000
40000
30000
20000
10000
0

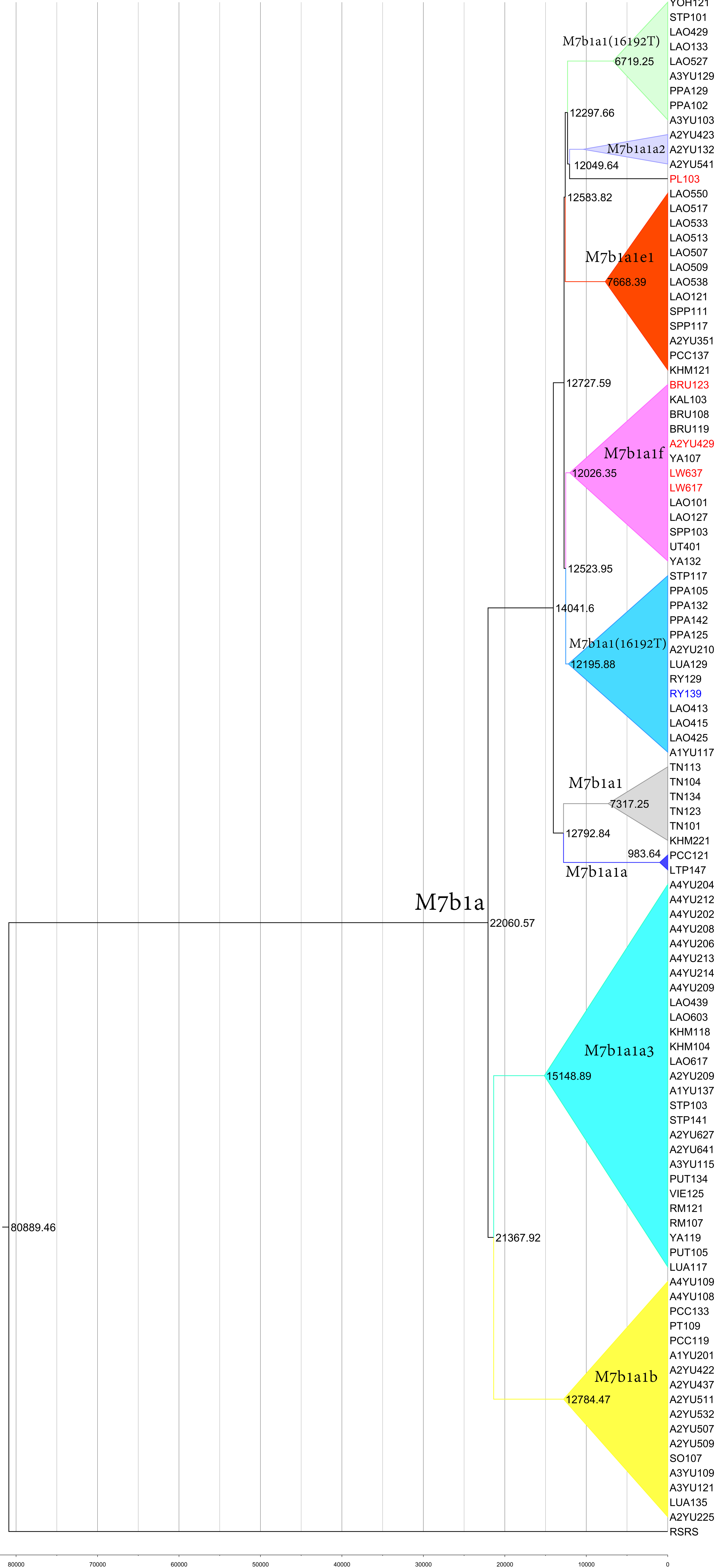

YOH121
STP101
LAO429
LAO133
LAO527
A3YU129
PPA129
PPA102
A3YU103
A2YU423
A2YU132
A2YU541
PL103
LAO550
LAO517
LAO533
LAO513
LAO507
LAO509
LAO538
LAO121
SPP111
SPP117
A2YU351
PCC137
KHM121
BRU123
KAL103
BRU108
BRU119
A2YU429
YA107
LW637
LW617
LAO101
LAO127
SPP103
UT401
YA132
STP117
PPA105
PPA132
PPA142
PPA125
A2YU210
LUA129
RY129
RY139
LAO413
LAO415
LAO425
A1YU117
TN113
TN104
TN134
TN123
TN101
KHM221
PCC121
LTP147
A4YU204
A4YU212
A4YU202
A4YU208
A4YU206
A4YU213
A4YU214
A4YU209
LAO439
LAO603
KHM118
KHM104
LAO617
A2YU209
A1YU137
STP103
STP141
A2YU627
A2YU641
A3YU115
PUT134
VIE125
RM121
RM107
YA119
PUT105
LUA117
A4YU109
A4YU108
PCC133
PT109
PCC119
A1YU201
A2YU422
A2YU437
A2YU511
A2YU532
A2YU507
A2YU509
SO107
A3YU109
A3YU121
LUA135
A2YU225
RSRS
M7b1a1(16192T)
6719.25
12297.66
M7b1a1a2
12049.64
12583.82
M7b1a1e1
7668.39
12727.59
M7b1a1f
12026.35
12523.95
14041.6
M7b1a1(16192T)
12195.88
M7b1a1
7317.25
12792.84
983.64
M7b1a1a
M7b1a
22060.57
M7b1a1a3
15148.89
80889.46
21367.92
M7b1a1b
12784.47
80000
70000
60000
50000
40000
30000
20000
10000
0

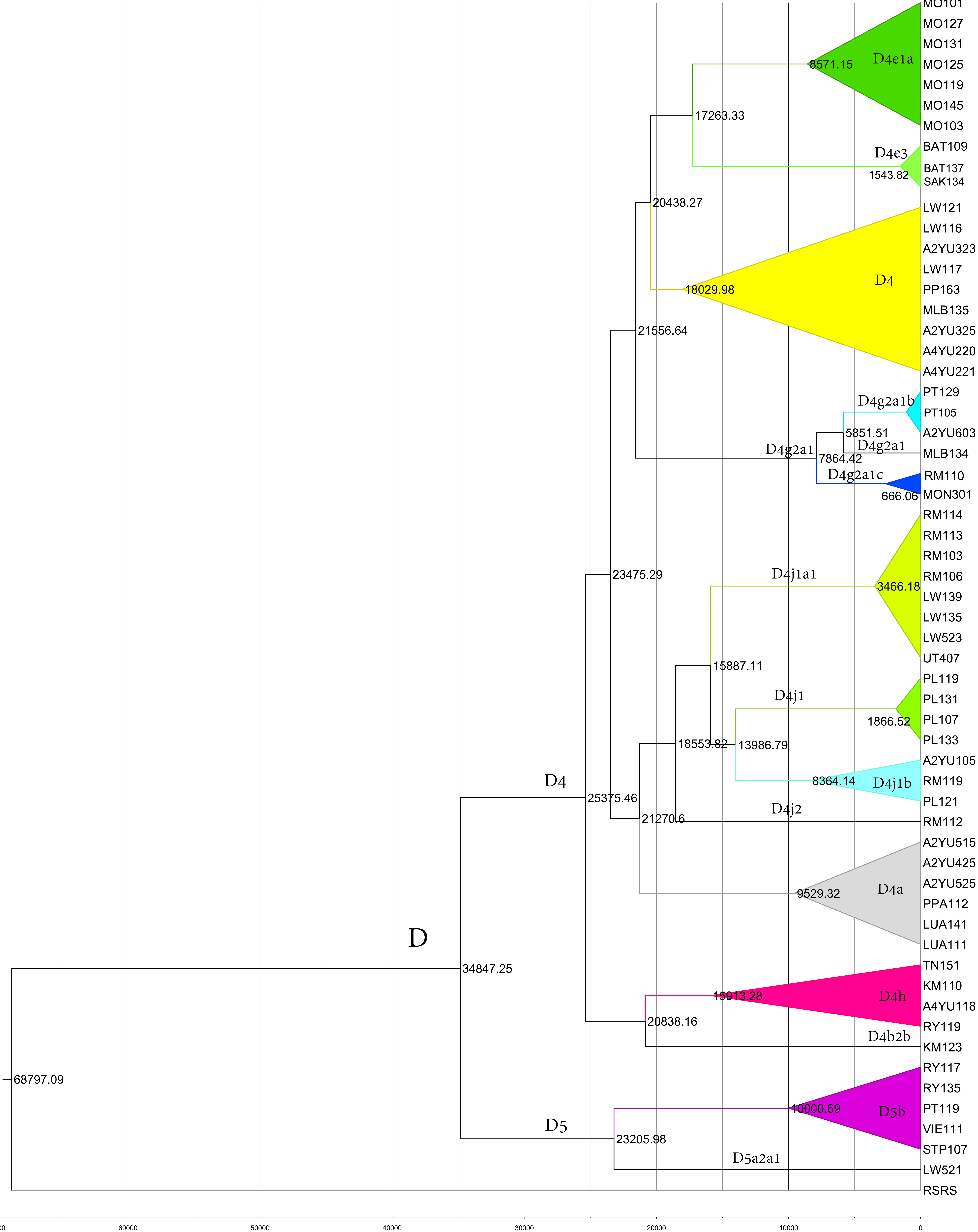

MO101
MO127
MO131
MO125
MO119
MO145
MO103
D4e1a
8571.15
17263.33
BAT109
BAT137
SAK134
D4e3
1543.82
20438.27
LW121
LW116
A2YU323
LW117
PP163
MLB135
A2YU325
A4YU220
A4YU221
D4
18029.98
21556.64
PT129
PT105
A2YU603
MLB134
D4g2a1b
5851.51
D4g2a1
D4g2a1
7864.42
D4g2a1c
RM110
MON301
666.06
RM114
RM113
RM103
RM106
LW139
LW135
LW523
UT407
D4j1a1
3466.18
23475.29
15887.11
PL119
PL131
PL107
PL133
D4j1
1866.52
18553.82
13986.79
A2YU105
RM119
PL121
8364.14
D4j1b
D4
25375.46
21270.6
D4j2
RM112
A2YU515
A2YU425
A2YU525
PPA112
LUA141
LUA111
9529.32
D4a
D
34847.25
TN151
KM110
A4YU118
RY119
15913.28
D4h
20838.16
D4b2b
KM123
68797.09
RY117
RY135
PT119
VIE111
STP107
10000.69
D5b
D5
23205.98
D5a2a1
LW521
RSRS
60000
50000
40000
30000
20000
10000
0

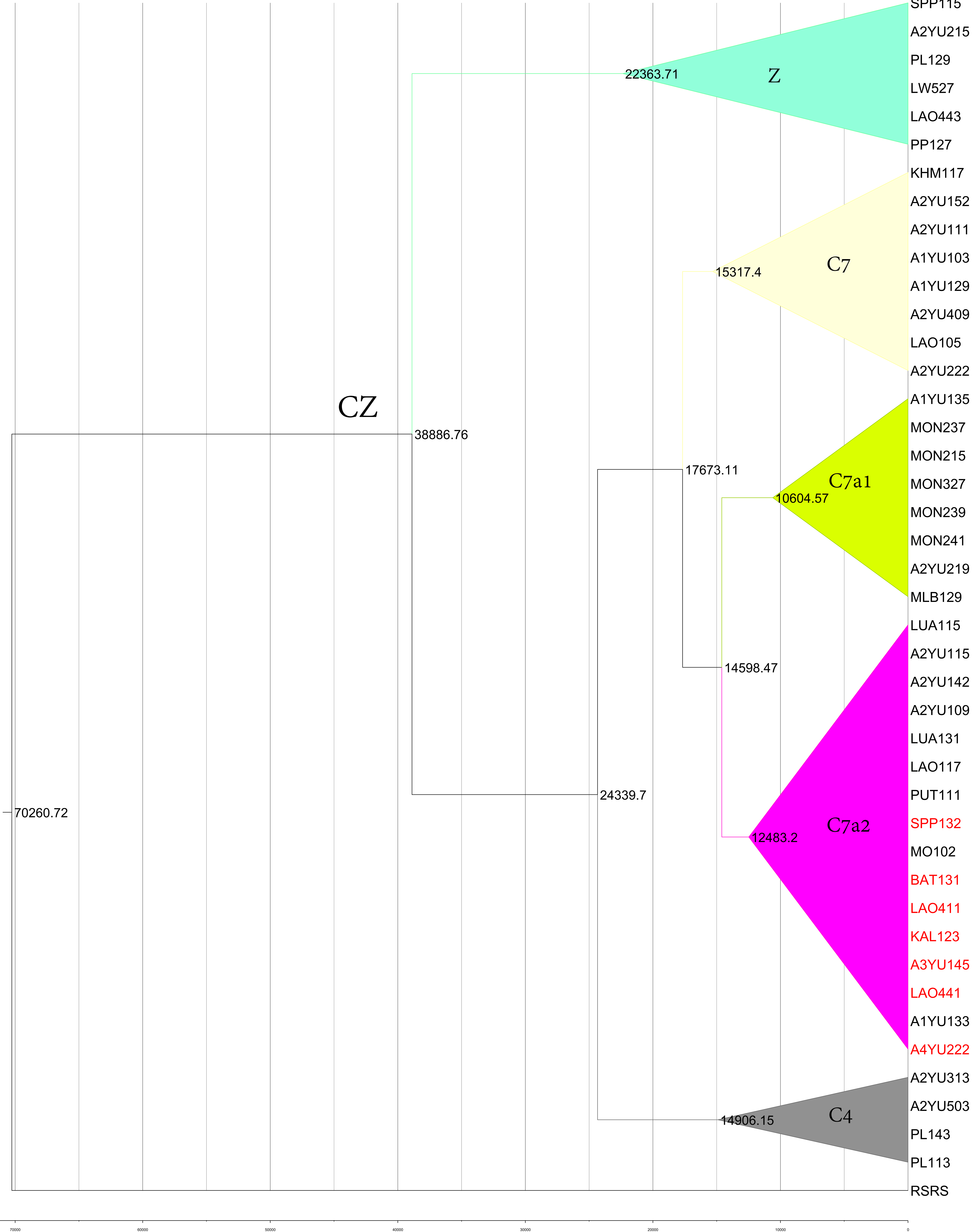

SPP115
A2YU215
PL129
LW527
LAO443
PP127
KHM117
A2YU152
A2YU111
A1YU103
A1YU129
A2YU409
LAO105
A2YU222
A1YU135
MON237
MON215
MON327
MON239
MON241
A2YU219
MLB129
LUA115
A2YU115
A2YU142
A2YU109
LUA131
LAO117
PUT111
SPP132
MO102
BAT131
LAO411
KAL123
A3YU145
LAO441
A1YU133
A4YU222
A2YU313
A2YU503
PL143
PL113
RSRS
Z
C7
C7a1
C7a2
C4
CZ
22363.71
15317.4
38886.76
17673.11
10604.57
14598.47
24339.7
70260.72
12483.2
14906.15
70000
60000
50000
40000
30000
20000
10000
0

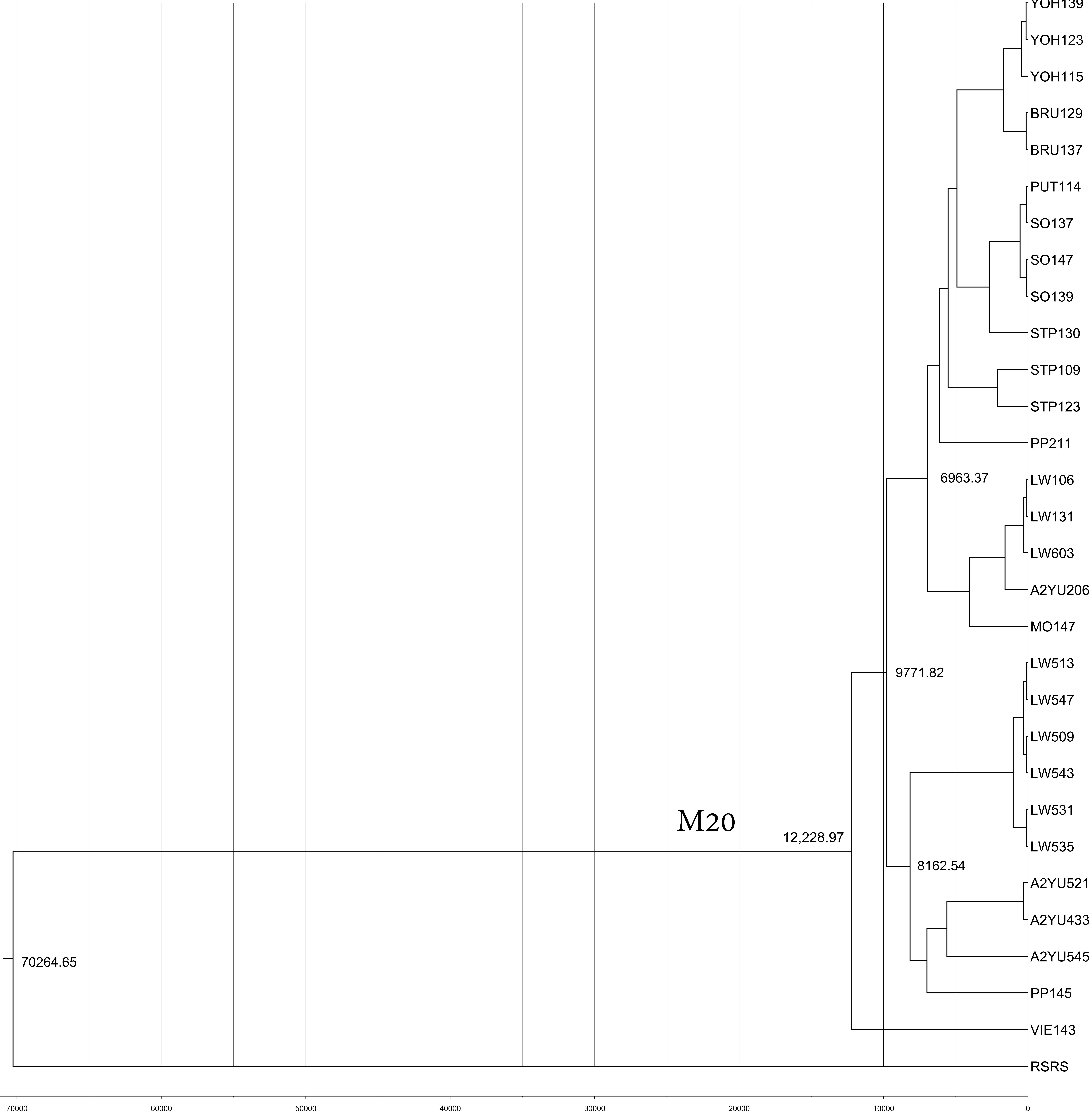

YOH139
YOH123
YOH115
BRU129
BRU137
PUT114
SO137
SO147
SO139
STP130
STP109
STP123
PP211
6963.37
LW106
LW131
LW603
A2YU206
MO147
LW513
9771.82
LW547
LW509
LW543
LW531
M20
12,228.97
LW535
8162.54
A2YU521
A2YU433
70264.65
A2YU545
PP145
VIE143
RSRS
70000
60000
50000
40000
30000
20000
10000
0

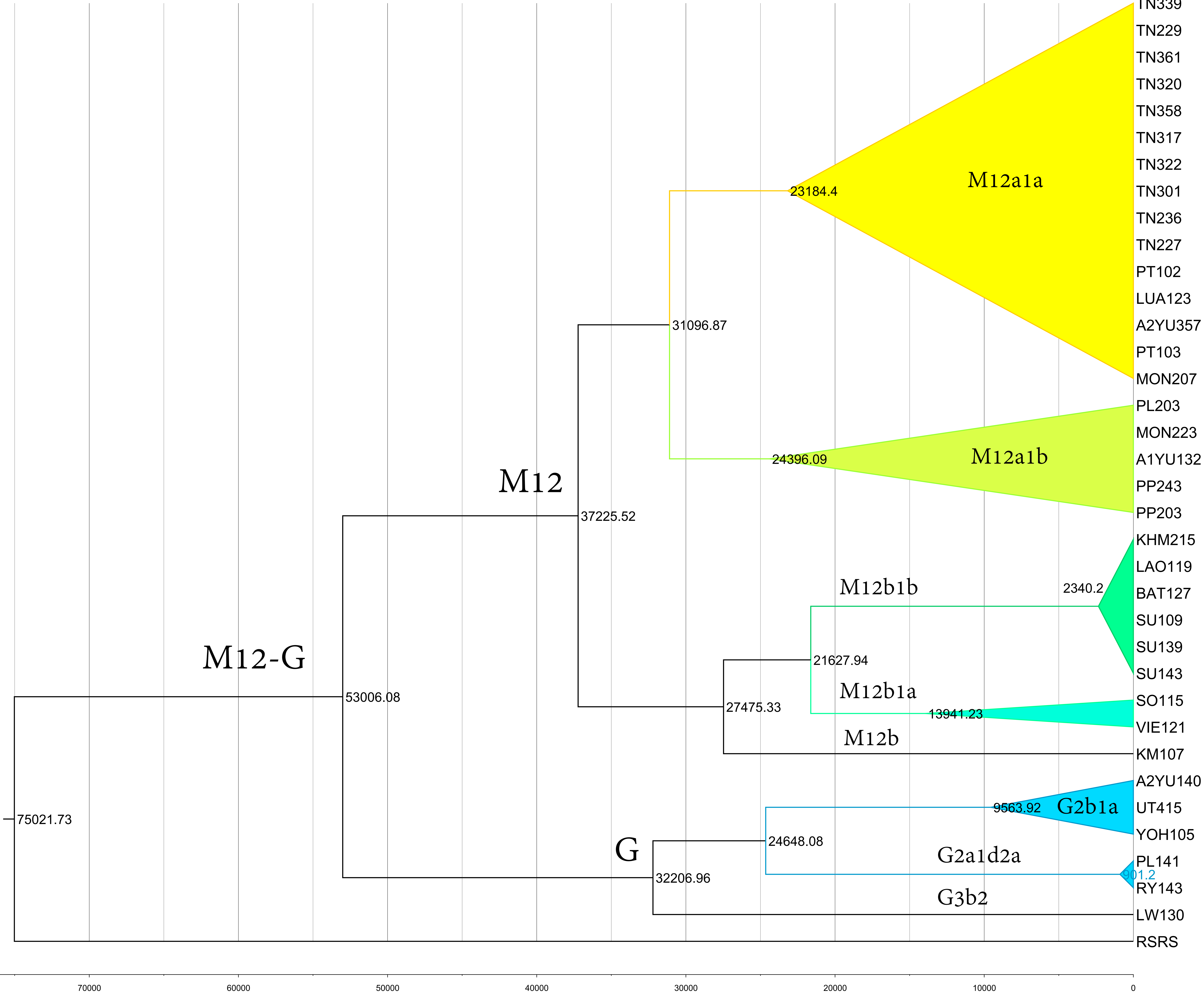

M12a1a
M12a1b
M12
M12-G
M12b1b
M12b1a
M12b
G2b1a
G2a1d2a
G3b2
G
23184.4
31096.87
24396.09
37225.52
2340.2
21627.94
27475.33
13941.23
53006.08
75021.73
9563.92
24648.08
901.2
32206.96
TN339
TN229
TN361
TN320
TN358
TN317
TN322
TN301
TN236
TN227
PT102
LUA123
A2YU357
PT103
MON207
PL203
MON223
A1YU132
PP243
PP203
KHM215
LAO119
BAT127
SU109
SU139
SU143
SO115
VIE121
KM107
A2YU140
UT415
YOH105
PL141
RY143
LW130
RSRS
70000
60000
50000
40000
30000
20000
10000
0

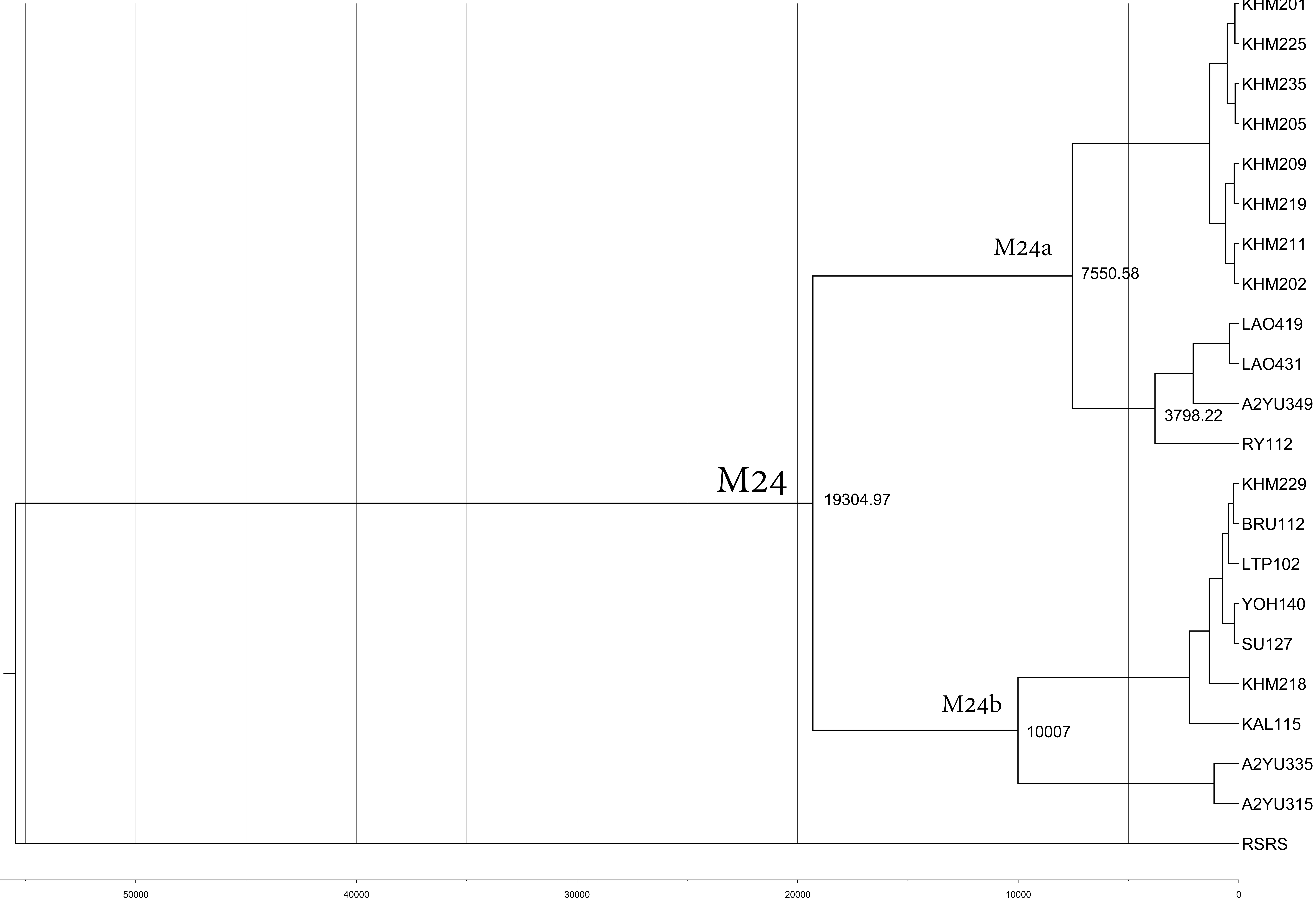

KHM201
KHM225
KHM235
KHM205
KHM209
KHM219
KHM211
KHM202
LAO419
LAO431
A2YU349
RY112
KHM229
BRU112
LTP102
YOH140
SU127
KHM218
KAL115
A2YU335
A2YU315
RSRS
M24a
7550.58
3798.22
M24
19304.97
M24b
10007
50000
40000
30000
20000
10000
0

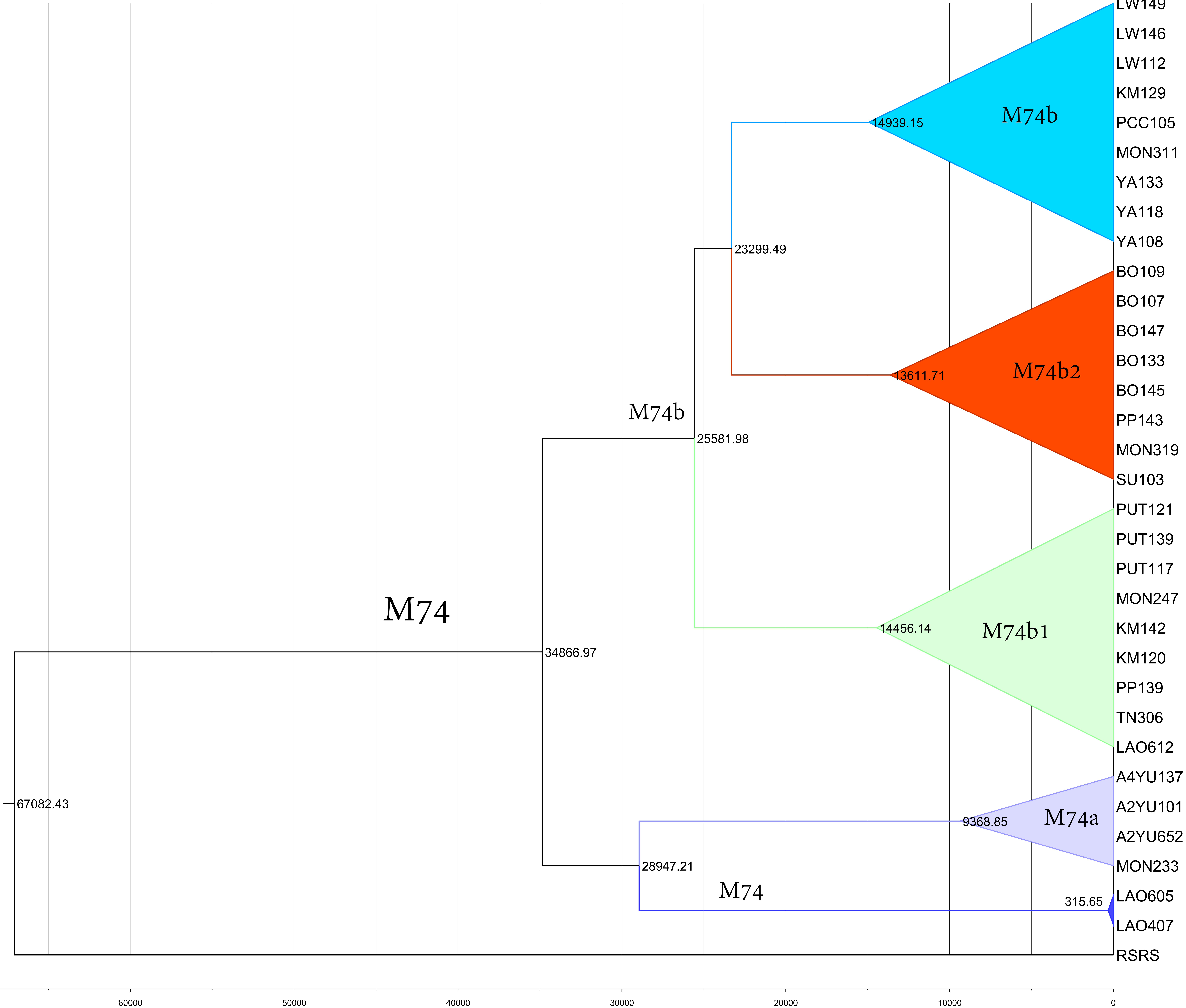

LW149
LW146
LW112
KM129
PCC105
MON311
YA133
YA118
YA108
BO109
BO107
BO147
BO133
BO145
PP143
MON319
SU103
PUT121
PUT139
PUT117
MON247
KM142
KM120
PP139
TN306
LAO612
A4YU137
A2YU101
A2YU652
MON233
LAO605
LAO407
RSRS
M74b
14939.15
23299.49
M74b2
13611.71
M74b
25581.98
M74
14456.14
M74b1
34866.97
67082.43
9368.85
M74a
28947.21
M74
315.65
60000
50000
40000
30000
20000
10000
0

Fig. S3 Bayesian maximum clade credibility (MCC) trees. MCC trees for 17 haplogroups (F1a, F1f, B5a, B4, B6a, R22, R9b, W3a1b, A, N9a, M7b1a, D, CZ, M20, M12-G, M24, and M74). Tree topology and coalescent time estimates were generated by BEAST and TreeAnnotator with parameters described in the Material and Methods. To aid visualization, some trees were schematically modified to combine individuals in the same lineage or sublineage by Figtree. Sample codes and RSRS are indicated at the tips of the tree.

F1a

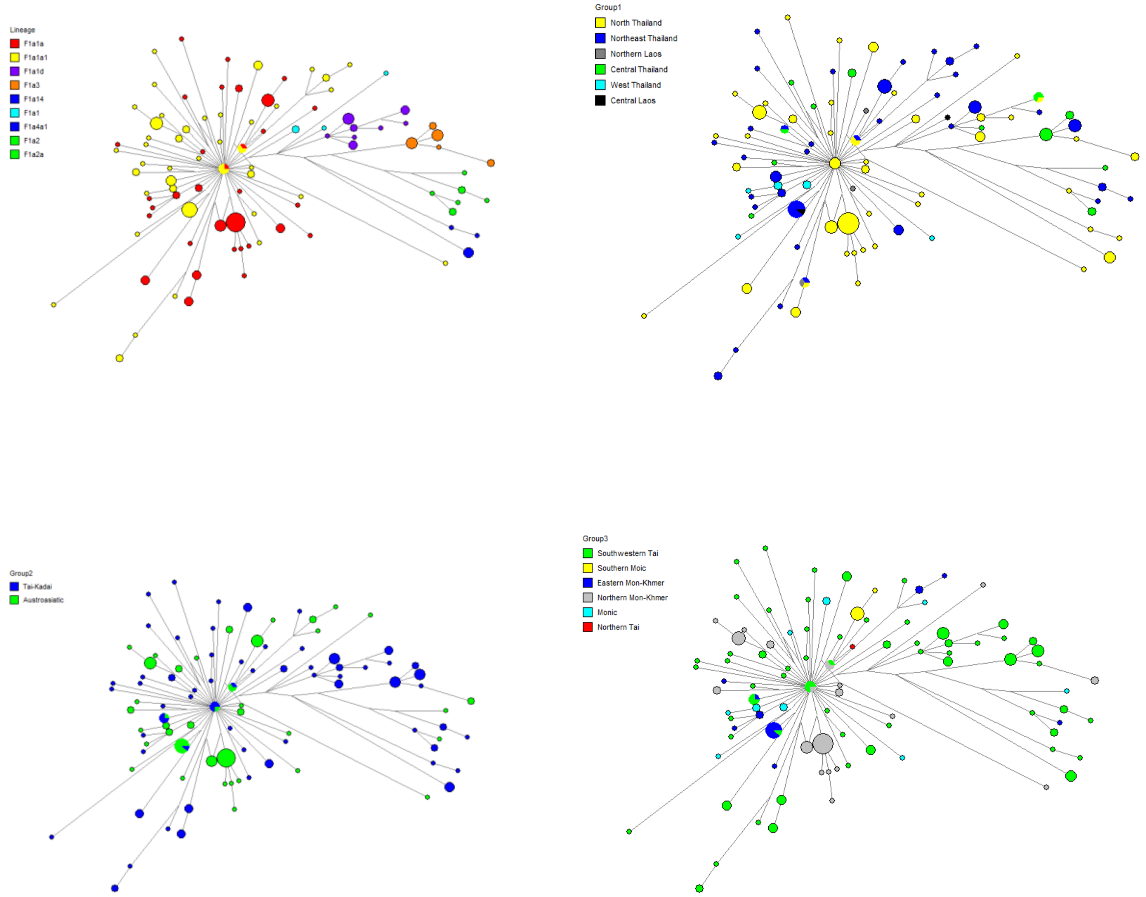
Lineage
F1a1a
F1a1a1
F1a1d
F1a3
F1a14
F1a1
F1a4a1
F1a2
F1a2a
Group1
North Thailand
Northeast Thailand
Northern Laos
Central Thailand
West Thailand
Central Laos
Group2
Tai-Kadai
Austroasiatic
Group3
Southwestern Tai
Southern Moic
Eastern Mon-Khmer
Northern Mon-Khmer
Monic
Northern Tai

F1f

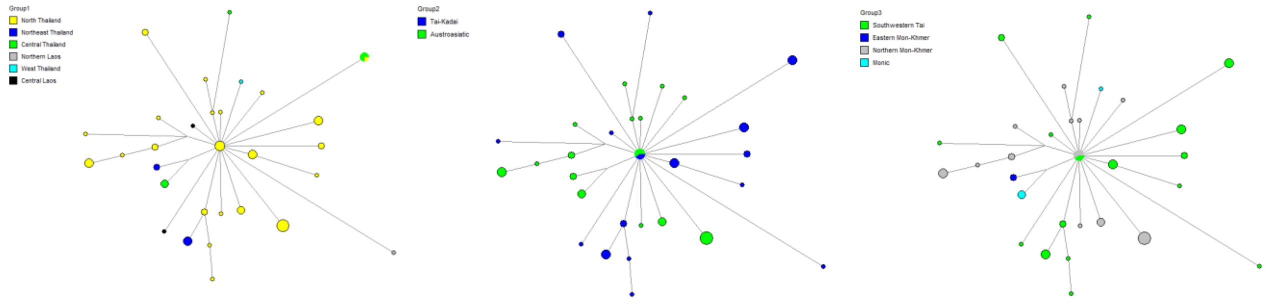
Group1
North Thailand
Northeast Thailand
Central Thailand
Northern Laos
West Thailand
Central Laos
Group2
Tai-Kadai
Austroasiatic
Group3
Southwestern Tai
Eastern Mon-Khmer
Northern Mon-Khmer
Monic

B5a

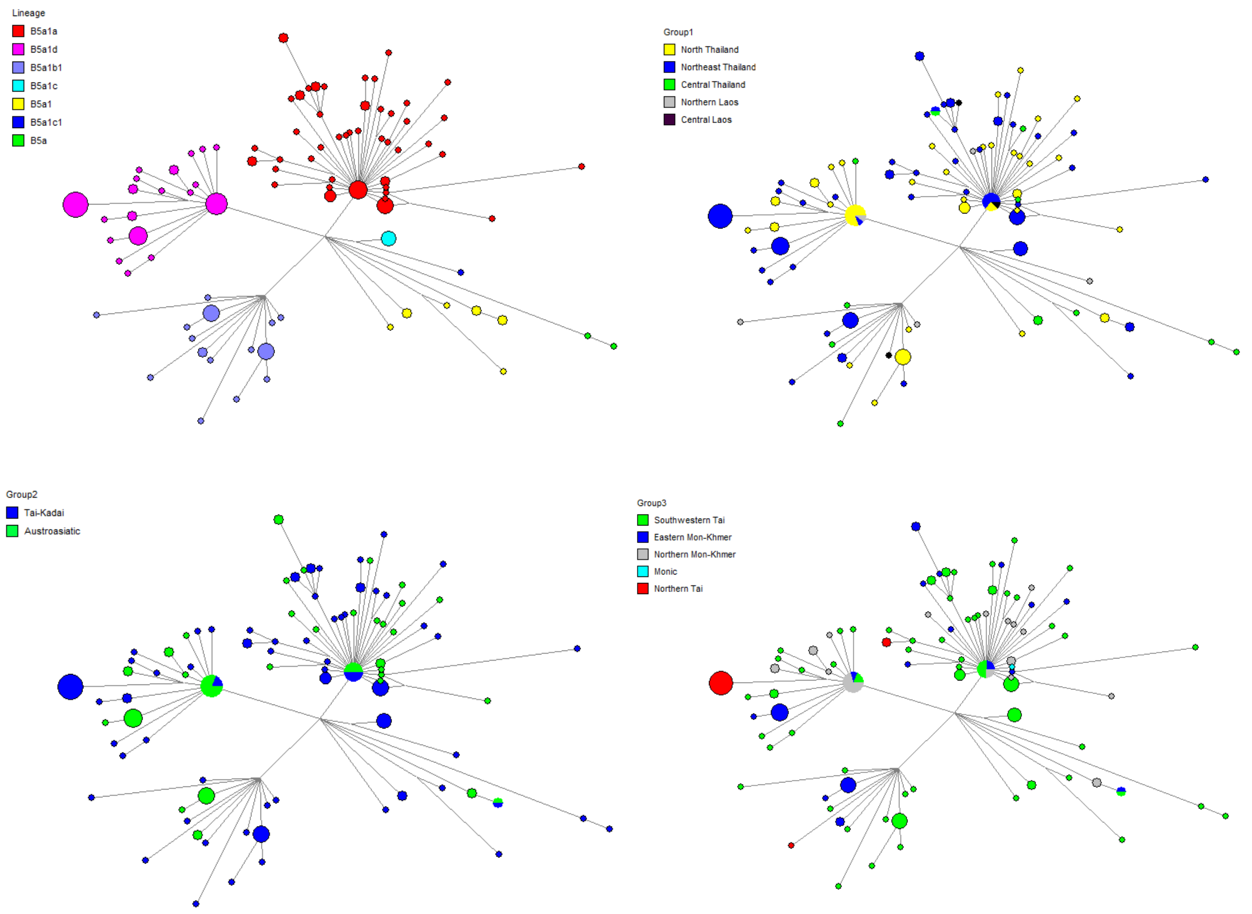
Lineage
B5a1a
B5a1d
B5a1b1
B5a1c
B5a1
B5a1c1
B5a
Group1
North Thailand
Northeast Thailand
Central Thailand
Northern Laos
Central Laos
Group2
Tai-Kadai
Austroasiatic
Group3
Southwestern Tai
Eastern Mon-Khmer
Northern Mon-Khmer
Monic
Northern Tai

B4

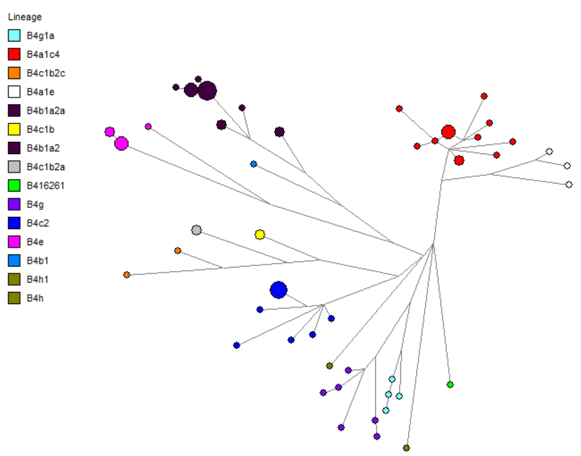
Lineage
B4g1a
B4a1c4
B4c1b2c
B4a1e
B4b1a2a
B4c1b
B4b1a2
B4c1b2a
B416261
B4g
B4c2
B4e
B4b1
B4h1
B4h

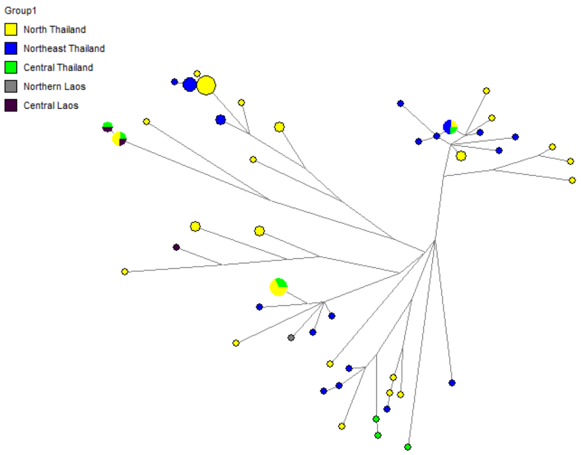
Group1
North Thailand
Northeast Thailand
Central Thailand
Northern Laos
Central Laos

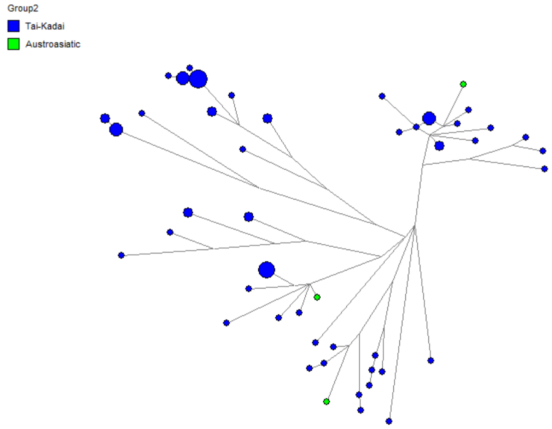
Group2
Tai-Kadai
Austroasiatic

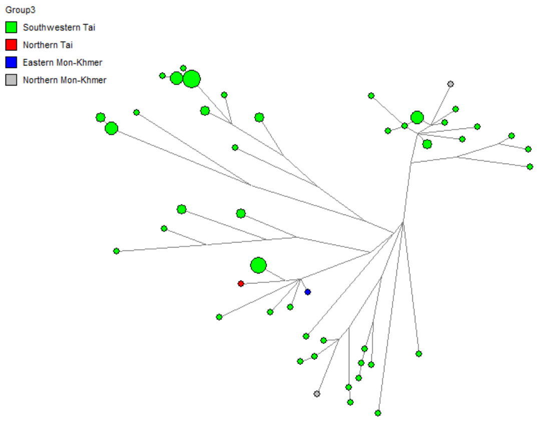
Group3
Southwestern Tai
Northern Tai
Eastern Mon-Khmer
Northern Mon-Khmer

M7

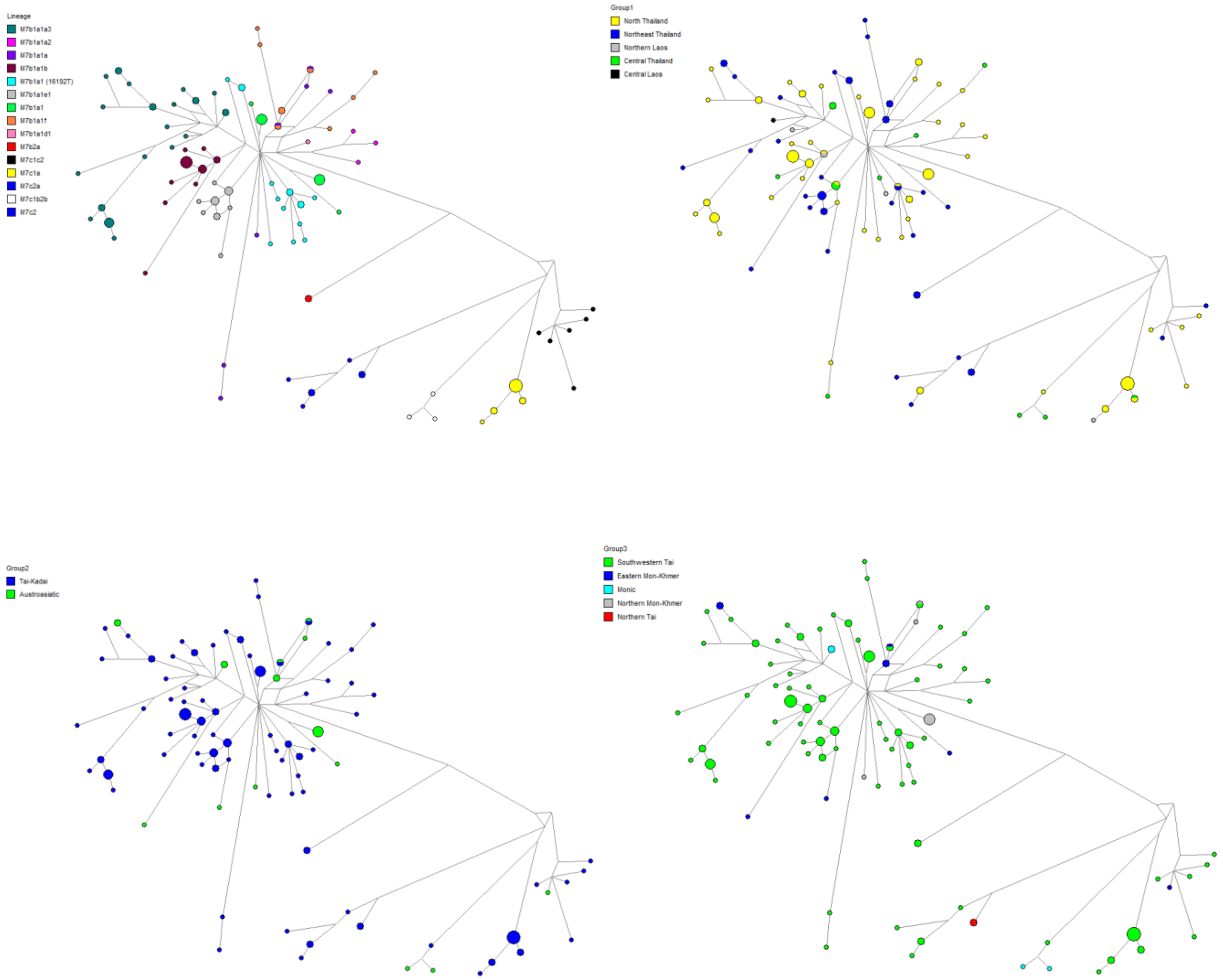
Lineage
M7b1a1a3
M7b1a1a2
M7b1a1a
M7b1a1b
M7b1a1 (16192T)
M7b1a1e1
M7b1a1
M7b1a1f
M7b1a1d1
M7b2a
M7c1c2
M7c1a
M7c2a
M7c1b2b
M7c2
Group1
North Thailand
Northeast Thailand
Northern Laos
Central Thailand
Central Laos
Group2
Tai-Kadai
Austroasiatic
Group3
Southwestern Tai
Eastern Mon-Khmer
Monic
Northern Mon-Khmer
Northern Tai

D

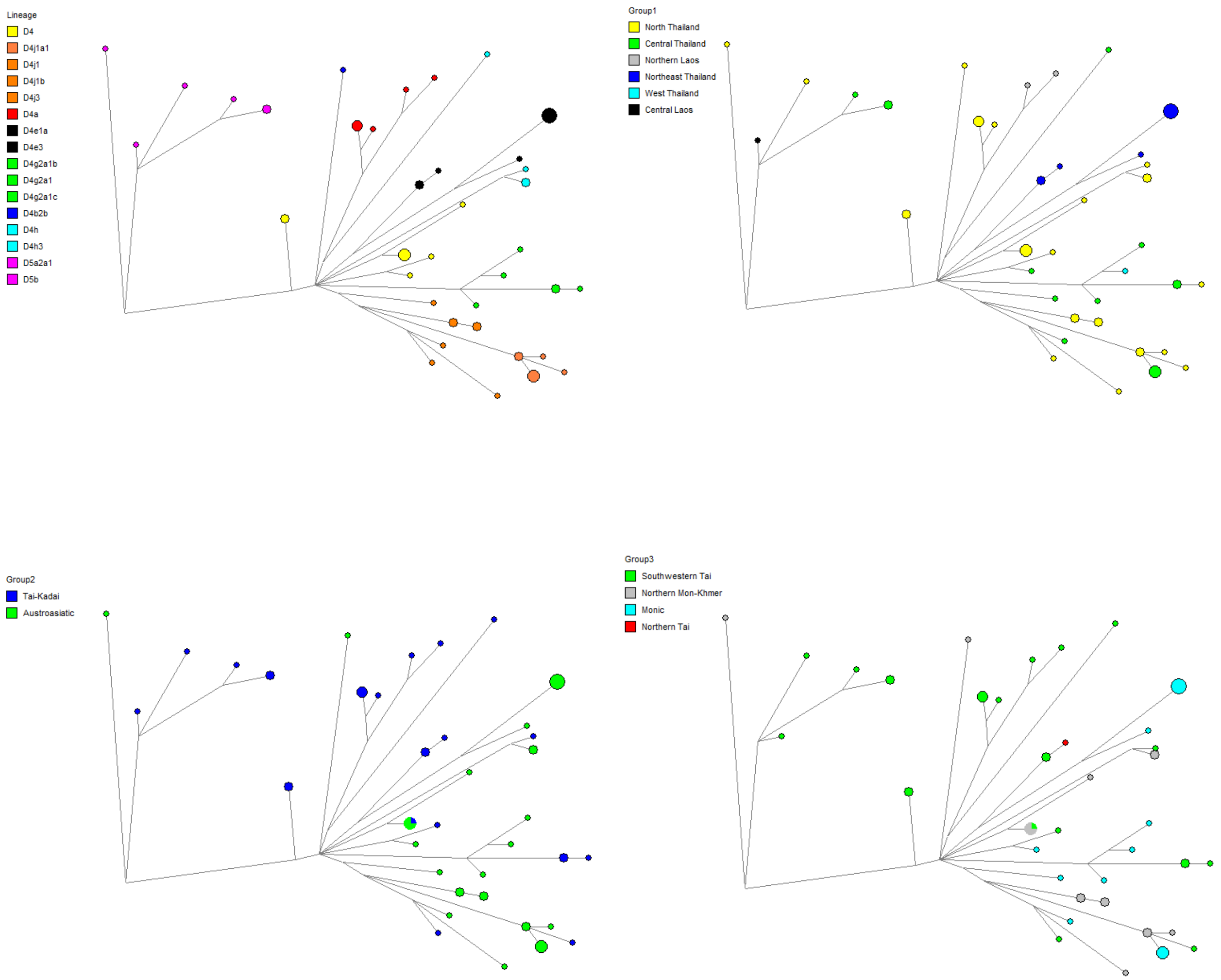
Lineage
D4
D4j1a1
D4j1
D4j1b
D4j3
D4a
D4e1a
D4e3
D4g2a1b
D4g2a1
D4g2a1c
D4b2b
D4h
D4h3
D5a2a1
D5b
Group1
North Thailand
Central Thailand
Northern Laos
Northeast Thailand
West Thailand
Central Laos
Group2
Tai-Kadai
Austroasiatic
Group3
Southwestern Tai
Northern Mon-Khmer
Monic
Northern Tai

M8

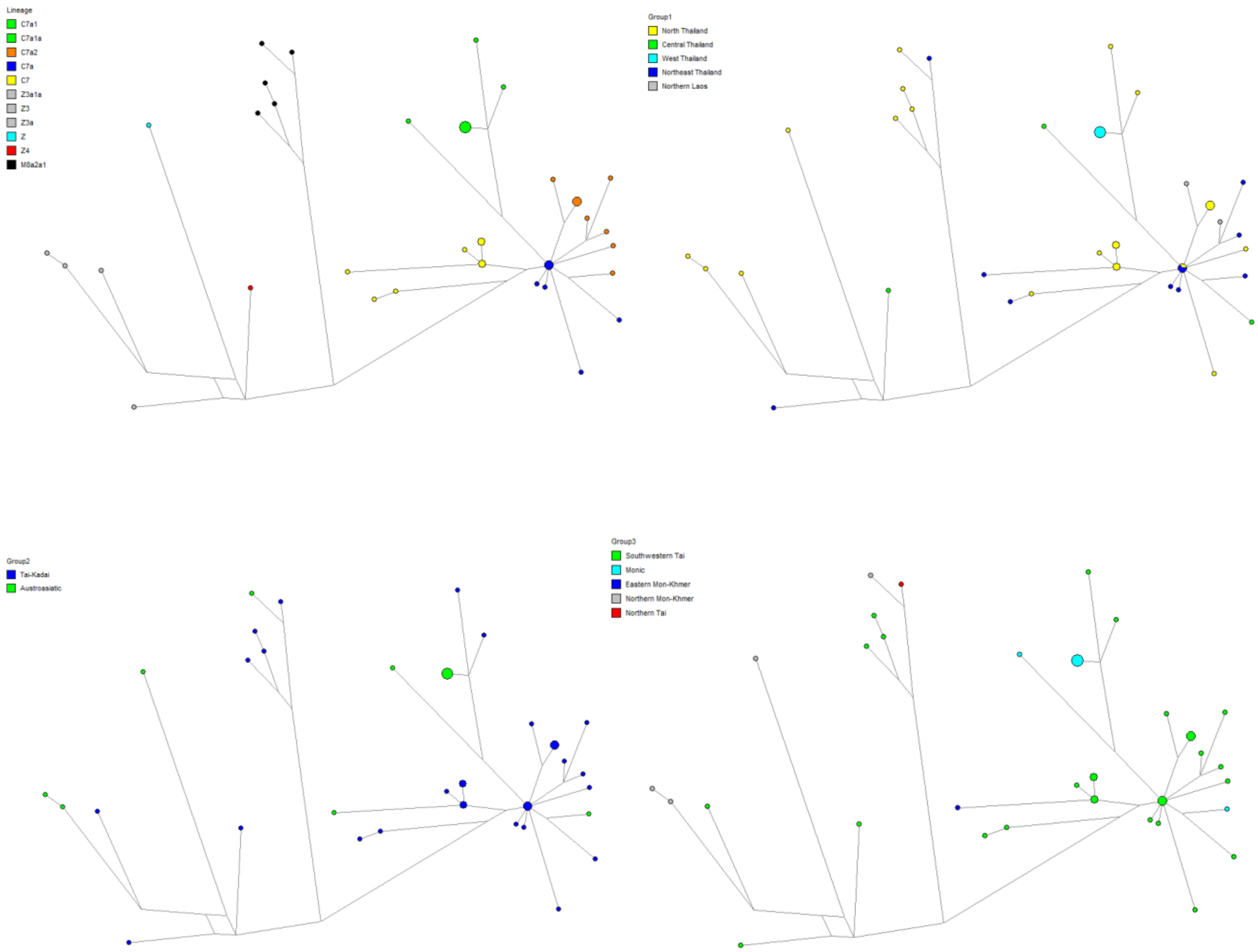
Lineage
C7a1
C7a1a
C7a2
C7a
C7
Z3a1a
Z3
Z3a
Z
Z4
M8a2a1
Group1
North Thailand
Central Thailand
West Thailand
Northeast Thailand
Northern Laos
Group2
Tai-Kadai
Austroasiatic
Group3
Southwestern Tai
Monic
Eastern Mon-Khmer
Northern Mon-Khmer
Northern Tai

M12-G

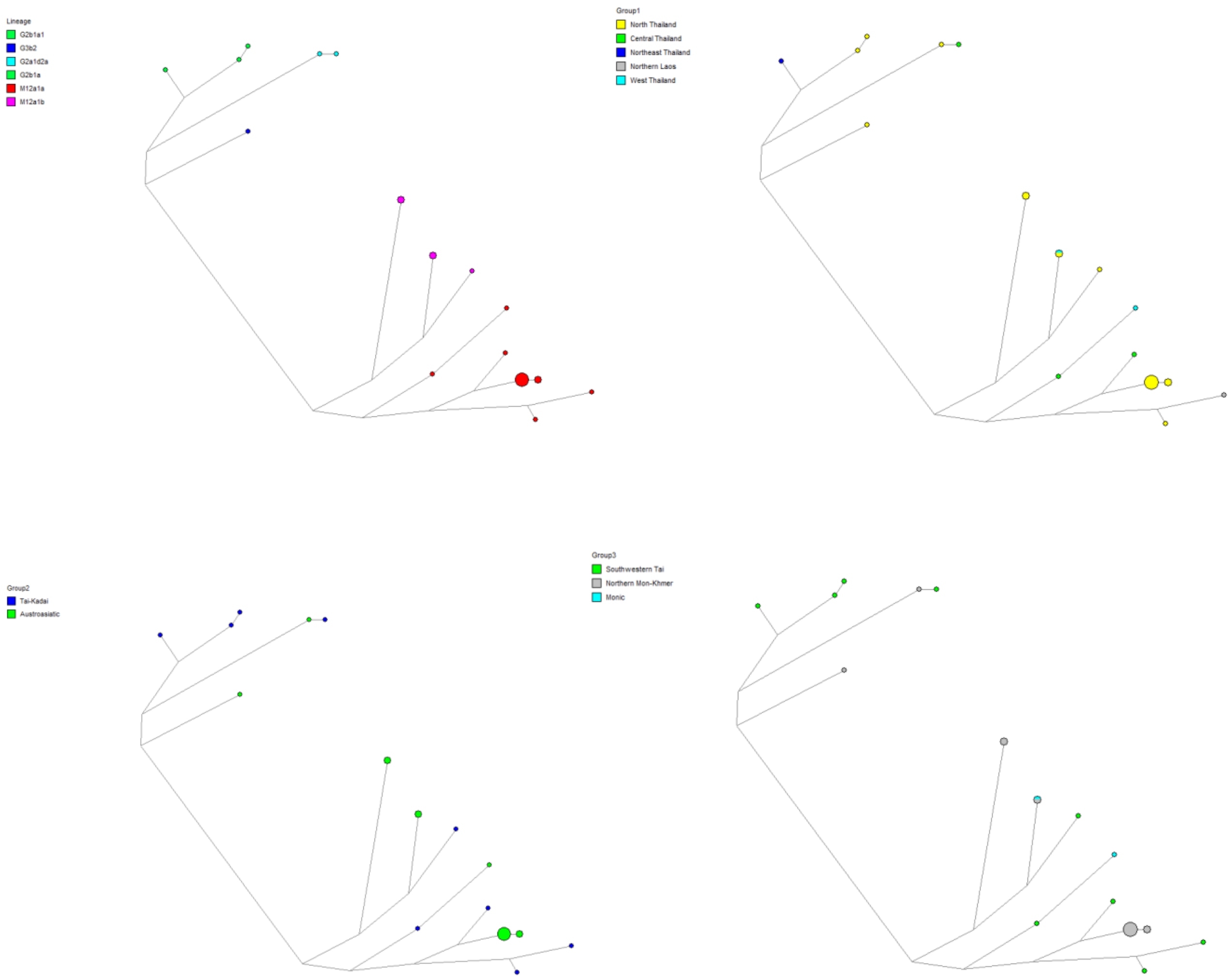
Lineage
G2b1a1
G3b2
G2a1d2a
G2b1a
M12a1a
M12a1b
Group1
North Thailand
Central Thailand
Northeast Thailand
Northern Laos
West Thailand
Group2
Tai-Kadai
Austroasiatic
Group3
Southwestern Tai
Northern Mon-Khmer
Monic

M20

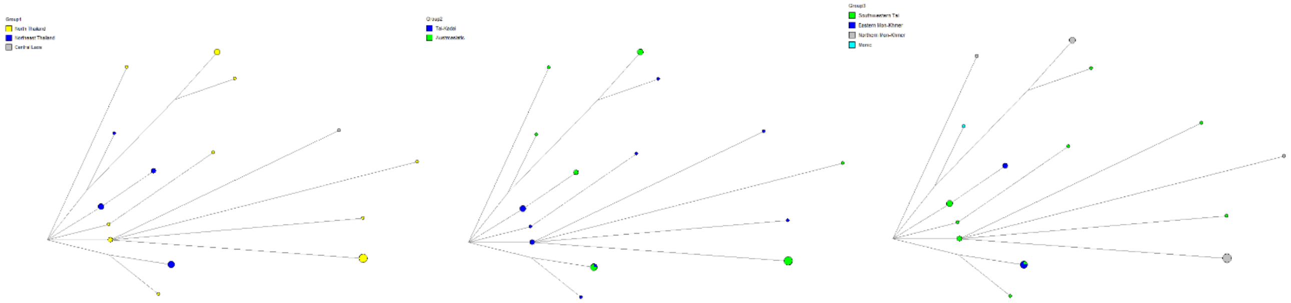
Group1
North Thailand
Northeast Thailand
Central Laos
Group2
Tai-Kadai
Austroasiatic
Group3
Southwestern Tai
Eastern Mon-Khmer
Northern Mon-Khmer
Monic

M24

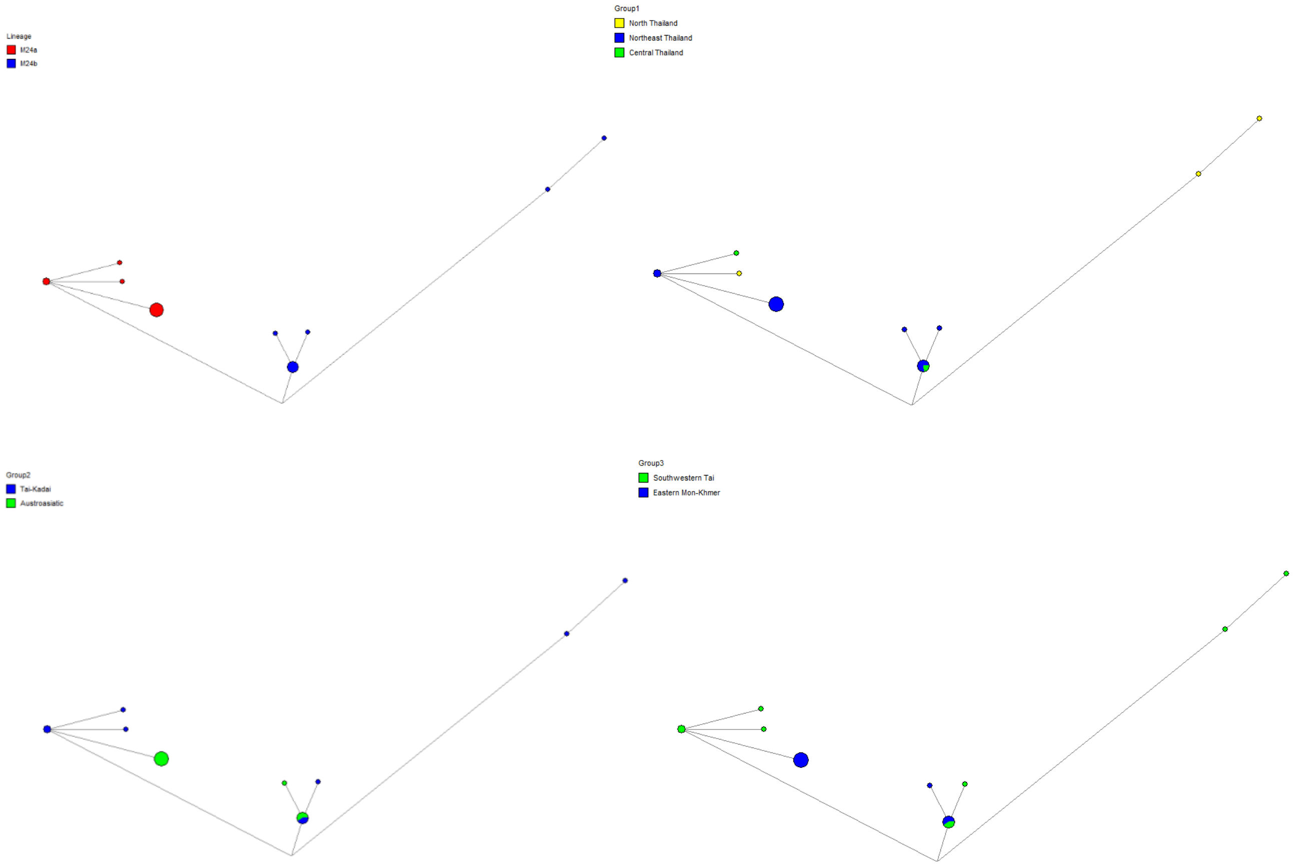
Lineage
M24a
M24b
Group1
North Thailand
Northeast Thailand
Central Thailand
Group2
Tai-Kadai
Austroasiatic
Group3
Southwestern Tai
Eastern Mon-Khmer

M74

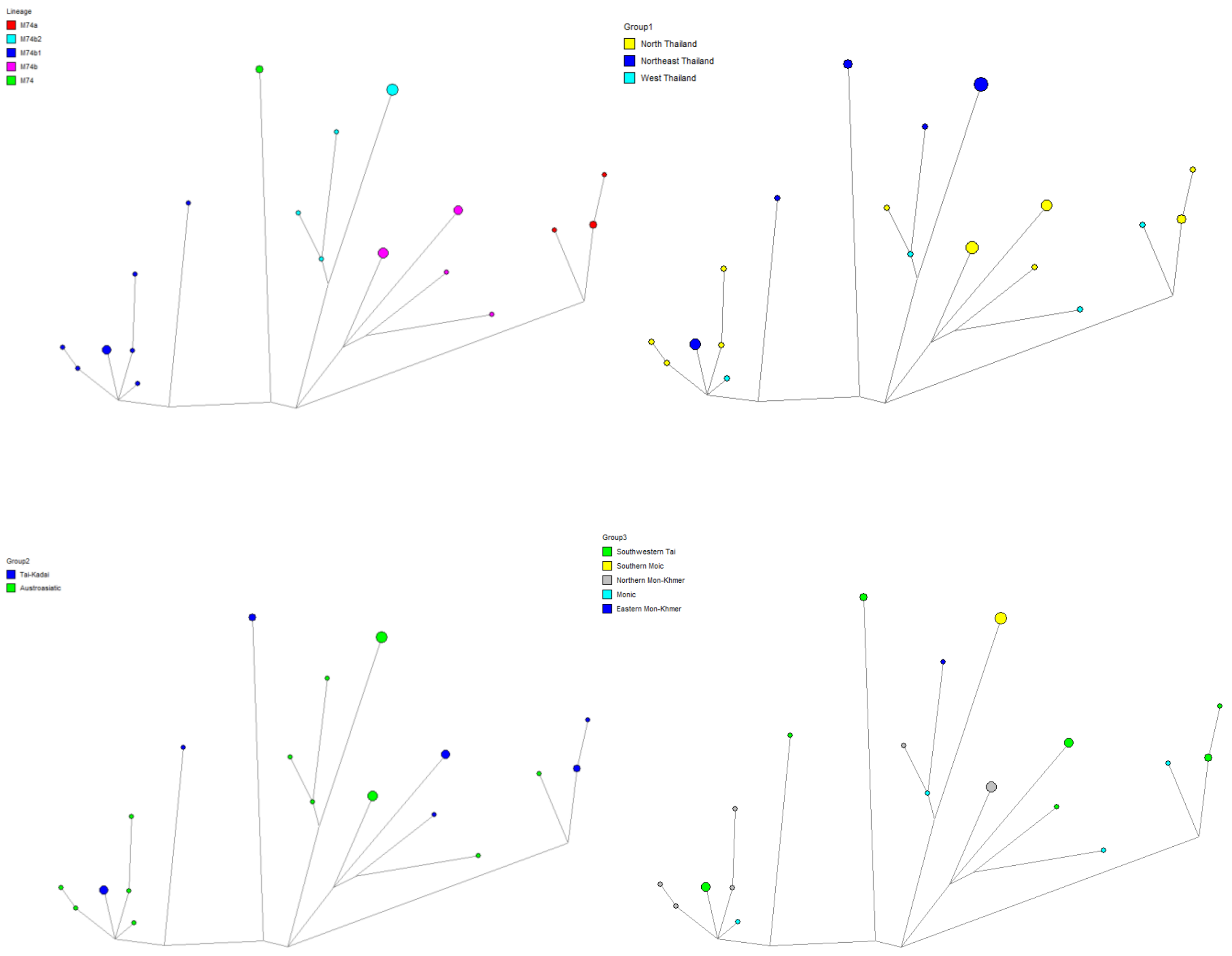
Lineage
M74a
M74b2
M74b1
M74b
M74
Group1
North Thailand
Northeast Thailand
West Thailand
Group2
Tai-Kadai
Austroasiatic
Group3
Southwestern Tai
Southern Moic
Northern Mon-Khmer
Monic
Eastern Mon-Khmer

R9b

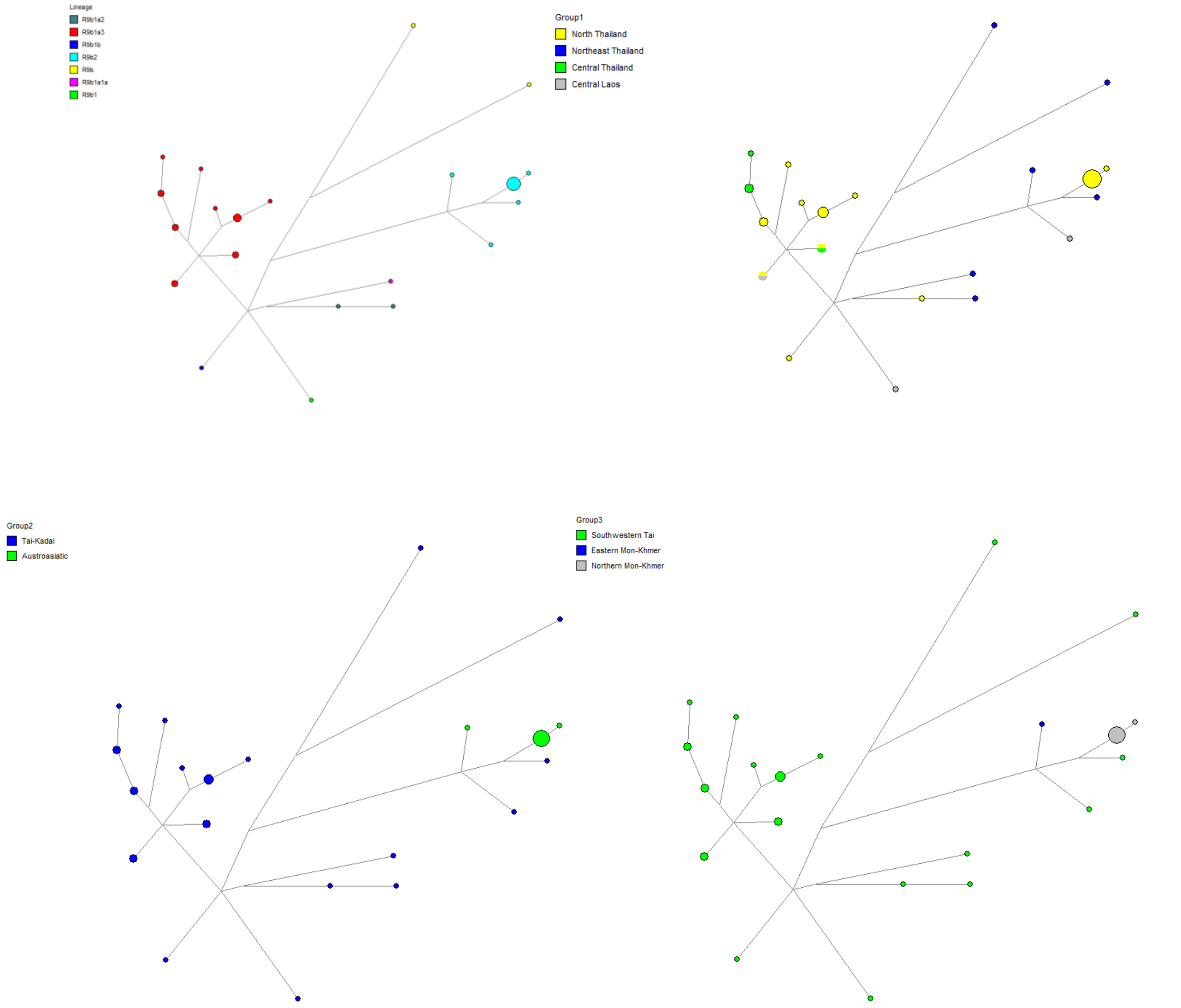
Lineage
R9b1a2
R9b1a3
R9b1b
R9b2
R9b
R9b1a1a
R9b1
Group1
North Thailand
Northeast Thailand
Central Thailand
Central Laos
Group2
Tai-Kadai
Austroasiatic
Group3
Southwestern Tai
Eastern Mon-Khmer
Northern Mon-Khmer

R22

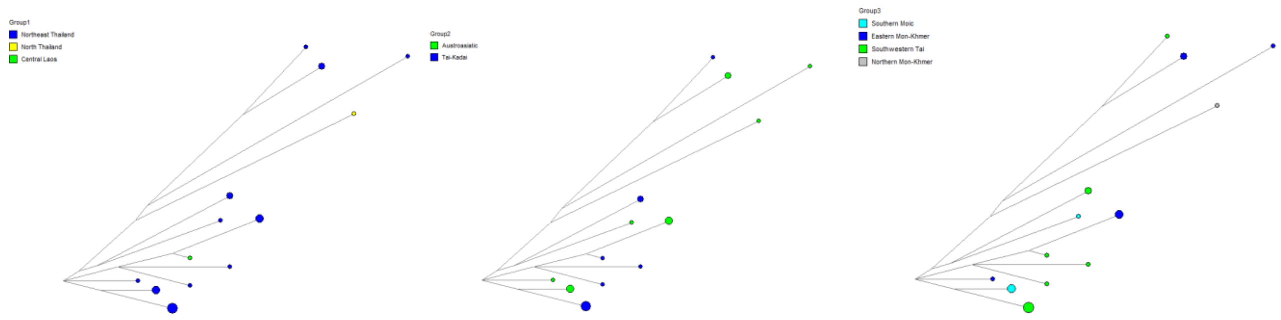
Group1
Northeast Thailand
North Thailand
Central Laos
Group2
Austroasiatic
Tai-Kadai
Group3
Southern Moic
Eastern Mon-Khmer
Southwestern Tai
Northern Mon-Khmer

N9a

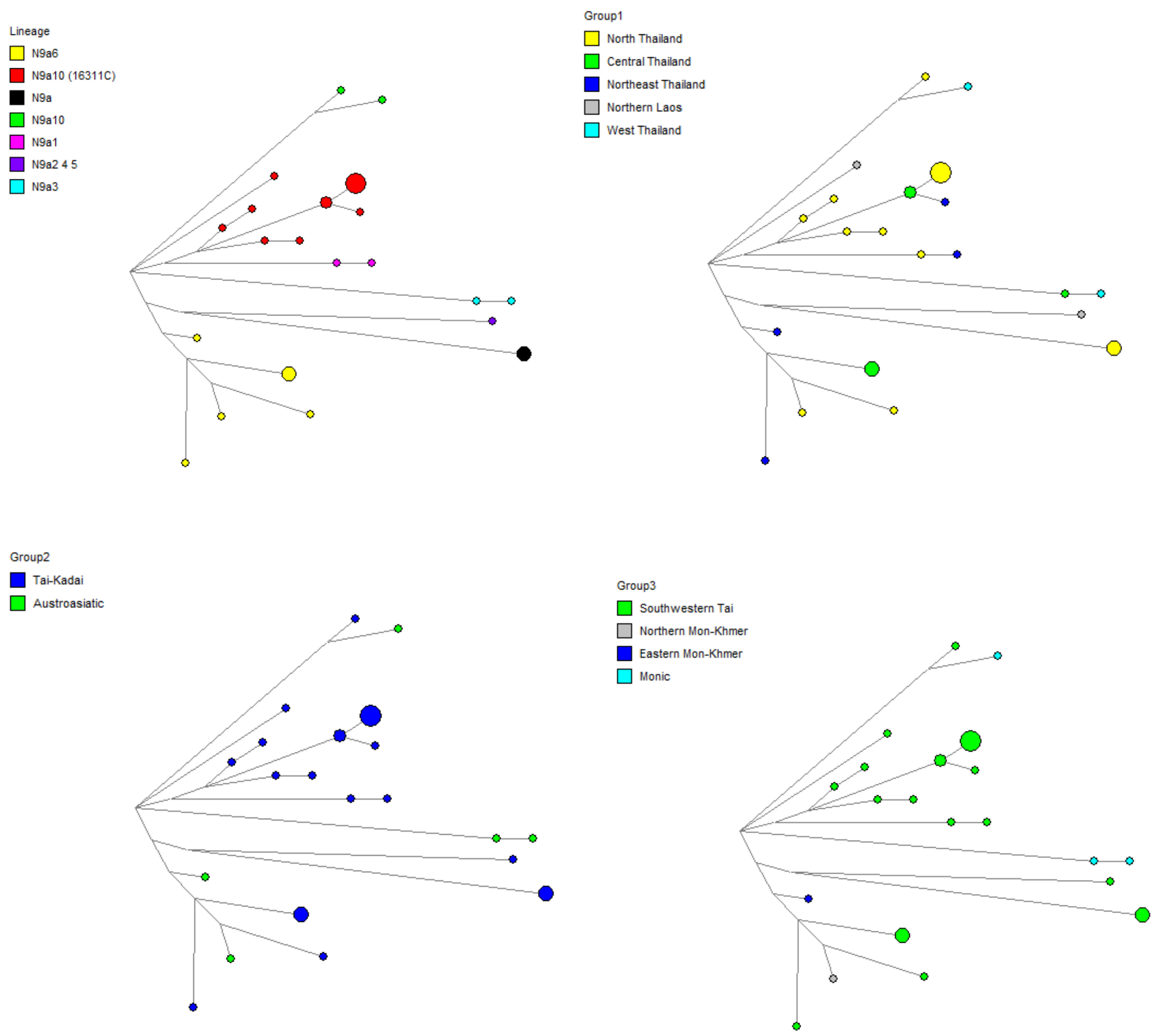
Lineage
N9a6
N9a10 (16311C)
N9a
N9a10
N9a1
N9a2 4 5
N9a3
Group1
North Thailand
Central Thailand
Northeast Thailand
Northern Laos
West Thailand
Group2
Tai-Kadai
Austroasiatic
Group3
Southwestern Tai
Northern Mon-Khmer
Eastern Mon-Khmer
Monic

B4c2

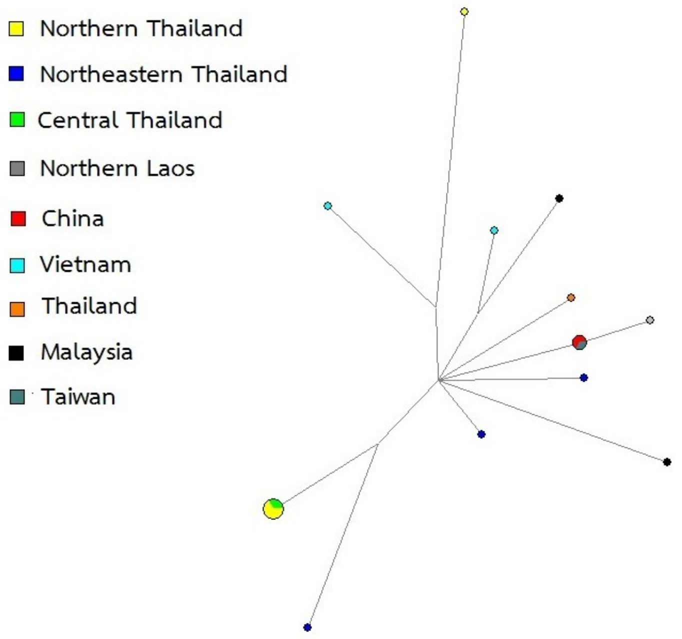
Northern Thailand
Northeastern Thailand
Central Thailand
Northern Laos
China
Vietnam
Thailand
Malaysia
Taiwan

B6a

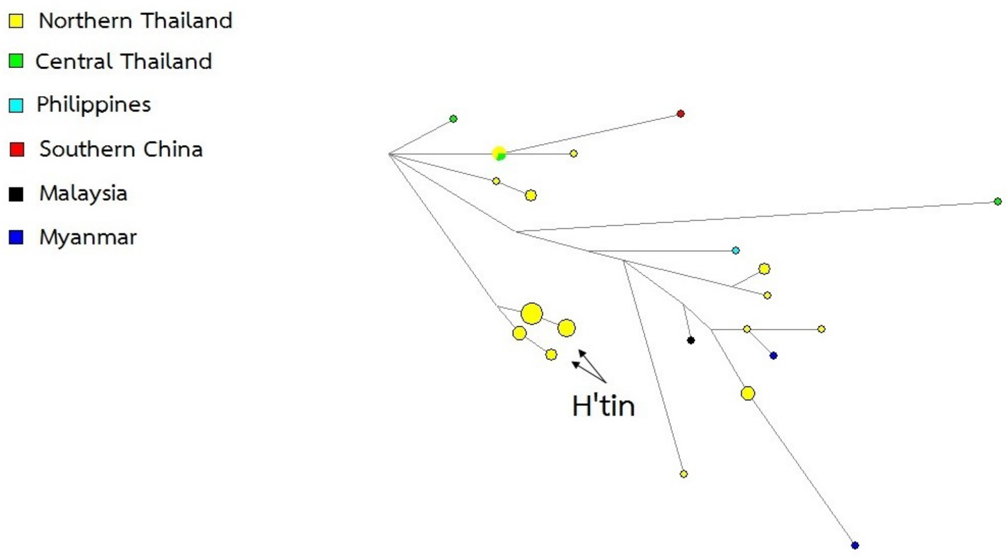
Northern Thailand
Central Thailand
Philippines
Southern China
Malaysia
Myanmar
H'tin

R22

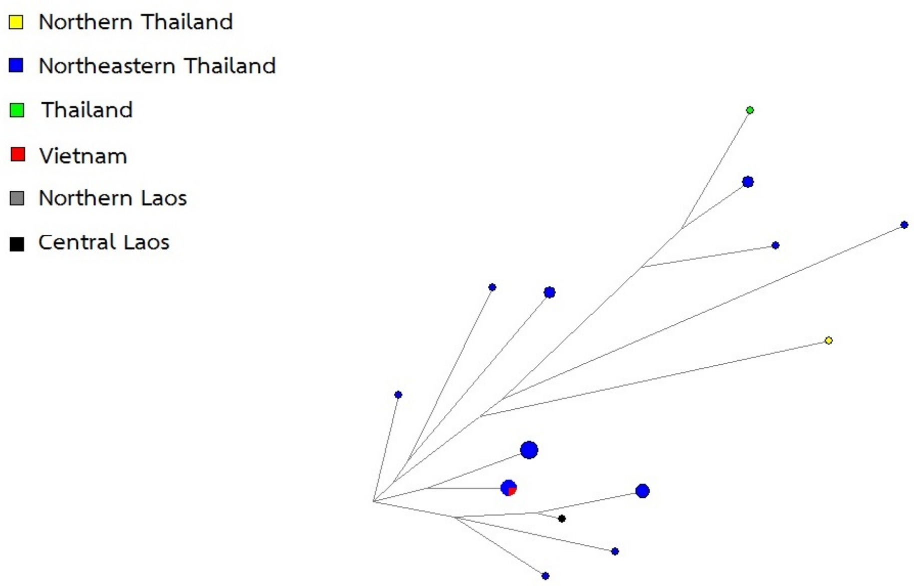

Fig. S4 Networks of 14 major haplogroups (F1a, F1f, B5a, B4, M7, D, M8, M12-G, M20, M24, M74, R9b, R22 and N9a) based on Thai/Lao mtDNA genomes and three haplogroups (B4c2, B6a and R22) based on sequences in Thai/Lao populations and other East Asian populations. Networks were generated by the program Network and post-processed by Network Publisher. The networks were colored variously according to lineages, geographic regions and languages. Geographic and linguistic classifications are as in the AMOVA analysis (Table 1).

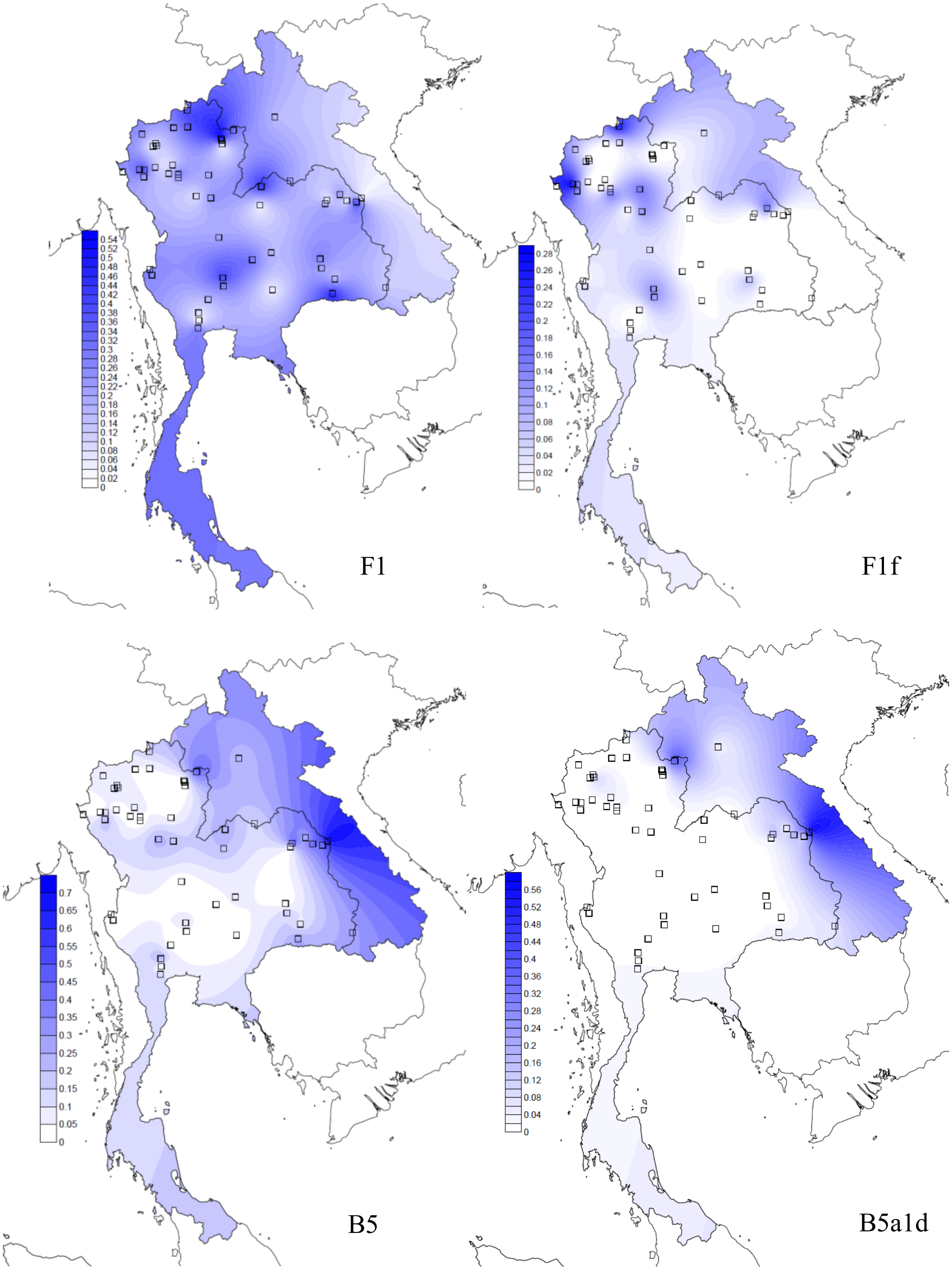
F1
F1f
B5
B5a1d

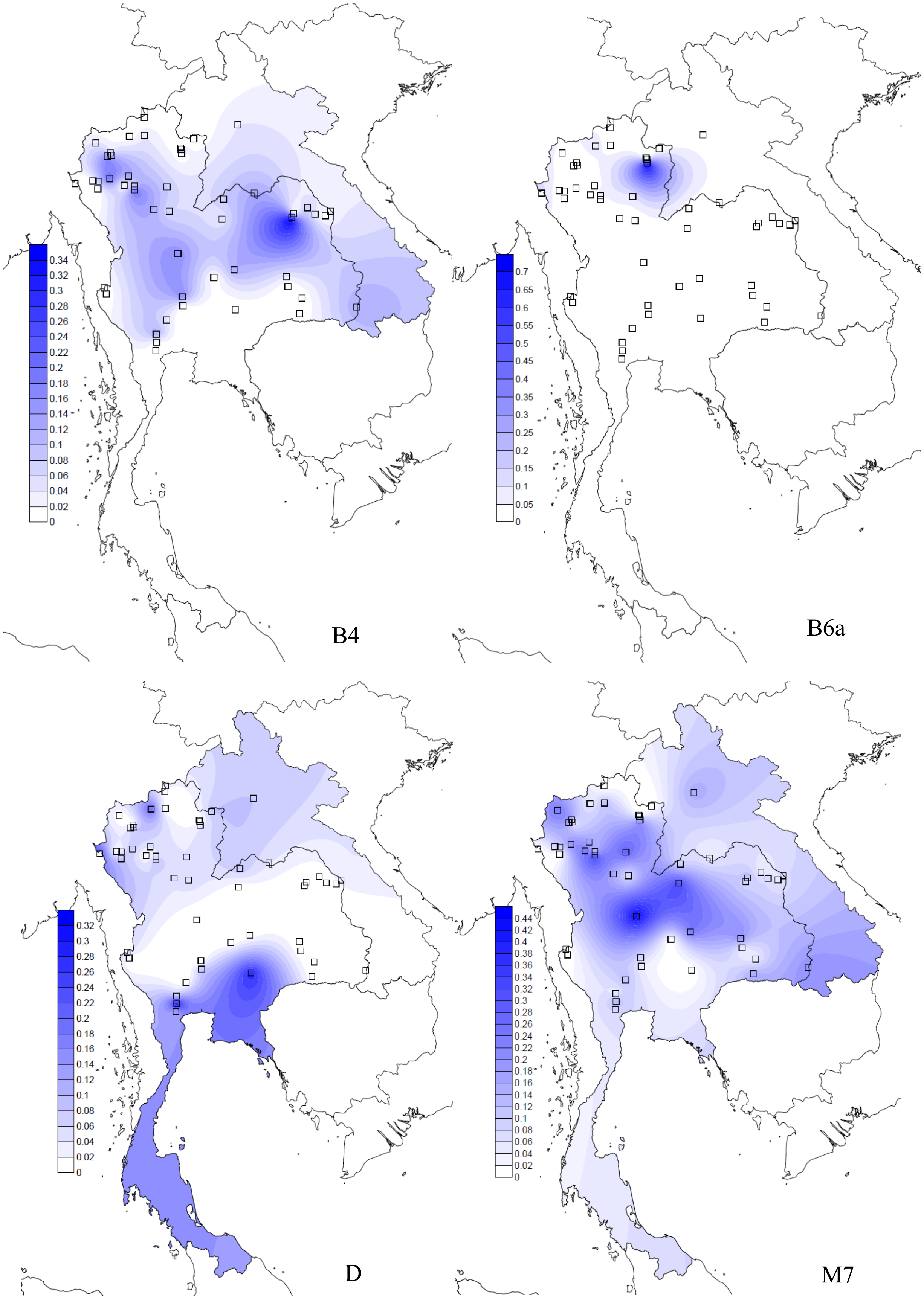

0.34
0.32
0.3
0.28
0.26
0.24
0.22
0.2
0.18
0.16
0.14
0.12
0.1
0.08
0.06
0.04
0.02
0
B4
0.7
0.65
0.6
0.55
0.5
0.45
0.4
0.35
0.3
0.25
0.2
0.15
0.1
0.05
0
B6a
0.32
0.3
0.28
0.26
0.24
0.22
0.2
0.18
0.16
0.14
0.12
0.1
0.08
0.06
0.04
0.02
0
D
0.44
0.42
0.4
0.38
0.36
0.34
0.32
0.3
0.28
0.26
0.24
0.22
0.2
0.18
0.16
0.14
0.12
0.1
0.08
0.06
0.04
0.02
0
M7

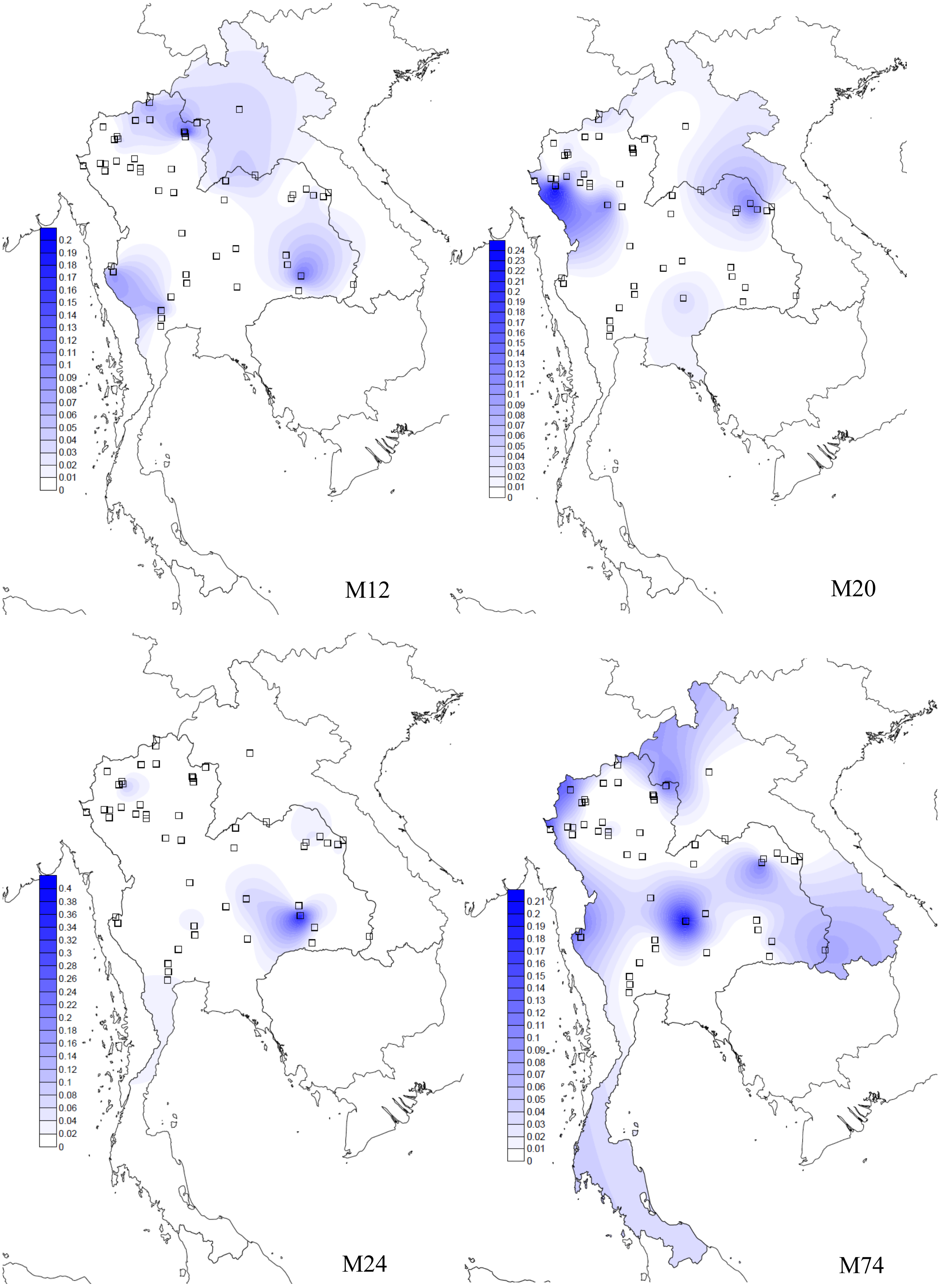
M12
0.2
0.19
0.18
0.17
0.16
0.15
0.14
0.13
0.12
0.11
0.1
0.09
0.08
0.07
0.06
0.05
0.04
0.03
0.02
0.01
0
M20
0.24
0.23
0.22
0.21
0.2
0.19
0.18
0.17
0.16
0.15
0.14
0.13
0.12
0.11
0.1
0.09
0.08
0.07
0.06
0.05
0.04
0.03
0.02
0.01
0
M24
0.4
0.38
0.36
0.34
0.32
0.3
0.28
0.26
0.24
0.22
0.2
0.18
0.16
0.14
0.12
0.1
0.08
0.06
0.04
0.02
0
M74
0.21
0.2
0.19
0.18
0.17
0.16
0.15
0.14
0.13
0.12
0.11
0.1
0.09
0.08
0.07
0.06
0.05
0.04
0.03
0.02
0.01
0

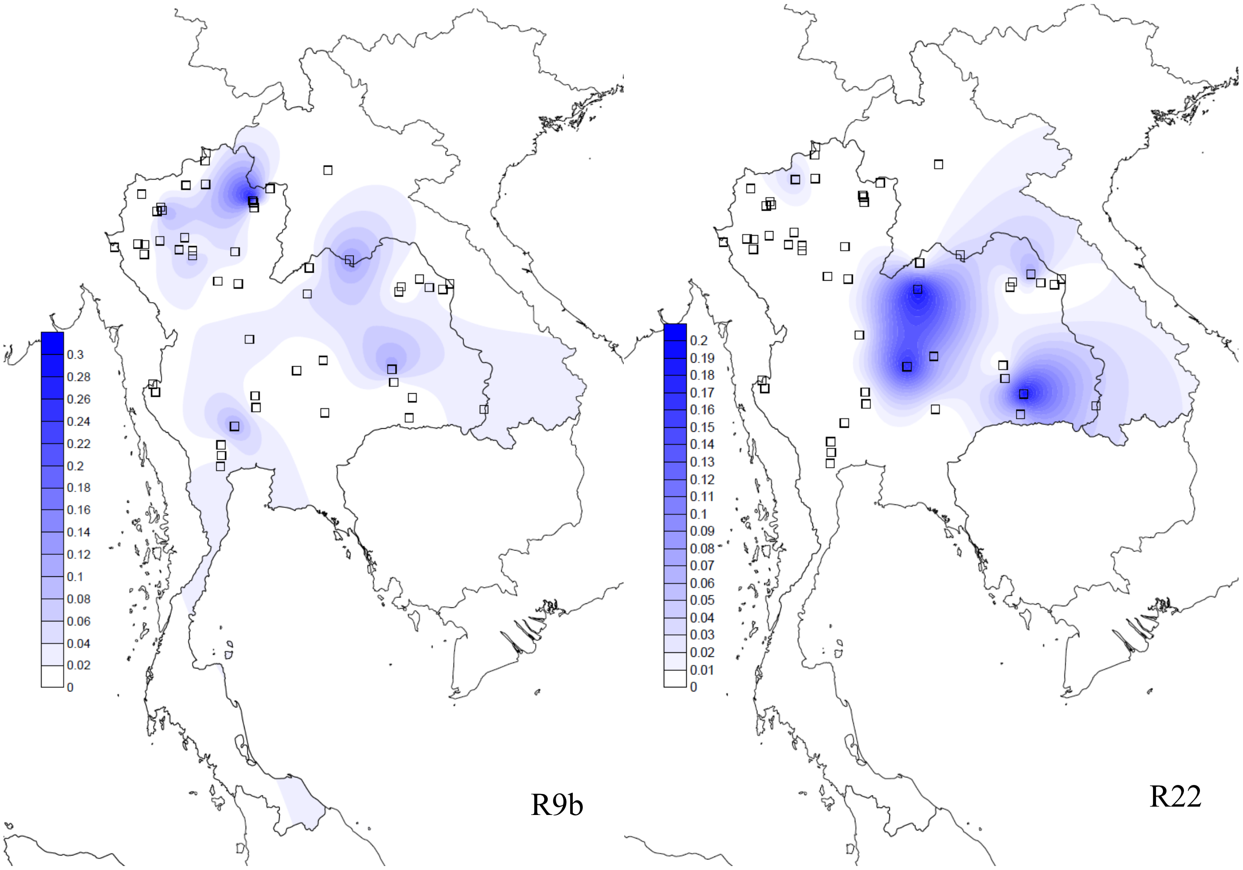

Fig. S5 The contour maps of the frequencies of 14 haplogroups (F1, F1f, B5, B5a1d, B4, B6a, D, M7, M12, M20, M24, M74, R9b and R22) in Thai/Lao populations. The maps were generated by Golden Software Surfer 10.0.

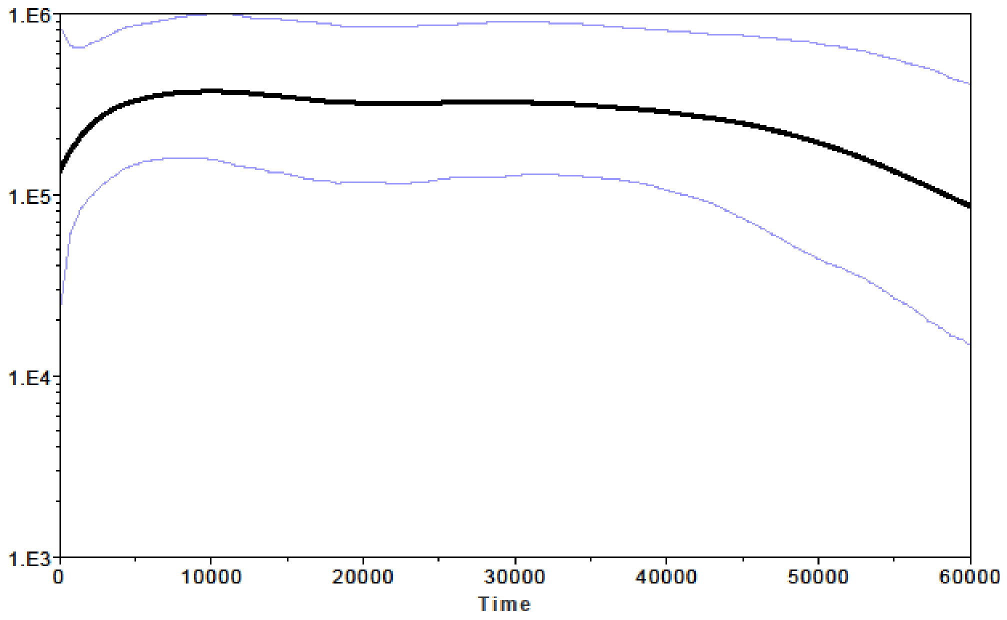

Khon Mueang1 (KM1)

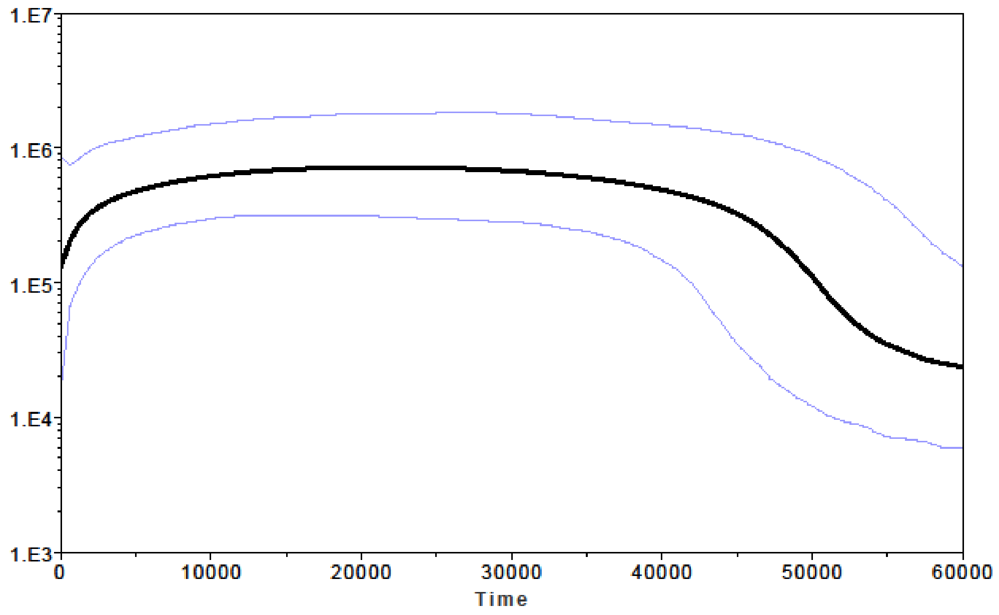

Khon Mueang2 (KM2)

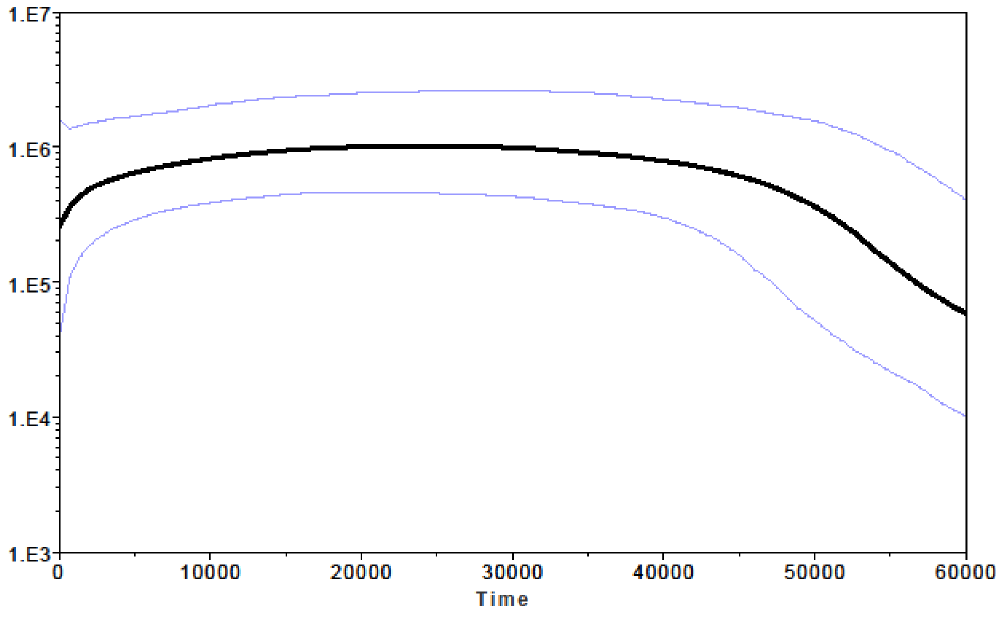

Khon Mueang3 (KM3)

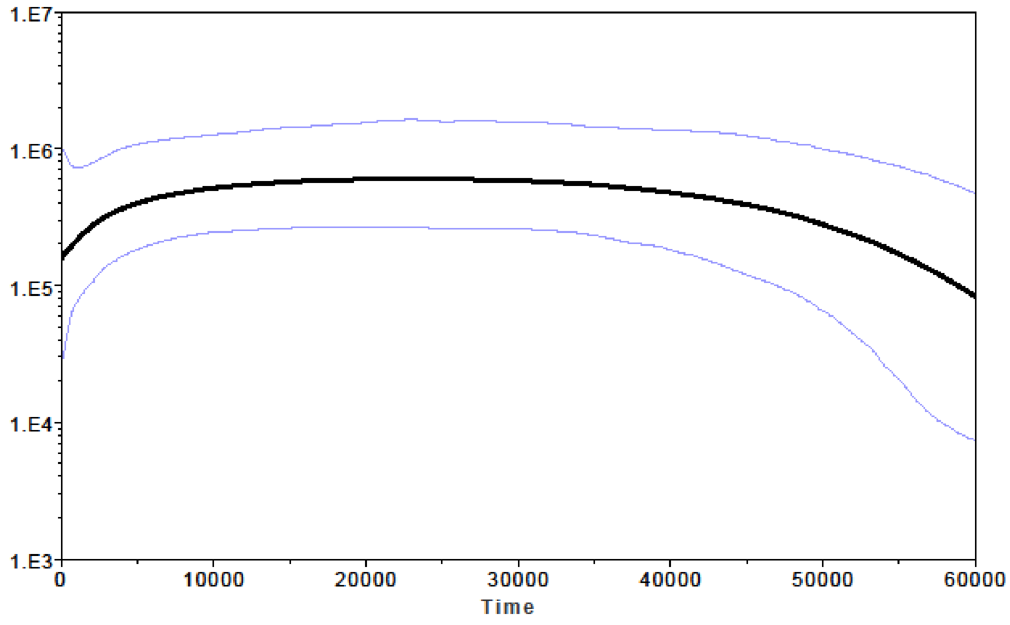

Khon Mueang4 (KM4)

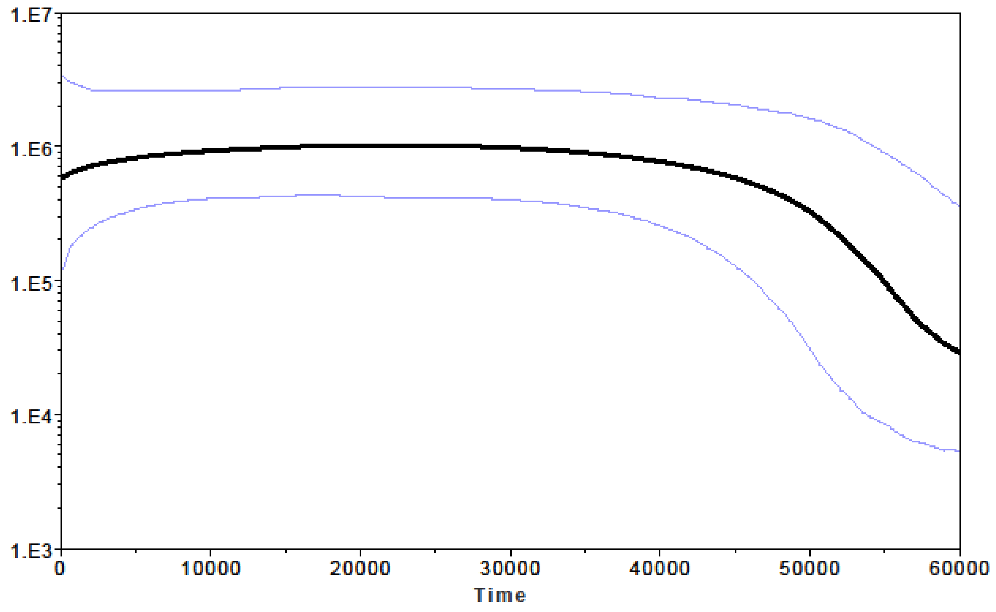

Khon Mueang5 (KM5)

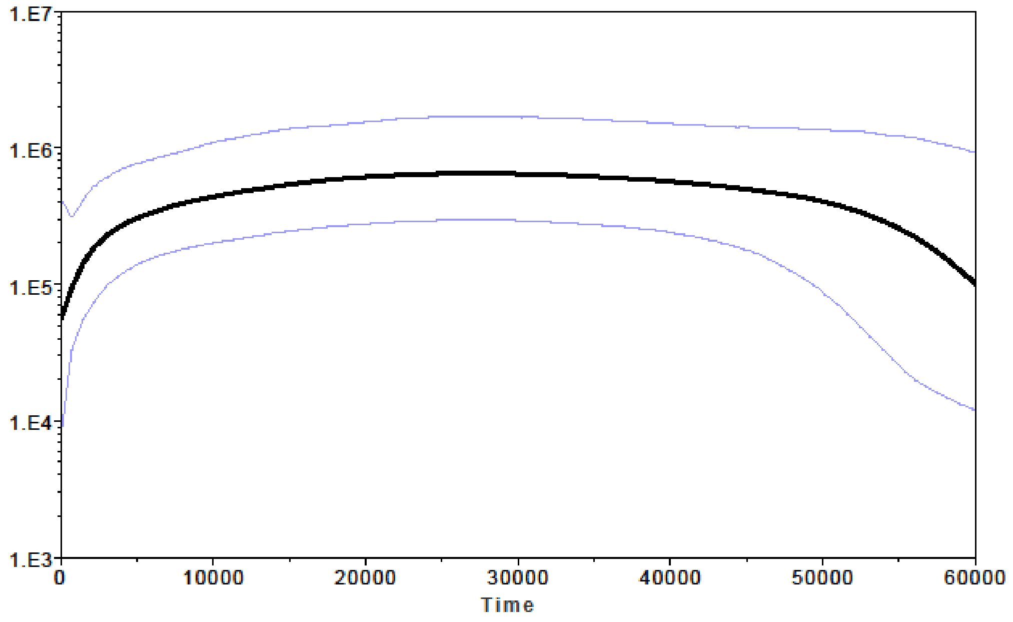

Khon Mueang6 (KM6)

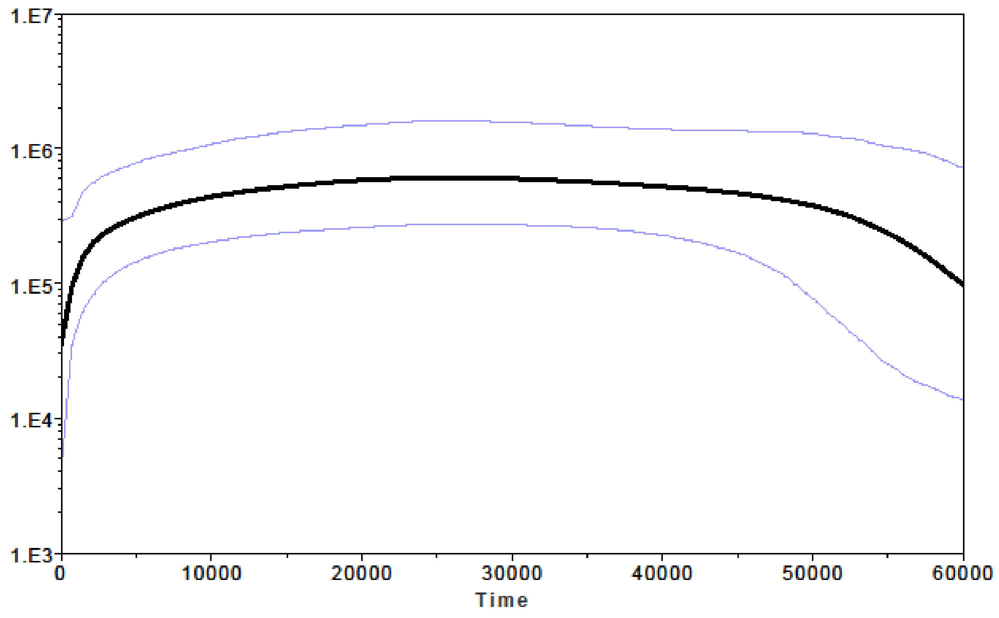

Khon Mueang7 (KM7)

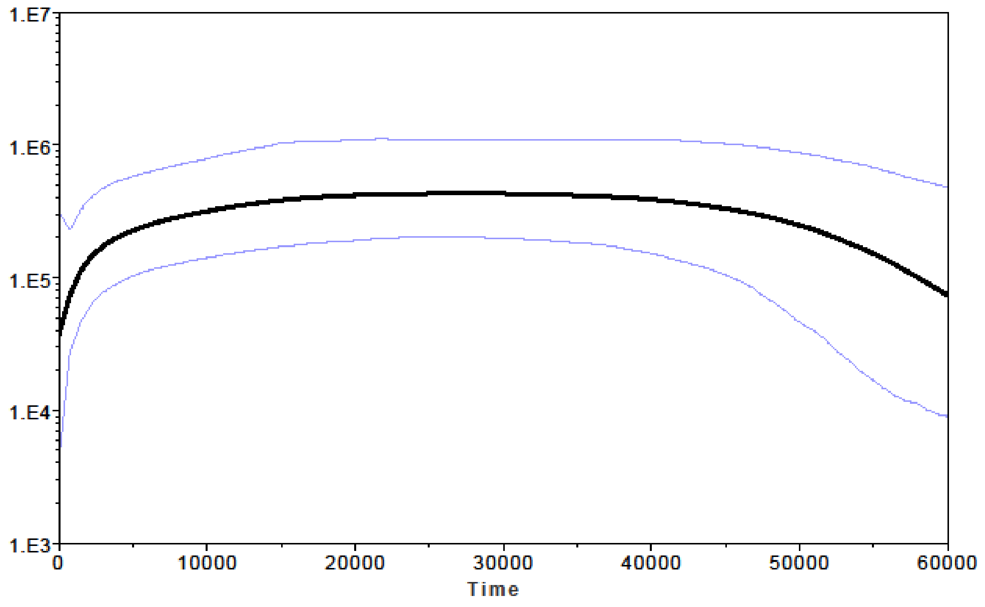

Khon Mueang8 (KM8)

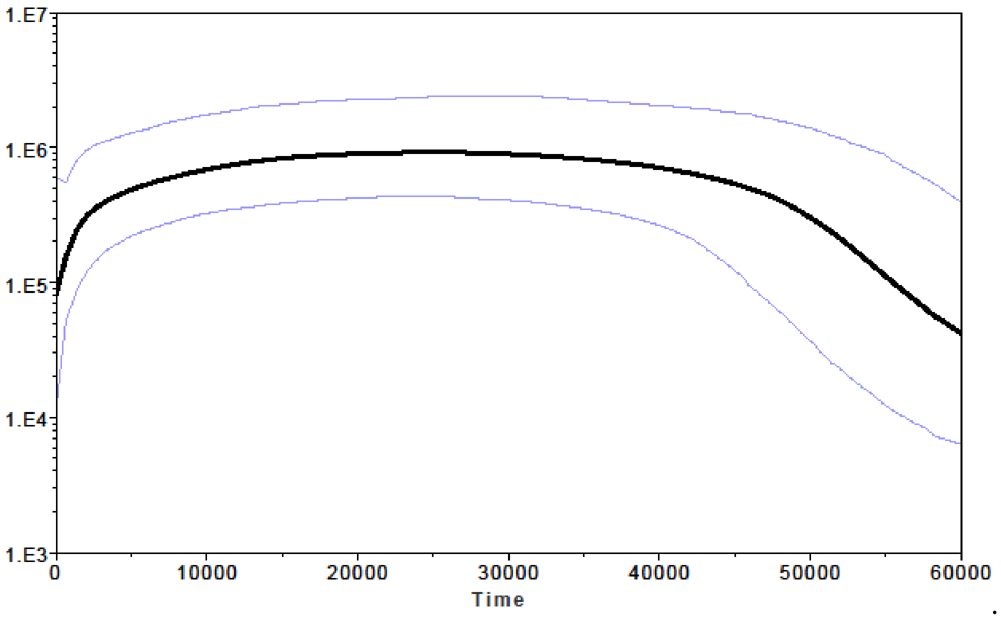

Khon Mueang9 (KM9)

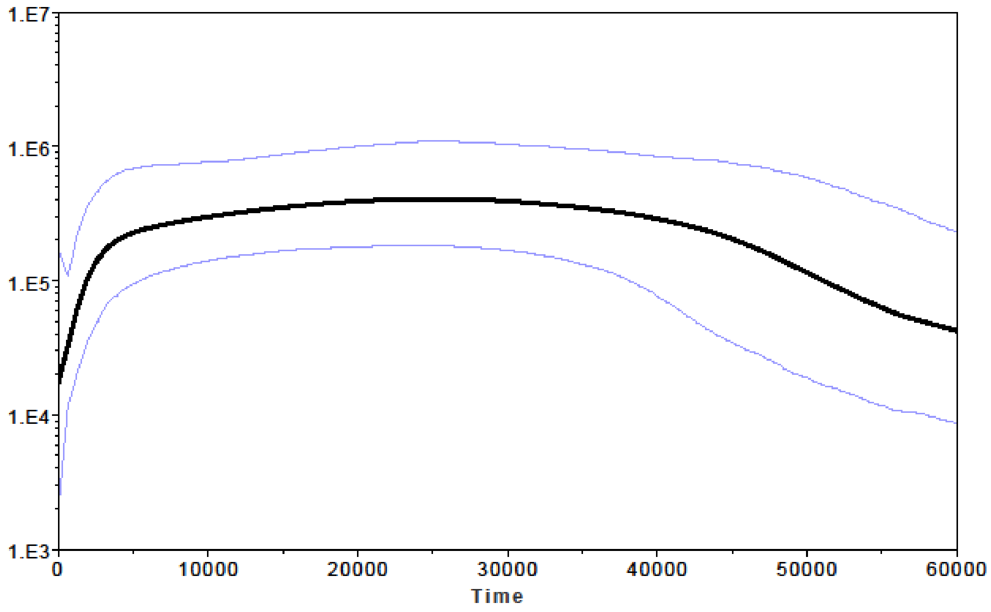

Khon Mueang10 (KM10)

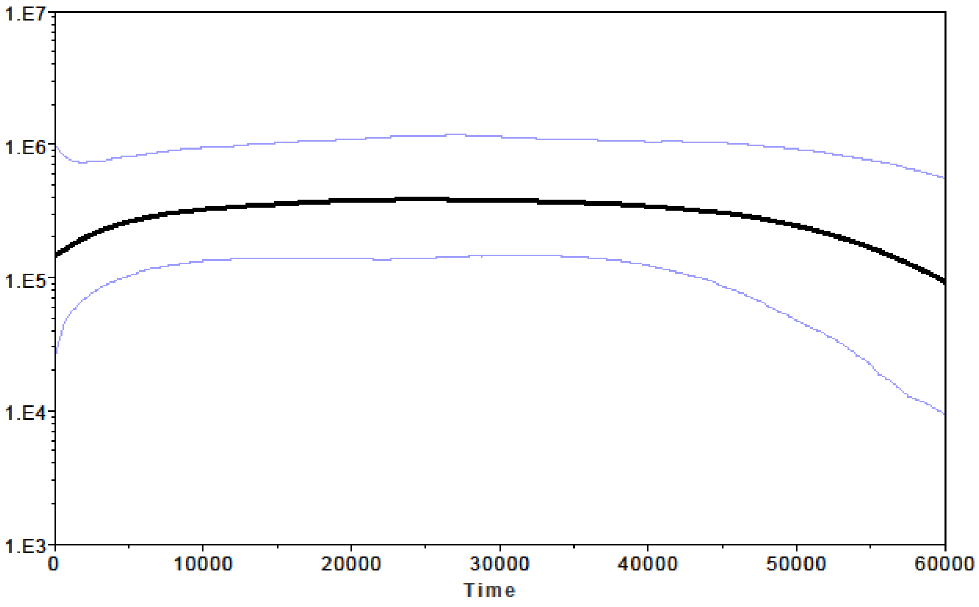

Yuan1 (YU1)

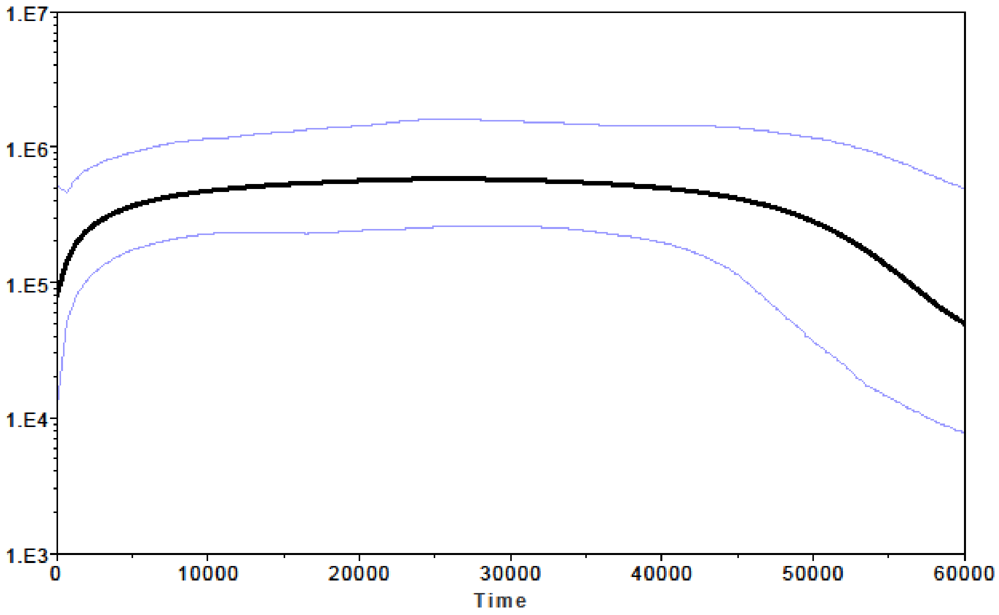

Yuan2 (YU2)

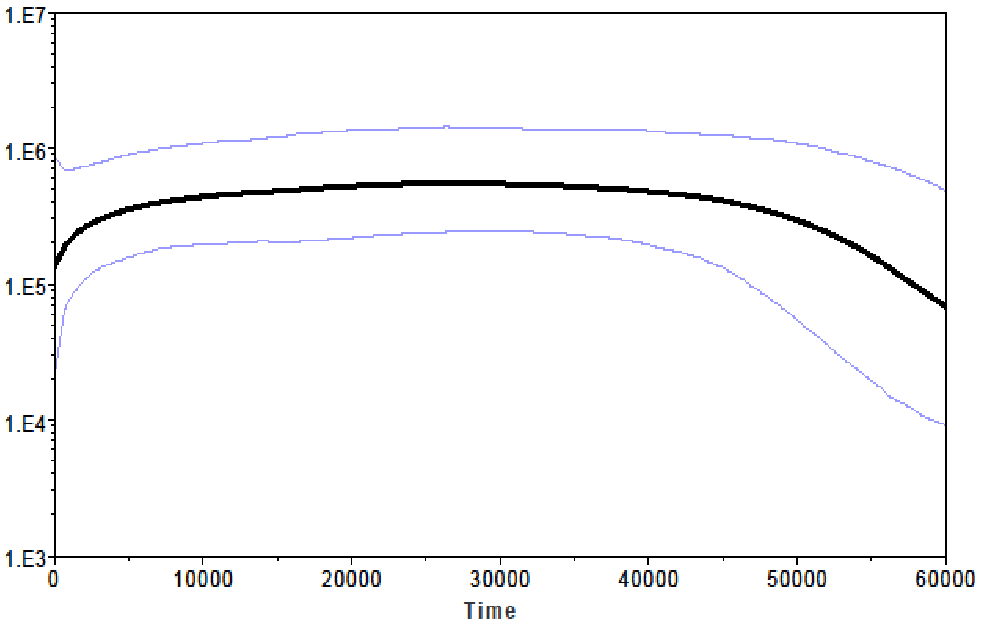

Shan (SH)

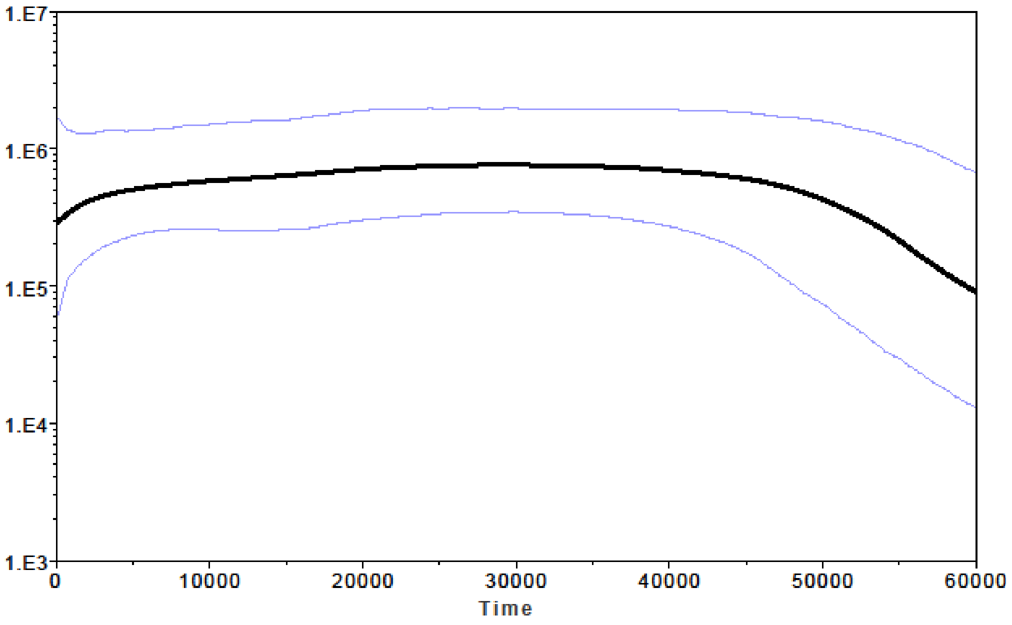

Lao Isan1 (IS1)

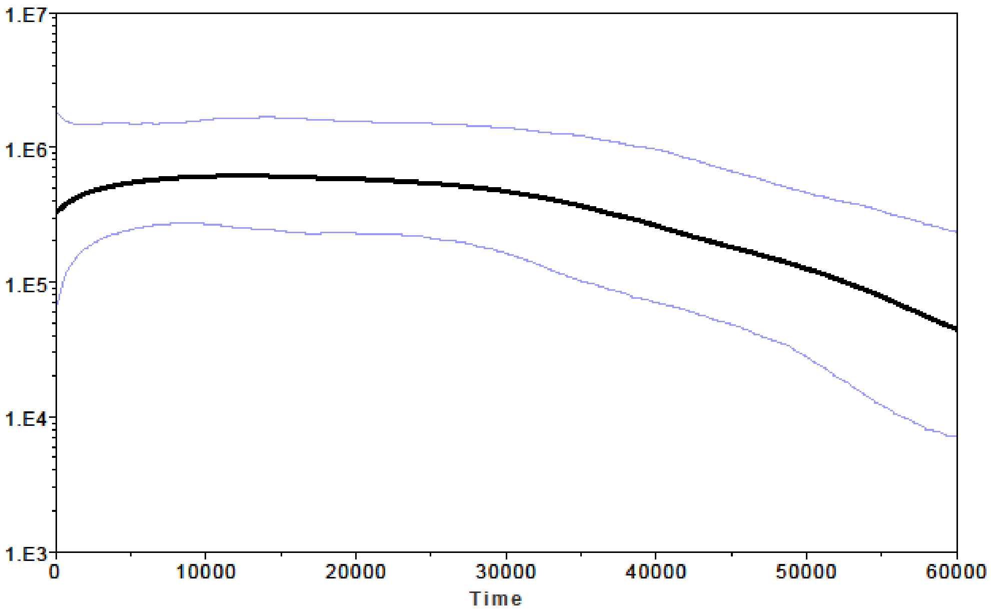

Lao Isan2 (IS2)

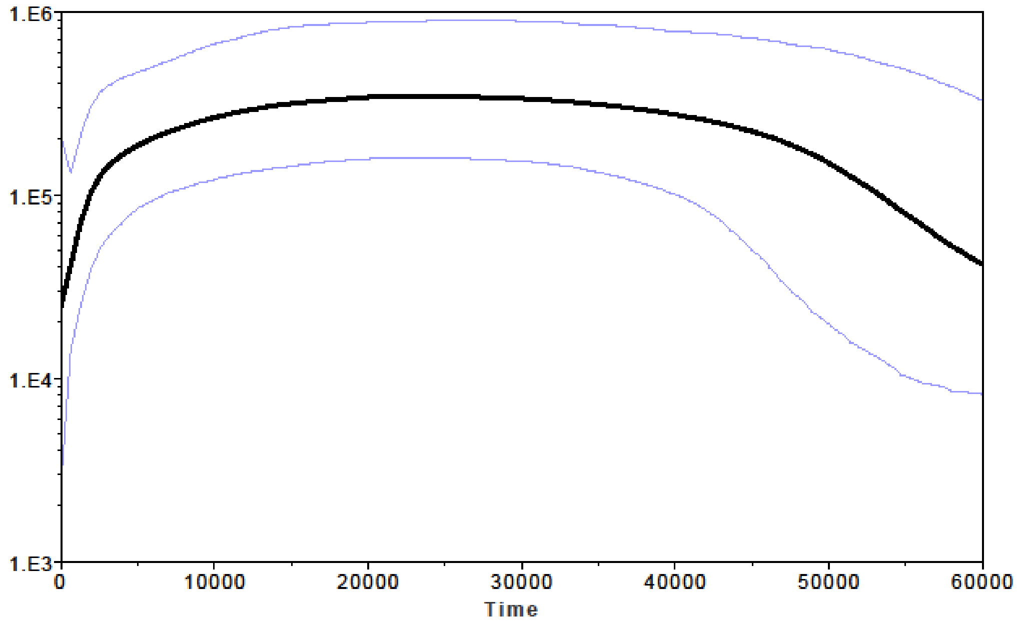

Lao Isan3 (IS3)

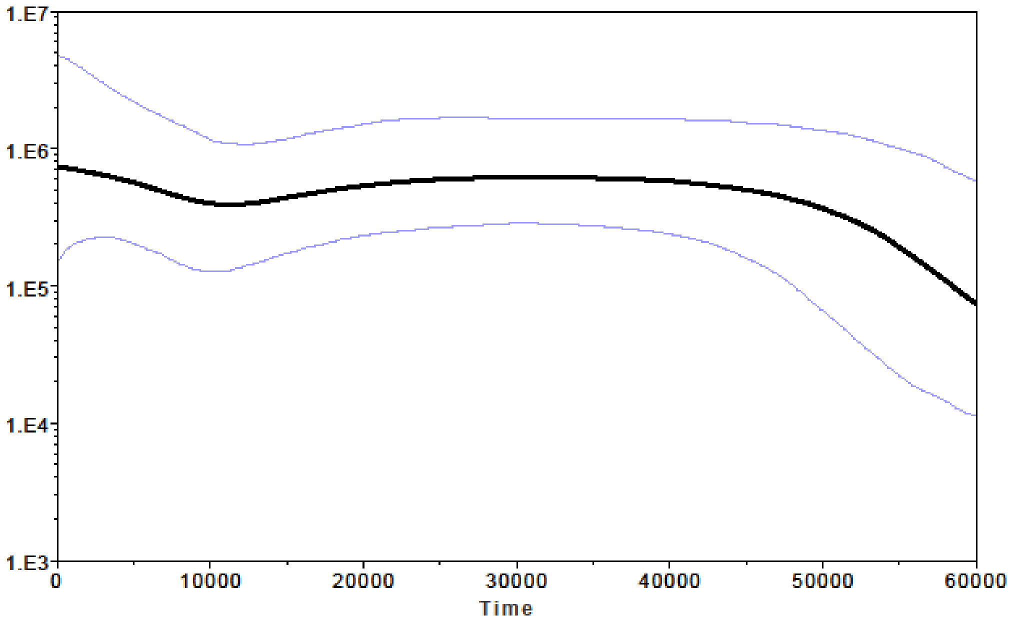

Lao Isan4 (IS4)

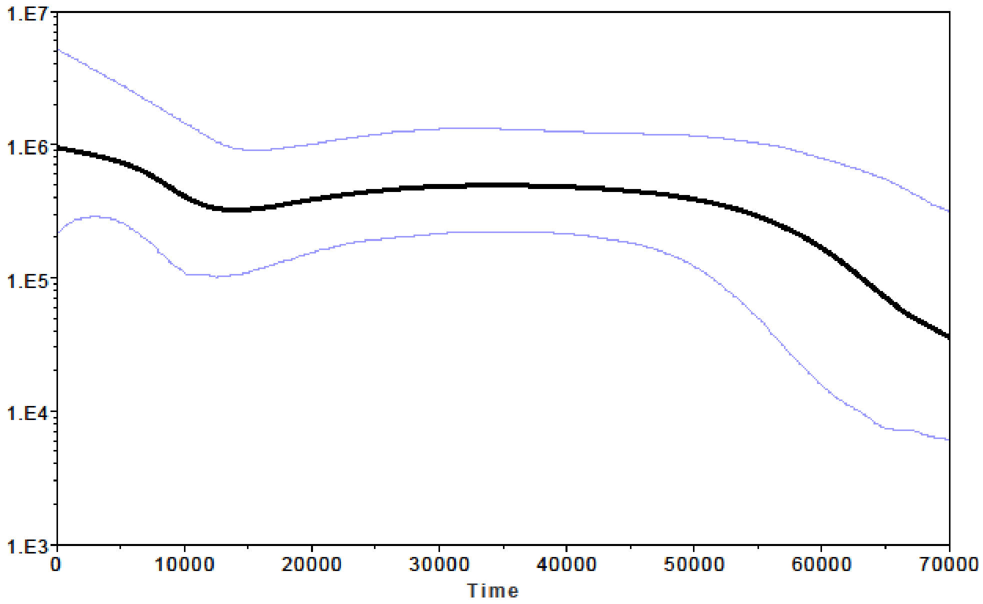

Lao Lum1 (LA1)

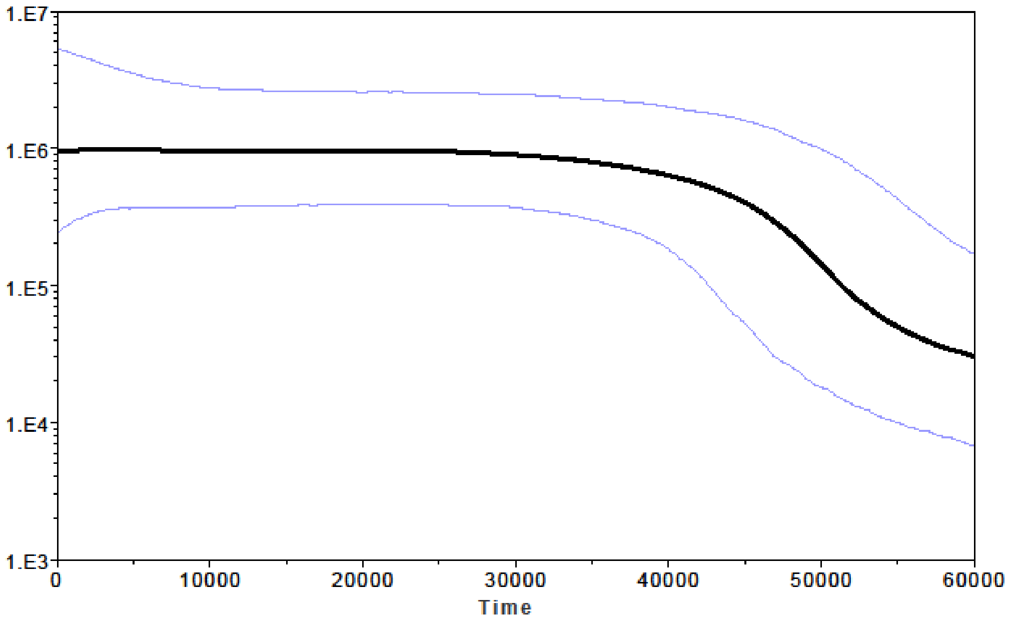

Lao Lum2 (LA2)

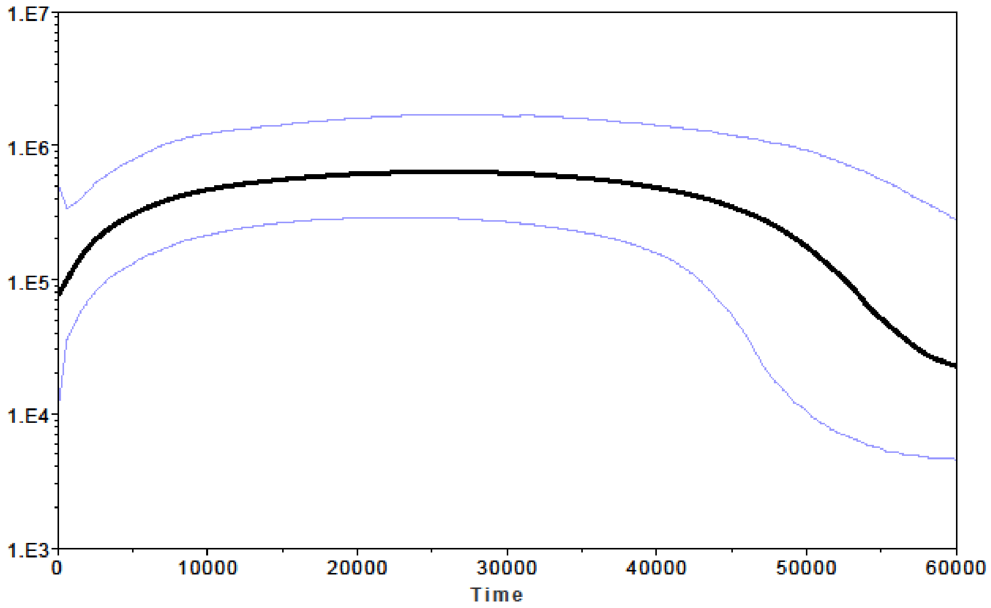

Phutai (PT)

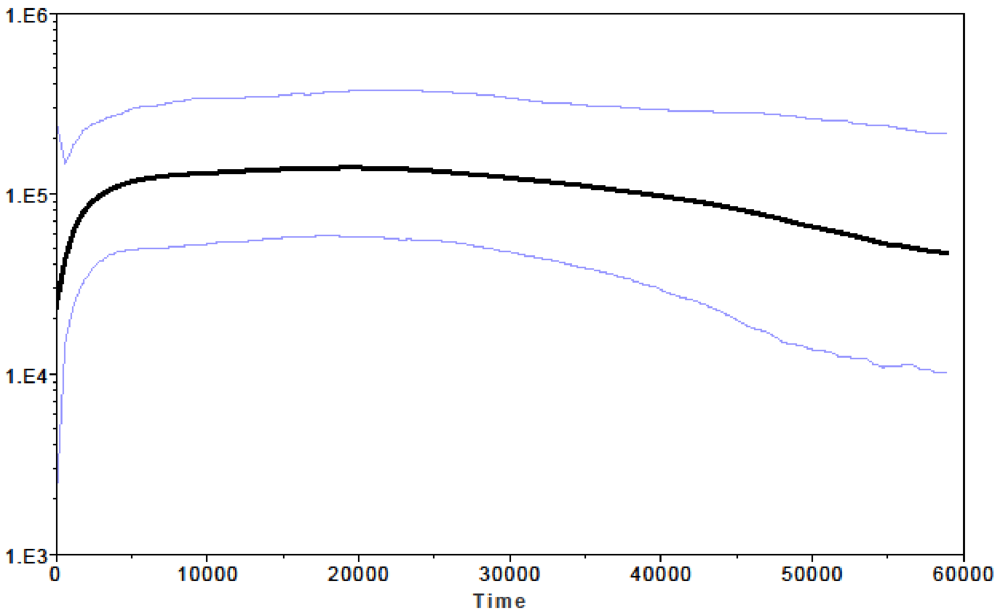

Kalueang (KL)

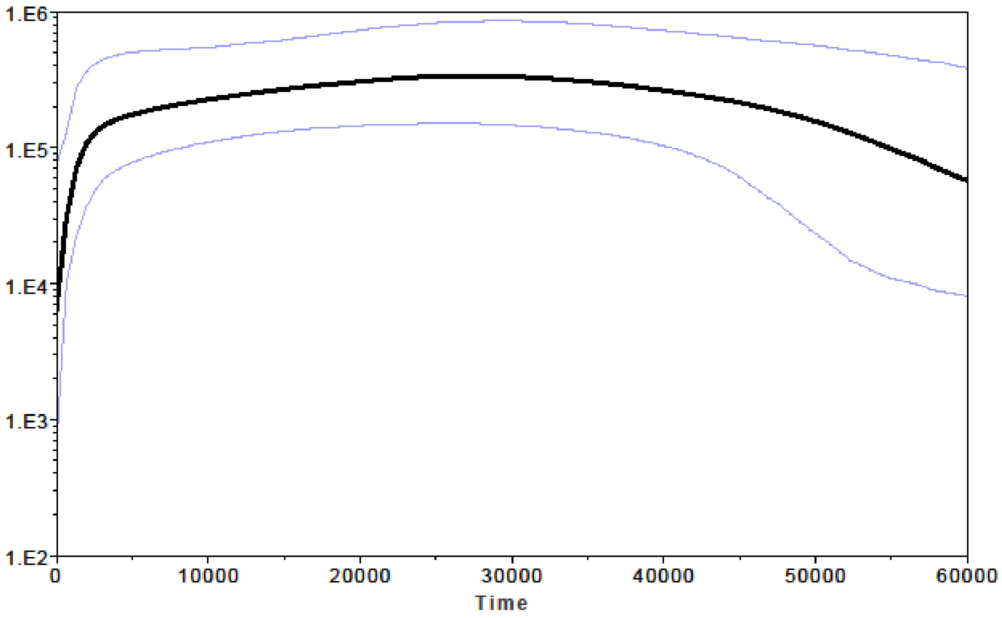

Seak (SK)

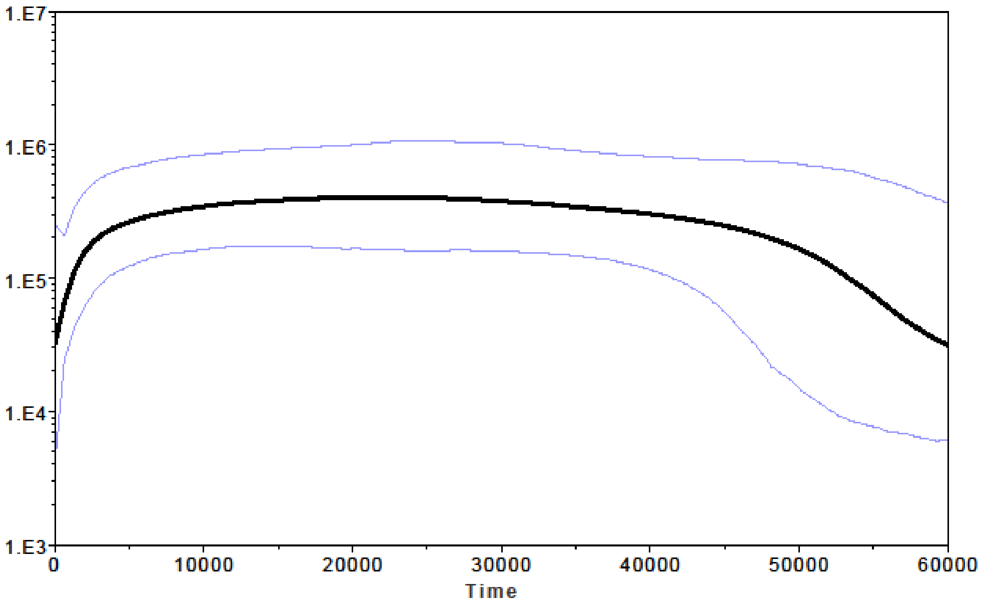

Nyaw (NY)

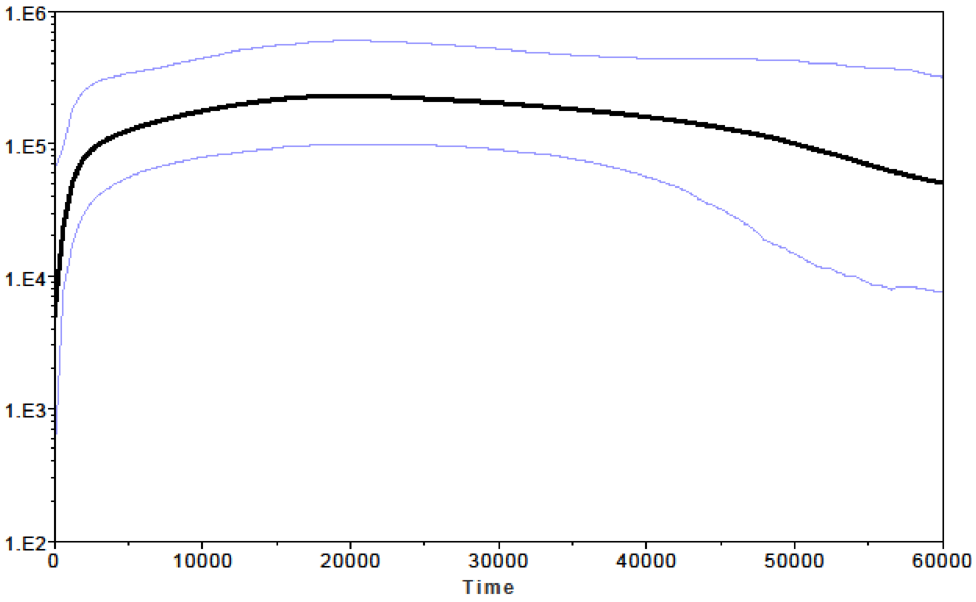

Black Tail (BT1)

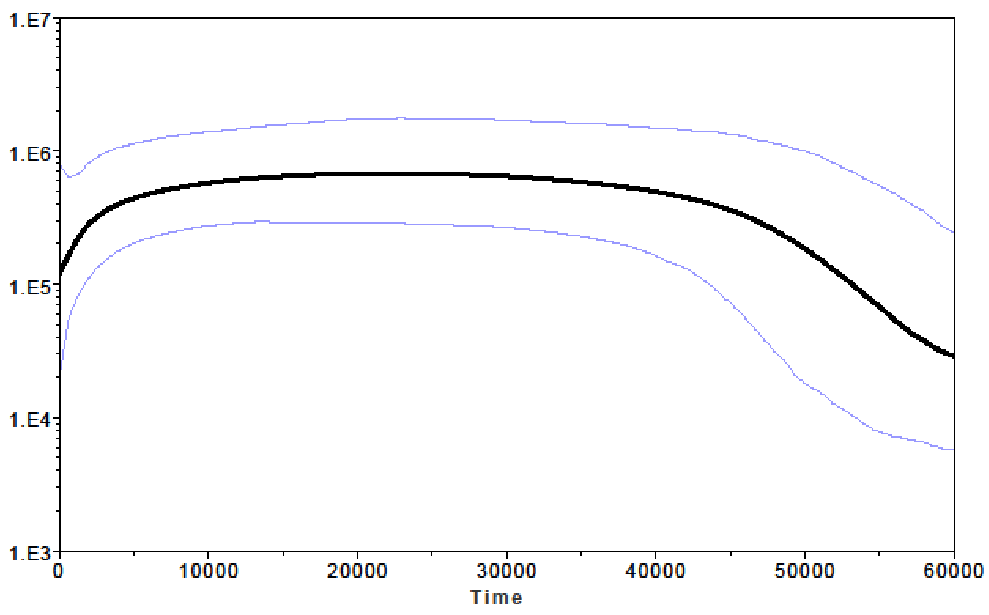

Black Tai2 (BT2)

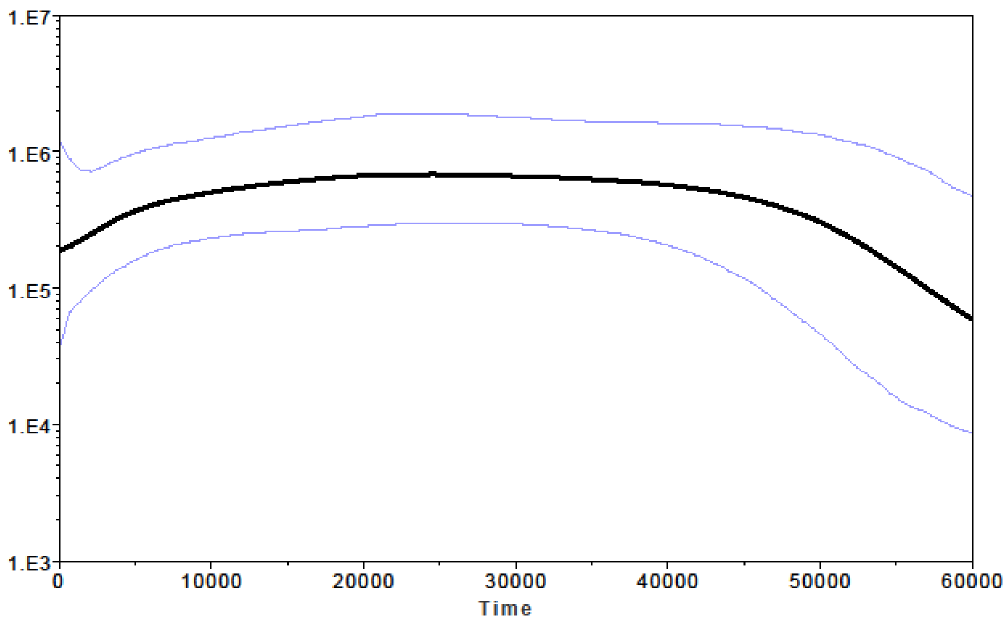

Phuan1 (PU1)

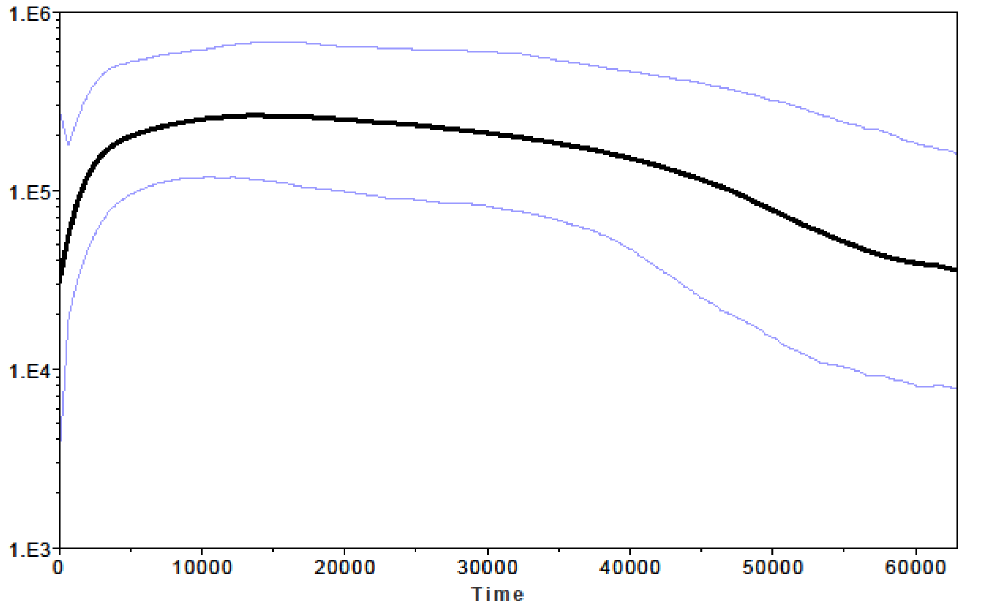

Phuan2 (PU2)

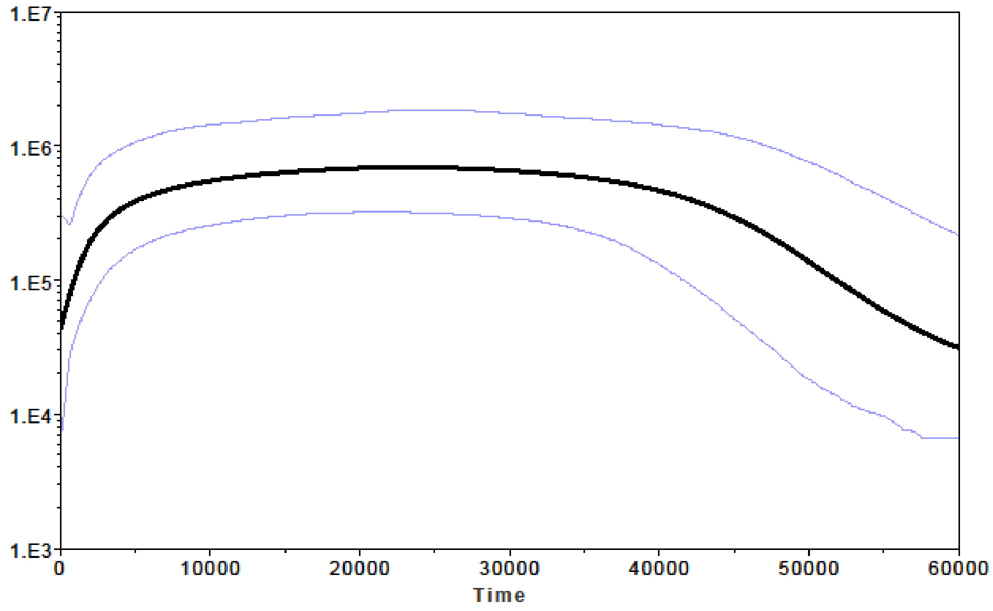

Phuan3 (PU3)

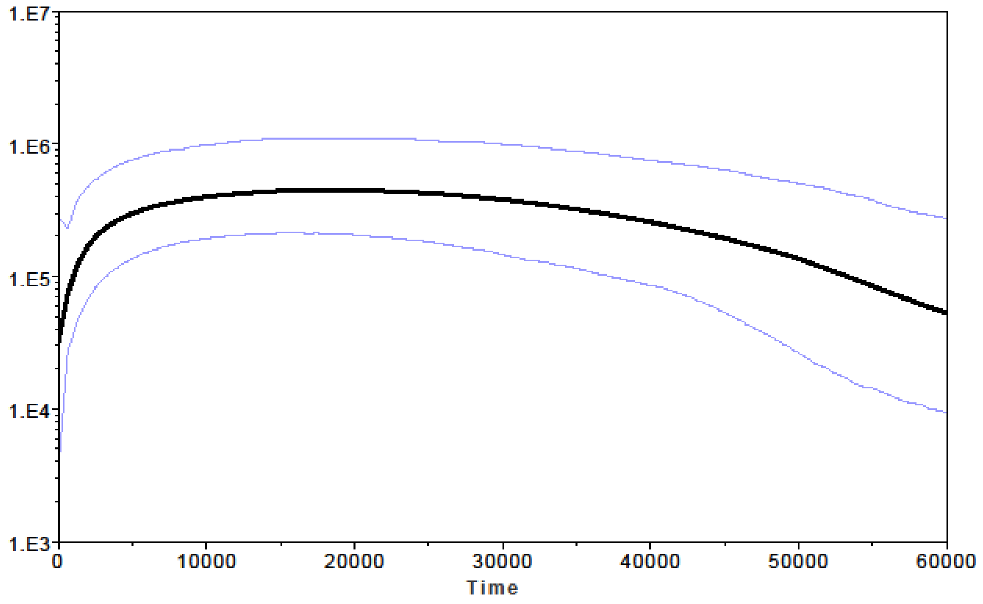

Phuan4 (PU4)

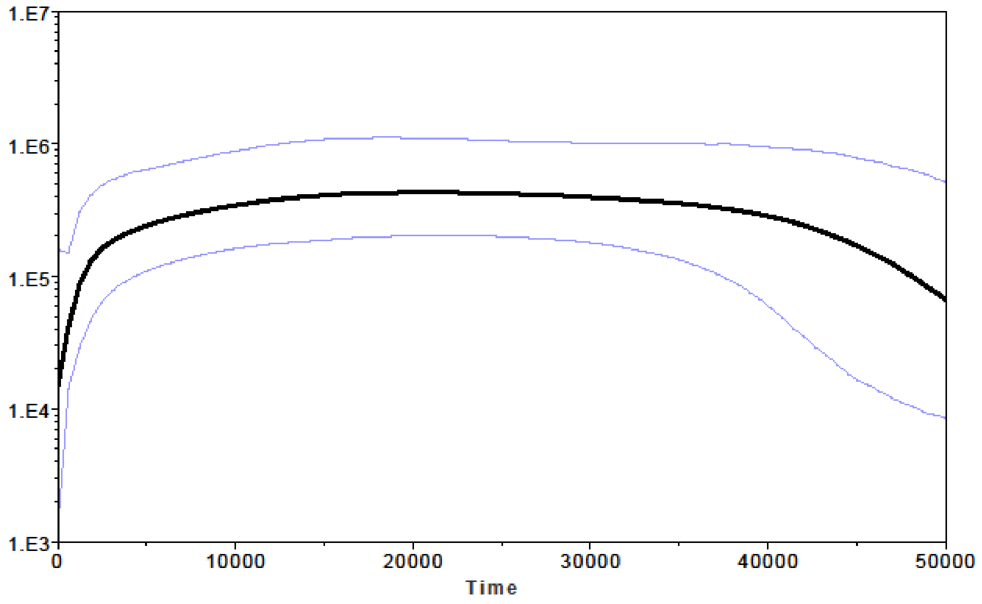

Phuan5 (PU5)

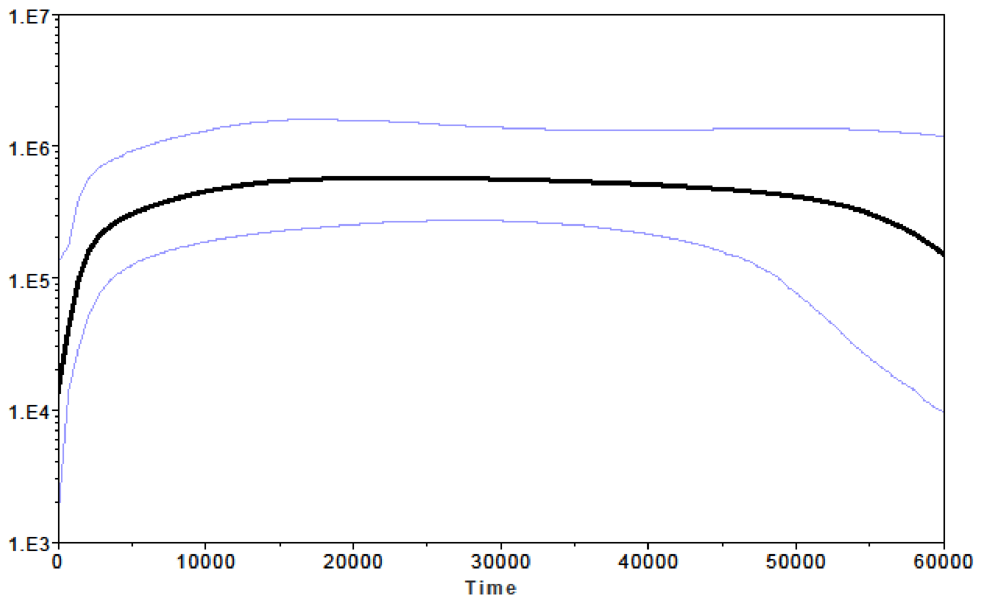

Mon1 (MO1)

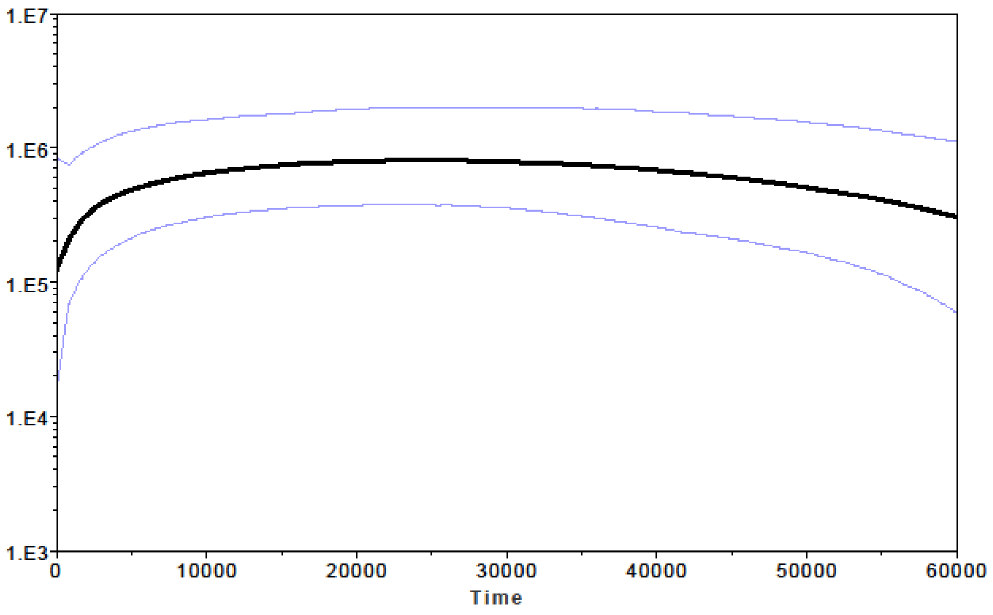

Mon2 (MO2)

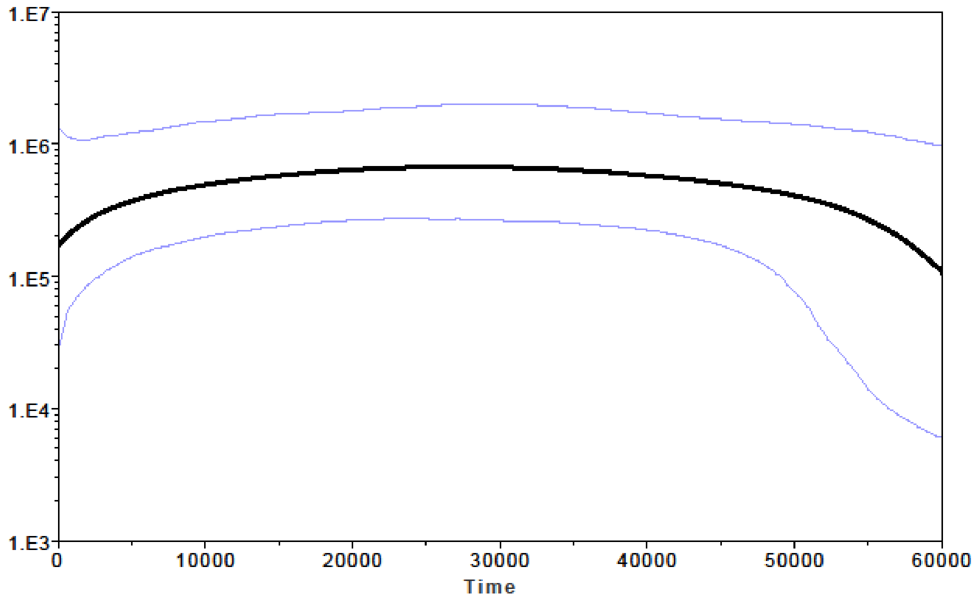

Mon3 (MO3)

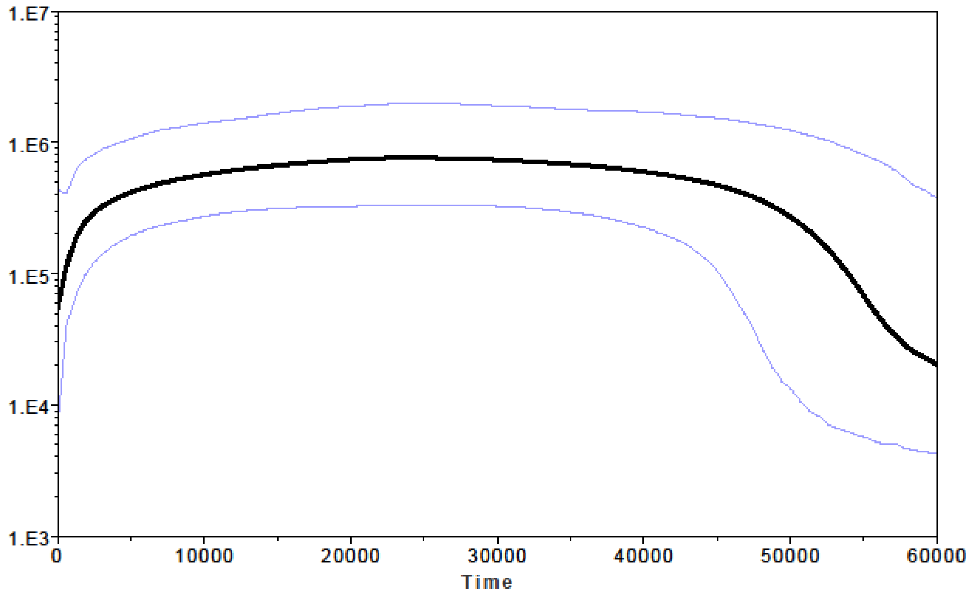

Mon4 (MO4)

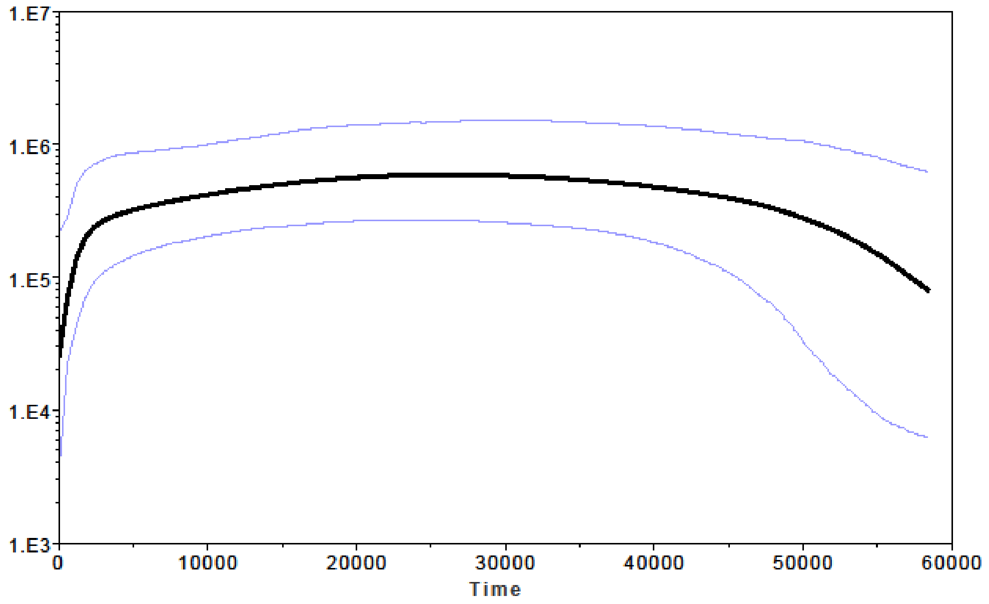

Mon5 (MO5)

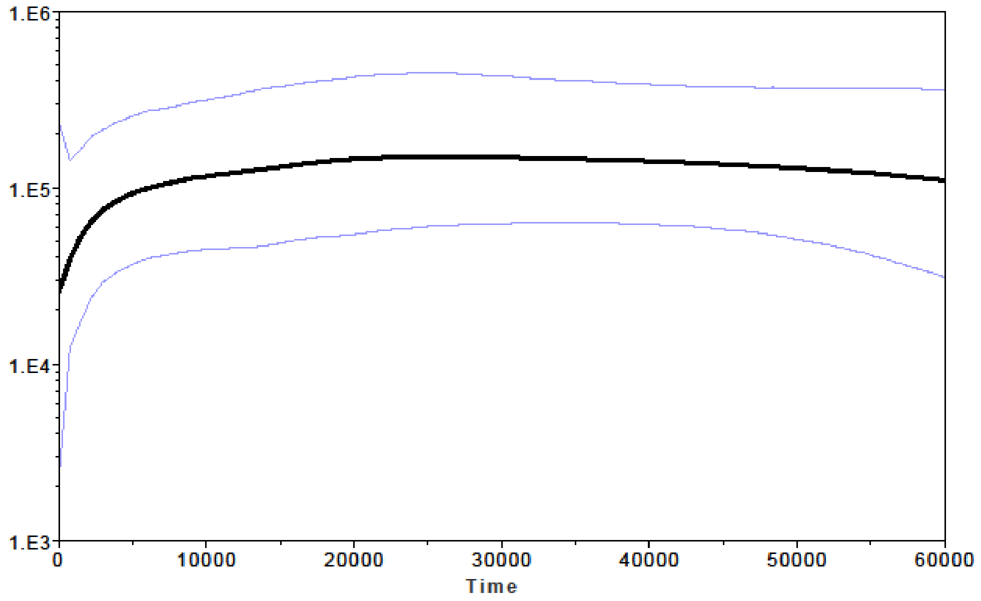

Khmer1 (KH1)

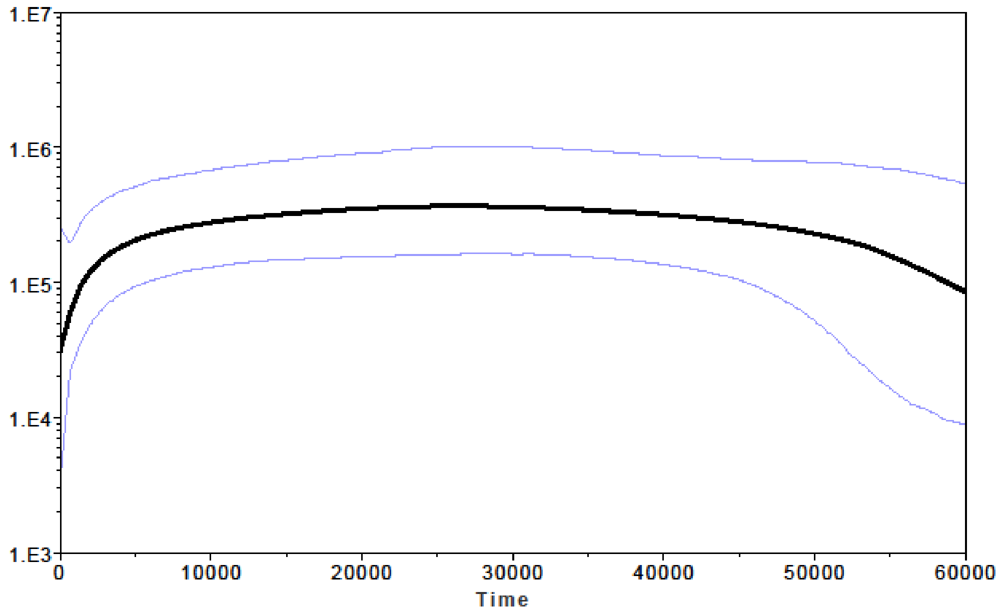

Khmer2 (KH2)

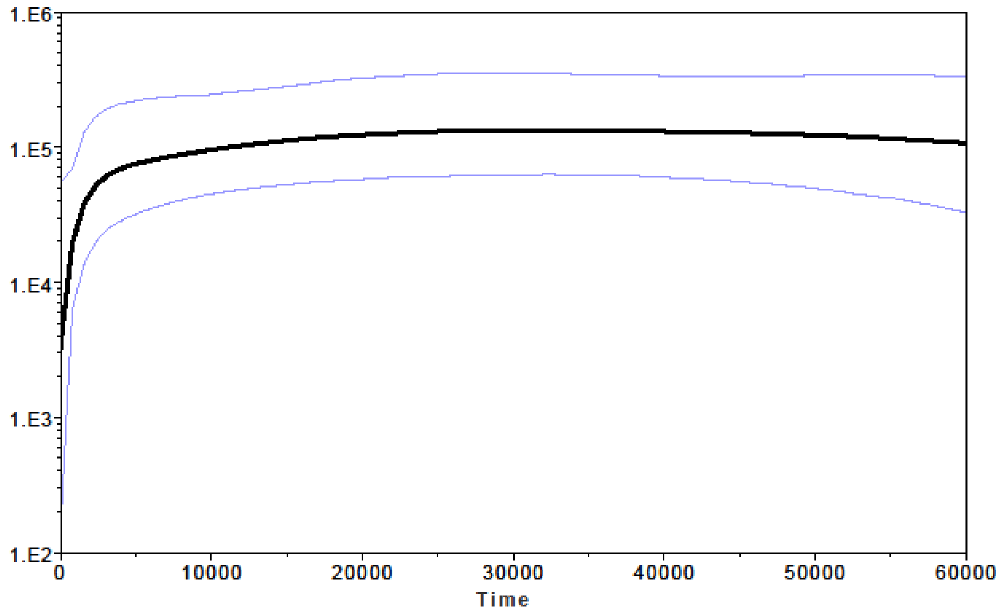

Nyahkur (BO)

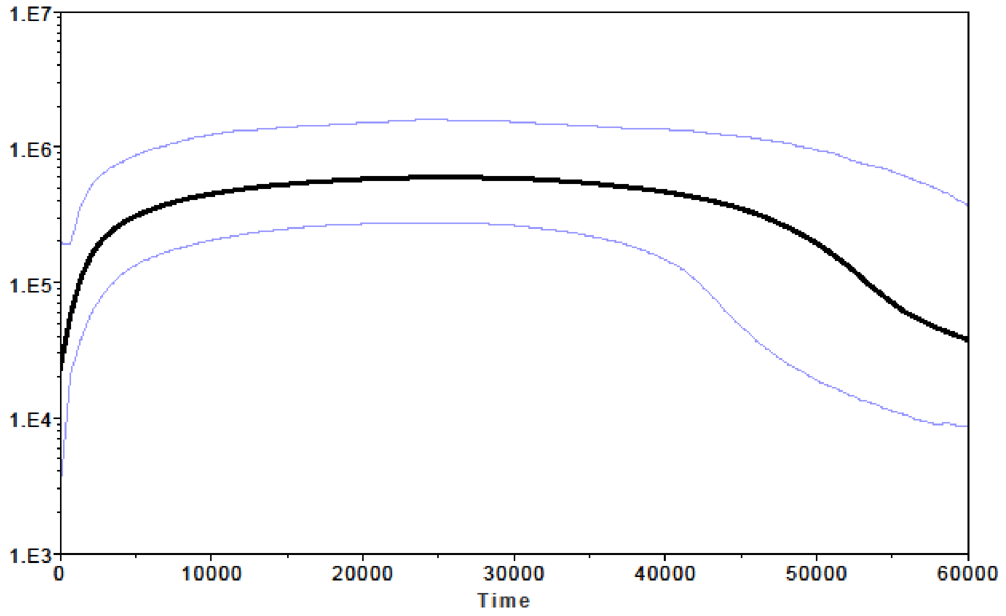

Suay (SU)

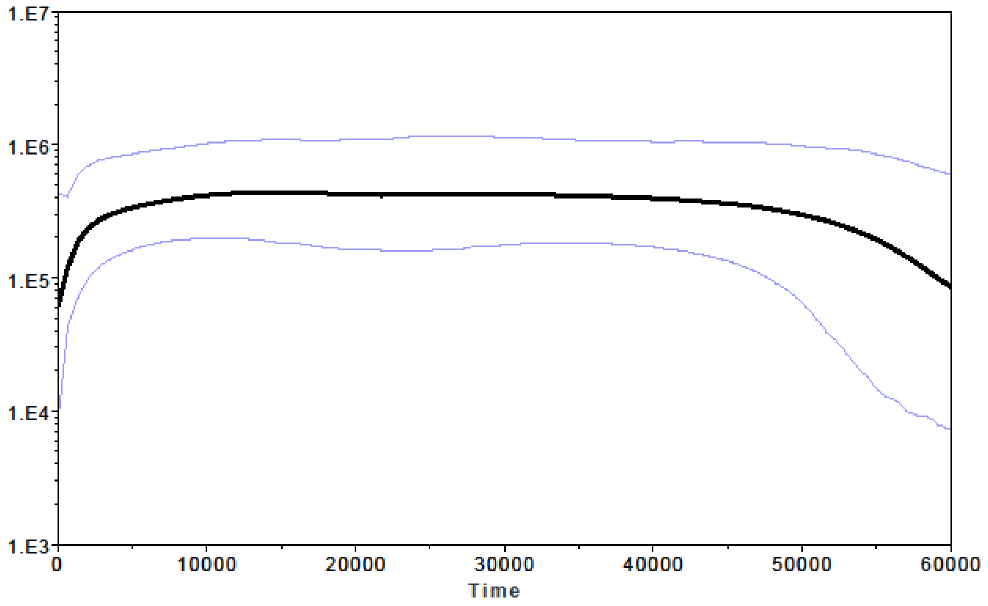

Soa (SO)

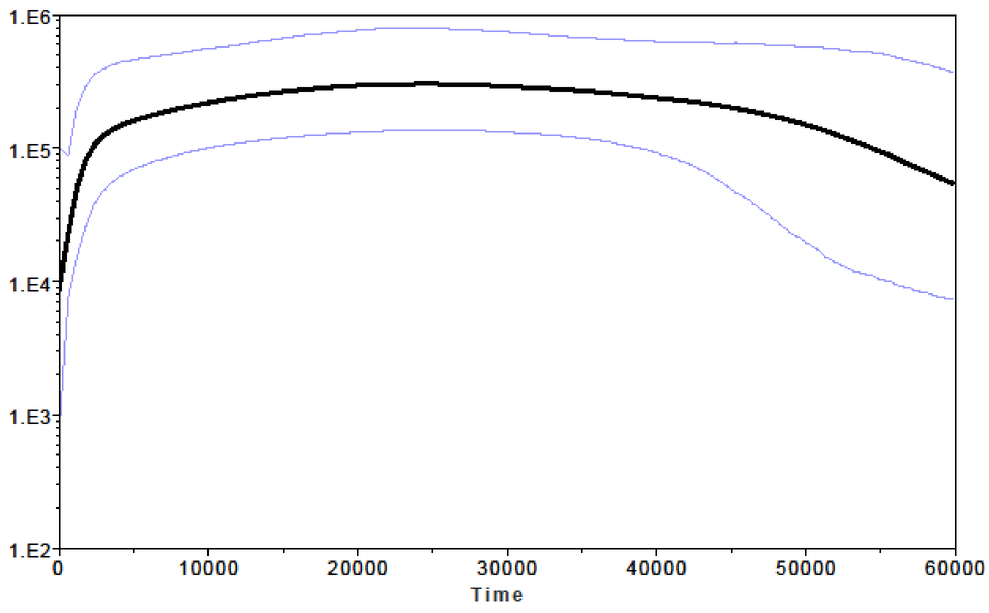

Bru (BU)

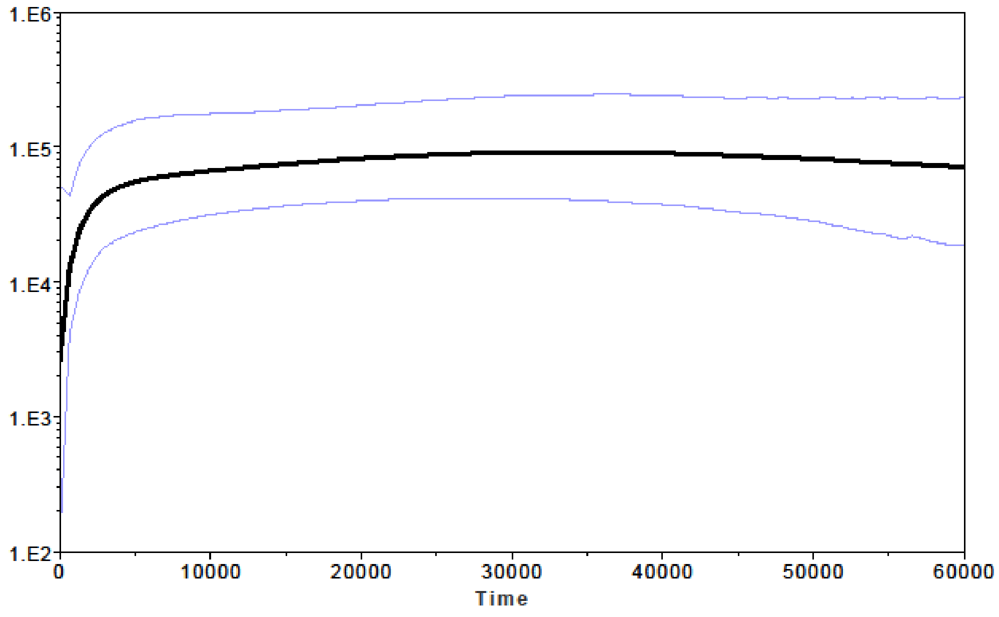

H’tin1 (TN1)

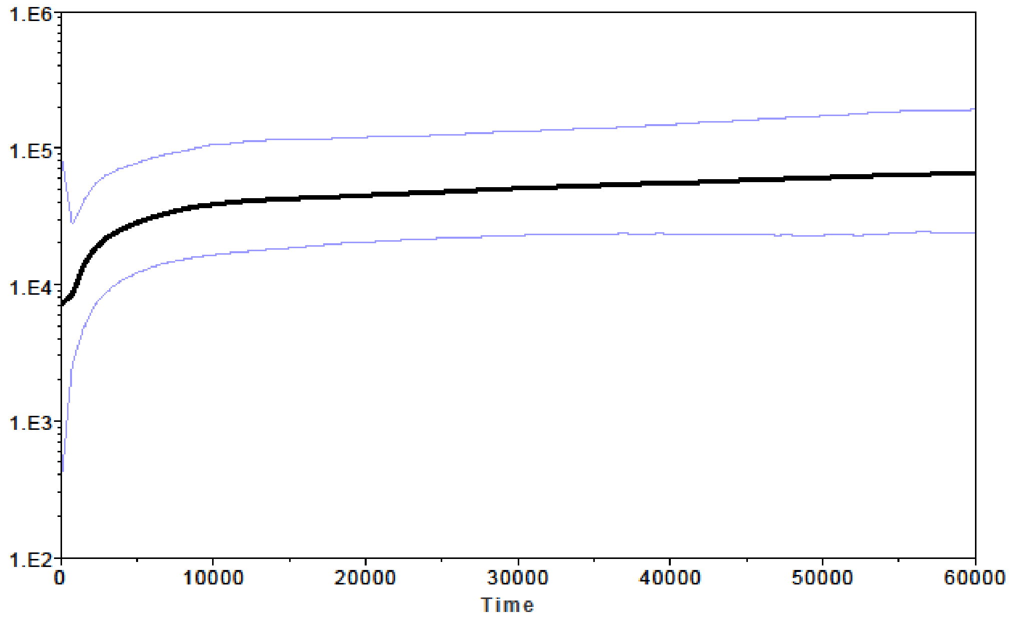

H’tin2 (TN2)

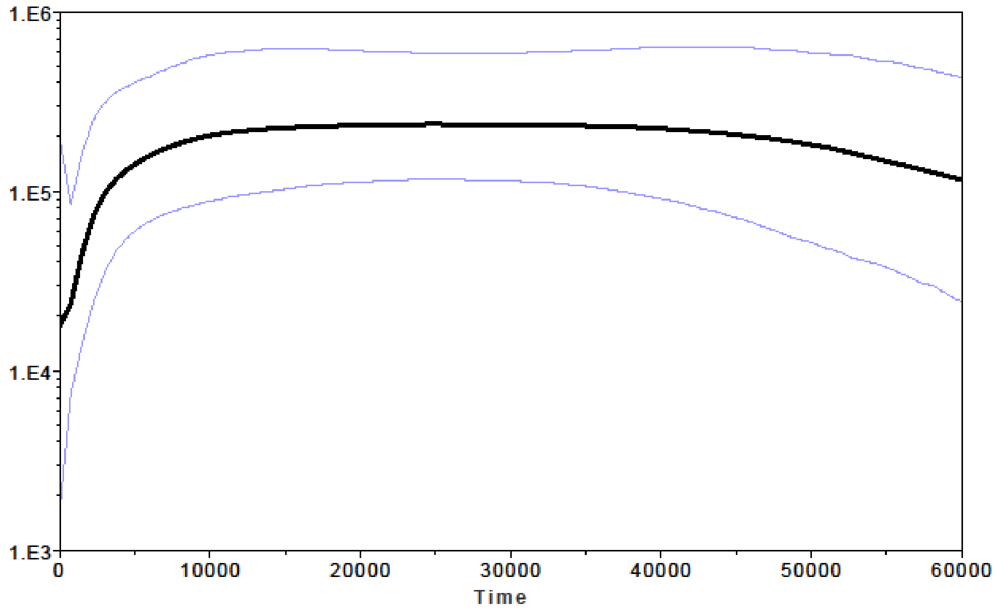

H’tin3 (TN3)

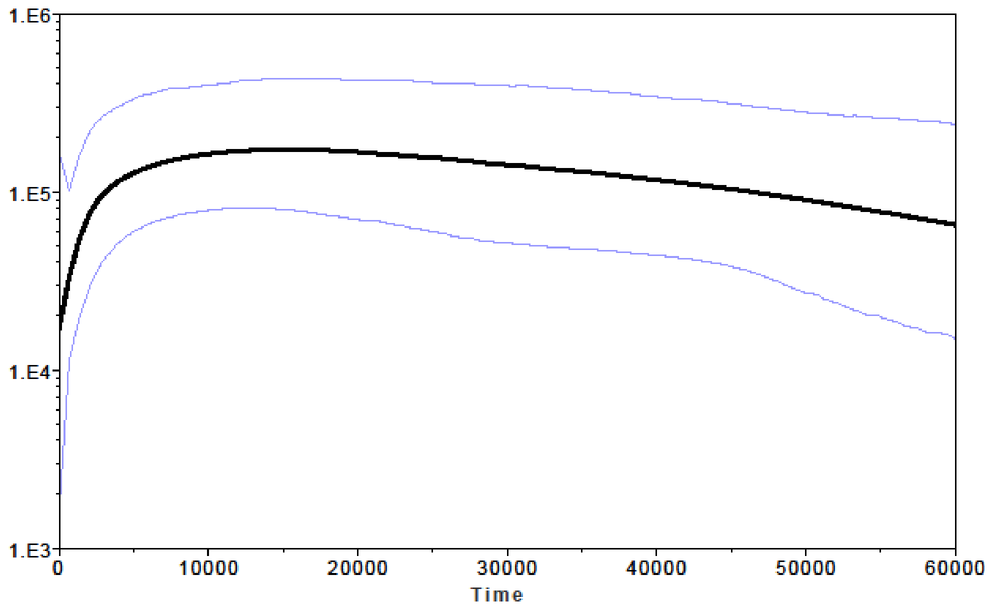

Khamu (KA)

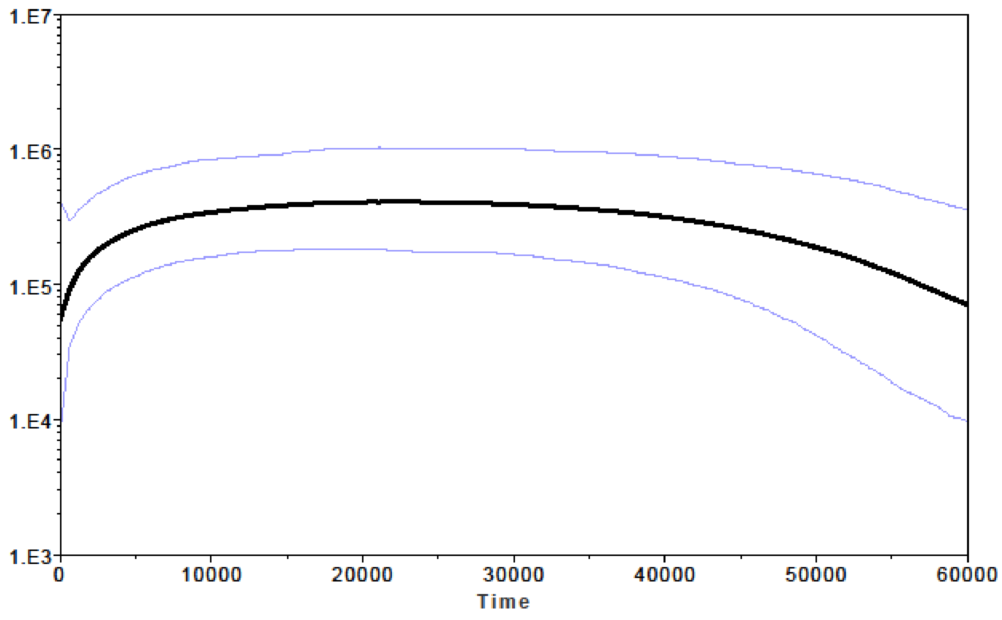

Blang1 (BL1)

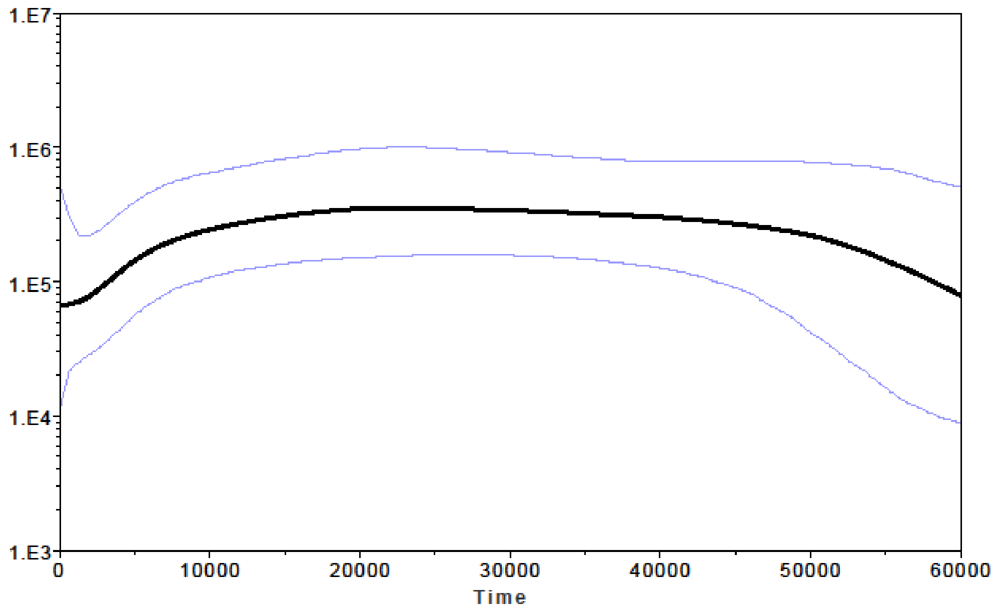

Blang2 (BL2)

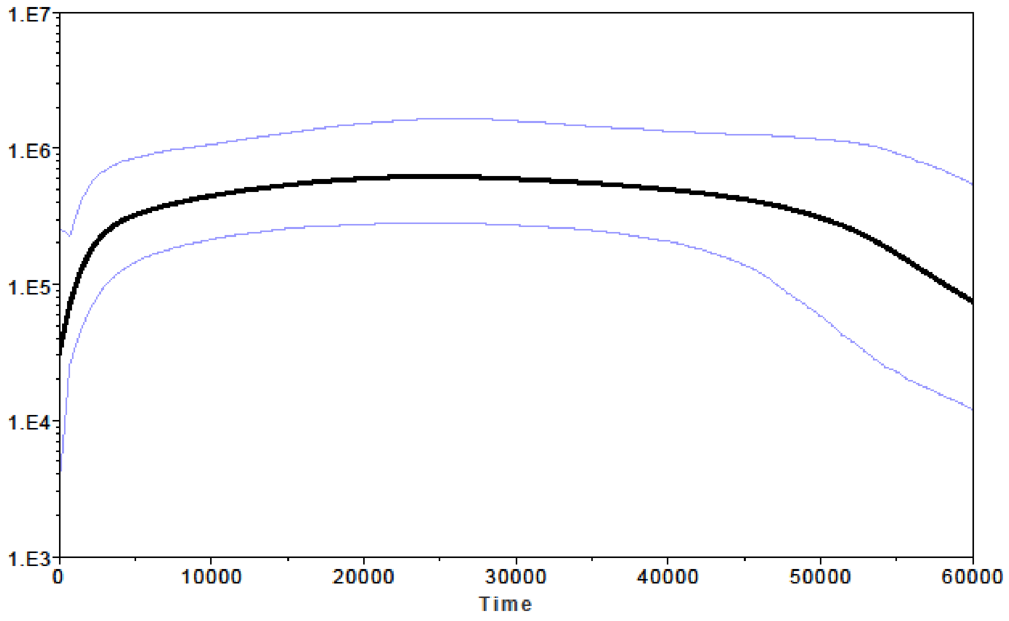

Paluang (PL)

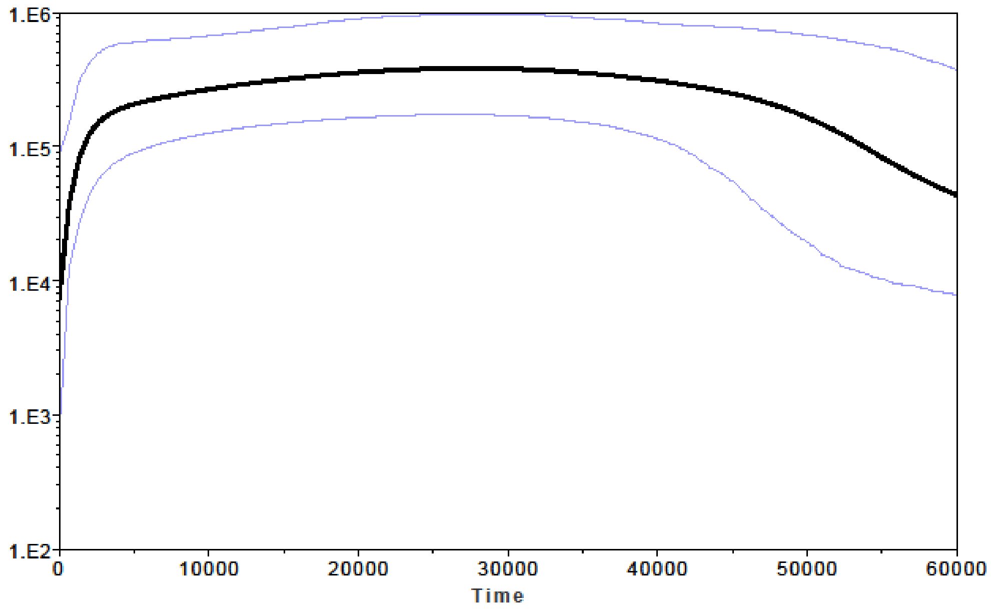

Lawa1 (LW1)

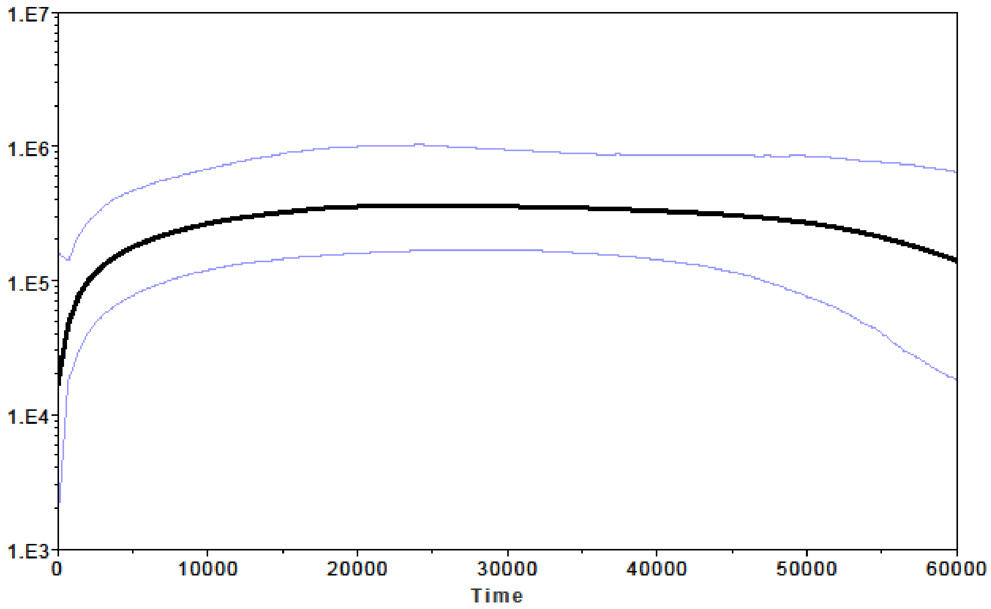

Lawa2 (LW2)

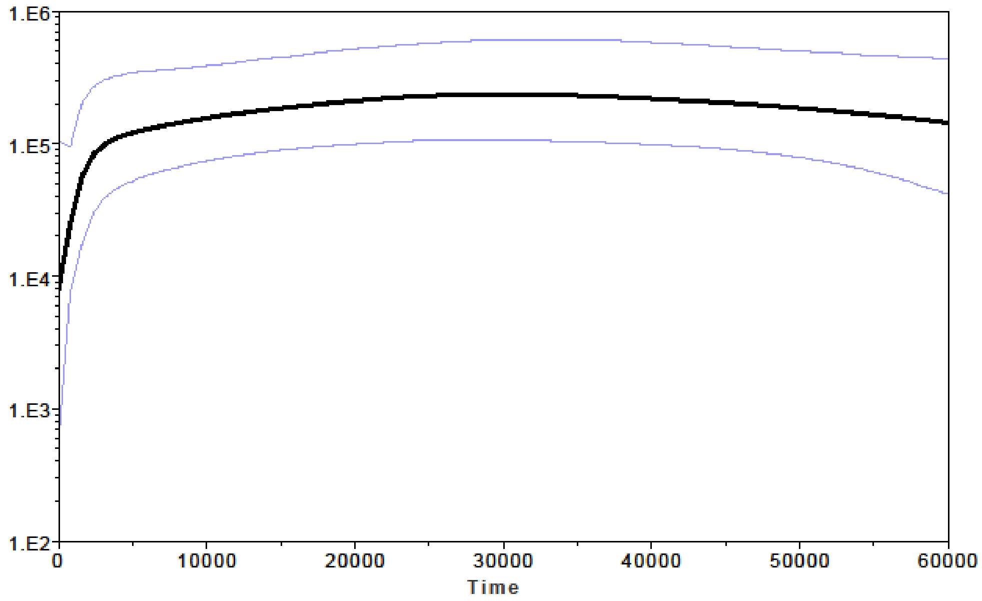

Lawa3 (LW3)

Fig. S6 Bayesian skyline plots show the effective population size change over time in each studied population. Details concerning the settings are provided in the Methods.

Test1 (Northern Thai people)

1,000 best simulations

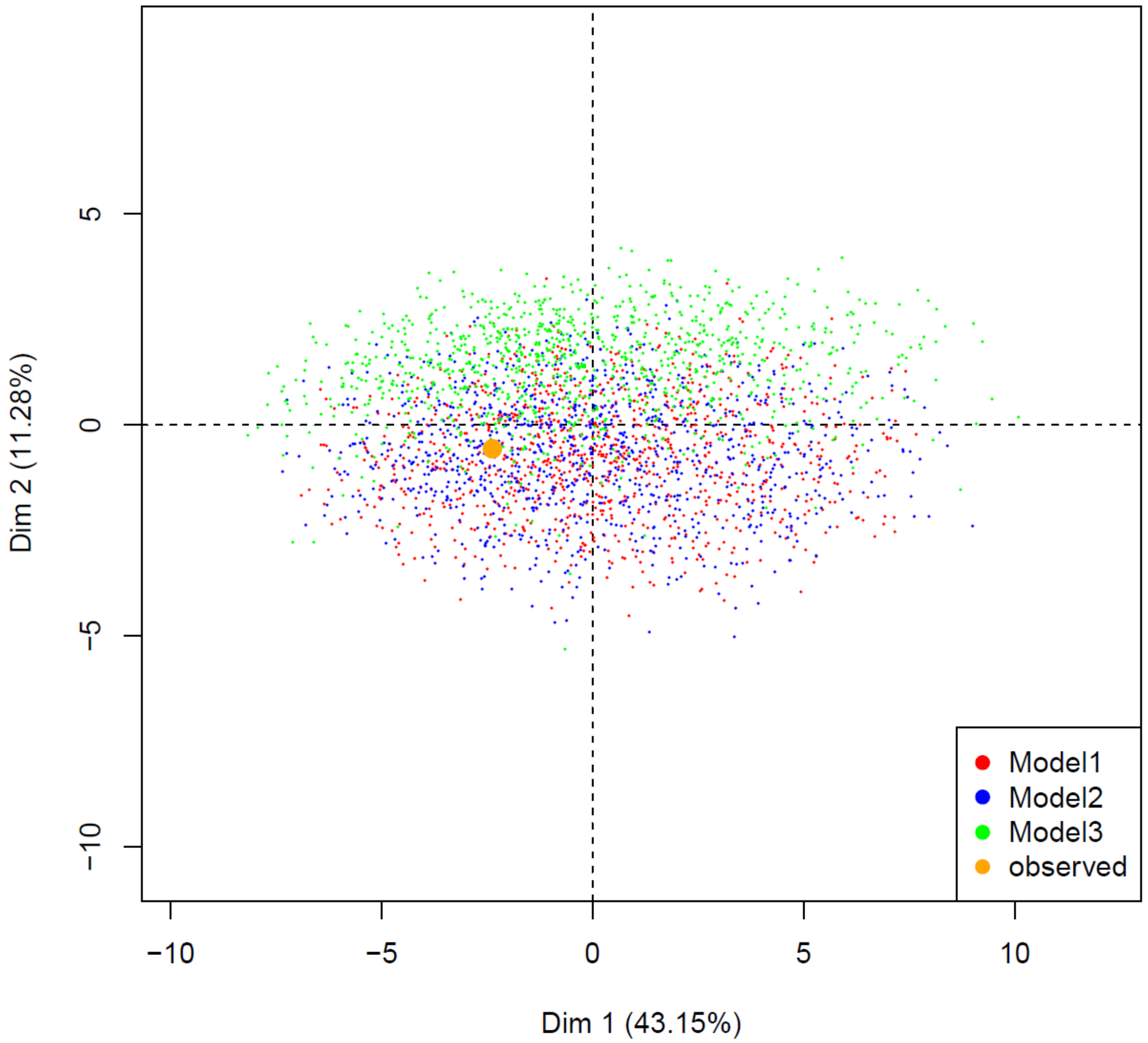

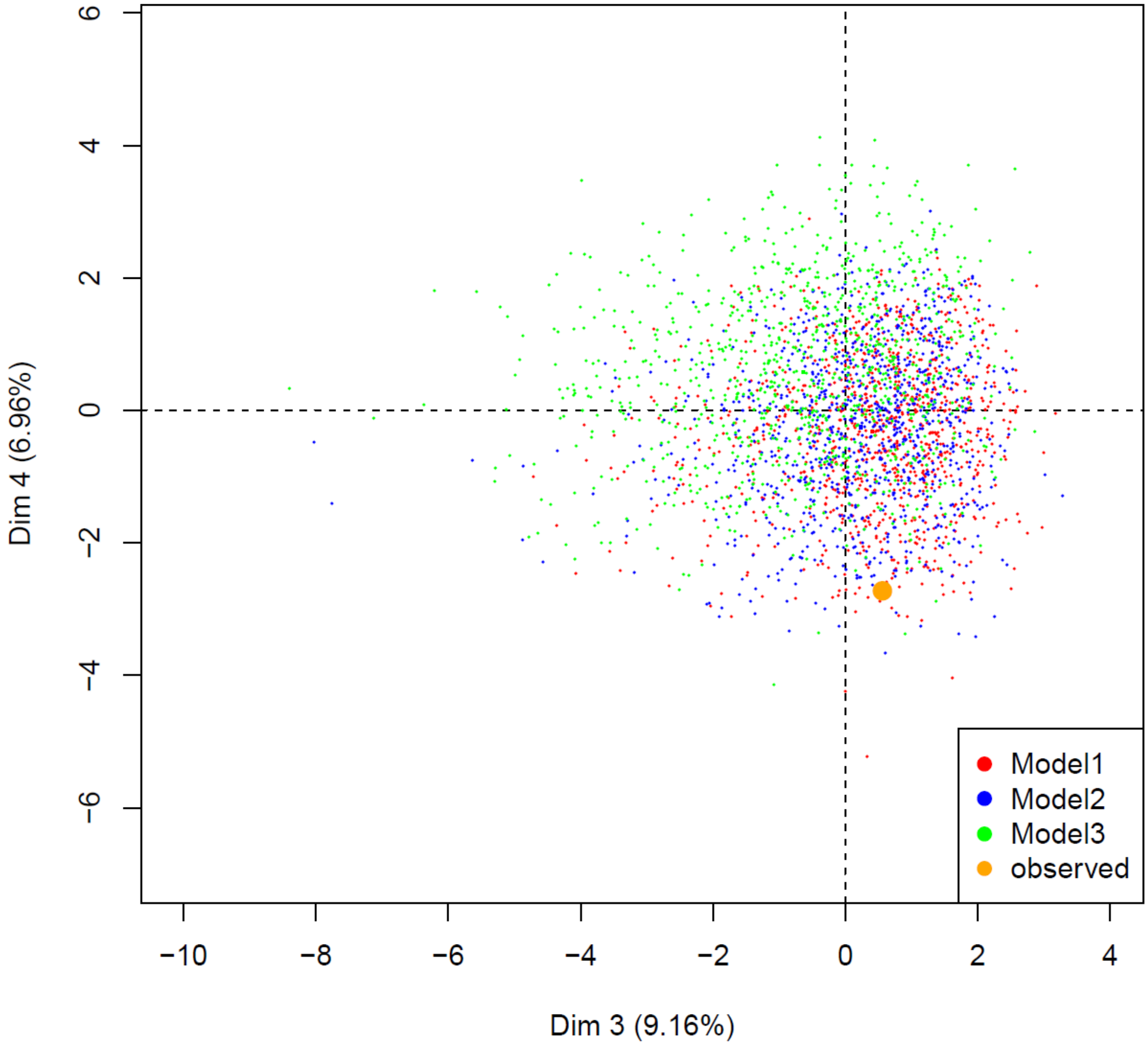

Model1
Model2
Model3
observed
Dim 3 (9.16%)
Dim 4 (6.96%)
−10
−8
−6
−4
−2
0
2
4
6

5,000 best simulations

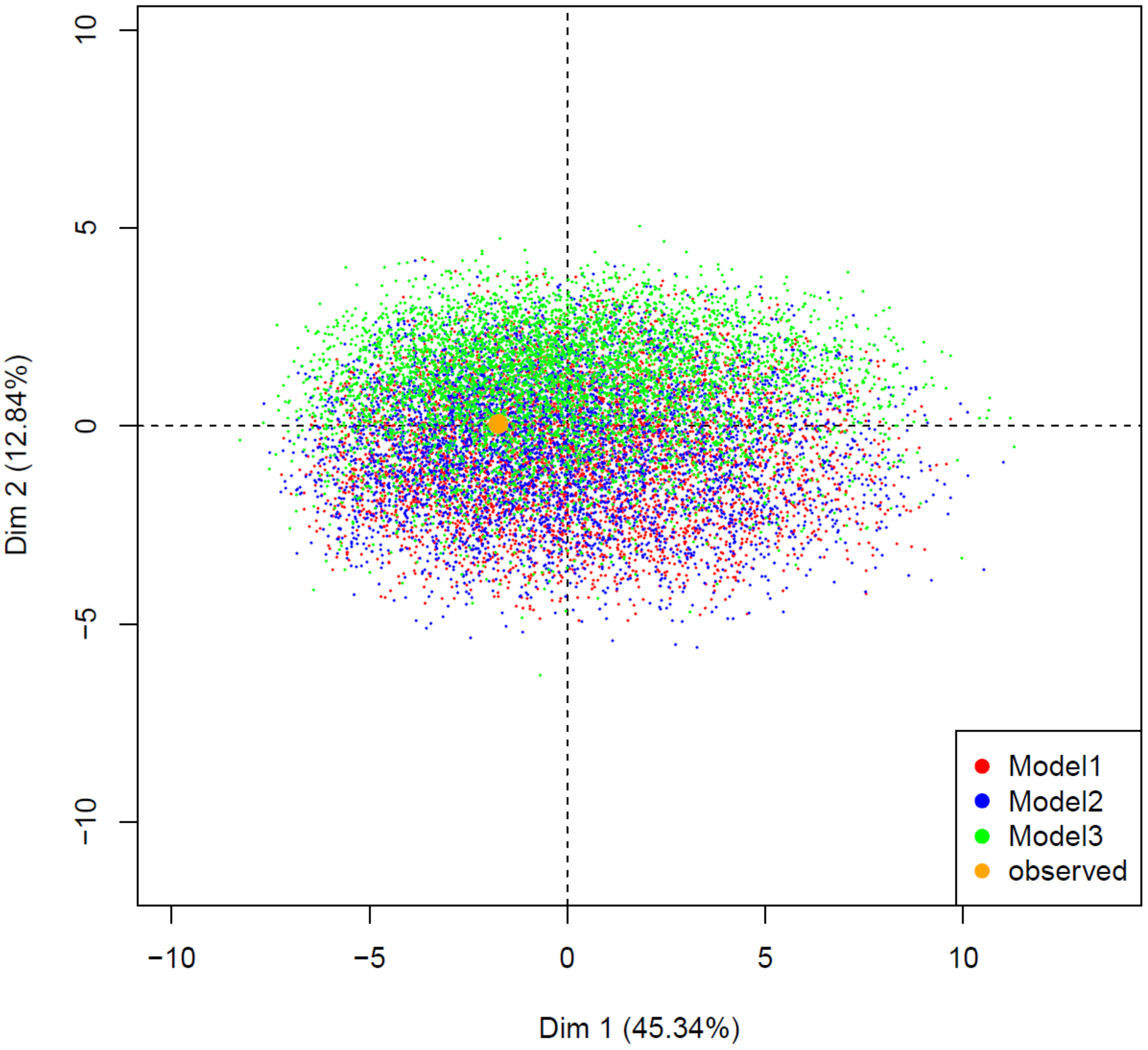

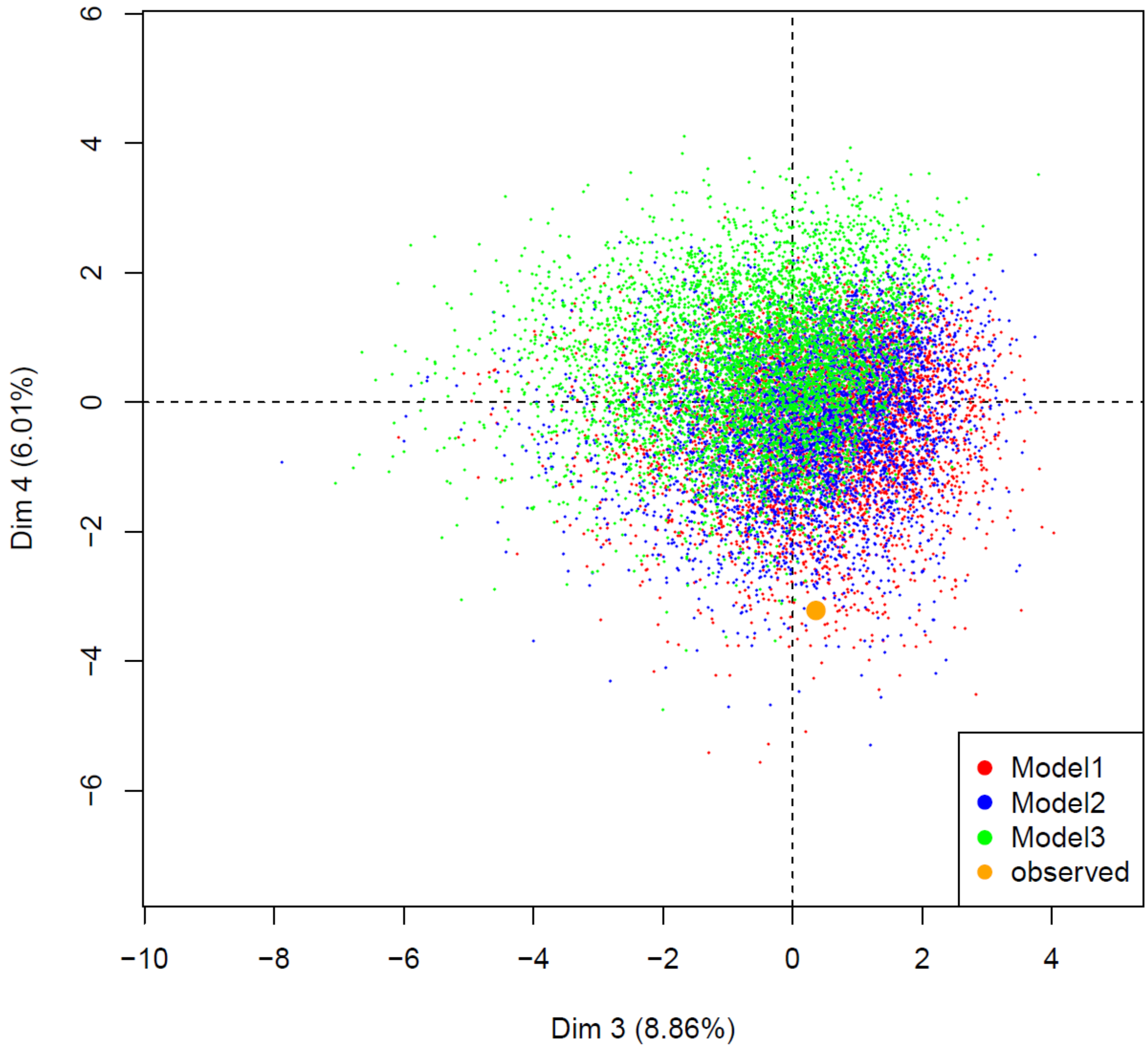

6
4
2
0
−2
−4
−6
Dim 4 (6.01%)
−10
−8
−6
−4
−2
0
2
4
Dim 3 (8.86%)
Model1
Model2
Model3
observed

Test 2 (Northeastern Thai and Lao people)

1,000 best simulations

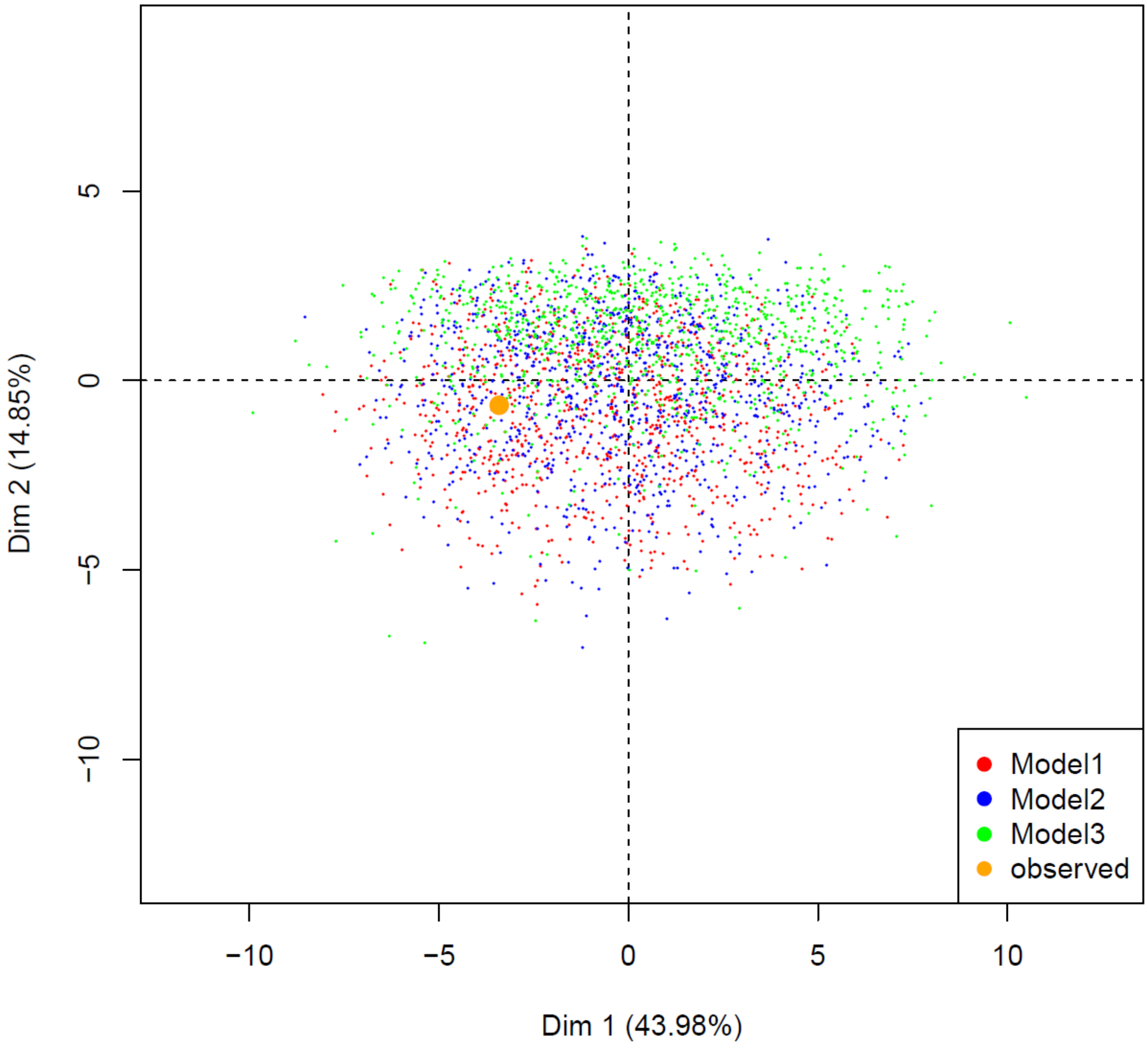

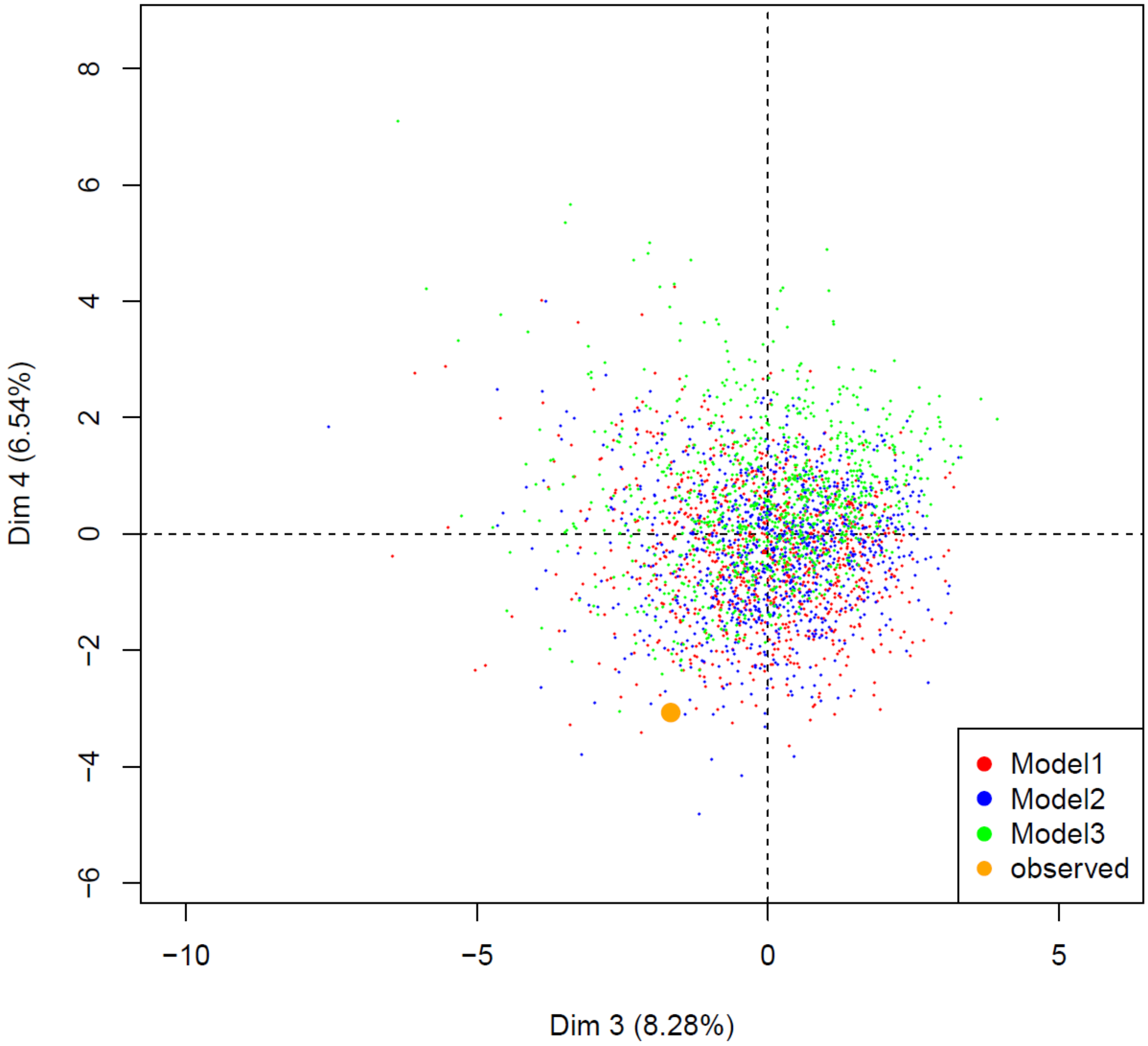

Dim 4 (6.54%)
Dim 3 (8.28%)
8
6
4
2
0
−2
−4
−6
−10
−5
0
5
Model1
Model2
Model3
observed

5,000 best simulations

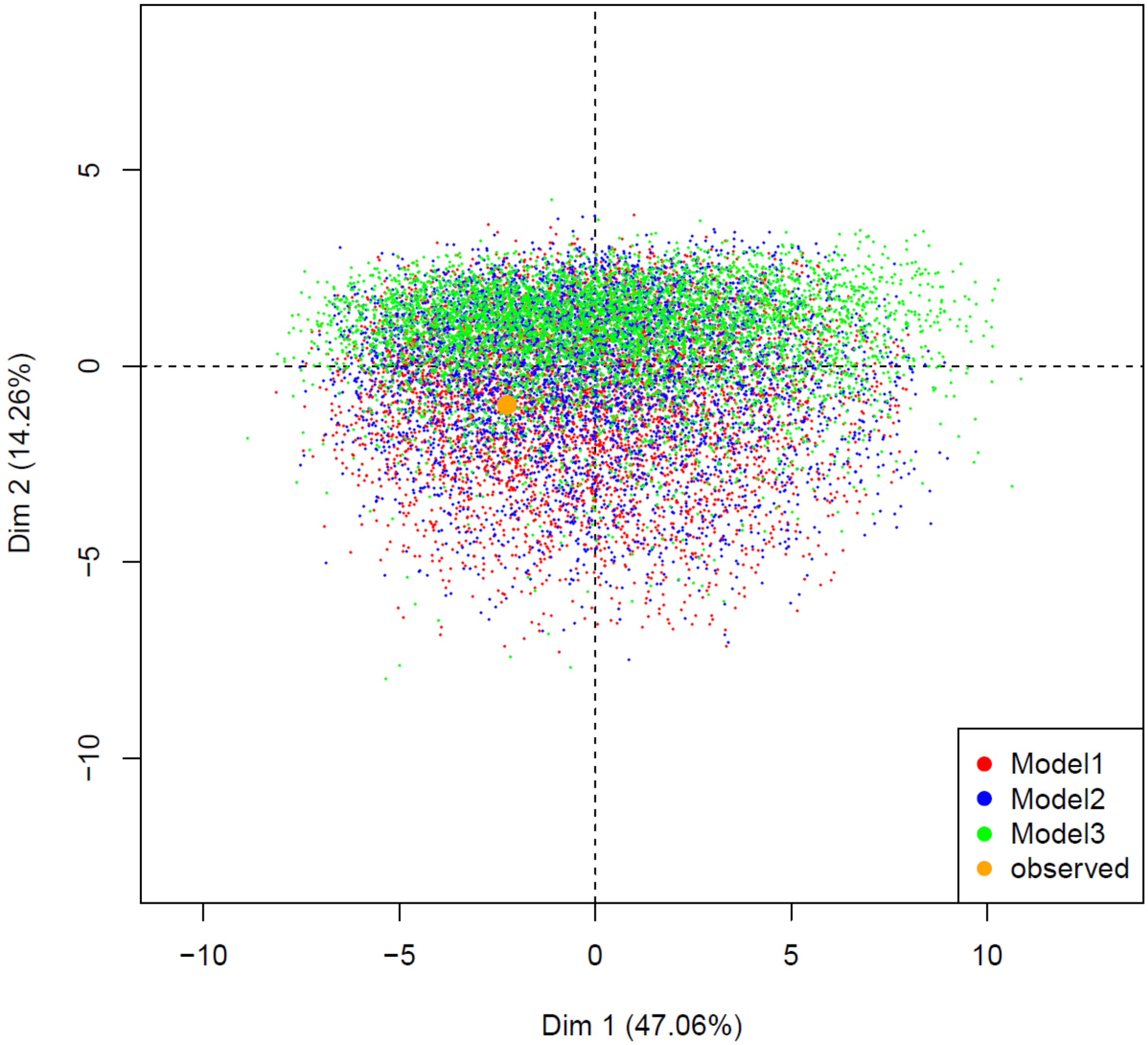

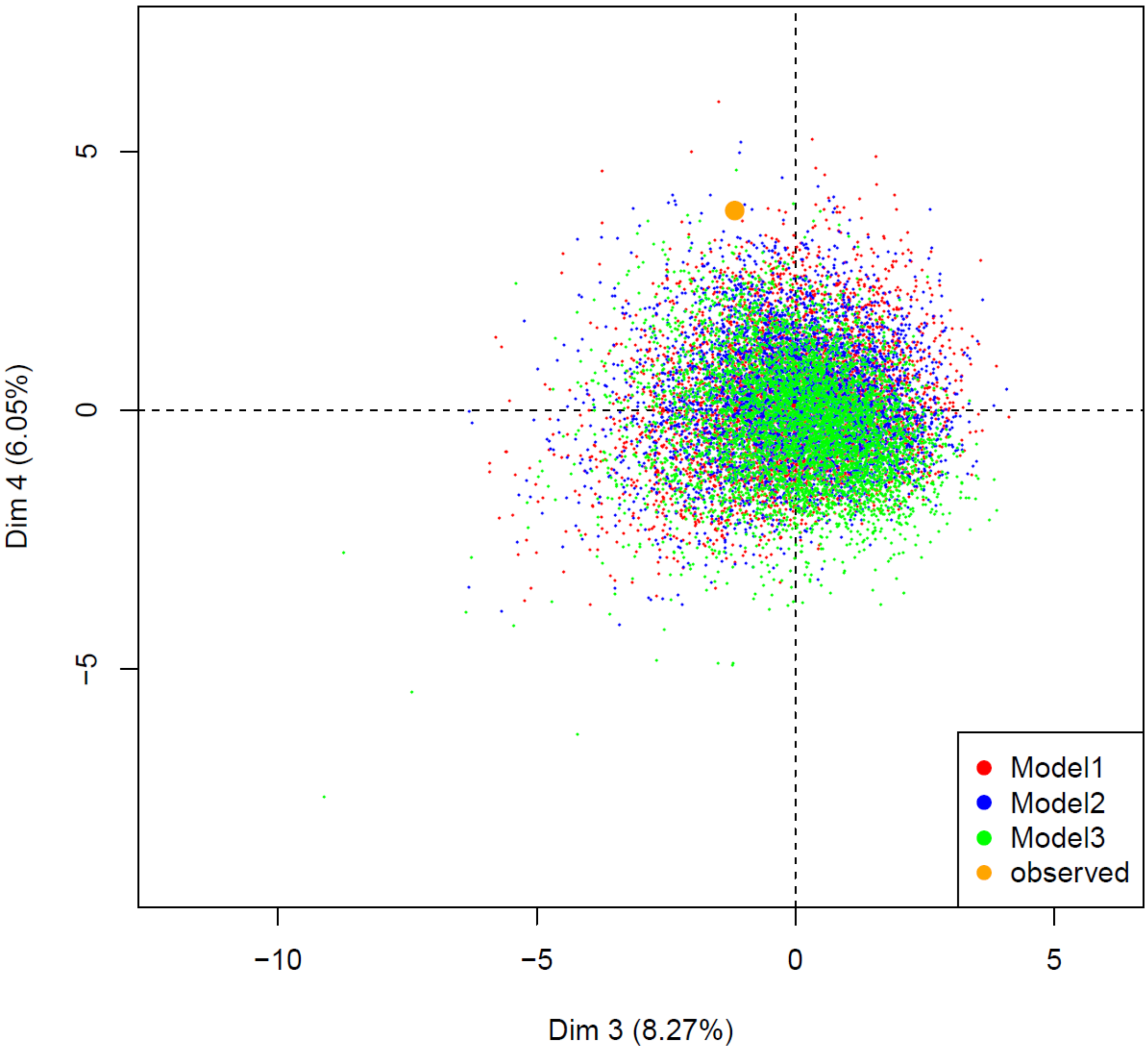

Model1
Model2
Model3
observed
Dim 3 (8.27%)
Dim 4 (6.05%)
−10
−5
0
5
5
0
−5

Test 3 (Northeastern Thai people)

1,000 best simulations

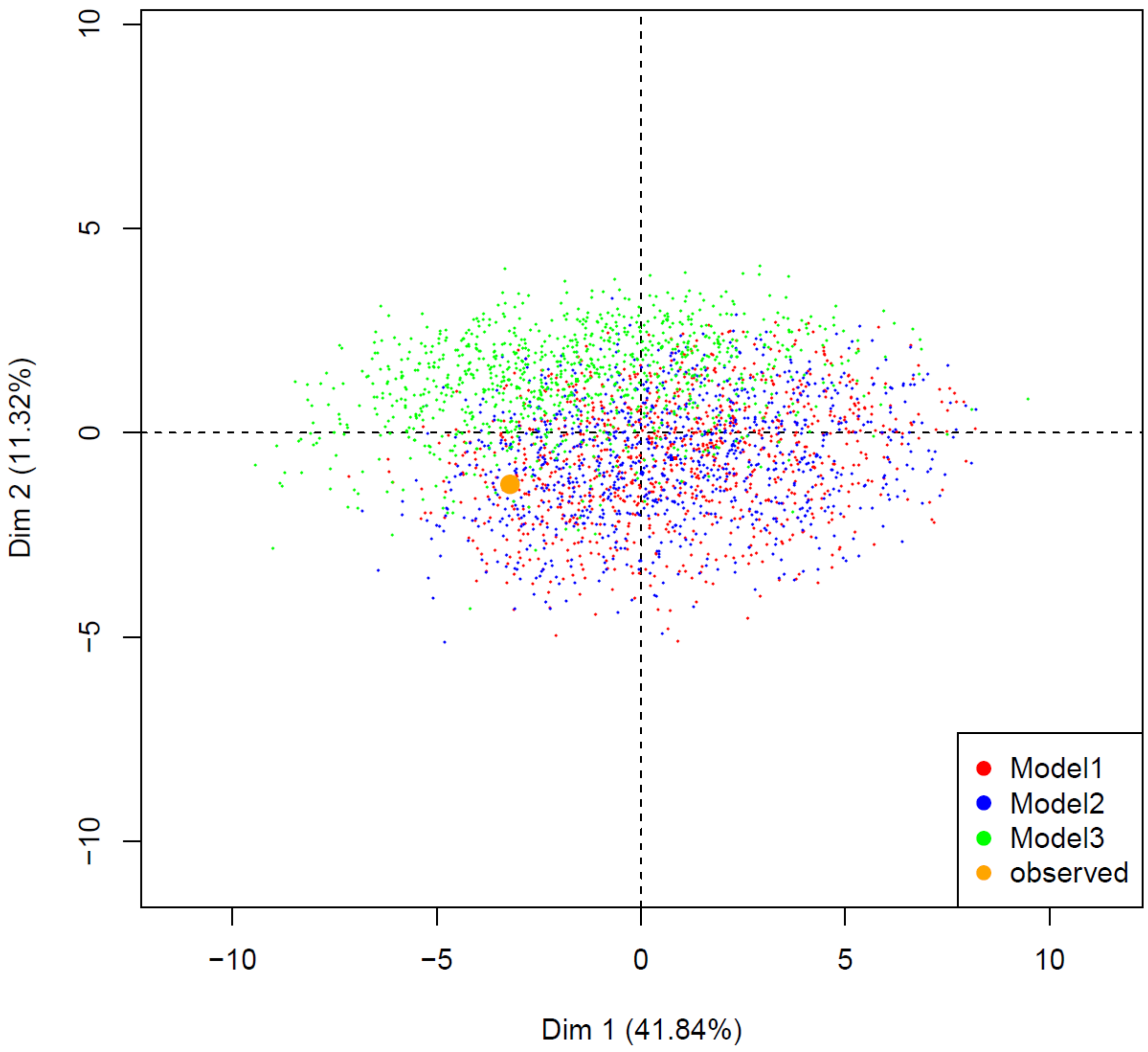

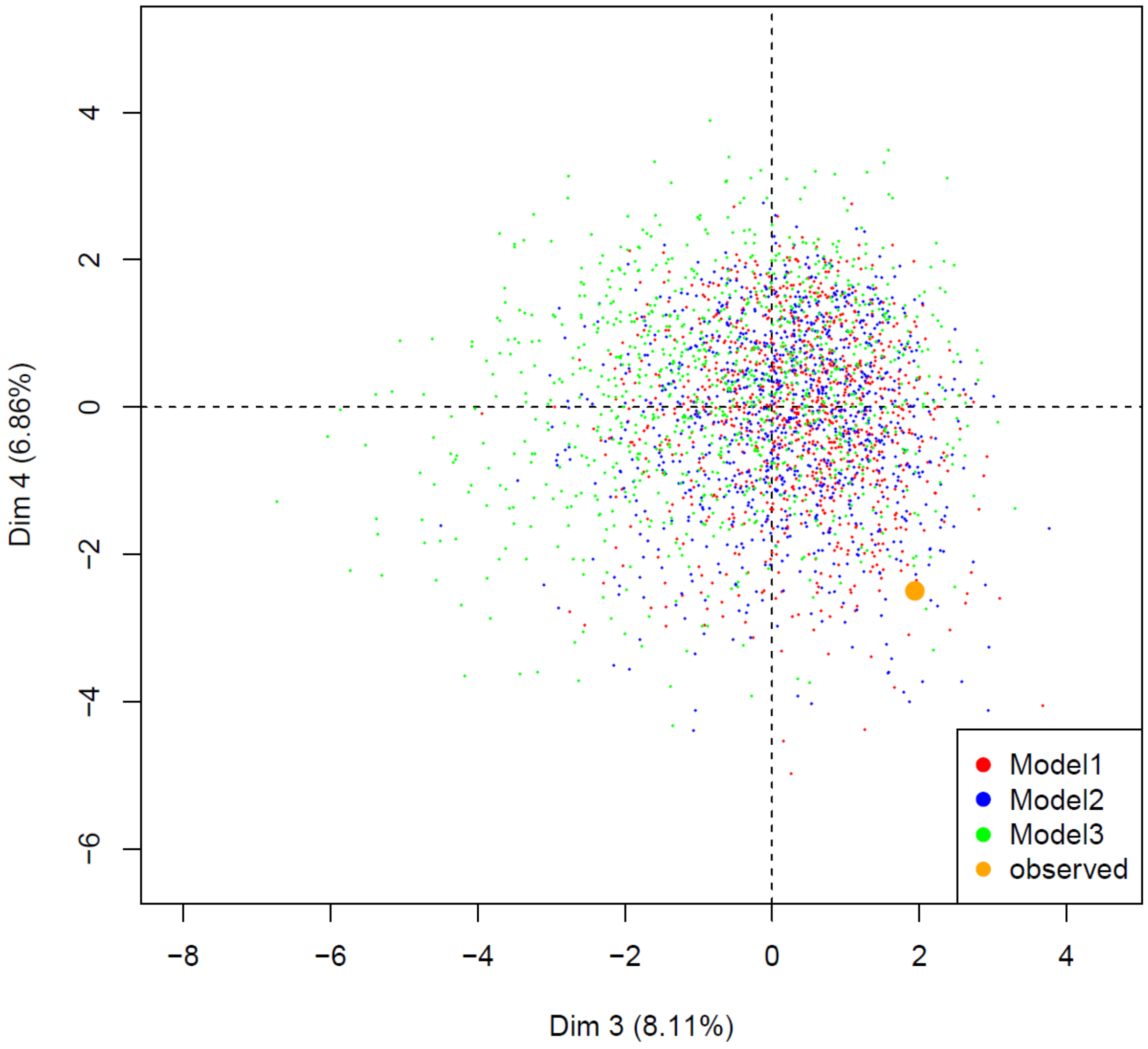

Dim 4 (6.86%)
4
2
0
−2
−4
−6
−8
−6
−4
−2
0
2
4
Dim 3 (8.11%)
Model1
Model2
Model3
observed

5,000 best simulations

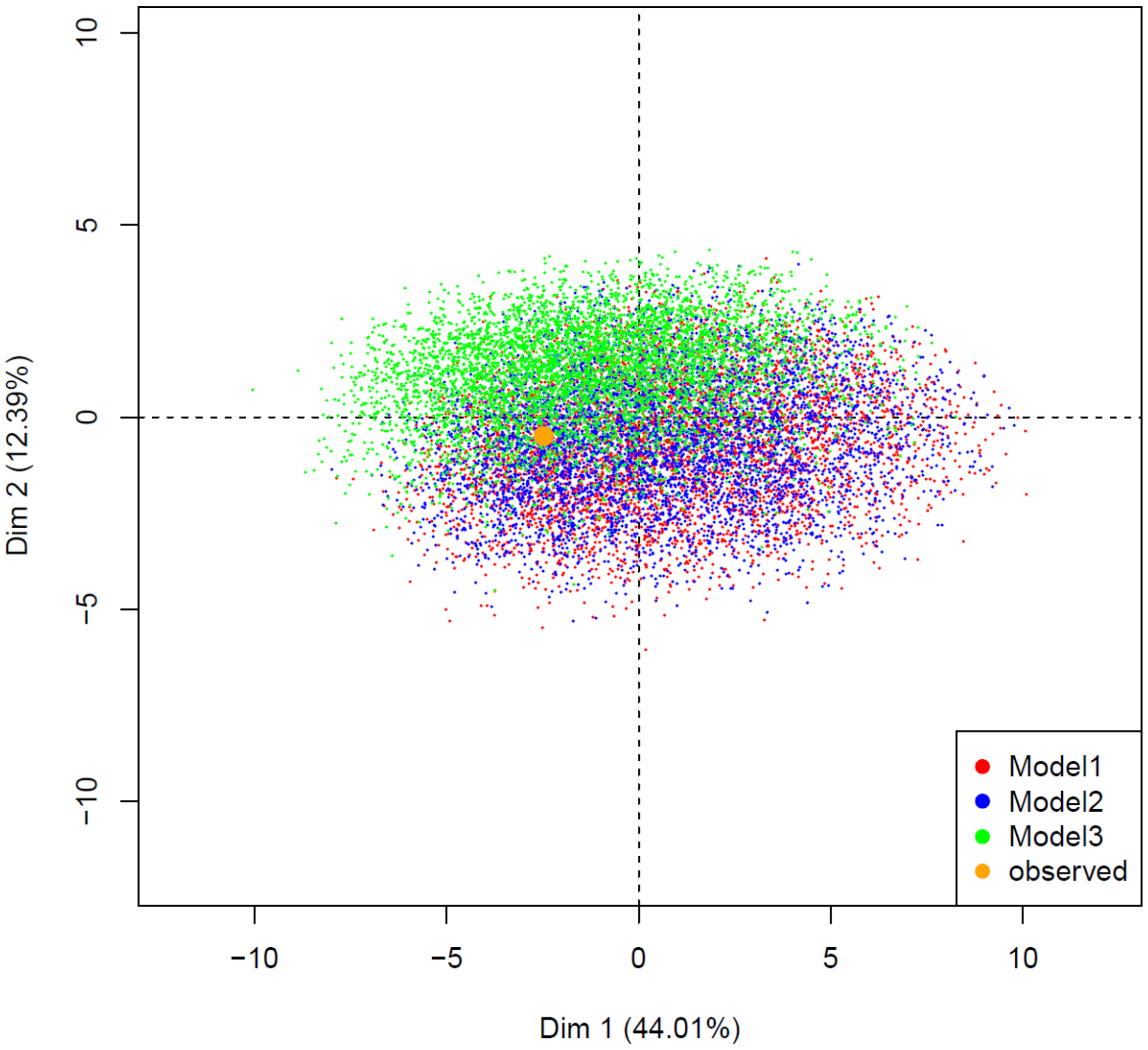

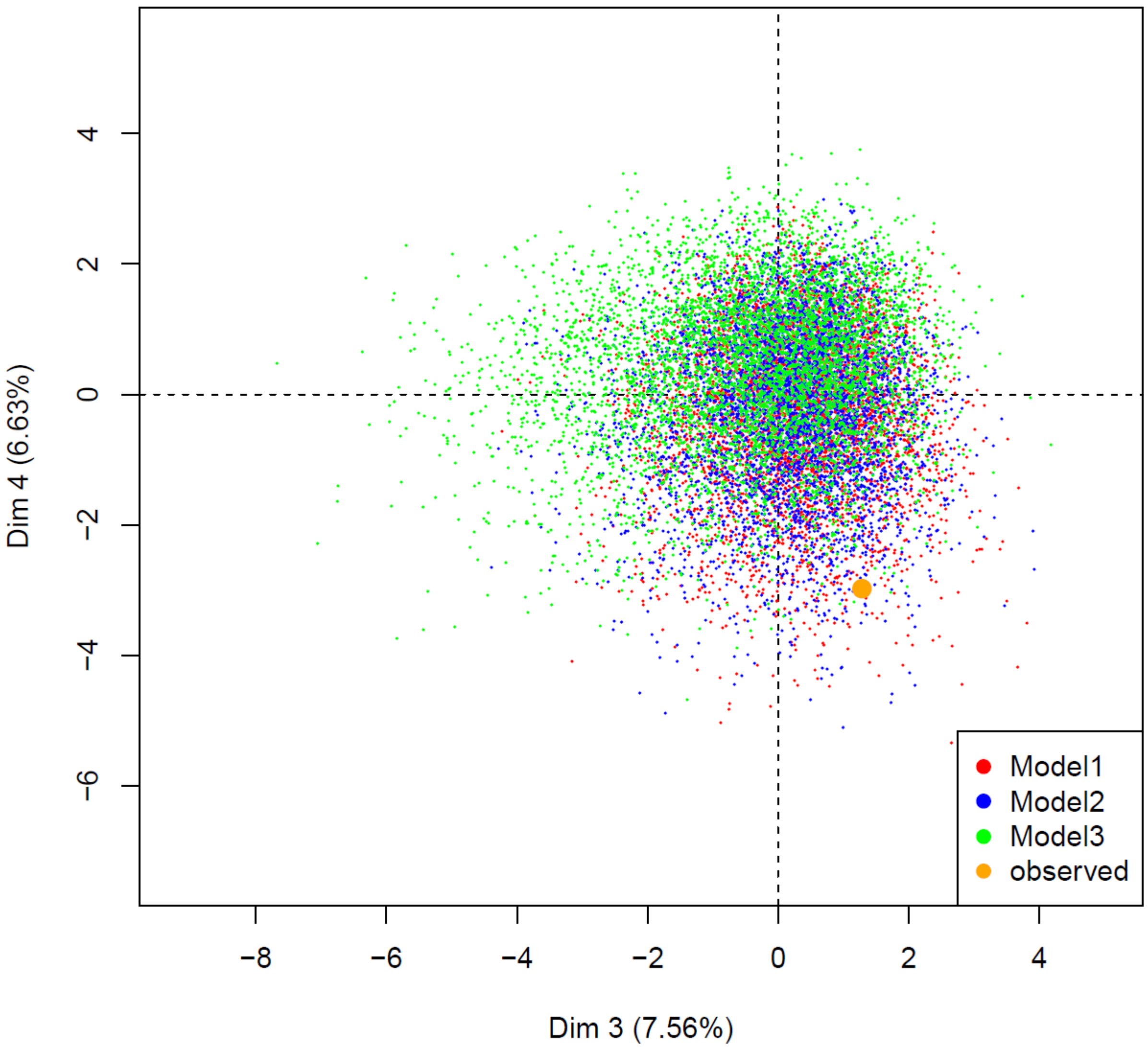

Fig. S7 Principal Component Analysis (PCA) plots reveal the fit between the simulated and observed data generated by the best 1,000 (or 5,000) simulations for each model. Model 1, Model 2, and Model 3 correspond to models of demic diffusion, admixture and cultural diffusion, respectively.
