# Supplementary material for: Complete mitochondrial genomes of Thai and Lao populations indicate an ancient origin of Austroasiatic groups and demic diffusion in the spread of Tai–Kadai languages
Source: Hum Genet. 2016 Nov 11;136(1):85–98. doi: 10.1007/s00439-016-1742-y (PMC5214972; doi:10.1007/s00439-016-1742-y)
Supplement: Supplementary file 4 — Supplementary material 4 (PDF 1093 kb) [file 439_2016_1742_MOESM4_ESM.pdf]

Online Resource 4 is detail of mtDNA haplogroup lineages found in this study.

Focusing on the lineages common in SEA, haplogoups F1, F1a and sublineages thereof exist in most studied populations (14.91%) with the exception of five populations, i.e. KM3, KM10, PU1, MO5 and LW1 (see Fig. 1 for population abbreviations). It is interesting that MO5 has no F1 lineages, indicating a non-SEA origin, consistent with haplogroups of likely South Asian origin in MO5. The F1a1a subclade network, which is also a major lineage in multiple MSEA populations (Jinam et al. 2012; Summerer et al. 2014; Zhang et al. 2013), shows a star-like structure suggesting lineage expansions (Fig. S4 in Online Resource 3). The sporadic occurrence of F1a1a is observed in ISEA populations (Hill et al. 2006; Thangaraj et al. 2005). The age of F1a1a was estimated at ~10.07 kya (Fig. S3 in Online Resource 3) which is similar to previous estimates from Malaysian aborigines (Jinam et al. 2012; Hill et al. 2006, indicating the expansion of this lineage across a vast area of SEA during the early Holocene. However, the age of F1a1a in this study is much lower than an estimate from the coding region in Cambodian populations of ~ 36.73 kya (Zhang et al. 2013). It might be possibly resulted by differences methods of age estimation for this haplogrop. The other subclade of F1a is F1a1d (1.38%) which is restricted to the TK populations, especially the BT and PU groups. F1a1d, with an estimated age of ~6.67 kya (Fig. S3), also occurs in many AN populations from Taiwan (Loo et al. 2011; Ko et al. 2014), indicating genetic connections among these TK and AN groups.

F1f, which is rarely observed elsewhere, is present at a frequency of 5.27% in the AA and TK populations, with a high prevalence in some northern Thai AA groups, i.e. Lawa (21.43%) and Blang (24.00%). The network of F1f indicates the core node is largely localized in northern Thailand with evidence of lineage expansion (Fig. S4), while the F1f contour map indicates high frequency in the area further north (Fig. S5 in Online Resource 3). We propose that F1f originated and diverged ~12.51 kya (Fig. S3) in the area of northern Thailand and southern China, where its sister clades, i.e. F1a, F1b and F1c, are also suggested to have originated (Hill et al., 2007). The F1f MCC tree shows the divergence of five clades during the early Holocene (Fig. S3).

B5a, the second most common haplogroup (12.97%), is also common in several MSEA populations (Bodner et al. 2011; Peng et al. 2010; Zhang et al. 2013) and southern Chinese minorities (Wen et al. 2005; Li et al. 2007). This haplogroup has a patchy distribution in AN

groups from Taiwan (Ko et al. 2014), Malaysia (Hill et al. 2006) and Indonesia (Hill et al. 2007; Gunnarsdóttir et al. 2011b), as well as minorities in Myanmar (Summerer et al. 2014), and occurs rarely in the Philippines (Delfin et al. 2014) and northern Asia (Derenko et al. 2007). B5a coalesces ~20.16 kya (Fig. S3) and bifurcates into two clades, the B5a1d clade and a clade encompassing B5a1*, B5a1a, B5a1b1, and B5a1c (Fig. S3). Interestingly, B5a1d is predominant in SK (57.69%) and KA (40.00%) groups in our study and also in Cambodian Suay (41.61%) and Cambodian Laos (15.38%) from a previous study (Zhang et al. 2013), but is rarely found elsewhere. The Suay and Laos constitute the vast majority in southern Laos, although some of them live as a minority group in Cambodia. Laos is also the homeland for SK and KA groups prior to their recent movement to Thailand (Schliesinger 2000, 2001). We therefore suggest that B5a1d, which arose ~8.70 kya (Fig. S3), is autochthonous to the area of present-day Laos, which is also reflected in the contour map (Fig. S5). Lineage expansions are also detected in the B5a1a and B5a1d networks (Fig. S4).

Several B4 sublineages were detected, i.e. B4 (16261), B4a, B4b, B4c, B4e, B4g and B4h, with B4b1a2a, B4a1c4 and B4c2 as the three most common lineages (Fig. S3). B4 bifurcates into two major ancient clades ~34.81 kya (Fig. S3) and all three common sublineages originated ~9.06-10.62 kya (Fig. 4 and Fig. S3). B4 is almost entirely restricted to TK populations (except for three AA samples from TN3 and SO, which may reflect recent gene flow). Previous studies reported the sporadic occurrence of B4c2 in populations from Taiwan[7] and ISEA (Gunnarsdóttir et al. 2011a; Jinam et al. 2012), but a higher frequency is observed in populations from MSEA (Peng et al. 2010; Zhang et al. 2013). B4b1a2 has only been reported previously in the Cambodian Lao (Zhang et al. 2013) and sporadically elsewhere from MSEA (Kong et al. 2003) and ISEA (Jinam et al. 2012). Networks of B4c2 and B4b1a2a reflect population expansions and the absence of shared sequences suggests no recent gene flow (Fig. S4); we therefore suggest an ancient genetic link between MSEA and ISEA during the early Holocene, in agreement with a previous study (Jinam et al. 2012). B4a1c4 has only been previously reported in one Thai sample (Soares et al. 2011), so this haplogroup is restricted to TK speaking groups in Thailand. In keeping with previous studies (Summerer et al. 2014; Zhang et al. 2013) we do not detect B4a1a1a, which is associated with the Austronesian expansion through Near and Remote Oceania (Duggan et al. 2014); the AN expansion to Oceania therefore did not involve populations from MSEA. We also do not detect several other sublineages of B4

(e.g., B4b1a2h, B4b1a2f, B4b1a2g, B4b1a2b, B4b1a2c and B4b1a2d) which are common in Taiwan and/or the Philippines (Gunnarsdóttir et al. 2011a; Ko et al. 2014), further attesting to the distinct maternal histories of MSEA and ISEA.

M7 has the third highest frequency in the current study, accounting for 10.86% of the samples. This haplogroup is common in the Han and Japanese and MSEA populations (Peng et al. 2010; Summerer et al. 2014; Zhang et al. 2013). Two subclades of M7, namely M7b and M7c (Fig. 4), are found in the studied populations at frequencies of 8.59% and 2.27%, respectively. These two subclades diverged ~50.28 kya (Fig. 4 and Fig. S3); we found ten sublineages of M7b (all within M7b1) that began diverging ~16.72 kya (Fig. S3). The youngest clade is M7b1a1e1 (~5.93 kya: Fig. S3) which shows a high frequency in IS3 (28.00%). M7b1a1 is the most common of these in our populations (8.43%), and has a higher frequency in TK (6.97%) than AA groups (1.46%). M7b1a1 has been reported to be elevated in populations from China, Japan and MSEA (Chen et al. 2015; Kong et al. 2003; Meng et al. 2015; Soares et al. 2016; Tanaka et al. 2004). We also observe a new sublineage of M7b1a1, namely M7b1a1-16192T, at a frequency of 1.20%. This sublineage diverged ~11.18 kya and is restricted only to TK speaking populations. It should be noted that M7b lineages are not found in the MO1-MO4 groups and there are only two M7b samples in the MO5 population, attesting to the different maternal genetic history of the Mon compared to other SEA populations. M7c is proposed to have originated in southern China (Kong et al. 2011). The present study shows two main subclades of M7c (M7c1 and M7c2) that coalesce ~30.54 kya (Fig. S3). M7c1 is also common in TK groups (observed in only 3 AA samples) while M7c2 is restricted to TK groups. M7c1 has been sporadically observed in southern Chinese populations, Hainan Islander and Tsou from Taiwan (Gan et al. 2008; Ko et al. 2014; Kong et al. 2011; Peng et al. 2010) and thus could be a genetic linkage between the TK and southern Chinese populations. M7c2 has been sporadically found in East Asian populations (Derenko et al. 2007; Jinam et al. 2012; Kong et al. 2006; Loo et al. 2014). In addition, we do not find M7c3c, which is associated with the spread of Austronesian speakers (Ko et al. 2014; Peng et al., 2010; Trejaut et al. 2005).

Turning now to the less frequent lineages, haplogroup D (overall frequency of 4.62%) is composed of two lineages, D4 (frequency 4.21%) and D5 (frequency 0.40%) which diverged ~34.84 kya (Fig. S3). D4 occurs throughout East Asia, including Northeast Asia (Derenko et al.

2012), Japan (Tanaka et al. 2004; Zheng et al. 2011), Tibet (Ji et al. 2012), and India (Chandrasekar et al. 2009). In this study, we detect D4 in both TK and AA groups, but at high frequency in four AA populations: MO1 (28.00%), MO5 (31.38%), LW1 (22.72%) and PL (20.00%) (Fig. 1). The D4 lineage shows a signature of an expansion (Fig. S5). B6a, which arose ~34.42 kya (Fig. S3) and is sporadically found in China (Kong et al. 2003), the Philippines (Tabbada et al. 2010), and Malaysia (Jinam et al. 2012), is present at low frequency in MSEA (1.80%; previous study (Summerer et al. 2014) and 2.80%; the current study). The network of B6a with sequences outside Thailand (Fig. S4) indicates that the center of this lineage is possibly located in the area of present-day Thailand and then spread throughout SEA. The contour map also suggests an origin in the area of northern Thailand (Fig. S5). Surprisingly, B6a is at extremely high frequency (68.00%) in the H'tin subgroup Mal (TN1), which probably reflects genetic drift (Fig. S5).

We observe a patchy distribution of R9b and R22 (overall frequencies of 2.80% and 1.86%, respectively). R9b is distributed in both MSEA and ISEA and possibly originated in MSEA and spread to ISEA during the Austronesian expansion (Hill et al. 2006; Macaulay et al. 2005). The TN2 group shows a particularly high frequency of R9b2 (32.00%), which probably reflects genetic drift. We estimate the coalescent age of R9b at ~38.67 kya (Fig. S3), considerably older than a previous estimate of 29.00 kya (Hill et al. 2006), which probably reflects the increased sampling of R9b from our study. Therefore, we support the place of origin of R9b in MSEA (Hill et al. 2006), most likely in the area of present-day northeastern Thailand and Laos. R22 is found in the Nicobar Islands and ISEA (Hill et al. 2007; Simonson et al. 2011; Trivedi et al. 2006) and also in MSEA, i.e. Vietnam (Peng et al. 2010) and Cambodia (Zhang et al. 2013). R22 is considered to be an ancient maternal lineage of SEA (Hill et al. 2007) and the estimated age in the present study is ~39.21 kya (Fig. S3) which is much older than previous estimates of 29.80 kya (Hill et al. 2007) and 19.00 kya (Zhang et al. 2013). R22 is at highest frequency in northeastern Thai populations (Fig. S5), especially the BO (17.39%) and SU (25.00%) groups (Fig. 1); we therefore suggest that R22 is likely to have arisen in the region of northeastern Thailand.

We found six sublineages of N9a (overall frequency of 2.50%) that do not show signatures of lineage expansion (Fig. S4). N9a has been suggested to be a native lineage in East

Asian populations and is prevalent in northeast Asia (4.60-8.00%) (Derenko et al. 2007; Tanaka et al. 2004). It is less prevalent (frequencies of 1.00-4.50%) in populations from MSEA and southwestern China (Bpdner et al. 2011; Chandrasekar et al. 2009; Peng et al. 2010; Zhang et al. 2013) and rare in ISEA populations (Hill et al. 2007), except for N9a6. N9a6 was previously found only in ISEA with an estimated age of ~17.20 kya (Jinam et al. 2012), compared to our estimate of ~12.05 kya (Fig. S3). N9a10 was previously found in Atayal, Payuma and Minnan from Taiwan (2.00-6.00%) (Ko et al. 2014; Trejaut et al. 2005) with an estimated age of ~14.88-17.31 kya (Loo et al. 2011), similar to our estimate of ~17.05 kya (Fig. S3). N9a10 (16311C), the most common subclade of N9a and very limited elsewhere (Ji et al., 2012), is found in TK groups with an age of ~13.74 kya (Table 2 and Fig. S3). N10 (frequency 0.65%) is the second oldest lineage in this study with an age of ~52.03 kya (Fig. S3) which is lower than a previous estimate of 66.4 kya (Fregel et al. 2015) but much older and younger than a previous estimate (21.33 and 63.44 kya) depending the methods used (Kong et al. 2011). The major sublineage of N10 is N10a, with an age of ~11.31 kya. N10a was previously detected mainly in Hani, Yi, and Han Chinese from southern China, suggesting an origin in southern China (Kong et al. 2011). N10a was found at high frequency (12.5%) in the LW3 group, providing evidence for a northern source contributing to this population. LW3 also has a high frequency of haplogroup A (37.50%) which was previously reported to be specific to North and Central Asia (Derenko et al. 2007). The coalescent time of A is ~ 24.40 kya (Fig. S3), similar to previous estimates (Fregel et al. 2015). There are 4 sublineages of A in our data, of which A14 and A17 are the most common. There are no previous reports of A14 and A17 in MSEA.

CZ, which is derived from M8, occurred at a frequency of 3.08%. C7 is the most common sublineage (2.59%), while C4 and Z are found with lower frequency. The estimated age of C7 lineage ~18.59 kya (Fig. S3). The KM1, KM2 and MO2 groups have high frequencies of C7 (16.00%, 25.00%, 17.39%, respectively; Table S1 in Online Resource 1). C7 is reported to be common in eastern Asian and northeastern Indian populations with an age of ~26.00 to 28.00 kya (Derenko et al. 2010).

M12 and sublineages thereof are older than 20.00 kya (Fig. S3). The TN3 group has a high frequency of M12a1a (28.00%), which is the most common subclade of M12 and was previously reported to be specific to Hainan Island (Peng et al. 2010). M74 is found at a frequency of 2.59% with an estimated coalescence age of ~34.86 kya for the sublineages M74a

(0.32%) and M74b (2.11%) (Fig. S3). This estimated age is younger than one previous estimate of ~52.30 kya (Jinam et al. 2012), while the other estimates from southern Chinese and MSEA populations ranged from 22.83 to 43.80 kya depending on the methods used (Kong et al. 2011; Zhang et al. 2013). M74b is at high frequency in the BO group (21.74%). Both M74a and M74b have been reported at low frequency in Cambodia (Zhang et al. 2013) but the origin of this haplogroup was suggested to be in southern China (Kong et al. 2006). Another basal haplogroup at high frequency in BO (26.08%) is M51, with an estimated age of ~30.00 kya. M51 occurs at high frequency in the Jenu Kuruba (29.00%) of southern India and sporadically in the Hill Kolam (1.30%) of central India, Tharu (2.50-4.20%) of Nepal, Phnong (12.70%) and Stieng (8.00%) of Cambodia, and Cham (1.19%) of Vietnam (Chandrasekar et al. 2009; Fornarino et al. 2009; Peng et al. 2010; Zhang et al. 2013). M51 is suggested to represent an ancient maternal component in this region (Peng et al. 2010).

M20 (frequency = 2.35%) is at high frequency in the LW2 group (25.00%) and sporadically in many of the studied populations; it has been rarely reported elsewhere (Jinam et al. 2012; Kong et al. 2011). Our estimate of the age of M20 of ~12.22 kya (Fig. S3) is older than a previous estimate of ~8.50 kya (Jinam et al. 2012), and there is a signal of population expansion (Fig. S5). M24 (frequency = 1.70%) reaches a frequency of 40% in the KH2 group, and has been reported in high frequency in the Naga (28.13%) and lower frequency in Burmese and Rakhine (1.00-3.00%) (Li et al. 2015). Our estimate of the age of M24 is ~19.30 kya, which is similar to a previous estimate of ~20.39 kya (Li et al. 2015).

Finally, haplogroup W, which was previously observed in India (Olivieri et al. 2013; Palanichamy et al. 2004) but not in MSEA, was detected as subhaplogroup W3a1b in two Mon populations (24.00% in MO1, 4.35% in MO2). This further supports an origin of these groups in India and/or gene flow from Indian populations. The age of W3a1b is ~13.41 kya, which falls in the same range as the sister clade W3a1a (~15.60 kya) from a previous study (Olivieri et al. 2013), reflecting the divergence of W3a1 during the late Pleistocene. Many other lineages show sporadic frequencies across the studied groups (Fig. 1; Table S1), with AA populations tending to show the greatest fluctuations.

References

Bodner M, Zimmermann B, Röck A, Kloss-Brandstätter A, Horst D, Horst B, Sengchanh S, Sanguansermsri T, Horst J, Krämer T, Schneider PM, Parson W (2011) Southeast Asian diversity: first insights into the complex mtDNA structure of Laos. BMC Evol Biol 11:49

Chandrasekar A, Kumar S, Sreenath J, Sarkar BN, Urade BP, Mallick S, Bandopadhyay SS, Pinuma Barua P, Barik SS, Basu D, Kiran U, Gangopadhyay P, Sahani R, Prasad BVR, Gangopadhyay S, Lakshmi GR, Ravuri RR, Padmaja K, Venugopal PN, Sharma MB, Rao VR (2009) Updating phylogeny of mitochondrial DNA macrohaplogroup M in India: dispersal of modern human in south Asian corridor. PLoS One 4:e7447

Chen F, Yin CY, Qian XQ, Fan HT, Deng YJ, Zhang YD, Meng HT, Shen CM, Yang CH, Jin R, Zhu BF, Xu P (2015) Single nucleotide polymorphisms of mitochondrial DNA HVS-I and HVS-II in Chinese Bai ethnic group. Electrophoresis 36:930-936

Delfin FS, Min-Shan KA, Li M, Gunnarsdóttir ED, Tabbada KA, Salvador JM, Calacal GC, Sagum MS, Datar FA, Padilla SG, De Ungria MC, Stoneking M (2014) Complete mtDNA genomes of Filipino ethnolinguistic groups: A melting pot of recent and ancient lineages in the Asia-Pacific region. Eur J Hum Genet 22:228-237

Derenko M, Malyarchuk B, Grzybowski T, Denisova G, Dambueva I, Perkova M, Dorzhu C, Luzina F, Lee HK, Vanecek T, Villems R, Zakharov I (2007) Phylogeographic analysis of mitochondrial DNA in Northern Asian populations. Am J Hum Genet 81:1025-1041

Derenko M, Malyarchuk B, Grzybowski T, Denisova G, Rogalla U, Perkova M, Dambueva I, Zakharov I (2010) Origin and post-glacial dispersal of mitochondrial DNA haplogroups C and D in Northern Asia. PLoS One 5:e15214

Derenko M, Malyarchuk B, Denisova G, Perkova M, Rogalla U, Grzybowski T, Khusnutdinova E, Dambueva I, Zakharov I (2012) Complete mitochondrial DNA analysis of Eastern Eurasian haplogroups rarely found in populations of Northern Asia and Eastern Europe. PLoS One 7:e32179

Duggan A, Evans B, Friedlaender FR, Friedlaender JS, Koki G, Merriwether DA, Kayser M, Stoneking M (2014) Maternal history of Oceania from complete mtDNA genomes: contrasting ancient diversity with recent homogenization due to the Austronesian expansion. Am J Hum Genet 94(5):721-733

Fornarino S, Pala M, Battaglia V, Maranta R, Achilli A, Modiano G, Torroni A, Semino O, Santachiara-Benerecetti SA (2009) Mitochondrial and Y-chromosome diversity of the Tharus (Nepal): a reservoir of genetic variation. BMC Evol Biol 9:154

Fregel R, Cabrera V, Larruga JM, Abu-Amero KK, González AM (2015) Carriers of mitochondrial DNA macrohaplogroup N lineages reached Australia around 50,000 years ago following a northern Asian route. PLoS One 10:e0129839

Gan RJ, Pan SL, Mustavich LF, Qin ZD, Cai XY, Qian J, Liu CW, Peng JH, Li SL, Xu JS, Jin L, Li H (2008) Pinghua population as an exception of Han Chinese's coherent genetic structure. J Hum Genet 53:303-313

Gunnarsdóttir ED, Li M, Bauchet M, Finstermeier K, Stoneking M (2011a) High throughput sequencing of complete human mtDNA genomes from the Philippines. Genome Res 21:1-11

Gunnarsdóttir ED, Nandineni MR, Li M, Myles S, Gil D, Pakendorf B, Stoneking M (2011b) Larger mitochondrial DNA than Y-chromosome differences between matrilocal and patrilocal groups from Sumatra. Nat Commun 2:228

Hill C, Soares P, Mormina M, Macaulay V, Clarke D, Blumbach PB, Vizuete-Forster M, Forster P, Bulbeck D, Oppenheimer S, Richards M (2007) A mitochondrial stratigraphy for island southeast Asia. Am J Hum Genet 80:29-43

Hill C, Soares P, Mormina M, Macaulay V, Meehan W, Blackburn J, Clarke D, Raja JM, Ismail P, Bulbeck D, Oppenheimer S, Richards M (2006) Phylogeography and ethnogenesis of aboriginal Southeast Asians. Mol Biol Evol 23:2480-2491

Ji F, Sharpley MS, Derbeneva O, Alves LS, Qian P, Wang Y, Chalkia D, Lvova M, Xu J, Yao W, Simon M, Platt J, Xu S, Angelin A, Davila A, Huang T, Wang PH, Chuang LM, Moore LG, Qian G, Wallace DC (2012) Mitochondrial DNA variant associated with

Leber hereditary optic neuropathy and high-altitude Tibetans. Proc Natl Acad Sci USA 109:7391-7396

Jinam TA, Hong LC, Phipps ME, Stoneking M, Ameen M, Edo J, HUGO Pan-Asian SNP Consortium, Saitou N (2012) Evolutionary history of continental Southeast Asians: “early train” hypothesis based on genetic analysis of mitochondrial and autosomal DNA data. Mol Biol Evol 29:3513-3527

Ko AM, Chen CY, Fu Q, Delfin F, Li M, Chiu HL, Stoneking M, Ko YC (2014) Early Austronesians: Into and out of Taiwan. Am J Hum Genet 94:426-436

Kong QP, Yao YG, Sun C, Bandelt HJ, Zhu CL, Zhang YP (2003) Phylogeny of East Asian mitochondrial DNA lineages inferred from complete sequences. Am J Hum Genet 73:671-676

Kong QP, Bandelt HJ, Sun C, Yao YG, Salas A, Achilli A, Wang CY, Zhong L, Zhu CL, Wu SF, Torroni A, Zhang YP (2006) Updating the East Asian mtDNA phylogeny: a prerequisite for the identification of pathogenic mutations. Hum Mol Genet 15(13):2076-2086

Kong QP, Sun C, Wang HW, Zhao M, Wang WZ, Zhong L, Hao XD, Pan H, Wang SY, Cheng YT, Zhu CL, Wu SF, Liu LN, Jin JQ, Yao YG, Zhang YP (2011) Large-scale mtDNA screening reveals a surprising matrilineal complexity in East Asia and its implications to the peopling of the region. Mol Biol Evol 28:513-522

Li H, Cai X, Winograd-Cort ER, Wen B, Cheng X, Qin Z, Liu W, Liu Y, Pan S, Qian J, Tan CC, Jin L (2007) Mitochondrial DNA diversity and population differentiation in southern East Asia. Am J Phys Anthropol 134:481-488

Li YC, Wang HW, Tian JY, Liu LN, Yang LQ, Zhu CL, Wu SF, Kong QP, Zhang YP (2015) Ancient inland human dispersals from Myanmar into interior East Asia since the Late Pleistocene. Sci Reports 5:9473

Loo JH, Trejaut JA, Yen JC, Chen ZS, Lee CL, Lin M (2011) Genetic affinities between the Yami tribe people of Orchid Island and the Philippine Islanders of the Batanes archipelago. BMC Genet 12:21

Loo JH, Trejaut JA, Yen JC, Chen ZS, Ng WM, Huang CY, Hsu KN, Hung KH, Hsiao Y, Wei YH, Lin M (2014) Mitochondrial DNA association study of type 2 diabetes with or without ischemic stroke in Taiwan. BMC Res Notes 7:223

Macaulay V, Hill C, Achilli A, Rengo C, Clarke D, Meehan W, Blackburn J, Semino O, Scozzari R, Cruciani F, Taha A, Shaari NK, Raja JM, Ismail P, Zainuddin Z, Goodwin W, Bulbeck D, Bandelt HJ, Oppenheimer S, Torroni A, Richards M (2005) Single, rapid coastal settlement of Asia revealed by analysis of complete mitochondrial genomes. Science 308:1034-1036

Meng JH, Yao J, Xing JX, Xuan JF, Wang BJ, Ding M (2015) Investigation of control region sequences of mtDNA in a Chinese Maonan population. Mitochondrial DNA 29:1-5

Olivieri A, Pala M, Gandini F, Kashani BH, Perego UA, Woodward SR, Grugni V, Battaglia V, Semino O, Achilli A, Richards MB, Torroni A (2013) Mitogenomes from two uncommon haplogroups mark late glacial/postglacial expansions from the Near East and Neolithic dispersals within Europe. PLoS One 8:e70492

Palanichamy MG, Sun C, Agrawal S, Bandelt HJ, Kong QP, Khan F, Wang CY, Chaudhuri TK, Palla V, Zhang YP (2004) Phylogeny of mitochondrial DNA macrohaplogroup N in India, based on complete sequencing: implications for the peopling of South Asia. Am J Hum Genet 75(6):966-978

Peng MS, Quang HH, Dang KP, Trieu AV, Wang HW, Yao YG, Kong QP, Zhang YP (2010) Tracing the Austronesian footprint in mainland Southeast Asia: a perspective from mitochondrial DNA. Mol Biol Evol 27:2417-2430

Schliesinger J (2000) Ethnic groups of Thailand: non-Tai speaking peoples. White Lotus Press, Bangkok

Schliesinger J (2001) Tai group of Thailand, Volume 1: Introduction and overview. White Lotus Press, Bangkok

Simonson TS, Xing J, Barrett R, Jerah E, Loa P, Zhang Y, Watkins WS, Witherspoon DJ, Huff CD, Woodward S, Mowry B, Jorde LB (2011) Ancestry of the Iban is predominantly

Southeast Asia: genetic evidence from autosomal, mitochondrial, and Y chromosomes. PLoS One 6(1):e16338

Soares P, Rito T, Trejaut J, Mormina M, Hill C, Tinkler-Hundal E, Braid M, Clarke DJ, Loo JH, Thomson N, Denham T, Donohue M, Macaulay V, Lin M, Oppenheimer S, Richards MB (2011) Ancient voyaging and polynesian origins. Am J Hum Genet 88(2):239-247

Soares P, Trejaut JA, Rito T, Cavadas B, Hill C, Eng KK, Mormina M, Brandão A, Fraser RM, Wang TY, Loo JH, Snell C, Ko TM, Amorim A, Pala M, Macaulay V, Bulbeck D, Wilson JF, Gusmão L, Pereira L, Oppenheimer S, Lin M, Richards MB (2016) Resolving the ancestry of Austronesian-speaking populations. Hum Genet 135(3):309-326

Summerer M, Horst J, Erhart J, Weißensteiner H, Schönherr S, Pacher D, Forer L, Horst D, Manhart A, Horst B, Sanguansermsri T, Brandstätter AK (2014) Large-scale mitochondrial DNA analysis in Southeast Asia reveals evolutionary effects of cultural isolation in the multi-ethnic population of Myanmar. BMC Evol 14:17

Tabbada KA, Trejaut J, Loo JH, Chen YM, Lin M, Mirazón-Lahr M, Kivisild T, De Ungria MC (2010) Philippine mitochondrial DNA diversity: a populated viaduct between Taiwan and Indonesia. Mol Biol Evol 27:21-31

Tanaka M, Cabrera VM, González AM, Larruga JM, Takeyasu T, Fuku N, Guo LJ, Hirose R., Fujita, Y, Kurata M, Shinoda K, Umetsu K, Yamada Y, Oshida Y,Sato Y, Hattori N, Mizuno Y, Arai Y, Hirose N, Ohta S, Ogawa O, Tanaka Y, Kawamori R, Shamoto-Nagai M, Maruyama W, Shimokata H, Suzuki R,Shimodaira H (2004) Mitochondrial genome variation in Eastern Asia and the peopling of Japan. Genome Res 14:1832-1850

Thangaraj K, Chaubey G, Kivisild T, Reddy AG, Singh VK, Rasalkar AA, Singh L (2005) Reconstructing the origin of Andaman Islanders. Science 308:996

Trejaut JA, Kivisild T, Loo JH, Lee CL, He CL, Hsu CJ, Li ZY, Lin M (2005) Traces of archaic mitochondrial lineages persist in Austronesian-speaking Formosan populations. PLoS Biol 3:e247

Trivedi R, Sitalaximi T, Banerjee J, Singh A, Sircar PK, Kashyap VK (2006) Molecular insights into the origins of the Shompen, a declining population of the Nicobar archipelago. J Hum Genet 51:217-226

Wen B, Li H, Gao S, Mao X, Gao Y, Li F, Zhang F, He Y, Dong Y, Zhang Y, Huang W, Jin J, Xiao C, Lu D, Chakraborty R, Su B, Deka R, Jin L (2005) Genetic structure of Hmong-Mien speaking populations in East Asia as revealed by mtDNA lineages. Mol Biol Evol 22:725-733

Zhang X, Qi X, Yang Z, Serey B, Sovannary T, Bunnath L, Aun HS, Samnom H, Zhang H, Lin Q, Oven MV, Shi H, Su B (2013) Analysis of mitochondrial genome diversity identifies new and ancient maternal lineages in Cambodian aborigines. Nat Commun 4:2599

Zheng HX, Yan S, Qin ZD, Wang Y, Tan JZ, Li H, Jin L (2011) Major population expansion of East Asians began before Neolithic time: evidence of mtDNA genomes. PLoS One 6:e25835
